# Common differentially expressed genes and pathways correlating both coronary artery disease and atrial fibrillation

**DOI:** 10.17179/excli2020-3262

**Published:** 2021-01-18

**Authors:** Youjing Zheng, Jia-Qiang He

**Affiliations:** 1 Department of Biomedical Sciences and Pathobiology, College of Veterinary Medicine, Virginia Tech, Blacksburg, VA 24061, USA

**Keywords:** coronary artery disease, atrial fibrillation, differentially expressed gene, signaling pathway, bioinformatics

## Abstract

Coronary artery disease (CAD) and atrial fibrillation (AF) share common risk factors, such as hypertension and diabetes. The patients with CAD often suffer concomitantly AF, but how two diseases interact with each other at cellular and molecular levels remain largely unknown. The present study aims to dissect the common differentially expressed genes (DEGs) that are concurrently associated with CAD and AF. Two datasets [GSE71226 for CAD) and GSE31821 for AF] were analyzed with GEO2R and Venn Diagram to identify the DEGs. Signaling pathways, gene enrichments, and protein-protein interactions (PPI) of the identified common DEGs were further analyzed with Kyoto Encyclopedia of Gene and Genome (KEGG), Database for Annotation, Visualization and Integrated Discovery (DAVID), and Search Toll for the Retrieval of Interacting Genes (STRING). 565 up- and 1367 down-regulated genes in GSE71226 and 293 up- and 68 down-regulated genes in GSE31821 were identified. Among those, 21 common DEGs were discovered from both datasets, which lead to the findings of 4 CAD and 21 AF pathways, 3 significant gene enrichments (intracellular cytoplasm, protein binding, and vascular labyrinthine layer), and 3 key proteins (membrane metallo-endopeptidase (MME), transferrin receptor 1 (TfR1), and Lysosome-associated membrane glycoprotein 1 (LAMP1)). Together, these data implied that these three proteins may play a central role in development of both CAD and AF.

## Introduction

Cardiovascular disease is the leading cause of death in the developed countries (Virani et al., 2020[45]). Of all cardiovascular diseases (*e.g.,* acute myocardial infarction, heart failure, valvular heart disease, cerebrovascular accident, transient ischemic attack, peripheral arterial disease, sudden cardiac arrest, ventricular arrhythmia, venous thromboembolism, and pulmonary embolism), coronary artery disease (CAD) is the most common type and contributes the highest rate of death (Michniewicz et al., 2018[28]; Virani et al., 2020[45]); whereas of all cardiac arrhythmia (*e.g.,* supraventricular tachycardia, ventricular tachycardia, sinus-node dysfunction, and heart block), atrial fibrillation (AF) is the most typical disorder, and it affects about 37.6 million individuals globally in 2017 (Go et al., 2001[12]; Michniewicz et al., 2018[28]; Virani et al., 2020[45]). 

Interestingly, studies found that AF is highly associated with the increased risk of many other diseases, such as CAD, stroke, heart failure, diabetes, sudden cardiac death, and mortality, especially within the aging populations (Motloch et al., 2017[30]; Murakami et al., 2017[32]; Virani et al., 2020[45]). In the case of CAD, it was demonstrated that both AF and CAD share the same risk factors and impact on each other (Kristensen et al., 2020[22]; Lieder et al., 2018[25]; Motloch et al., 2017[30]). A systematic review and meta-analysis of 15 cohort studies, for example, demonstrated that AF was associated with a 1.54-fold increased risk of myocardial infarction induced by CAD (Ruddox et al., 2017[40]). Overall, about 17-46.5 % patients with AF suffer concomitantly CAD while the patients with CAD have a low prevalence rate (0.2 % to 5 %) of AF, suggesting the significant effects of AF on promoting morbidity and mortality of concomitant diseases (Michniewicz et al., 2018[28]).

Meanwhile, the outcomes of the patients with CAD is modulated by AF; however, it's still unclear whether the presence of CAD simply increases the risk of AF or changes the impact of other risk factors (Mehta et al., 2003[27]; Pilgrim et al., 2013[34]). The management of AF with concomitant CAD is still a huge clinical challenge (Gladding et al., 2020[11]). Fully understanding the similarities in the pathogenesis of AF and CAD may reveal the mechanisms underlying both diseases and facilitate discovery of new therapy targets. 

Bioinformatic analysis of gene profiles offers a novel approach to explore the underlying mechanisms of disease at the molecular level. This technique has been widely utilized in basic and clinical studies (Kumar et al., 2016[23]), yet only limited data is reported regarding interlinkages of critical genes and signaling molecules between CAD and AF (Kertai et al., 2015[17]). In this paper, we aimed to profile the common differentially expressed genes (DEGs) of CAD and AF by using the sequencing databases of these two diseases and identified potential pathways modulating the development of CAD and AF. 

## Materials and Methods

### Data sources

The datasets of gene of interest with sequence number GSE71226 and GSE31821 were downloaded from the Gene Expression Omnibus (GEO) database (https://www.ncbi.nlm.nih.gov/geo/). In GSE71226 microarray dataset, 3 samples from the patients with CAD and 3 samples of healthy subjects were included; while in GSE31821 dataset, 4 samples from the patients with AF and 2 samples of healthy subjects were enrolled. Both datasets were collected from GPL570 Platforms ((HG-U133_Plus_2) Affymetrix Human Genome U133 Plus 2.0 Array). The detail information is shown in Table 1(Tab. 1).

### Identifications of differentially expressed genes 

DEGs between patients and healthy subjects were identified via GEO2R online tools (log_2_FC > 1 or log_2_FC < -1, p value <0.05) (Davis and Meltzer, 2007[6]). The row data were then run in Venn Diagram (http://bioinformatics.psb.ugent.be/webtools/Venn/) to identify the common DEGs between 2 datasets. The DEGs with log_2_FC < -1 were considered as down-regulated genes, while the DEGs with log_2_FC > 1 were regarded as up-regulated genes. Heatmap of gene expression was made by R package ggplot2 as described previously (Aibar et al., 2015[1]; Walter et al., 2015[46]).

### Gene ontology enrichment analysis

The Gene ontology (GO) analysis has become a common way to analyze large scale genomic data (Zheng et al., 2008[52]). Kyoto encyclopedia of genes and genomes (KEGG) (https://www.genome.jp/kegg/) is a biological genomic database that focuses on computerization of molecular linkage among genomes, gene functions, and biochemical (metabolic and regulatory) pathways of all organisms under normal and disease conditions (Ogata et al., 1999[33]). Database for Annotation, Visualization and Integration Discovery (DAVID) (https://david.ncifcrf.gov/) and Cystoscape software (https://cytoscape.org/) were used for the GO enrichment and KEGG pathway analysis of integrated differential genes. DAVID (v6.8) is an online bioinformatic tool that is designed to identify gene and protein functions and visualize different signaling pathways. In these analyses, bar plots were made by R package heatmap to show the ten most significant enriched GO terms (Aibar et al., 2015[1]; Walter et al., 2015[46]) 

### Protein-protein interaction network mapping 

The online Search Tool for the Retrieval of Interacting Genes (STRING) (https://string-db.org/) was used to analyze the protein-protein interaction (PPI) network of the DEGs as described in the previously published study (Szklarczyk et al., 2015[42]). Since PPI is known to modulate a variety of biological process, such as cellular metabolisms, development processes, and cell-to-cell interactions, thus, it could be used to predict key protein(s) that regulate cellular specific functions or to be screened as potential therapeutic target(s) (Rao et al., 2014[36]). 

## Results

### Up- and down-regulated genes that were concurrently expressed in the patients with CAD and AF

**Figure 1:**
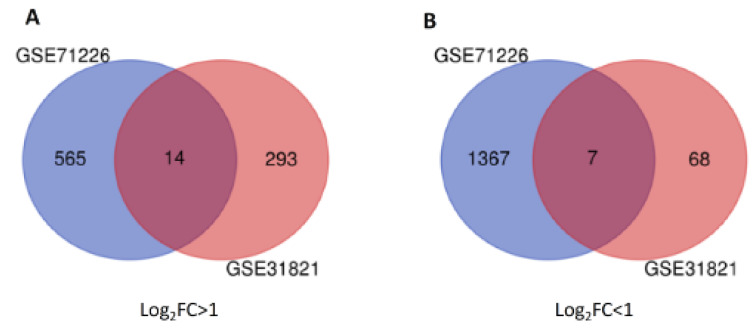
Twenty-one common DEGs from two datasets were identified in Venn Diagram. Panel A shows 14 up-regulated DEGs (at the center) from GSE71226 dataset (in blue) and GSE31821 (in red). Panel B shows 7 down-regulated genes from GSE71226 dataset (in blue) and GSE31821 (in red). DEGs: differentially expressed genes. GSExxxx: gene set enrichment #; Log_2_FC > 1 or < 1: fold changes in logarithms to base 2 between patients and healthy subjects are greater (up-regulated) or lower (down-regulated) than 1.

To outline the profiles of DEGs, two datasets were analyzed with GEO2R software. From GSE71226 dataset of the patients with CAD patients and healthy subjects, a total of 1932 DEGs was identified, among which 565 genes were up-regulated (*p < 0.05*, log_2_FC > 1) and 1367 genes were down-regulated (*p < 0.05*, log_2_FC < -1) (Supplementary Table 2 and Figure 1(Fig. 1)). Similarly, from GSE31821 dataset of the patients with AF and healthy subjects, a total of 361 genes were extracted, among which 293 genes were up-regulated (*p < 0.05*, log_2_FC > 1) and 68 genes were down-regulated (*p < 0.05*, log_2_FC < -1) (Supplementary Table 3 and Figure 1(Fig. 1)), suggesting significantly differential expressions of multitudinous genes in the patients with CAD and AF.

To further determine the common DEGs that exist in both datasets, we ran two datasets on Venn Diagram and confirmed 21 common DEGs, which comprises 14 up-regulated genes (*p < 0.05*, log_2_FC > 1) and 7 down-regulated genes (*p < 0.05*, log_2_FC < -1) (Table 2(Tab. 2) and Figure 1(Fig. 1)). To unveil the expression patterns of the DEGs among all groups, the top 100 DEGs were selected based on the *p*-values (*p < 0.05)*, and constructed as a cluster heatmap to show the cross-correlation of those genes among each individual.

**Figure 2:**
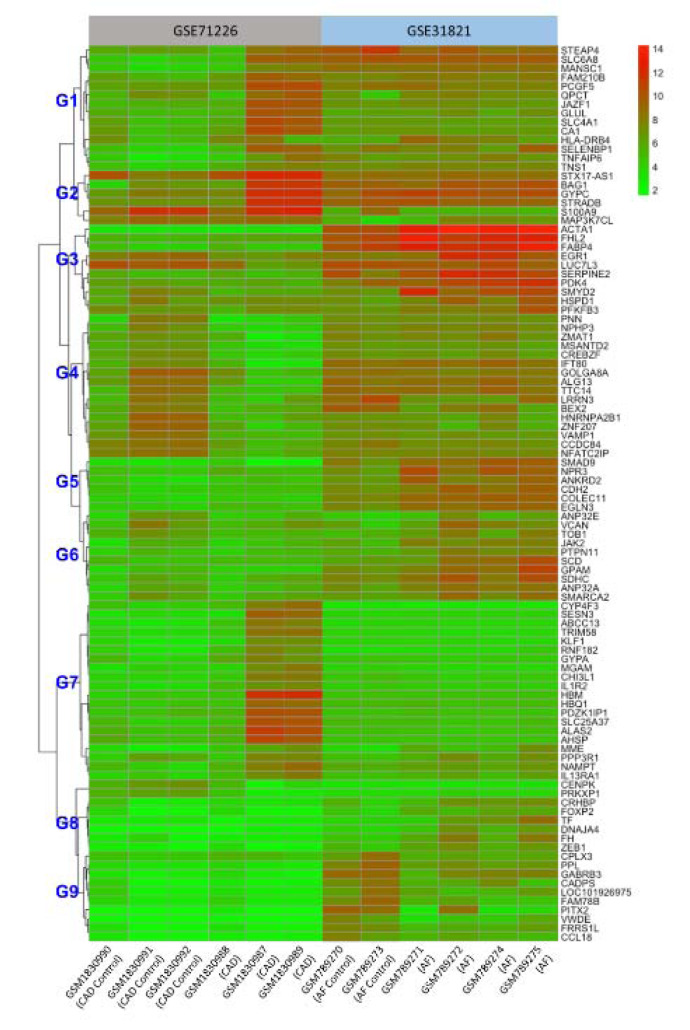
Cluster heatmap of the top 100 DEGs from both GSE71226 and GSE31821 datasets. The colored codes from green to red indicate expression levels from low (in green) to high (in red). The sample identification # (GSM….) is listed on the bottom (x-axis). The left gene tree was roughly grouped into 9 groups (G1 to 9) for easy reference in text. The right side lists all 100 genes. See Figure 1 and Supplementary Table 1 for other abbreviations.

As shown in Figure 2(Fig. 2), there are significant differences of gene expression profiles between healthy subjects and patients. Overall, 5 healthy subjects from two datasets exhibited similar patterns of gene expressions except for group 1 (G1), 3, 5, and 9; while significant differences of expression levels were observed in all groups (G1-9) between 3 CAD patients and 4 AF patients with high expressions in G1, 2, 7 and low expressions in G3, 4, 5, 6, 8, and 9 in the patients with CAD compared to the same groups of the patients with AF. Interestingly, STX17-AS1, BAG1, GYPC, STRADB, S100A9, and HBM are the mostly expressed genes in the CAD patients while ACTA1, FHL2, FABP4, and EGR1 are the mostly expressed genes in the AF patients. The common DEGs between CAD and AF appear in G1 and 2, such as STEAP4, SLC6A8, GYPC, and STRADB. Together, these data demonstrated that majority of the DEGs expressed differently between CAD and AF, but a small group of genes expressed concurrently.

### Variable and common GO terms between CAD and AF

**Figure 3:**
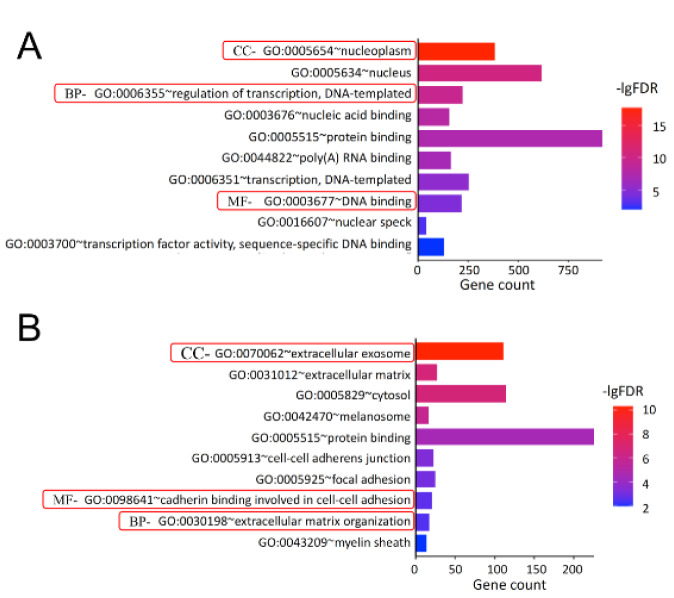
GO enrichment analysis of the DEGs. Panel A shows the results of top 10 GO enrichment in GSE71226 dataset. Panel B shows the results of top 10 GO enrichment GSE31821 dataset. In all panels, X-axis represents counts of the DEGs and Y-axis refers the enriched GO terms (BP, MF, and CC). The colored codes from blue to red indicate significance from low to high. GO: gene ontology; BP: biological process; MF: molecular function; CC: cellular components; -lgFDR: Log_10_ false discovery rate (*p*-value). See Figure 1, 2 and Supplementary Table 1 for other abbreviations.

To characterize the three critical terms, biological process (BP), molecular function (MF), and cellular component (CC) of the DEGs identified above, the GO (*i.e.,* over-representation or term enrichment) enrichment analysis was performed on two datasets and the results (*i.e.,* terms) are presented as graph (ontology) structure shown in Figure 3(Fig. 3). 

From GSE71226 dataset of the patients with CAD and healthy subjects, it was found that for the BP term, the DEGs were mostly enriched in the regulation of transcription (GO: 0006355, *p *= 1.91E-13); while for MF term, they were mostly enriched in the DNA binding (GO:0003676, *p *= 5.75E-12); and lastly for CC term, they were mostly enriched in the nucleoplasm (GO: 0005654, *p *= 1.24E-21) (Table 3(Tab. 3) and Figure 3A(Fig. 3)). Similarly, from GSE31821 dataset of the patients with AF and healthy subjects, it was found that for the BP term, the DEGs were mostly enriched in the extracellular matrix (GO:0030198, *p *= 2.07E-06); while for MF term, they were mostly enriched in the cadherin binding in cell-cell adhesion (GO:0098641, *p *= 1.62E-06), and lastly for CC term, they were mostly enriched in the extracellular exosome (GO: 0070062, *p *= 4.89E-11) (Table 3(Tab. 3) and Figure 3B(Fig. 3)). Together, these data demonstrated that the DEGs identified from the patients with CAD or AF were expressed (enriched) differentially in the aspects of the BP, MF, and CC. 

However, when the 21 common DEGs were analyzed, the resulting GO terms are different from the above. Specifically, for the BP, the common DEGs were particularly enriched in the regulation of labyrinthine layer in embryonic blood vessel; while for the MF, they were remarkably enriched in the protein binding, and lastly for the CC, they were substantially enriched in the intracellular cytoplasm (Table 4(Tab. 4)), implying that those terms (BP, MF, and CC) may represent the common pathogenesis in the development of CAD and AF. 

### Numerous but not common pathways were detected in both CAD and AF

Next, we used DAVID software to map the KEGG pathways of the identified DEGs from both datasets. Briefly, from GSE71226 dataset of the patients with CAD and healthy subjects, four key pathways were determined: 1) mRNA surveillance pathway; 2) eukaryotic ribosome biogenesis pathway; 3) glucagon signaling pathway; and 4) other types of O-glycan biosynthesis (Table 5(Tab. 5)). However, from GSE31821 dataset of the patients with AF and healthy subjects, twenty-one key pathways were discovered, including 1) Focal adhesion; 2) MAPK; 3) Amoebiasis; 4) Cancer; 5) PI3K-Akt; 6) Wnt signaling; 7) ECM-receptor interaction; 8) Platelet activation; 9) Toxoplasmosis; and 10) Proteoglycans in cancer (see Table 5(Tab. 5) for the rest 11 pathways). Interestingly, only the hematopoietic cell lineage signaling pathway was enriched by the common DEGs, and the statistical test is close to significance (*p=0.085*), implying that the hematopoietic cell lineage signaling pathway “may be” an interactive linkage between CAD and AF that involves membrane metallo-endopeptidase (MME, also known as Neprilysin or Neutral endopeptidase 24.11) (Sankhe et al., 2020[41]), and TfR1.

### Protein-protein interaction network and molecular analysis

PPIs, either via strong or weak physical or functional interactions, play fundamental roles in cellular functions and biological processes of all organisms under normal condition and disease development (Rao et al., 2014[36]). In this respect, the present study used STRING to mine all proteins coded by the DEGs for potential interactions within and between the datasets from the patients with CAD and/or AF. 

**Figure 4:**
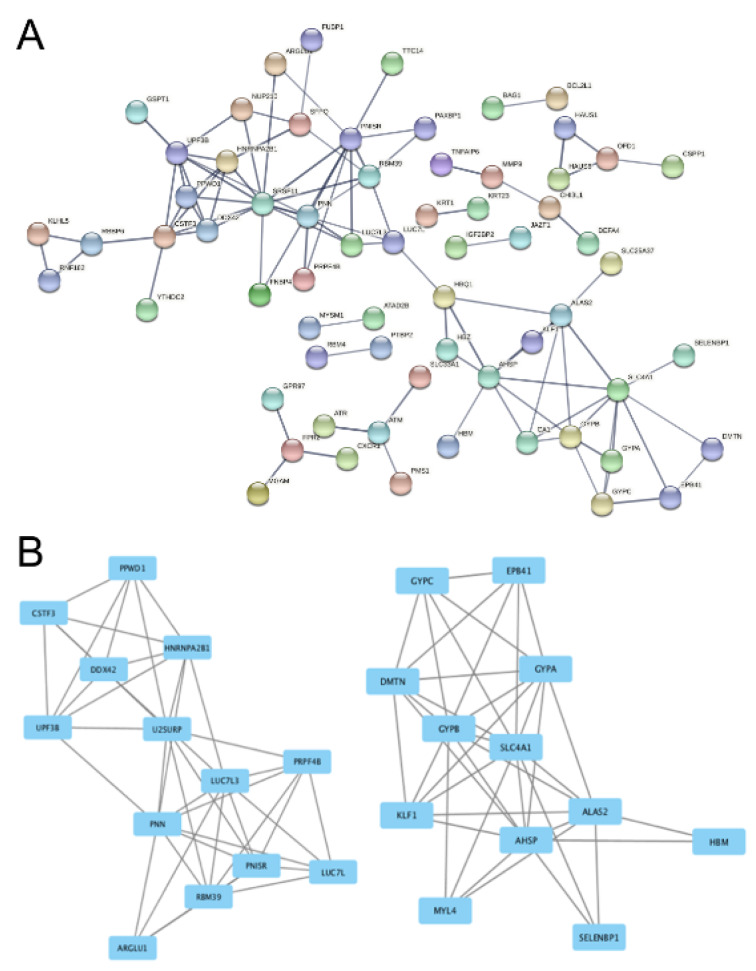
Protein-protein-interaction analysis of the DEGs in GSE71226 dataset. Panel A shows the overall network in GSE71226 dataset. Panel B shows the derived modules from the network. The rectangles stand for DEGs and the lines stand for their interactions. U2SURP, LUC7L and DDX42 in Module A (on the left side) and GYPC, EPB41 and ALAS2 in Module B (on the right side) are the most important nodes. See Supplementary Table 1 for all abbreviations.

Specifically, from GSE71226 dataset of the patients with CAD and healthy subjects, an intricate PPI network was recognized by STRING analysis. Since the network is so complex as shown in Figure 4A(Fig. 4), it is unlikely to decode network(s) of interest; thus, we screened 2 functional modules with the help of Cystoscape software in the network. The results showed that Module A (Figure 4B(Fig. 4), the left side) and Module B (Figure 4B(Fig. 4), the right side) contain 13 and 12 nodes, respectively. Among them, U2 snRNP-associated SURP motif-containing protein (U2SURP, a RNA binding protein (De Maio et al., 2018[7])), Luc7-like protein 3 (LUC7L3, a DNA/RNA binding protein (Tufarelli et al., 2001[43])), and Pinin (PNN, a DNA/RNA binding protein (Hsu et al., 2020[13])) are the most important nodes in Module A; while in Module B, it was found that Glycophorin-C (GYPC, an erythrocyte regulatory protein (Jaskiewicz et al., 2018[16])), Protein 4.1 (EBP41 or Beatty's protein, an erythrocyte structural and regulatory protein (Kiyomitsu and Cheeseman, 2013[19])), and Alpha-hemoglobin-stabilizing protein (ALAS2, a hemoglobin regulatory protein (Che Yaacob et al., 2020[5])) are the most important nodes.

**Figure 5:**
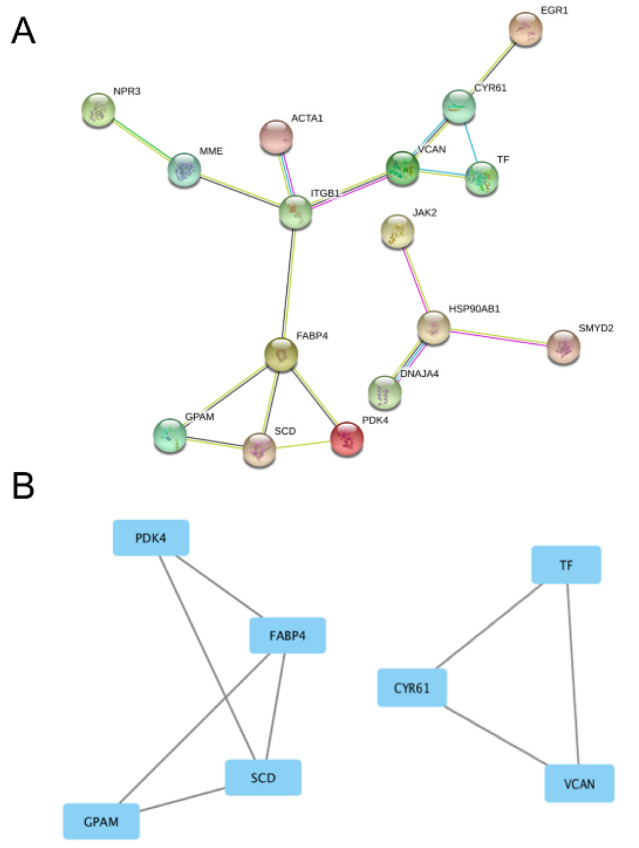
Protein-protein-interaction analysis of the DEGs in GSE31821 dataset. Panel A shows the overall network in GSE31821 dataset. Panel B shows the derived modules from the network. The rectangles stand for DEGs and the lines stand for their interactions. PDK4, FABP4, SCD and GPAM in Module A (on the left side) and CYR61, TF and VCAN in Module B (on the right side) are the most important nodes. See Supplementary Table 1 for all abbreviations.

On other hand, when the same approach was used in GSE31821 dataset of the patients with AF and healthy subjects, a relatively simple PPI network (containing 2 clusters with 9 nodes) was identified by STRING analysis (Figure 5(Fig. 5)). Further, Cystoscape analysis found that Module A (Figure 5B(Fig. 5), the left side) and Module B (Figure 5B(Fig. 5), the right side) have 4 and 3 nodes, respectively. Among these, Acyl-CoA desaturase (SCD, an enzyme involving biosynthesis of monounsaturated fatty acids (Vanhercke et al., 2011[44])), Fatty acid-binding protein (FABP4, a fatty acid transport protein (Rezar et al., 2020[37])), and Glycerol-3-phosphate acyltransferase 1 (GPAM, an enzyme involving glycerolipids biosynthesis (Mitka et al., 2019[29])) were the mostly critical nodes in Module A; while Serotransferrin (TF, an iron transport protein (Jamnongkan et al., 2019[15])), Protein CYR61 (CYR61, a cellular growth regulatory protein (Huang et al., 2017[14])), and Versican core protein (VCAN, an extracellular matrix proteoglycan (Gardela et al., 2020[10])) are the most critical nodes in Module B.

**Figure 6:**
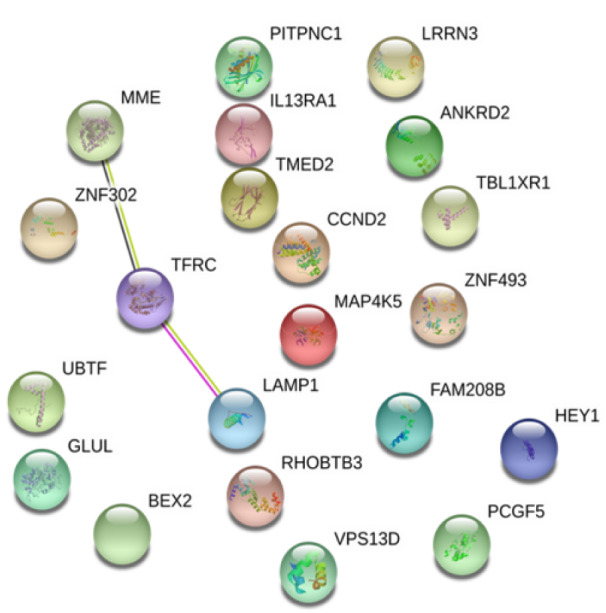
Protein-protein-interaction analysis of the 21 common DEGs in both datasets. Among 21 common DEGs, MME, TFRC and LAMP1 are the most significant nodes from the PPI network.

Interestingly, when the common DEGs from two datasets were analyzed using the same approach, it was found that only three proteins, MME, Transferrin receptor protein 1 (TfR1), and Lysosome-associated membrane glycoprotein 1 (LAMP1, an integral membrane protein with unknown function (Kirschner et al., 2016[18])), interacted each other while the remaining 18 proteins had no significant influencing characteristics (Figure 6(Fig. 6)). 

Taken together, these data suggested that although the PPI within and between two datasets are complex and most (if not all) functional interactions remain largely unknown, three proteins (MME, TfR1, LAMP1) may be concurrently involved in the development of CAD and AF.

## Discussion

In the present study, we investigated the common DEGs and molecular networks of two datasets consisting of the healthy subjects and the patients with CAD or AF using various bioinformatic tools. Overall, 565 up-regulated and 1367 down-regulated genes were discovered in the dataset from the patients with CAD, while 293 up-regulated and 68 down-regulated genes were revealed in the dataset from the patients with AF. From these genes, 21 common DEGs were highly enriched in the intracellular cytoplasm, protein binding, and labyrinthine layer of vessel in both CAD and AF patients. These common DEGs are involved in 4 pathways in the CAD dataset and 21 pathways in the AF dataset. Further analysis of those pathways identified three important proteins (MME, TfR1, LAMP1) that highly co-expressed in the CAD and AF patients. To the best of our knowledge, this is the first study to investigate the cross-correlation of all DEGs between the CAD and AF datasets. The findings may facilitate a better understanding of the mechanisms underlying the pathogenesis of CAD and AF.

The close relationship between CAD and AF has been well recognized in literature, including the fact such as patients with AF develop a high prevalence of CAD (Michniewicz et al., 2018[28]). It is known that genetic factors contribute importantly to both CAD and AF and studying the genetic basis of cardiovascular disease has made significant contribution to understand disease biology and promote cardiovascular therapy (Yla-Herttuala and Baker, 2017[48]). Numerous studies have identified a number of key genes and critical modules that are associated with CAD and AF by analyzing the microarrays data using bioinformatic tool and platform (Wang et al., 2016[47]; Zhang et al., 2014[51]). However, the genomic correlations between two diseases have not been fully investigated. 

Our studies found that there are significant number of up- and down-regulated genes in each disease, but these genes may not be directly correlated each other within two diseases. However, among those were 21 common DEGs identified from two datasets, including 14 up- and 7 down-regulated genes that could be involved in the pathogenesis of CAD and AF. At the protein levels, three major candidates of MME, TfR1, and LAMP1 that were encoded by the corresponding genes in those 21 common DEGs, were revealed by PPI network analysis, suggesting that these proteins may play a critical role in the development of two diseases. 

MME is a 100 kD type II transmembrane glycoprotein and plays an important role by enzymatically modulating the metabolism of glucagon, enkephalins, substance P, neurotensin, oxytocin, bradykinin, and atrial natriuretic peptides (ANP) (Roques, 1998[39]). Among these, ANP is a key peptide synthesized by the heart and contributes critical regulatory roles in normal cardiovascular homeostasis and cardiovascular disease (Munagala et al., 2004[31]). It was reported that MME is up-regulated in the heart of patients with heart failure and in the neutrophils of patients with early phase of acute myocardial infarction (Fielitz et al., 2002[9]; Knecht et al., 2002[20]). MME also controls local antifibrotic peptide bradykinin through the degradation of bradykinin in the extracellular space of heart tissue (Fielitz et al., 2002[9]). Our results indicated that MME is one of the common genes concurrently expressed in both CAD and AF, suggesting that MME could become a therapy target for the AF patients with CAD. 

TRFC gene encodes a cell surface receptor, termed transferring receptor 1 (TfR1), necessary for cellular iron uptake via the receptor-mediated endocytosis and it is essential for the function of red blood cell and development of the nervous system (Levy et al., 1999[24]). Both iron overload and iron deficiency, which are directly controlled by transferring receptor, were found to cause cardiomyopathy and heart failure (Anand and Gupta, 2018[3]; Kremastinos and Farmakis, 2011[21]). The present finding of highly expressed TfR1 in both CAD and AF provides additional evidence regarding the potential role of TfR1 in the pathogenesis of cardiovascular diseases. 

LAMP1 is a member of membrane glycoprotein family, and LAMP1/2 are major components of lysosomal membrane (Eskelinen, 2006[8]). Studies demonstrated LAMP1 is involved in autophagy process via mediating fusion between autophagosome and lysosomes; but the detailed mechanism is not fully understood. It was reported that the excessive autophagy by intracellular stress devoted significantly negative impacts on the developments of various cardiovascular diseases, including CAD and heart failure (Martinet et al., 2007[26]). Our study found a remarkable up-regulation of LAP1, supporting the possible involvement of LAMP1 in both CAD and AF. Surprisingly, mice with LAMP1 deficiency manifest normal lysosomal morphology and function (Andrejewski et al., 1999[4]). The discrepancy could be due to different species or experimental condition that need further investigation. 

The BP, MF, and CC are three terms commonly used in the GO enrichment analysis to reveal the involvement of genes of interest at different biological levels (Walter et al., 2015[46]). The present study found that the common DEGs for CAD and AF that were enriched mostly in the intracellular cytosol appear to be involved in the development of the labyrinthine layer during embryonic vessel development. Rinkenberger and Werb (2000[38]) demonstrated that the CC is involved in the labyrinthine layer of the placenta blood vessel progression and connected with cardiovascular system development; but future investigation is needed to address the biological role of the vascular labyrinthine layer in cardiovascular abnormalities, such as CAD and AF. 

Signaling pathway (or signaling cascade or biochemical cascade) is a series of cellular and molecular reactions that always take place in cells under normal and diseases conditions, including the development of CAD and AF. By running KEGG pathway analysis, Pocai (2019[35]) found that mRNA surveillance pathway, ribosome biogenesis, and glucagon signaling pathway were the major pathways that affect CAD. In the case of glucagon pathway, Ali et al. (2015[2]) demonstrated that glucagon administration impairs survival following ischemia in non-diabetic mouse and promote cardiomyocytes apoptosis. The present study identified 4 signaling pathways that were likely associated with CAD, supporting the above findings. By contrast, 21 pathways, such as Focal adhesion and MAPK pathways (see Table 5(Tab. 5)), were generated out of the AF dataset using the same approach. Among these, MAPK pathway is probably the most important signaling pathway related to the pathogenesis of cardiovascular disease, including AF (Zhang et al., 2003[50]). A study found that the MAPK pathway is involved in occurrence of AF in patients with rheumatic heart disease after cardiac surgery through promoting atrial fibrosis (Zhang et al., 2017[51]), which is consistent with our present finding on MAPK pathway.

In conclusion, the present study identified 21 common DEGs out of thousands of genes in the two datasets collected from the patients with CAD or AF. These common DEGs were highly enriched in the intracellular cytoplasm, protein binding, and vascular labyrinthine layer in patients. Three important protein candidates (MME, TfR1, and LAMP1) may play crucial roles in the disease development of both CAD and AF. We realized that the study comes with limitations. The subjects between the CAD and the AF have different ethnic background and medical history. The sample source and size should also be improved. Future studues using animal models with CAD and AF should be conducted to validate the hypothesis.

## Acknowledgements

None.

## Conflict of interest

The authors declare no conflict of interest.

## Funding

This work was supported by the NIH grant (1R15HL140528-01 for JQH), One-Health seed grant (PJ6SPVHJ for JQH) by the College of Veterinary Medicine at Virginia Tech and the Edward Via College of Osteopathic Medicine, Interdisciplinary Graduate Education Programs of Regenerative Medicine (IGEP-RM, for YJZ), and IRC Seed Grant (#178391 for JQH) by the College of Veterinary Medicine at Virginia Tech. The funders had no role in the study design, data collection and analysis, decision to publish, or preparation of the manuscript. 

## Supplementary material (online only)

The source datasets are available at 

https://www.ncbi.nlm.nih.gov/geo/.

Supplementary Table 1 - All abbreviations used in the this paper;

Supplementary Table 2 - All DEGs in GSE71226;

Supplementary Table 3 - All DEGs in GSE31821.

## Supplementary Material

Supplementary table 2

Supplementary table 3

Supplementary table 1

## Figures and Tables

**Table 1:**
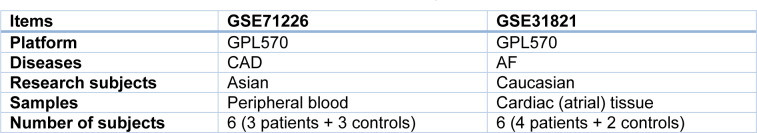
Basic information of datasets used in the study

**Table 2:**
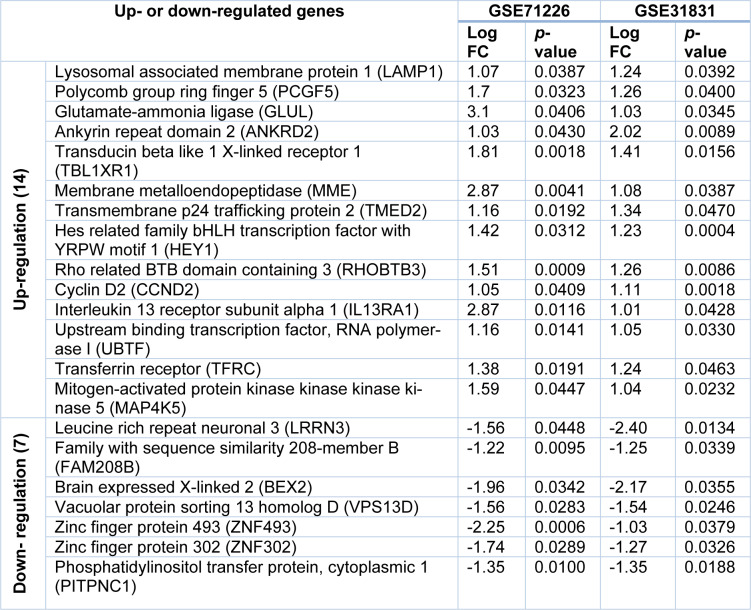
Twenty-one common differentially expressed genes (DEGs) identified in two datasets

**Table 3:**
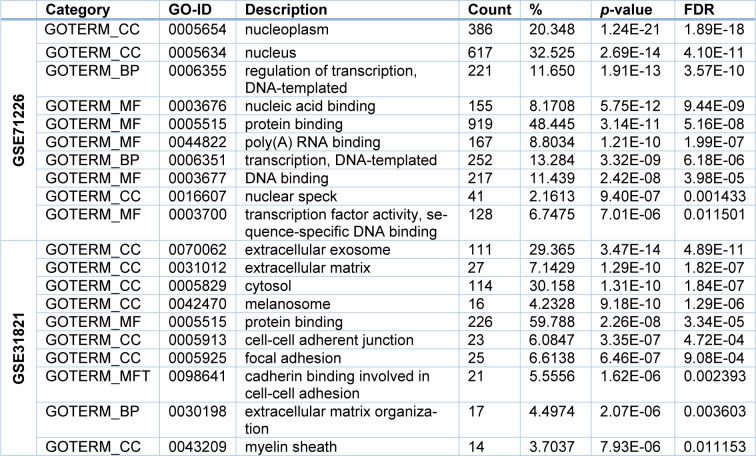
GO analysis of the top 10 genes in two datasets

**Table 4:**
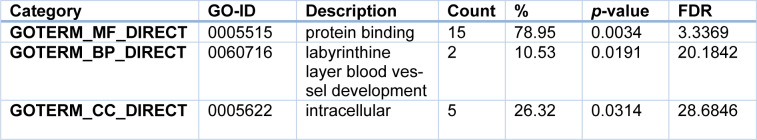
GO analysis of the 21 common DEGs

**Table 5:**
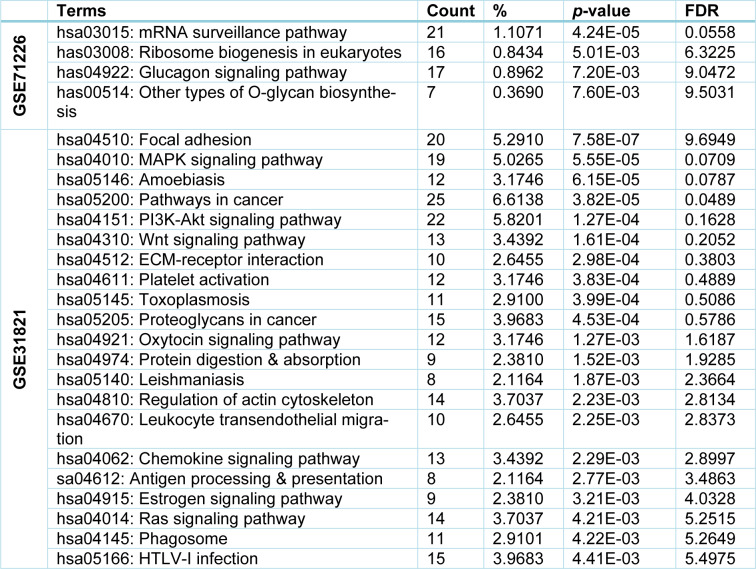
KEGG pathways identified in two datasets

## References

[1] Aibar S, Fontanillo C, Droste C, De Las Rivas J (2015). Functional gene networks: R/Bioc package to generate and analyse gene networks derived from functional enrichment and clustering. Bioinformatics.

[2] Ali S, Ussher JR, Baggio LL, Kabir MG, Charron MJ, Ilkayeva O (2015). Cardiomyocyte glucagon receptor signaling modulates outcomes in mice with experimental myocardial infarction. Mol Metab.

[3] Anand IS, Gupta P (2018). Anemia and iron deficiency in heart failure: Current concepts and emerging therapies. Circulation.

[4] Andrejewski N, Punnonen EL, Guhde G, Tanaka Y, Lüllmann-Rauch R, Hartmann D (1999). Normal lysosomal morphology and function in LAMP-1-deficient mice. J Biol Chem.

[5] Che Yaacob NS, Islam MA, Alsaleh H, Ibrahim IK, Hassan R (2020). Alpha-hemoglobin-stabilizing protein (AHSP): A modulatory factor in beta-thalassemia. Int J Hematol.

[6] Davis S, Meltzer PS (2007). GEOquery: a bridge between the Gene Expression Omnibus (GEO) and BioConductor. Bioinformatics.

[7] De Maio A, Yalamanchili HK, Adamski CJ, Gennarino VA, Liu Z, Qin J (2018). RBM17 Interacts with U2SURP and CHERP to regulate expression and splicing of RNA-processing proteins. Cell Rep.

[8] Eskelinen EL (2006). Roles of LAMP-1 and LAMP-2 in lysosome biogenesis and autophagy. Mol Aspects Med.

[9] Fielitz J, Dendorfer A, Pregla R, Ehler E, Zurbrügg H, Bartunek J (2002). Neutral endopeptidase is activated in cardiomyocytes in human aortic valve stenosis and heart failure. Circulation.

[10] Gardela J, Jauregi-Miguel A, Martinez CA, Rodriguez-Martinez H, Lopez-Bejar M, Alvarez-Rodriguez M (2020). Semen modulates the expression of NGF, ABHD2, VCAN, and CTEN in the reproductive tract of female rabbits. Genes (Basel).

[11] Gladding PA, Legget M, Fatkin D, Larsen P, Doughty R (2020). Polygenic risk scores in coronary artery disease and atrial fibrillation. Heart Lung Circ.

[12] Go AS, Hylek EM, Phillips KA, Chang Y, Henault LE, Selby JV (2001). Prevalence of diagnosed atrial fibrillation in adults: national implications for rhythm management and stroke prevention: The AnTicoagulation and Risk Factors in Atrial Fibrillation (ATRIA) Study. JAMA.

[13] Hsu SY, Mukda S, Leu S (2020). Expression and distribution pattern of pnn in ischemic cerebral cortex and cultured neural cells exposed to oxygen-glucose deprivation. Brain Sci.

[14] Huang YT, Lan Q, Lorusso G, Duffey N, Ruegg C (2017). The matricellular protein CYR61 promotes breast cancer lung metastasis by facilitating tumor cell extravasation and suppressing anoikis. Oncotarget.

[15] Jamnongkan W, Lebrilla CB, Barboza M, Techasen A, Loilome W, Sithithaworn P (2019). Discovery of serotransferrin glycoforms: Novel markers for diagnosis of liver periductal fibrosis and prediction of cholangiocarcinoma. Biomolecules.

[16] Jaskiewicz E, Peyrard T, Kaczmarek R, Zerka A, Jodlowska M, Czerwinski M (2018). The Gerbich blood group system: Old knowledge, new importance. Transfus Med Rev.

[17] Kertai MD, Li YJ, Ji Y, Qi W, Lombard FW, Shah SH (2015). Genome-wide association study of new-onset atrial fibrillation after coronary artery bypass grafting surgery. Am Heart J.

[18] Kirschner A, Thiede M, Blaeschke F, Richter GH, Gerke JS, Baldauf MC (2016). Lysosome-associated membrane glycoprotein 1 predicts fratricide amongst T cell receptor transgenic CD8+ T cells directed against tumor-associated antigens. Oncotarget.

[19] Kiyomitsu T, Cheeseman IM (2013). Cortical dynein and asymmetric membrane elongation coordinately position the spindle in anaphase. Cell.

[20] Knecht M, Pagel I, Langenickel T, Philipp S, Scheuermann-Freestone M, Willnow T (2002). Increased expression of renal neutral endopeptidase in severe heart failure. Life Sci.

[21] Kremastinos DT, Farmakis D (2011). Iron overload cardiomyopathy in clinical practice. Circulation.

[22] Kristensen KE, Knage CC, Nyhegn LH, Mulder BA, Rienstra M, Van Gelder IC (2020). Subclinical atherosclerosis is associated with incident atrial fibrillation: a systematic review and meta-analysis. Europace.

[23] Kumar D, Dash D (2016). Proteogenomic tools and approaches to explore protein coding landscapes of eukaryotic genomes. Adv Exp Med Biol.

[24] Levy JE, Jin O, Fujiwara Y, Kuo F, Andrews NC (1999). Transferrin receptor is necessary for development of erythrocytes and the nervous system. Nat Genet.

[25] Lieder H, Breithardt G, Heusch G (2018). Fatal attraction - A brief pathophysiology of the interaction between atrial fibrillation and myocardial ischemia. Int J Cardiol.

[26] Martinet W, Knaapen MW, Kockx MM, De Meyer GR (2007). Autophagy in cardiovascular disease. Trends Mol Med.

[27] Mehta RH, Dabbous OH, Granger CB, Kuznetsova P, Kline-Rogers EM, Anderson FA (2003). Comparison of outcomes of patients with acute coronary syndromes with and without atrial fibrillation. Am J Cardiol.

[28] Michniewicz E, Mlodawska E, Lopatowska P, Tomaszuk-Kazberuk A, Malyszko J (2018). Patients with atrial fibrillation and coronary artery disease - Double trouble. Adv Med Sci.

[29] Mitka I, Ropka-Molik K, Tyra M (2019). Functional analysis of genes involved in glycerolipids biosynthesis (GPAT1 and GPAT2) in pigs. Animals (Basel).

[30] Motloch LJ, Reda S, Larbig R, Wolff A, Motloch KA, Wernly B (2017). Characteristics of coronary artery disease among patients with atrial fibrillation compared to patients with sinus rhythm. Hellenic J Cardiol.

[31] Munagala VK, Burnett JC, Redfield MM (2004). The natriuretic peptides in cardiovascular medicine. Curr Probl Cardiol.

[32] Murakami N, Tanno M, Kokubu N, Nishida J, Nagano N, Ohnishi H (2017). Distinct risk factors of atrial fibrillation in patients with and without coronary artery disease: a cross-sectional analysis of the BOREAS-CAG Registry data. Open Heart.

[33] Ogata H, Goto S, Sato K, Fujibuchi W, Bono H, Kanehisa M (1999). KEGG: Kyoto encyclopedia of genes and genomes. Nucleic Acids Res.

[34] Pilgrim T, Kalesan B, Zanchin T, Pulver C, Jung S, Mattle H (2013). Impact of atrial fibrillation on clinical outcomes among patients with coronary artery disease undergoing revascularisation with drug-eluting stents. EuroIntervention.

[35] Pocai A (2019). Modulation of glucagon signaling: A metabolic approach for heart failure?. JACC Basic Transl Sci.

[36] Rao VS, Srinivas K, Sujini GN, Kumar GN (2014). Protein-protein interaction detection: Methods and analysis. Int J Proteomics.

[37] Rezar R, Jirak P, Gschwandtner M, Derler R, Felder TK, Haslinger M (2020). Heart-type fatty acid-binding protein (H-FABP) and its role as a biomarker in heart failure: What do we know so far?. J Clin Med.

[38] Rinkenberger J, Werb Z (2000). The labyrinthine placenta. Nat Genet.

[39] Roques BP (1998). Cell surface metallopeptidases involved in blood pressure regulation: Structure, inhibition and clinical perspectives. Pathol Biol (Paris).

[40] Ruddox V, Sandven I, Munkhaugen J, Skattebu J, Edvardsen T, Otterstad JE (2017). Atrial fibrillation and the risk for myocardial infarction, all-cause mortality and heart failure: A systematic review and meta-analysis. Eur J Prev Cardiol.

[41] Sankhe R, Pai SRK, Kishore A (2020). Tumour suppression through modulation of neprilysin signaling: A comprehensive review. Eur J Pharmacol.

[42] Szklarczyk D, Franceschini A, Wyder S, Forslund K, Heller D, Huerta-Cepas J (2015). STRING v10: protein-protein interaction networks, integrated over the tree of life. Nucleic Acids Res.

[43] Tufarelli C, Frischauf AM, Hardison R, Flint J, Higgs DR (2001). Characterization of a widely expressed gene (LUC7-LIKE;LUC7L) defining the centromeric boundary of the human alpha-globin domain. Genomics.

[44] Vanhercke T, Shrestha P, Green AG, Singh SP (2011). Mechanistic and structural insights into the regioselectivity of an acyl-CoA fatty acid desaturase via directed molecular evolution. J Biol Chem.

[45] Virani SS, Alonso A, Benjamin EJ, Bittencourt MS, Callaway CW, Carson AP (2020). Heart disease and stroke statistics-2020 update: A report from the American Heart Association. Circulation.

[46] Walter W, Sanchez-Cabo F, Ricote M (2015). GOplot: An R package for visually combining expression data with functional analysis. Bioinformatics.

[47] Wang T, Wang B (2016). Identification of microRNA-mRNA interactions in atrial fibrillation using microarray expression profiles and bioinformatics analysis. Mol Med Rep.

[48] Yla-Herttuala S, Baker AH (2017). Cardiovascular gene therapy: Past, present, and future. Mol Ther.

[49] Zhang D, Chen X, Wang Q, Wu S, Zheng Y, Liu X (2017). Role of the MAPKs/TGF-beta1/TRAF6 signaling pathway in postoperative atrial fibrillation. PLoS One.

[50] Zhang W, Elimban V, Nijjar MS, Gupta SK, Dhalla NS (2003). Role of mitogen-activated protein kinase in cardiac hypertrophy and heart failure. Exp Clin Cardiol.

[51] Zhang X, Cheng X, Liu H, Zheng C, Rao K, Fang Y (2014). Identification of key genes and crucial modules associated with coronary artery disease by bioinformatics analysis. Int J Mol Med.

[52] Zheng Q, Wang XJ (2008). GOEAST: A web-based software toolkit for Gene Ontology enrichment analysis. Nucleic Acids Res.

