# Supplementary material for: Common differentially expressed genes and pathways correlating both coronary artery disease and atrial fibrillation
Source: EXCLI J. 2021 Jan 18;20:126–41. doi: 10.17179/excli2020-3262 (PMC7868642; doi:10.17179/excli2020-3262)
Supplement: Supplementary table 2 [file EXCLI-20-126-s-001.pdf]

EXCLI Journal 2021;20:126-141– ISSN 1611-2156
Received: December 08, 2020, accepted: January 11, 2021, published: January 18, 2021

Supplementary material to:

Original article:

COMMON DIFFERENTIALLY EXPRESSED GENES AND PATHWAYS CORRELATING BOTH CORONARY ARTERY DISEASE AND ATRIAL FIBRILLATION

Youjing Zheng, Jia-Qiang He*

Department of Biomedical Sciences and Pathobiology, College of Veterinary Medicine, Virginia Tech, Blacksburg, VA 24061, USA

* **Corresponding author:** Jia-Qiang He, Department of Biomedical Sciences and Pathobiology, Virginia Tech, Phase II, Room 252B, Blacksburg, VA 24061, USA. Tel: 1-540-231-2032. E-mail: jiahe@vt.edu

https://orcid.org/0000-0002-4825-7046 Youjing Zheng
https://orcid.org/0000-0002-0640-5960 Jia-Qiang He

http://dx.doi.org/10.17179/excli2020-3262

This is an Open Access article distributed under the terms of the Creative Commons Attribution License (http://creativecommons.org/licenses/by/4.0/).

Supplementary Table 2: DEGs in GSE71226 dataset. Up- and down-regulated genes are on page S1-S18 and page S18-S64, respectively.

**565 up-regulated genes**				
**ID**	***p* value**	**logFC**	**Gene symbol**	**Gene title**
**240336_at**	0.02848	5.33	HBM	Hemoglobin subunit mu
**204848_x_at**	0.037185	4.49	HBG2///HBG1	Hemoglobin subunit gamma 2///hemoglobin subunit gamma 1
**211560_s_at**	0.019702	4.18	ALAS2	5'-aminolevulinate synthase 2
**231078_at**	0.026593	4.06	SLC25A37	Solute carrier family 25 member 37
**214433_s_at**	0.012574	3.99	SELENBP1	Selenium binding protein 1
**233371_at**	0.005686	3.89	ABCC13	ATP binding cassette subfamily C member 13 (pseudogene)
**211475_s_at**	0.048215	3.86	BAG1	BCL2 associated athanogene 1
**235683_at**	0.045627	3.86	SESN3	Sestrin 3
**202219_at**	0.025228	3.76	SLC6A8	Solute carrier family 6 member 8
**1553589_a_at**	0.039121	3.75	PDZK1IP1	PDZK1 interacting protein 1
**219630_at**	0.026088	3.71	PDZK1IP1	PDZK1 interacting protein 1
**213515_x_at**	0.023848	3.68	HBG2///HBG1	Hemoglobin subunit gamma 2///hemoglobin subunit gamma 1
**241881_at**	0.019701	3.61	TRIM58	Tripartite motif containing 58

EXCLI Journal 2021;20:126-141– ISSN 1611-2156
Received: December 08, 2020, accepted: January 11, 2021, published: January 18, 2021

**219672_at**	0.037109	3.55	AHSP	Alpha hemoglobin stabilizing protein
**205592_at**	0.028653	3.48	SLC4A1	Solute carrier family 4 member 1 (Diego blood group)
**205950_s_at**	0.021762	3.39	CA1	Carbonic anhydrase 1
**1552713_a_at**	0.012073	3.37	SLC4A1	Solute carrier family 4 member 1 (Diego blood group)
**230720_at**	0.005023	3.29	RNF182	Ring finger protein 182
**1555167_s_at**	0.011506	3.26	NAMPT	Nicotinamide phosphoribosyltransferase
**225800_at**	0.030469	3.22	JAZF1	JAZF zinc finger 1
**206026_s_at**	0.026852	3.19	TNFAIP6	TNF alpha induced protein 6
**223266_at**	0.041247	3.19	STRADB	STE20-related kinase adaptor beta
**209396_s_at**	0.020758	3.1	CHI3L1	Chitinase 3 like 1
**220807_at**	0.035619	3.1	HBQ1	Hemoglobin subunit theta 1
**217202_s_at**	0.040557	3.1	GLUL	Glutamate-ammonia ligase
**206515_at**	0.016854	3.09	CYP4F3	Cytochrome P450 family 4 subfamily F member 3
**211372_s_at**	0.002982	3.02	IL1R2	Interleukin 1 receptor type 2
**210504_at**	0.027167	2.94	KLF1	Kruppel like factor 1
**202947_s_at**	0.044811	2.94	GYPC	Glycophorin C (Gerbich blood group)
**205837_s_at**	0.00186	2.91	GYPA	Glycophorin A (MNS blood group)
**1552583_s_at**	0.008415	2.91	ABCC13	ATP binding cassette subfamily C member 13 (pseudogene)
**209395_at**	0.041373	2.91	CHI3L1	Chitinase 3 like 1
**228634_s_at**	0.00345	2.88	YBX3	Y-box binding protein 3
**203434_s_at**	0.004089	2.87	MME	Membrane metallo-endopeptidase
**210904_s_at**	0.011555	2.87	IL13RA1	Interleukin 13 receptor subunit alpha 1
**224693_at**	0.008975	2.83	FAM210B	Family with sequence similarity 210 member B
**206522_at**	0.036426	2.82	MGAM	Maltase-glucoamylase
**211821_x_at**	0.004341	2.78	GYPA	Glycophorin A (MNS blood group)
**218864_at**	0.004351	2.78	TNS1	Tensin 1
**214407_x_at**	0.008268	2.76	GYPB	Glycophorin B (MNS blood group)
**220945_x_at**	0.023818	2.76	MANSC1	MANSC domain containing 1
**1558688_at**	0.008146	2.7	STX17-AS1	STX17 antisense RNA 1
**227935_s_at**	0.032299	2.7	PCGF5	Polycomb group ring finger 5
**218963_s_at**	0.013502	2.64	KRT23	Keratin 23
**215242_at**	0.009474	2.63	PIGC	Phosphatidylinositol glycan anchor biosynthesis class C
**211820_x_at**	0.013152	2.61	GYPA	Glycophorin A (MNS blood group)
**210395_x_at**	0.047611	2.6	MYL4	Myosin light chain 4
**213843_x_at**	0.013075	2.52	SLC6A8	Solute carrier family 6 member 8
**220757_s_at**	0.033641	2.47	MIR4746///UBXN6	Microrna 4746///UBX domain protein 6

EXCLI Journal 2021;20:126-141– ISSN 1611-2156
Received: December 08, 2020, accepted: January 11, 2021, published: January 18, 2021

**203435_s_at**	0.029537	2.46	MME	Membrane metallo-endopeptidase
**203691_at**	0.032145	2.46	PI3	Peptidase inhibitor 3
**206222_at**	0.000362	2.45	TNFRSF10C	TNF receptor superfamily member 10c
**204505_s_at**	0.003366	2.44	DMTN	Dematin actin binding protein
**205568_at**	0.037595	2.42	AQP9	Aquaporin 9
**215037_s_at**	0.01634	2.38	BCL2L1	BCL2 like 1
**214464_at**	0.021483	2.37	CDC42BPA	CDC42 binding protein kinase alpha
**206765_at**	0.01426	2.34	KCNJ2	Potassium voltage-gated channel subfamily J member 2
**210655_s_at**	0.033242	2.32	FOXO3B///FOXO3	Forkhead box O3B pseudogene///forkhead box O3
**41469_at**	0.026199	2.31	PI3	Peptidase inhibitor 3
**207827_x_at**	0.043831	2.3	SNCA	Synuclein alpha
**218644_at**	0.005216	2.29	PLEK2	Pleckstrin 2
**225387_at**	0.042324	2.29	TSPAN5	Tetraspanin 5
**205900_at**	0.002615	2.28	KRT1	Keratin 1
**206207_at**	0.03661	2.28	CLC	Charcot-Leyden crystal galectin
**206647_at**	0.031235	2.28	HBZ	Hemoglobin subunit zeta
**203936_s_at**	0.018717	2.27	MMP9	Matrix metallopeptidase 9
**210773_s_at**	0.014872	2.26	FPR2	Formyl peptide receptor 2
**210772_at**	0.018787	2.26	FPR2	Formyl peptide receptor 2
**1554481_a_at**	0.009745	2.23	EPB41	Erythrocyte membrane protein band 4.1
**213608_s_at**	0.038543	2.22	SRRD	SRR1 domain containing
**1552348_at**	0.030526	2.2	PRSS33	Protease, serine 33
**206196_s_at**	0.024554	2.19	RUNDC3A	RUN domain containing 3A
**218847_at**	0.027815	2.19	IGF2BP2	Insulin like growth factor 2 mrna binding protein 2
**226928_x_at**	0.026105	2.17	SLC25A37	Solute carrier family 25 member 37
**242267_x_at**	0.044868	2.16	LOC105369595	Uncharacterized LOC105369595
**201365_at**	0.002454	2.15	OAZ2	Ornithine decarboxylase antizyme 2
**207459_x_at**	0.041186	2.15	GYPB	Glycophorin B (MNS blood group)
**202101_s_at**	0.004618	2.14	RALB	RALB Ras like proto-oncogene B
**207094_at**	0.004702	2.14	CXCR1	C-X-C motif chemokine receptor 1
**207269_at**	0.044265	2.14	DEFA4	Defensin alpha 4
**231933_at**	0.033743	2.13	8-Mar	Membrane associated ring-CH-type finger 8
**201333_s_at**	0.005993	2.09	ARHGEF12	Rho guanine nucleotide exchange factor 12
**221551_x_at**	0.040961	2.09	ST6GAL-NAC4	ST6 N-acetylgalactosaminide alpha-2,6-sialyltransferase 4
**210854_x_at**	0.028678	2.07	SLC6A8	Solute carrier family 6 member 8
**226489_at**	0.002389	2.06	TMCC3	Transmembrane and coiled-coil domain family 3

EXCLI Journal 2021;20:126-141– ISSN 1611-2156
Received: December 08, 2020, accepted: January 11, 2021, published: January 18, 2021

**216833_x_at**	0.008884	2.04	GYPB	Glycophorin B (MNS blood group)
**210484_s_at**	0.048154	2.04	LOC254896///TNFRSF10C	Uncharacterized LOC254896///TNF receptor superfamily member 10c
**220404_at**	0.013028	2.02	ADGRG3	Adhesion G protein-coupled receptor G3
**205896_at**	0.016258	2.02	SLC22A4	Solute carrier family 22 member 4
**239206_at**	0.046343	2.02	CR1L	Complement component 3b/4b receptor 1-like
**213319_s_at**	0.001569	1.99	YBX3	Y-box binding protein 3
**238245_at**	0.011847	1.99	ENPP7	Ectonucleotide pyrophosphatase/phosphodiesterase 7
**205389_s_at**	0.006431	1.98	ANK1	Ankyrin 1
**206632_s_at**	0.021246	1.98	APOBEC3B	Apolipoprotein B mrna editing enzyme catalytic subunit 3B
**241620_at**	0.023945	1.98	SMCHD1	Structural maintenance of chromosomes flexible hinge domain containing 1
**227366_at**	0.002293	1.97	RILP	Rab interacting lysosomal protein
**223549_s_at**	0.009283	1.94	ESPN	Espin
**202448_s_at**	0.002851	1.91	ZER1	Zyg-11 related cell cycle regulator
**209697_at**	0.01377	1.91	PPP3CC	Protein phosphatase 3 catalytic subunit gamma
**222678_s_at**	0.014571	1.88	DCUN1D1	Defective in cullin neddylation 1 domain containing 1
**224327_s_at**	0.001678	1.87	DGAT2	Diacylglycerol O-acyltransferase 2
**239853_at**	0.005656	1.87	KLC3	Kinesin light chain 3
**203289_s_at**	0.004674	1.86	NPRL3	NPR3 like, GATOR1 complex subunit
**207890_s_at**	0.014227	1.85	MMP25	Matrix metallopeptidase 25
**55705_at**	0.048261	1.85	R3HDM4	R3H domain containing 4
**207384_at**	0.005895	1.84	PGLYRP1	Peptidoglycan recognition protein 1
**222804_x_at**	0.007153	1.84	DCAF10	DDB1 and CUL4 associated factor 10
**201291_s_at**	0.039252	1.82	TOP2A	Topoisomerase (DNA) II alpha
**222634_s_at**	0.001779	1.81	TBL1XR1	Transducin (beta)-like 1 X-linked receptor 1
**209179_s_at**	0.014463	1.8	MBOAT7	Membrane bound O-acyltransferase domain containing 7
**223285_s_at**	0.033372	1.8	ST6GALNAC4	ST6 N-acetylgalactosaminide alpha-2,6-sialyltransferase 4
**226248_s_at**	0.024043	1.8	KIAA1324	Kiaa1324
**1569207_s_at**	0.016352	1.79	TCP11L2	T-complex 11 like 2
**206470_at**	0.017151	1.79	PLXNC1	Plexin C1
**220832_at**	0.024289	1.78	TLR8	Toll like receptor 8
**226064_s_at**	0.044995	1.78	DGAT2	Diacylglycerol O-acyltransferase 2
**230585_at**	0.030493	1.78	KCNJ15	Potassium voltage-gated channel subfamily J member 15
**217889_s_at**	0.038876	1.77	CYBRD1	Cytochrome b reductase 1

EXCLI Journal 2021;20:126-141– ISSN 1611-2156
Received: December 08, 2020, accepted: January 11, 2021, published: January 18, 2021

**214446_at**	0.035169	1.76	ELL2	Elongation factor for RNA polymerase II 2
**221237_s_at**	0.009721	1.76	OSBP2	Oxysterol binding protein 2
**231079_at**	0.000382	1.76	NANOG	Nanog homeobox
**40850_at**	0.012943	1.76	FKBP8	FK506 binding protein 8
**204748_at**	0.037497	1.75	PTGS2	Prostaglandin-endoperoxide synthase 2
**221060_s_at**	0.024561	1.75	TLR4	Toll like receptor 4
**222730_s_at**	0.021457	1.74	ZDHHC2	Zinc finger DHHC-type containing 2
**214321_at**	0.01642	1.7	NOV	Nephroblastoma overexpressed
**223432_at**	0.026791	1.7	OSBP2	Oxysterol binding protein 2
**206145_at**	0.019437	1.69	RHAG	Rh-associated glycoprotein
**225167_at**	0.028709	1.69	FRMD4A	FERM domain containing 4A
**231982_at**	0.024947	1.69	SMIM24	Small integral membrane protein 24
**235845_at**	0.001485	1.69	SP5	Sp5 transcription factor
**204750_s_at**	0.01759	1.66	DSC2	Desmocollin 2
**225271_at**	0.003811	1.66	TMEM63B	Transmembrane protein 63B
**205041_s_at**	0.042785	1.65	ORM2///ORM 1	Orosomucoid 2///orosomucoid 1
**220173_at**	0.042656	1.65	BBOF1	Basal body orientation factor 1
**216671_x_at**	0.013121	1.63	MUC8	Mucin 8
**227757_at**	0.02507	1.63	CUL4A	Cullin 4A
**219227_at**	0.007867	1.62	CCNJL	Cyclin J like
**221220_s_at**	0.010718	1.62	SCYL2	SCY1 like pseudokinase 2
**208459_s_at**	0.011139	1.59	XPO7	Exportin 7
**211081_s_at**	0.044719	1.59	MAP4K5	Mitogen-activated protein kinase kinase kinase kinase 5
**215886_x_at**	0.044298	1.59	USP12	Ubiquitin specific peptidase 12
**218825_at**	0.004564	1.59	EGFL7	EGF like domain multiple 7
**228361_at**	0.02258	1.58	E2F2	E2F transcription factor 2
**214428_x_at**	0.005956	1.56	C4B_2///C4B///C4A	Complement component 4B (Chido blood group), copy 2///complement component 4B (Chido blood group)///complement component 4A (Rodgers blood group)
**203030_s_at**	0.04273	1.55	PTPRN2	Protein tyrosine phosphatase, receptor type N2
**214510_at**	0.000655	1.55	GPR20	G protein-coupled receptor 20
**217040_x_at**	0.013203	1.55	SOX15	SRY-box 15
**1560527_at**	0.009088	1.54	NFE4	Nuclear factor, erythroid 4
**1565537_at**	0.024677	1.54	NKX1-1	NK1 homeobox 1
**226726_at**	0.018965	1.54	MBOAT2	Membrane bound O-acyltransferase domain containing 2
**1570402_at**	0.002468	1.52	KLC3	Kinesin light chain 3
**221747_at**	0.001208	1.52	TNS1	Tensin 1
**1557455_s_at**	0.034515	1.51	MOSPD1	Motile sperm domain containing 1
**1569105_at**	0.024071	1.51	SETD5	SET domain containing 5

EXCLI Journal 2021;20:126-141– ISSN 1611-2156
Received: December 08, 2020, accepted: January 11, 2021, published: January 18, 2021

**216048_s_at**	0.00092	1.51	RHOBTB3	Rho related BTB domain containing 3
**216549_s_at**	0.041387	1.5	TBC1D22B	TBC1 domain family member 22B
**223094_s_at**	0.029507	1.5	ANKH	ANKH inorganic pyrophosphate transport regulator
**208353_x_at**	0.015184	1.49	ANK1	Ankyrin 1
**210087_s_at**	0.013826	1.49	MPZL1	Myelin protein zero like 1
**221497_x_at**	0.008542	1.49	EGLN1	Egl-9 family hypoxia inducible factor 1
**227781_x_at**	0.010268	1.49	FAM57B	Family with sequence similarity 57 member B
**241013_at**	0.001852	1.49	FAM124A	Family with sequence similarity 124 member A
**203887_s_at**	0.031081	1.48	THBD	Thrombomodulin
**205020_s_at**	0.04462	1.48	ARL4A	ADP ribosylation factor like gtpase 4A
**207042_at**	0.032009	1.47	E2F2	E2F transcription factor 2
**230458_at**	0.00872	1.47	SLC45A1	Solute carrier family 45 member 1
**1553976_a_at**	0.036395	1.45	DPCD	Deleted in primary ciliary dyskinesia homolog (mouse)
**202411_at**	0.020843	1.45	IFI27	Interferon alpha inducible protein 27
**228412_at**	0.006902	1.44	LOC643072	Uncharacterized LOC643072
**238074_at**	0.001479	1.44	WDR27	WD repeat domain 27
**238428_at**	0.040153	1.44	KCNJ15	Potassium voltage-gated channel subfamily J member 15
**37408_at**	0.00361	1.44	MRC2	Mannose receptor C type 2
**201044_x_at**	0.040012	1.43	DUSP1	Dual specificity phosphatase 1
**218030_at**	0.017588	1.43	GIT1	GIT arfgap 1
**240029_at**	0.010328	1.43	C11orf94	Chromosome 11 open reading frame 94
**241433_at**	0.022784	1.43	RCOR3	REST corepressor 3
**1569206_at**	0.033737	1.42	TCP11L2	T-complex 11 like 2
**205919_at**	0.037469	1.42	HBE1	Hemoglobin subunit epsilon 1
**218839_at**	0.03118	1.42	HEY1	Hes related family bhlh transcription factor with YRPW motif 1
**229996_s_at**	0.032954	1.42	PCGF5	Polycomb group ring finger 5
**230299_s_at**	0.003193	1.42	WNT5B	Wnt family member 5B
**213478_at**	0.015479	1.41	KAZN	Kazrin, periplakin interacting protein
**221627_at**	0.049742	1.41	TRIM10	Tripartite motif containing 10
**206531_at**	0.009525	1.4	DPF1	Double PHD fingers 1
**206573_at**	0.007338	1.4	KCNQ3	Potassium voltage-gated channel subfamily Q member 3
**207321_s_at**	0.04452	1.4	ABCB9	ATP binding cassette subfamily B member 9
**209661_at**	0.030866	1.4	KIFC3	Kinesin family member C3
**214103_s_at**	0.000992	1.4	RAP2A	RAP2A, member of RAS oncogene family

EXCLI Journal 2021;20:126-141– ISSN 1611-2156
Received: December 08, 2020, accepted: January 11, 2021, published: January 18, 2021

**222871_at**	0.029309	1.4	KLHDC8A	Kelch domain containing 8A
**237818_at**	0.009224	1.4	LARP6	La ribonucleoprotein domain family member 6
**205021_s_at**	0.022889	1.39	FOXN3	Forkhead box N3
**206123_at**	0.005129	1.39	LLGL1	LLGL1, scribble cell polarity complex component
**221163_s_at**	0.00911	1.39	MLXIPL	MLX interacting protein like
**221672_s_at**	0.007189	1.39	TRAPPC9	Trafficking protein particle complex 9
**226442_at**	0.009217	1.39	ABTB1	Ankyrin repeat and BTB domain containing 1
**236218_at**	0.030064	1.39	PHOSPHO1	Phosphoethanolamine/phosphocholine phosphatase
**215668_s_at**	0.026815	1.38	PLXNB1	Plexin B1
**228052_x_at**	0.003679	1.38	TCF3	Transcription factor 3
**229032_at**	0.019244	1.38	WSCD2	WSC domain containing 2
**240686_x_at**	0.001913	1.38	TFRC	Transferrin receptor
**244882_at**	0.010764	1.38	TNRC18	Trinucleotide repeat containing 18
**209498_at**	0.022823	1.37	CEACAM1	Carcinoembryonic antigen related cell adhesion molecule 1
**217683_at**	0.035831	1.37	HBE1	Hemoglobin subunit epsilon 1
**1560767_at**	0.041478	1.36	HCG22	HLA complex group 22
**205654_at**	0.03067	1.36	C4BPA	Complement component 4 binding protein alpha
**206911_at**	0.012753	1.36	TRIM25	Tripartite motif containing 25
**209589_s_at**	0.006436	1.36	EPHB2	EPH receptor B2
**209850_s_at**	0.020094	1.36	CDC42EP2	CDC42 effector protein 2
**213740_s_at**	0.002174	1.36	TMEM262	Transmembrane protein 262
**228350_at**	0.04328	1.36	UNC13D	Unc-13 homolog D
**206440_at**	0.003312	1.35	LIN7A	Lin-7 homolog A, crumbs cell polarity complex component
**216332_at**	0.005745	1.35	POU6F1	POU class 6 homeobox 1
**223952_x_at**	0.026552	1.35	DHRS9	Dehydrogenase/reductase 9
**204371_s_at**	0.020682	1.34	KHSRP	KH-type splicing regulatory protein
**216485_s_at**	0.001369	1.34	TPSAB1	Tryptase alpha/beta 1
**220427_at**	0.02496	1.34	OBSCN	Obscurin, cytoskeletal calmodulin and titin-interacting rhogef
**237252_at**	0.003908	1.34	THBD	Thrombomodulin
**238916_at**	0.017848	1.34	LINC00938	Long intergenic non-protein coding RNA 938
**217055_x_at**	0.004953	1.33	SLC1A2	Solute carrier family 1 member 2
**220541_at**	0.049075	1.33	MMP26	Matrix metallopeptidase 26
**225015_s_at**	0.00251	1.33	STK40	Serine/threonine kinase 40
**238105_x_at**	0.020117	1.33	WNT7B	Wnt family member 7B
**215860_at**	0.004757	1.32	SYT12	Synaptotagmin 12
**216694_at**	0.00155	1.32	PQLC3	PQ loop repeat containing 3
**223597_at**	0.003795	1.32	ITLN1	Intelectin 1

EXCLI Journal 2021;20:126-141– ISSN 1611-2156
Received: December 08, 2020, accepted: January 11, 2021, published: January 18, 2021

**228950_s_at**	0.009125	1.32	WLS	Wntless Wnt ligand secretion mediator
**229837_s_at**	0.001907	1.32	KCTD5	Potassium channel tetramerization domain containing 5
**235250_at**	0.02152	1.32	FLCN	Folliculin
**210446_at**	0.01243	1.31	GATA1	GATA binding protein 1
**216464_x_at**	0.013824	1.31	PTGDR2	Prostaglandin D2 receptor 2
**219892_at**	0.027087	1.31	TM6SF1	Transmembrane 6 superfamily member 1
**238129_s_at**	0.002337	1.31	FZD2	Frizzled class receptor 2
**1552541_at**	0.004259	1.3	TAGAP	T-cell activation rhogtpase activating protein
**1556199_a_at**	0.031318	1.3	RGS9BP	Regulator of G-protein signaling 9 binding protein
**1558849_at**	0.04077	1.3	LINC00908	Long intergenic non-protein coding RNA 908
**1566908_at**	0.024629	1.3	STPG3	Sperm-tail PG-rich repeat containing 3
**204697_s_at**	0.006985	1.3	CHGA	Chromogranin A
**220528_at**	0.010246	1.3	VNN3	Vanin 3
**229652_s_at**	0.010817	1.3	FOXN3	Forkhead box N3
**231710_at**	0.006657	1.3	CAPS	Calcyphosine
**235507_at**	0.004352	1.3	PCMTD1	Protein-L-isoaspartate (D-aspartate) O-methyltransferase domain containing 1
**1558174_at**	0.002432	1.29	FAM43A	Family with sequence similarity 43 member A
**205110_s_at**	0.029658	1.29	FGF13	Fibroblast growth factor 13
**206620_at**	0.012144	1.29	GRAP	GRB2-related adaptor protein
**208090_s_at**	0.010341	1.29	AIRE	Autoimmune regulator
**216925_s_at**	0.029014	1.29	TAL1	TAL bhlh transcription factor 1, erythroid differentiation factor
**221956_at**	0.012807	1.29	LRCH4	Leucine rich repeats and calponin homology domain containing 4
**231780_at**	0.00414	1.29	GBGT1	Globoside alpha-1,3-N-acetylgalactosaminyltransferase 1
**237266_at**	0.014952	1.29	KCNIP2-AS1	KCNIP2 antisense RNA 1
**241670_x_at**	0.010242	1.29	NBAT1	Neuroblastoma associated transcript 1
**1559870_at**	0.014725	1.28	LOC100129129	Uncharacterized LOC100129129
**206080_at**	0.012858	1.28	PLCH2	Phospholipase C eta 2
**243777_at**	0.009769	1.28	RAB29	RAB29, member RAS oncogene family
**1554508_at**	0.026622	1.27	PIK3AP1	Phosphoinositide-3-kinase adaptor protein 1
**1567027_at**	0.017391	1.27	SH3GL1P2	SH3 domain containing GRB2 like 1, endophilin A2 pseudogene 2
**203154_s_at**	0.040961	1.27	PAK4	P21 (RAC1) activated kinase 4
**205566_at**	0.004414	1.27	ABHD2	Abhydrolase domain containing 2

**214348_at**	0.015564	1.27	TACR2	Tachykinin receptor 2
**218368_s_at**	0.008276	1.27	TNFRSF12A	TNF receptor superfamily member 12A
**228713_s_at**	0.008472	1.27	HSD17B14	Hydroxysteroid 17-beta dehydrogenase 14
**1554681_a_at**	0.009252	1.26	CCDC187	Coiled-coil domain containing 187
**1555214_a_at**	0.020118	1.26	CLEC7A	C-type lectin domain family 7 member A
**204909_at**	0.010185	1.26	DDX6	DEAD-box helicase 6
**206934_at**	0.017948	1.26	SIRPB1	Signal regulatory protein beta 1
**210684_s_at**	0.014535	1.26	DLG4	Discs large MAGUK scaffold protein 4
**223924_at**	0.021273	1.26	TTC25	Tetratricopeptide repeat domain 25
**228589_at**	0.004854	1.26	BASP1	Brain abundant membrane attached signal protein 1
**235583_at**	0.003287	1.26	ILDR1	Immunoglobulin like domain containing receptor 1
**243653_at**	0.008599	1.26	SHROOM3	Shroom family member 3
**1552978_a_at**	0.005388	1.25	SCAMP1	Secretory carrier membrane protein 1
**220413_at**	0.002185	1.25	SLC39A2	Solute carrier family 39 member 2
**236904_x_at**	0.008894	1.25	TECTA	Tectorin alpha
**204312_x_at**	0.016148	1.24	CREB1	Camp responsive element binding protein 1
**210994_x_at**	0.00223	1.24	TRIM23	Tripartite motif containing 23
**213779_at**	0.01196	1.24	EMID1	EMI domain containing 1
**220573_at**	0.011604	1.24	KLK14	Kallikrein related peptidase 14
**232517_s_at**	0.04036	1.24	HELZ2	Helicase with zinc finger 2
**238112_at**	0.013299	1.24	LOC283177	Uncharacterized LOC283177
**1558152_at**	0.012609	1.23	LOC100131262	Uncharacterized LOC100131262
**209597_s_at**	0.008216	1.23	PNMA2	Paraneoplastic Ma antigen 2
**216010_x_at**	0.034912	1.23	FUT3	Fucosyltransferase 3 (Lewis blood group)
**218843_at**	0.016351	1.23	FNDC4	Fibronectin type III domain containing 4
**224501_at**	0.024493	1.23	PERM1	PPARGC1 and ESRR induced regulator, muscle 1
**241795_at**	0.018281	1.23	RHEB	Ras homolog enriched in brain
**244085_at**	0.014437	1.23	ZNF653	Zinc finger protein 653
**1554631_at**	0.036988	1.22	ATM	ATM serine/threonine kinase
**202410_x_at**	0.008611	1.22	INS-IGF2///IGF2	INS-IGF2 readthrough///insulin like growth factor 2
**203040_s_at**	0.035398	1.22	HMBS	Hydroxymethylbilane synthase
**205604_at**	0.021422	1.22	HOXD9	Homeobox D9
**210885_s_at**	0.006518	1.22	TRIM15	Tripartite motif containing 15
**213805_at**	0.036965	1.22	ABHD5	Abhydrolase domain containing 5
**213809_x_at**	0.007639	1.22	TCF3	Transcription factor 3

**204535_s_at**	0.012151	1.21	REST	RE1 silencing transcription factor
**205016_at**	0.014634	1.21	TGFA	Transforming growth factor alpha
**205620_at**	0.002815	1.21	F10	Coagulation factor X
**208335_s_at**	0.012495	1.21	ACKR1	Atypical chemokine receptor 1 (Duffy blood group)
**223123_s_at**	0.024303	1.21	PITHD1	PITH domain containing 1
**230839_at**	0.012873	1.21	PRMT8	Protein arginine methyltransferase 8
**231741_at**	0.011392	1.21	S1PR3	Sphingosine-1-phosphate receptor 3
**233891_at**	0.010007	1.21	MUC3	Intestinal mucin-like
**1556855_a_at**	0.020644	1.2	ATP11A-AS1	ATP11A antisense RNA 1
**206851_at**	0.025284	1.2	RNASE3	Ribonuclease A family member 3
**209137_s_at**	0.029447	1.2	USP10	Ubiquitin specific peptidase 10
**218959_at**	0.038727	1.2	HOXC10	Homeobox C10
**221049_s_at**	0.045714	1.2	POLL	Polymerase (DNA) lambda
**228278_at**	0.004905	1.2	NFIX	Nuclear factor I X
**230170_at**	0.005548	1.2	OSM	Oncostatin M
**234321_x_at**	0.005107	1.2	NHSL1	NHS like 1
**211484_s_at**	0.008801	1.19	DSCAM	DS cell adhesion molecule
**211619_s_at**	0.002568	1.19	ALPPL2///ALPP	Alkaline phosphatase, placental like 2///alkaline phosphatase, placental
**231623_at**	0.0194	1.19	TMEM174	Transmembrane protein 174
**233911_s_at**	0.012193	1.19	PPM1H	Protein phosphatase, Mg2+/Mn2+ dependent 1H
**236981_at**	0.003377	1.19	C17orf99	Chromosome 17 open reading frame 99
**239416_at**	0.006163	1.19	FBXL6	F-box and leucine rich repeat protein 6
**240643_at**	0.019417	1.19	TTBK1	Tau tubulin kinase 1
**1552559_a_at**	0.0254	1.18	CDK15	Cyclin dependent kinase 15
**1555561_a_at**	0.007335	1.18	UGGT2	UDP-glucose glycoprotein glucosyltransferase 2
**231144_at**	0.006307	1.18	SMARCD3	SWI/SNF related, matrix associated, actin dependent regulator of chromatin, subfamily d, member 3
**231659_at**	0.007315	1.18	ST3GAL3	ST3 beta-galactoside alpha-2,3-sialyltransferase 3
**239663_x_at**	0.006569	1.18	LARP1	La ribonucleoprotein domain family member 1
**240818_at**	0.033552	1.18	OVCH1-AS1	OVCH1 antisense RNA 1
**243151_at**	0.011604	1.18	LOC100506282	Uncharacterized LOC100506282
**201710_at**	0.013272	1.17	MYBL2	MYB proto-oncogene like 2
**206471_s_at**	0.041958	1.17	PLXNC1	Plexin C1
**210086_at**	0.037837	1.17	HR	Hair growth associated
**210430_x_at**	0.027362	1.17	RHD	Rh blood group D antigen
**214438_at**	0.033858	1.17	HLX	H2.0 like homeobox

EXCLI Journal 2021;20:126-141– ISSN 1611-2156
Received: December 08, 2020, accepted: January 11, 2021, published: January 18, 2021

**229484_at**	0.00329	1.17	PPM1J	Protein phosphatase, Mg2+/Mn2+ dependent 1J
**230275_at**	0.005718	1.17	ARSI	Arylsulfatase family member I
**231080_at**	0.016276	1.17	CDAN1	Codanin 1
**236496_at**	0.030417	1.17	DEGS2	Delta 4-desaturase, sphingolipid 2
**238326_at**	0.024428	1.17	ODF3B	Outer dense fiber of sperm tails 3B
**239499_at**	0.011357	1.17	DNAH2	Dynein axonemal heavy chain 2
**1552615_at**	0.046241	1.16	ACACB	Acetyl-coa carboxylase beta
**204427_s_at**	0.019237	1.16	TMED2	Transmembrane p24 trafficking protein 2
**205003_at**	0.048408	1.16	DOCK4	Dedicator of cytokinesis 4
**213783_at**	0.019605	1.16	MFNG	MFNG O-fucosylpeptide 3-beta-N-acetylglucosaminyltransferase
**216243_s_at**	0.028082	1.16	IL1RN	Interleukin 1 receptor antagonist
**218045_x_at**	0.025465	1.16	PTMS	Parathymosin
**219992_at**	0.016597	1.16	TAC3	Tachykinin 3
**225168_at**	0.032929	1.16	FRMD4A	FERM domain containing 4A
**227281_at**	0.008725	1.16	SLC29A4	Solute carrier family 29 member 4
**232819_s_at**	0.025934	1.16	LTBR	Lymphotoxin beta receptor
**239145_at**	0.004224	1.16	ZNF414	Zinc finger protein 414
**239920_at**	0.014103	1.16	UBTF	Upstream binding transcription factor, RNA polymerase I
**241894_at**	0.010779	1.16	VMO1	Vitelline membrane outer layer 1 homolog
**1552853_at**	0.020205	1.15	VWA5B1	Von Willebrand factor A domain containing 5B1
**1556900_at**	0.010435	1.15	APCDD1L-AS1	APCDD1L antisense RNA 1 (head to head)
**1570328_s_at**	0.008466	1.15	LINC01620	Long intergenic non-protein coding RNA 1620
**204611_s_at**	0.048747	1.15	PPP2R5B	Protein phosphatase 2 regulatory subunit b'beta
**206877_at**	0.005228	1.15	MXD1	MAX dimerization protein 1
**211574_s_at**	0.030366	1.15	CD46	CD46 molecule
**215844_at**	0.006564	1.15	TNPO2	Transportin 2
**216019_x_at**	0.003136	1.15	MIR6716///PHLDB1	Microrna 6716///pleckstrin homology like domain family B member 1
**220187_at**	0.023338	1.15	STEAP4	STEAP4 metalloreductase
**228840_at**	0.019349	1.15	AMOTL1	Angiomotin like 1
**230564_at**	0.005133	1.15	SIPA1L3	Signal induced proliferation associated 1 like 3
**231458_at**	0.01933	1.15	LINC00658	Long intergenic non-protein coding RNA 658
**239370_at**	0.009298	1.15	LINC01133	Long intergenic non-protein coding RNA 1133
**244409_at**	0.03551	1.15	CCDC154	Coiled-coil domain containing 154
**1555465_at**	0.010507	1.14	MCOLN2	Mucolipin 2
**201474_s_at**	0.016704	1.14	ITGA3	Integrin subunit alpha 3

EXCLI Journal 2021;20:126-141– ISSN 1611-2156
Received: December 08, 2020, accepted: January 11, 2021, published: January 18, 2021

**206564_at**	0.011204	1.14	OPRL1	Opioid related nociceptin receptor 1
**207289_at**	0.007191	1.14	MMP25	Matrix metallopeptidase 25
**208474_at**	0.006244	1.14	CLDN6	Claudin 6
**208607_s_at**	0.009018	1.14	SAA2-SAA4///SAA2///SAA1	SAA2-SAA4 readthrough///serum amyloid A2///serum amyloid A1
**209019_s_at**	0.021413	1.14	PINK1	PTEN induced putative kinase 1
**216317_x_at**	0.02675	1.14	RHD///RHCE	Rh blood group D antigen///Rh blood group ccee antigens
**221284_s_at**	0.0248	1.14	SRC	SRC proto-oncogene, non-receptor tyrosine kinase
**221946_at**	0.035877	1.14	C9orf116	Chromosome 9 open reading frame 116
**226009_at**	0.032642	1.14	DPCD	Deleted in primary ciliary dyskinesia homolog (mouse)
**229451_at**	0.041232	1.14	GALNT9	Polypeptide N-acetylgalactosaminyltransferase 9
**230045_at**	0.003389	1.14	CNTN2	Contactin 2
**237767_at**	0.009234	1.14	FARP1	FERM, ARH/rhogef and pleckstrin domain protein 1
**238051_x_at**	0.009388	1.14	PWWP2B	PWWP domain containing 2B
**1554228_a_at**	0.020834	1.13	BAALC-AS2	BAALC antisense RNA 2
**206004_at**	0.004651	1.13	TGM3	Transglutaminase 3
**208403_x_at**	0.045665	1.13	MAX	MYC associated factor X
**210689_at**	0.004614	1.13	CLDN14	Claudin 14
**211049_at**	0.011714	1.13	TLX2	T-cell leukemia homeobox 2
**214537_at**	0.017575	1.13	HIST1H1D	Histone cluster 1, H1d
**217169_at**	0.014928	1.13	IGHM///IGHG1///IGHA1	Immunoglobulin heavy constant mu///immunoglobulin heavy constant gamma 1 (G1m marker)///immunoglobulin heavy constant alpha 1
**217767_at**	0.008474	1.13	C3	Complement component 3
**225165_at**	0.015342	1.13	PPP1R1B	Protein phosphatase 1 regulatory inhibitor subunit 1B
**230745_s_at**	0.016506	1.13	TOX3	TOX high mobility group box family member 3
**232697_at**	0.02281	1.13	LRFN2	Leucine rich repeat and fibronectin type III domain containing 2
**234716_at**	0.019952	1.13	ZIC1	Zic family member 1
**239464_at**	0.026091	1.13	KCNJ15	Potassium voltage-gated channel subfamily J member 15
**244797_at**	0.005445	1.13	LINC01159	Long intergenic non-protein coding RNA 1159
**1552516_a_at**	0.003198	1.12	HIPK1	Homeodomain interacting protein kinase 1
**200930_s_at**	0.039263	1.12	VCL	Vinculin
**206297_at**	0.028436	1.12	CTRC	Chymotrypsin C
**206429_at**	0.024949	1.12	F2RL1	F2R like trypsin receptor 1

**208416_s_at**	0.026391	1.12	SPTB	Spectrin beta, erythrocytic
**208504_x_at**	0.011987	1.12	PCDHB11	Protocadherin beta 11
**210794_s_at**	0.005617	1.12	MEG3	Maternally expressed 3 (non-protein coding)
**216345_at**	0.012929	1.12	ZSWIM8	Zinc finger SWIM-type containing 8
**217054_at**	0.043207	1.12	MUC3A	Mucin 3A, cell surface associated
**218417_s_at**	0.035279	1.12	SLC48A1	Solute carrier family 48 member 1
**224378_x_at**	0.01353	1.12	MAP1LC3A	Microtubule associated protein 1 light chain 3 alpha
**228490_at**	0.036865	1.12	ABHD2	Abhydrolase domain containing 2
**230385_at**	0.0361	1.12	3-Mar	Membrane associated ring-CH-type finger 3
**235160_at**	0.013872	1.12	ATF7	Activating transcription factor 7
**237840_at**	0.042906	1.12	LINC01118	Long intergenic non-protein coding RNA 1118
**241652_x_at**	0.04539	1.12	LIN7A	Lin-7 homolog A, crumbs cell polarity complex component
**1557601_s_at**	0.026523	1.11	LOC100132731///KIAA1257	Uncharacterized LOC100132731///KIAA1257
**201249_at**	0.015295	1.11	SLC2A1	Solute carrier family 2 member 1
**202718_at**	0.031997	1.11	IGFBP2	Insulin like growth factor binding protein 2
**205537_s_at**	0.009719	1.11	VAV2	Vav guanine nucleotide exchange factor 2
**205734_s_at**	0.015794	1.11	AFF3	AF4/FMR2 family member 3
**211670_x_at**	0.029845	1.11	SSX3	SSX family member 3
**221624_at**	0.011366	1.11	TCL6	T-cell leukemia/lymphoma 6 (non-protein coding)
**222210_at**	0.038049	1.11	MIR6785///TMEM94	Microrna 6785///transmembrane protein 94
**229482_at**	0.03759	1.11	DDX51	DEAD-box helicase 51
**230334_at**	0.017413	1.11	LOC100507291	Uncharacterized LOC100507291
**231275_at**	0.031744	1.11	LINC00982	Long intergenic non-protein coding RNA 982
**231734_at**	0.046514	1.11	RBP2	Retinol binding protein 2
**244269_at**	0.013189	1.11	LOC102723526	Uncharacterized LOC102723526
**1554115_at**	0.006242	1.1	C5orf58	Chromosome 5 open reading frame 58
**201945_at**	0.018835	1.1	FURIN	Furin, paired basic amino acid cleaving enzyme
**203191_at**	0.006239	1.1	ABCB6	ATP binding cassette subfamily B member 6 (Langereis blood group)
**204636_at**	0.007758	1.1	COL17A1	Collagen type XVII alpha 1 chain
**205164_at**	0.041743	1.1	GCAT	Glycine C-acetyltransferase
**207439_s_at**	0.030552	1.1	SLC35A2	Solute carrier family 35 member A2
**207787_at**	0.021613	1.1	KRT33B	Keratin 33B

EXCLI Journal 2021;20:126-141– ISSN 1611-2156
Received: December 08, 2020, accepted: January 11, 2021, published: January 18, 2021

**208052_x_at**	0.030411	1.1	CEACAM3	Carcinoembryonic antigen related cell adhesion molecule 3
**209491_s_at**	0.00908	1.1	CAND1.11///AMPD3	Uncharacterized LOC100130460///adenosine monophosphate deaminase 3
**212012_at**	0.009514	1.1	PXDN	Peroxidasin
**215977_x_at**	0.032096	1.1	GK	Glycerol kinase
**218916_at**	0.003437	1.1	ZNF768///ZNF747	Zinc finger protein 768///zinc finger protein 747
**220640_at**	0.017928	1.1	CSNK1G1	Casein kinase 1 gamma 1
**227389_x_at**	0.008892	1.1	IRF2BP2	Interferon regulatory factor 2 binding protein 2
**237900_at**	0.004894	1.1	KLHDC4	Kelch domain containing 4
**239933_x_at**	0.039831	1.1	BBOF1	Basal body orientation factor 1
**1564439_a_at**	0.018881	1.09	MRGPRG-AS1	MRGPRG antisense RNA 1
**207025_at**	0.015613	1.09	GJC2	Gap junction protein gamma 2
**208519_x_at**	0.023428	1.09	GNRH2	Gonadotropin releasing hormone 2
**211302_s_at**	0.032072	1.09	PDE4B	Phosphodiesterase 4B
**211889_x_at**	0.018788	1.09	CEACAM1	Carcinoembryonic antigen related cell adhesion molecule 1
**215712_s_at**	0.036974	1.09	IGFALS	Insulin like growth factor binding protein acid labile subunit
**215819_s_at**	0.038679	1.09	RHD///RHCE	Rh blood group D antigen///Rh blood group ccee antigens
**217702_at**	0.029531	1.09	IL27RA	Interleukin 27 receptor subunit alpha
**219150_s_at**	0.016523	1.09	ADAP1	Arfgap with dual PH domains 1
**222933_at**	0.008894	1.09	MORN1	MORN repeat containing 1
**230341_x_at**	0.032683	1.09	ADAMTS10	ADAM metallopeptidase with thrombospondin type 1 motif 10
**231752_at**	0.01346	1.09	NPBWR1	Neuropeptides B/W receptor 1
**233885_at**	0.01616	1.09	ARHGAP33	Rho gtpase activating protein 33
**235186_at**	0.023045	1.09	FAM231D///LOC388692	Family with sequence similarity 231 member D///uncharacterized LOC388692
**243285_at**	0.030725	1.09	LOC283335	Uncharacterized LOC283335
**1559714_at**	0.018898	1.08	RGR	Retinal G protein coupled receptor
**1562447_a_at**	0.015243	1.08	PPP1R26-AS1	PPP1R26 antisense RNA 1
**202242_at**	0.01562	1.08	TSPAN7	Tetraspanin 7
**203948_s_at**	0.015765	1.08	MPO	Myeloperoxidase
**204144_s_at**	0.043202	1.08	PIGQ	Phosphatidylinositol glycan anchor biosynthesis class Q
**205832_at**	0.014849	1.08	CPA4	Carboxypeptidase A4
**207914_x_at**	0.023506	1.08	EVX1	Even-skipped homeobox 1
**208243_s_at**	0.015918	1.08	CNR1	Cannabinoid receptor 1
**208352_x_at**	0.031869	1.08	ANK1	Ankyrin 1

EXCLI Journal 2021;20:126-141– ISSN 1611-2156
Received: December 08, 2020, accepted: January 11, 2021, published: January 18, 2021

**211253_x_at**	0.038097	1.08	PYY	Peptide YY
**215530_at**	0.013299	1.08	FANCA	Fanconi anemia complementation group A
**217174_s_at**	0.015227	1.08	APC2	APC2, WNT signaling pathway regulator
**220100_at**	0.021777	1.08	SLC22A11	Solute carrier family 22 member 11
**220385_at**	0.014189	1.08	JPH2	Junctophilin 2
**225566_at**	0.031961	1.08	NRP2	Neuropilin 2
**225799_at**	0.039373	1.08	LOC101930489///MIR4435-2HG///LINC00152	Uncharacterized LOC101930489///MIR4435-2 host gene///long intergenic non-protein coding RNA 152
**231445_at**	0.007527	1.08	PAX1	Paired box 1
**236254_at**	0.014248	1.08	VPS13B	Vacuolar protein sorting 13 homolog B
**242200_at**	0.013083	1.08	ADAMTSL5	ADAMTS like 5
**242774_at**	0.015249	1.08	SYNE2	Spectrin repeat containing nuclear envelope protein 2
**243239_at**	0.039537	1.08	SAMM50	SAMM50 sorting and assembly machinery component
**244250_at**	0.040003	1.08	ANXA6	Annexin A6
**1557123_a_at**	0.018039	1.07	CHADL	Chondroadherin like
**201551_s_at**	0.038684	1.07	LAMP1	Lysosomal associated membrane protein 1
**209973_at**	0.011961	1.07	NFKBIL1	NFKB inhibitor like 1
**209981_at**	0.011252	1.07	CSDC2	Cold shock domain containing C2
**210000_s_at**	0.004875	1.07	SOCS1	Suppressor of cytokine signaling 1
**210827_s_at**	0.012386	1.07	ELF3	E74 like ETS transcription factor 3
**211145_x_at**	0.04534	1.07	IFNA21	Interferon alpha 21
**211897_s_at**	0.011276	1.07	CRHR1	Corticotropin releasing hormone receptor 1
**213724_s_at**	0.014594	1.07	PDK2	Pyruvate dehydrogenase kinase 2
**223499_at**	0.004677	1.07	C1QTNF5///MFRP	C1q and tumor necrosis factor related protein 5///membrane frizzled-related protein
**225353_s_at**	0.007406	1.07	C1QC	Complement C1q C chain
**242684_at**	0.010981	1.07	ZNF425	Zinc finger protein 425
**1552558_a_at**	0.04961	1.06	RAI1	Retinoic acid induced 1
**1553523_at**	0.035294	1.06	NLRP14	NLR family pyrin domain containing 14
**1557540_at**	0.043906	1.06	LOC100507403	Uncharacterized LOC100507403
**201208_s_at**	0.026547	1.06	TNFAIP1	TNF alpha induced protein 1
**203588_s_at**	0.017001	1.06	TFDP2	Transcription factor Dp-2
**206778_at**	0.013636	1.06	CRYBB2	Crystallin beta B2
**207593_at**	0.015735	1.06	ABCG4	ATP binding cassette subfamily G member 4
**208110_x_at**	0.007508	1.06	MED25	Mediator complex subunit 25

EXCLI Journal 2021;20:126-141– ISSN 1611-2156
Received: December 08, 2020, accepted: January 11, 2021, published: January 18, 2021

**210357_s_at**	0.005516	1.06	SMOX	Spermine oxidase
**214091_s_at**	0.023651	1.06	GPX3	Glutathione peroxidase 3
**216830_at**	0.027993	1.06	LOC101929047///HERC2P10///HERC2	Uncharacterized LOC101929047///hect domain and RLD 2 pseudogene 10///HECT and RLD domain containing E3 ubiquitin protein ligase 2
**221165_s_at**	0.015783	1.06	IL22	Interleukin 22
**221409_at**	0.028786	1.06	OR2S2	Olfactory receptor family 2 subfamily S member 2 (gene/pseudogene)
**231910_at**	0.036242	1.06	NUDT14	Nudix hydrolase 14
**235641_at**	0.034837	1.06	TRIB1	Tribbles pseudokinase 1
**238136_at**	0.029914	1.06	SHISA7	Shisa family member 7
**244765_at**	0.011879	1.06	LOC102724782	Uncharacterized LOC102724782
**39854_r_at**	0.03005	1.06	PNPLA2	Patatin like phospholipase domain containing 2
**62987_r_at**	0.042283	1.06	CACNG4	Calcium voltage-gated channel auxiliary subunit gamma 4
**1559755_at**	0.009551	1.05	C21orf62-AS1	C21orf62 antisense RNA 1
**1567254_at**	0.037953	1.05	OR10D1P	Olfactory receptor family 10 subfamily D member 1 pseudogene
**200952_s_at**	0.040866	1.05	CCND2	Cyclin D2
**202280_at**	0.014679	1.05	GAK	Cyclin G associated kinase
**205390_s_at**	0.015884	1.05	ANK1	Ankyrin 1
**205616_at**	0.023947	1.05	GALNT8///KCNA6	Polypeptide N-acetylgalactosaminyltransferase 8///potassium voltage-gated channel subfamily A member 6
**206463_s_at**	0.018158	1.05	DHRS2	Dehydrogenase/reductase 2
**206903_at**	0.014484	1.05	EXOG	Endo/exonuclease (5'-3'), endonuclease G-like
**210579_s_at**	0.016522	1.05	TRIM10	Tripartite motif containing 10
**212720_at**	0.029108	1.05	PAPOLA	Poly(A) polymerase alpha
**213344_s_at**	0.034181	1.05	H2AFX	H2A histone family member X
**216473_x_at**	0.027063	1.05	DUX4L24///DBET///LOC100291626///DUX4///LOC100288289///DUX4L2///DUX4L3///DUX4L5///DUX4L6///DUX4L7///LOC652301///DUX4L4///DUX4L8///DUX4L1	Double homeobox 4 like 24///D4Z4 binding element transcript (non-protein coding)///double homeobox protein 4-like///double homeobox 4///double homeobox protein 4-like protein 2-like///double homeobox 4 like 2///double homeobox 4 like 3///double homeobox 4 like 5///double homeobox 4 like 6///double homeobox 4 like 7///double homeobox protein 4-like protein 4-like///double homeobox 4 like 4///double homeobox 4 like 8///double homeobox 4 like 1
**219385_at**	0.0117	1.05	SLAMF8	SLAM family member 8

EXCLI Journal 2021;20:126-141– ISSN 1611-2156
Received: December 08, 2020, accepted: January 11, 2021, published: January 18, 2021

**220438_at**	0.0189	1.05	QPCTL	Glutaminyl-peptide cyclotransfer-ase like
**221377_s_at**	0.030232	1.05	RBPJL	Recombination signal binding pro-tein for immunoglobulin kappa J re-gion like
**222831_at**	0.042323	1.05	SAP30L	SAP30 like
**223970_at**	0.022284	1.05	RETNLB	Resistin like beta
**224102_at**	0.009084	1.05	P2RY12	Purinergic receptor P2Y12
**224498_x_at**	0.013157	1.05	AXIN2	Axin 2
**226704_at**	0.011984	1.05	UBE2J2	Ubiquitin conjugating enzyme E2 J2
**234695_x_at**	0.0114	1.05	NUTM2B	NUT family member 2B
**234781_at**	0.041972	1.05	OR3A3///OR1E2///OR1E1	Olfactory receptor family 3 subfam-ily A member 3///olfactory receptor family 1 subfamily E member 2///ol-factory receptor family 1 subfamily E member 1
**237855_at**	0.015502	1.05	ZNF777	Zinc finger protein 777
**239492_at**	0.014228	1.05	SEC14L4	SEC14 like lipid binding 4
**240164_at**	0.009193	1.05	MUC4	Mucin 4, cell surface associated
**33579_i_at**	0.037981	1.05	GALR3	Galanin receptor 3
**1552582_at**	0.029441	1.04	ABCC13	ATP binding cassette subfamily C member 13 (pseudogene)
**1553826_a_at**	0.007482	1.04	PRR35	Proline rich 35
**1559471_s_at**	0.035467	1.04	D21S2088E	D21s2088e
**205897_at**	0.018011	1.04	NFATC4	Nuclear factor of activated T-cells 4
**206657_s_at**	0.015451	1.04	MYOD1	Myogenic differentiation 1
**210594_x_at**	0.031083	1.04	MPZL1	Myelin protein zero like 1
**211062_s_at**	0.034619	1.04	GPR78///CPZ	G protein-coupled receptor 78///car-boxypeptidase Z
**214556_at**	0.005621	1.04	SSTR4	Somatostatin receptor 4
**216013_at**	0.012085	1.04	ZXDB	Zinc finger, X-linked, duplicated B
**217270_s_at**	0.01514	1.04	DYRK1B	Dual specificity tyrosine phosphory-lation regulated kinase 1B
**221450_x_at**	0.01609	1.04	PCDHB13	Protocadherin beta 13
**222598_s_at**	0.019176	1.04	NAV2	Neuron navigator 2
**223333_s_at**	0.004569	1.04	ANGPTL4	Angiopoietin like 4
**229617_x_at**	0.025796	1.04	AP2A1	Adaptor related protein complex 2 alpha 1 subunit
**229875_at**	0.027074	1.04	ZDHHC22	Zinc finger DHHC-type containing 22
**230347_at**	0.017855	1.04	ORAI2	ORAI calcium release-activated calcium modulator 2
**231637_at**	0.028721	1.04	LOC100499194	Uncharacterized LOC100499194
**242386_x_at**	0.024723	1.04	LOC101927608	Uncharacterized LOC101927608
**243116_at**	0.023618	1.04	PIP5KL1	Phosphatidylinositol-4-phosphate 5-kinase like 1

**243171_at**	0.014664	1.04	LOC100128325	Uncharacterized LOC100128325
**36475_at**	0.01363	1.04	GCAT	Glycine C-acetyltransferase
**1553943_at**	0.010799	1.03	LOC171391	Uncharacterized LOC171391
**1554327_a_at**	0.006817	1.03	CANT1	Calcium activated nucleotidase 1
**1555746_at**	0.00863	1.03	CD79B	CD79b molecule
**1567068_at**	0.009874	1.03	OR4D1	Olfactory receptor family 4 subfamily D member 1
**205833_s_at**	0.035677	1.03	PART1	Prostate androgen-regulated transcript 1 (non-protein coding)
**212925_at**	0.027785	1.03	MISP	Mitotic spindle positioning
**213059_at**	0.018937	1.03	CREB3L1	Camp responsive element binding protein 3 like 1
**214839_at**	0.025961	1.03	MIR124-1///LINC00599	Microrna 124-1///long intergenic non-protein coding RNA 599
**216063_at**	0.036161	1.03	HBBP1	Hemoglobin subunit beta pseudogene 1
**216878_x_at**	0.03221	1.03	HAB1	B1 for mucin
**216993_s_at**	0.010469	1.03	COL11A2	Collagen type XI alpha 2 chain
**217430_x_at**	0.036273	1.03	COL1A1	Collagen type I alpha 1 chain
**220965_s_at**	0.020088	1.03	RSPH6A	Radial spoke head 6 homolog A
**221232_s_at**	0.043039	1.03	ANKRD2	Ankyrin repeat domain 2
**228436_at**	0.041585	1.03	KCNC4	Potassium voltage-gated channel subfamily C member 4

**1367 down-regulated genes**				
**ID**	***p* value**	**logFC**	**Gene symbol**	**Gene title**
**228157_at**	0.0131813	-3.21	ZNF207	Zinc finger protein 207
**205583_s_at**	0.0118503	-3.13	ALG13	ALG13, UDP-N-acetylglucosaminyltransferase subunit
**1567214_a_at**	0.0447192	-3.01	PNN	Pinin, desmosome associated protein
**229235_at**	0.0002107	-2.96	NFATC2IP	Nuclear factor of activated T-cells 2 interacting protein
**1558956_s_at**	0.0095004	-2.96	IFT80	Intraflagellar transport 80
**225594_at**	0.00065	-2.88	CREBZF	CREB/ATF bzip transcription factor
**213326_at**	0.0016883	-2.88	VAMP1	Vesicle associated membrane protein 1
**226344_at**	0.009078	-2.84	ZMAT1	Zinc finger matrin-type 1
**235410_at**	0.0077705	-2.83	NPHP3	Nephronophthisis 3 (adolescent)
**208798_x_at**	0.0422171	-2.82	GOLGA8A	Golgin A8 family member A
**225180_at**	0.0264898	-2.81	TTC14	Tetratricopeptide repeat domain 14
**235987_at**	0.0023233	-2.72	PRKXP1	Protein kinase, X-linked, pseudogene 1
**221768_at**	0.0449282	-2.71	SFPQ	Splicing factor proline and glutamine rich

**222848_at**	0.0131919	-2.7	CENPK	Centromere protein K
**227208_at**	0.0010889	-2.69	CCDC84	Coiled-coil domain containing 84
**228999_at**	0.0000695	-2.68	CHD2	Chromodomain helicase DNA binding protein 2
**221208_s_at**	0.0011506	-2.67	MSANTD2	Myb/SANT DNA binding domain containing 2
**203804_s_at**	0.0052885	-2.67	LUC7L3	LUC7 like 3 pre-mrna splicing factor
**225107_at**	0.0418059	-2.67	HNRNPA2B1	Heterogeneous nuclear ribonucleoprotein A2/B1
**228617_at**	0.0080524	-2.65	XAF1	XIAP associated factor 1
**213158_at**	0.0281159	-2.65	ZBTB20	Zinc finger and BTB domain containing 20
**223295_s_at**	0.0197121	-2.64	LUC7L	LUC7 like
**214016_s_at**	0.0270403	-2.59	SFPQ	Splicing factor proline and glutamine rich
**226316_at**	0.0102043	-2.58	RBM26	RNA binding motif protein 26
**218757_s_at**	0.0011845	-2.55	UPF3B	UPF3 regulator of nonsense transcripts homolog B (yeast)
**216069_at**	0.0035742	-2.55	PRMT2	Protein arginine methyltransferase 2
**209903_s_at**	0.0022391	-2.53	ATR	ATR serine/threonine kinase
**213483_at**	0.0233388	-2.53	PPWD1	Peptidylprolyl isomerase domain and WD repeat containing 1
**228661_s_at**	0.0056292	-2.52	LOC102606465	Uncharacterized LOC102606465
**218683_at**	0.0193091	-2.52	PTBP2	Polypyrimidine tract binding protein 2
**1570571_at**	0.0057362	-2.51	CCDC91	Coiled-coil domain containing 91
**227594_at**	0.0010862	-2.5	ZMYM6	Zinc finger MYM-type containing 6
**225705_at**	0.0041208	-2.49	CEP95	Centrosomal protein 95
**214314_s_at**	0.0499205	-2.49	EIF5B	Eukaryotic translation initiation factor 5B
**1557828_a_at**	0.0470481	-2.48	TMEM267	Transmembrane protein 267
**230036_at**	0.0135728	-2.47	SAMD9L	Sterile alpha motif domain containing 9 like
**231828_at**	0.0440054	-2.47	PSMD5-AS1	PSMD5 antisense RNA 1 (head to head)
**213703_at**	0.0362174	-2.47	LINC00342	Long intergenic non-protein coding RNA 342
**235274_at**	0.0015972	-2.46	GABPB1-AS1	GABPB1 antisense RNA 1
**1557733_a_at**	0.0195195	-2.46	CHRM3-AS2	CHRM3 antisense RNA 2
**200686_s_at**	0.0001544	-2.45	SRSF11	Serine and arginine rich splicing factor 11
**213049_at**	0.0140651	-2.45	RALGAPA1	Ral gtpase activating protein catalytic alpha subunit 1
**1554447_at**	0.0074338	-2.44	JPX	JPX transcript, XIST activator (non-protein coding)

EXCLI Journal 2021;20:126-141– ISSN 1611-2156
Received: December 08, 2020, accepted: January 11, 2021, published: January 18, 2021

**212179_at**	0.0170646	-2.44	PNISR	PNN interacting serine and arginine rich protein
**214945_at**	0.0198242	-2.43	LOC101930363///LOC101928349///LOC100507387///FAM153C///FAM153A///FAM153B	Uncharacterized LOC101930363///uncharacterized LOC101928349///uncharacterized LOC100507387///family with sequence similarity 153, member C///family with sequence similarity 153 member A///family with sequence similarity 153 member B
**230245_s_at**	0.0021599	-2.42	LINC00926	Long intergenic non-protein coding RNA 926
**212232_at**	0.029254	-2.41	FNBP4	Formin binding protein 4
**218649_x_at**	0.0332333	-2.41	NEMF	Nuclear export mediator factor
**213666_at**	0.0079343	-2.39	6-Sep	Septin 6
**1558739_at**	0.0086778	-2.39	BORCS5	BLOC-1 related complex subunit 5
**200685_at**	0.0141075	-2.39	SRSF11	Serine and arginine rich splicing factor 11
**211038_s_at**	0.0188877	-2.39	CROCCP2	Ciliary rootlet coiled-coil, rootletin pseudogene 2
**204352_at**	0.0096021	-2.38	TRAF5	TNF receptor associated factor 5
**238761_at**	0.0248894	-2.38	ELK4	ELK4, ETS transcription factor
**226098_at**	0.0184017	-2.37	IFT80	Intraflagellar transport 80
**236220_at**	0.0042403	-2.37	SLC16A10	Solute carrier family 16 member 10
**204793_at**	0.0102985	-2.37	GPRASP1	G protein-coupled receptor associated sorting protein 1
**229141_at**	0.0150531	-2.36	SFT2D3///WDR33	SFT2 domain containing 3///WD repeat domain 33
**228318_s_at**	0.028021	-2.36	CRIPAK	Cysteine rich PAK1 inhibitor
**228477_at**	0.041193	-2.36	ARGLU1	Arginine and glutamate rich 1
**212060_at**	0.0057294	-2.35	U2SURP	U2 snrnp associated SURP domain containing
**218352_at**	0.0081327	-2.35	RCBTB1	RCC1 and BTB domain containing protein 1
**226334_s_at**	0.0237127	-2.34	AHSA2	AHA1, activator of heat shock 90kda protein atpase homolog 2 (yeast)
**213212_x_at**	0.0328104	-2.34	LOC102724093///GOLGA6L4///GOLGA6L9///GOLGA6L5P	Golgin subfamily A member 6-like protein 4///golgin A6 family-like 4///golgin A6 family-like 9///golgin A6 family-like 5, pseudogene
**212980_at**	0.0038322	-2.33	AHSA2	AHA1, activator of heat shock 90kda protein atpase homolog 2 (yeast)
**218456_at**	0.008816	-2.33	CAPRIN2	Caprin family member 2
**212842_x_at**	0.0032582	-2.31	RGPD6///RGPD8///RGPD3///RGPD4///RGPD5	RANBP2-like and GRIP domain containing 6///RANBP2-like and GRIP domain containing 8///RANBP2-like and GRIP domain containing 3///RANBP2-like and

				GRIP domain containing 4///RANBP2-like and GRIP domain containing 5
**213262_at**	0.0067328	-2.31	SACS	Sacsin molecular chaperone
**202127_at**	0.0188111	-2.31	PRPF4B	Pre-mrna processing factor 4B
**218269_at**	0.0002486	-2.3	DROSHA	Drosha ribonuclease III
**220418_at**	0.0084638	-2.3	UBASH3A	Ubiquitin associated and SH3 domain containing A
**205739_x_at**	0.0107582	-2.3	ZNF107	Zinc finger protein 107
**203791_at**	0.0106617	-2.3	DMXL1	Dmx like 1
**209265_s_at**	0.0205641	-2.3	METTL3	Methyltransferase like 3
**217317_s_at**	0.0224851	-2.3	HERC2P9///HERC2P2	Hect domain and RLD 2 pseudogene 9///hect domain and RLD 2 pseudogene 2
**228751_at**	0.0194248	-2.28	CLK4	CDC like kinase 4
**74694_s_at**	0.011128	-2.27	RABEP2	Rabaptin, RAB gtpase binding effector protein 2
**228030_at**	0.0161026	-2.27	RBM6	RNA binding motif protein 6
**1552287_s_at**	0.0499995	-2.27	AFG3L1P	AFG3 like matrix AAA peptidase subunit 1, pseudogene
**203164_at**	0.0006043	-2.26	SLC33A1	Solute carrier family 33 member 1
**218331_s_at**	0.0116093	-2.26	FAM208B	Family with sequence similarity 208 member B
**236198_at**	0.0197373	-2.26	LINC01215	Long intergenic non-protein coding RNA 1215
**205590_at**	0.0492548	-2.26	RASGRP1	RAS guanyl releasing protein 1
**211064_at**	0.0006496	-2.25	ZNF493	Zinc finger protein 493
**241905_at**	0.0197212	-2.25	PIK3C2A	Phosphatidylinositol-4-phosphate 3-kinase catalytic subunit type 2 alpha
**218877_s_at**	0.005771	-2.24	TRMT11	Trna methyltransferase 11 homolog
**219378_at**	0.0472453	-2.24	NAA16	N(alpha)-acetyltransferase 16, nata auxiliary subunit
**212783_at**	0.043656	-2.24	RBBP6	RB binding protein 6, ubiquitin ligase
**222685_at**	0.0051805	-2.23	HAUS6	HAUS augmin like complex subunit 6
**227412_at**	0.0134422	-2.23	PPP1R3E	Protein phosphatase 1 regulatory subunit 3E
**225017_at**	0.016936	-2.23	CCDC14	Coiled-coil domain containing 14
**212672_at**	0.0484344	-2.23	ATM	ATM serine/threonine kinase
**213677_s_at**	0.0060206	-2.22	PMS1	PMS1 homolog 1, mismatch repair system component
**212176_at**	0.0228282	-2.22	PNISR	PNN interacting serine and arginine rich protein
**219233_s_at**	0.0180612	-2.22	GSDMB	Gasdermin B
**225760_at**	0.0487274	-2.22	MYSM1	Myb like, SWIRM and MPN domains 1

EXCLI Journal 2021;20:126-141– ISSN 1611-2156
Received: December 08, 2020, accepted: January 11, 2021, published: January 18, 2021

**1554250_s_at**	0.0367793	-2.22	STAG3L2///STAG3L3///TRIM73	Stromal antigen 3-like 2 (pseudogene)///stromal antigen 3-like 3 (pseudogene)///tripartite motif containing 73
**215043_s_at**	0.0008173	-2.21	GUSBP3///SMA5///SMA4	Glucuronidase, beta pseudogene 3///glucuronidase beta pseudogene///glucuronidase beta pseudogene
**203095_at**	0.0100613	-2.21	MTIF2	Mitochondrial translational initiation factor 2
**213243_at**	0.0309804	-2.21	VPS13B	Vacuolar protein sorting 13 homolog B
**215731_s_at**	0.0356856	-2.21	MPHOSPH9	M-phase phosphoprotein 9
**213387_at**	0.0042616	-2.19	ATAD2B	Atpase family, AAA domain containing 2B
**1558569_at**	0.0377076	-2.19	LOC100131541	Uncharacterized LOC100131541
**214093_s_at**	0.0123821	-2.18	FUBP1	Far upstream element binding protein 1
**218244_at**	0.0268634	-2.18	NOL8	Nucleolar protein 8
**201967_at**	0.0499141	-2.18	RBM6	RNA binding motif protein 6
**228174_at**	0.0045392	-2.17	SCAI	Suppressor of cancer cell invasion
**225297_at**	0.0198577	-2.17	HAUS1	HAUS augmin like complex subunit 1
**226587_at**	0.0009421	-2.16	PWAR6	Prader Willi/Angelman region RNA 6
**220035_at**	0.0456172	-2.16	NUP210	Nucleoporin 210
**223218_s_at**	0.0039384	-2.15	NFKBIZ	NFKB inhibitor zeta
**205584_at**	0.0240853	-2.15	ALG13	ALG13, UDP-N-acetylglucosaminyltransferase subunit
**227979_at**	0.0051931	-2.14	RBM4	RNA binding motif protein 4
**55872_at**	0.0173946	-2.14	ZNF512B	Zinc finger protein 512B
**227105_at**	0.0308366	-2.14	CSPP1	Centrosome and spindle pole associated protein 1
**204291_at**	0.0338913	-2.14	ZNF518A	Zinc finger protein 518A
**220206_at**	0.0050024	-2.13	ZMYM1	Zinc finger MYM-type containing 1
**225178_at**	0.0109898	-2.13	TTC14	Tetratricopeptide repeat domain 14
**226404_at**	0.0207764	-2.13	RBM39	RNA binding motif protein 39
**228106_at**	0.0237035	-2.13	DCAF16	DDB1 and CUL4 associated factor 16
**1559263_s_at**	0.0241961	-2.13	ZC3H12D	Zinc finger CCCH-type containing 12D
**1558700_s_at**	0.0040884	-2.12	ZNF260	Zinc finger protein 260
**240452_at**	0.0121203	-2.12	GSPT1	G1 to S phase transition 1
**1567213_at**	0.0177225	-2.12	PNN	Pinin, desmosome associated protein
**228974_at**	0.0012077	-2.11	ZNF677	Zinc finger protein 677
**228722_at**	0.0073927	-2.11	PRMT2	Protein arginine methyltransferase 2

EXCLI Journal 2021;20:126-141– ISSN 1611-2156
Received: December 08, 2020, accepted: January 11, 2021, published: January 18, 2021

**228506_at**	0.0197465	-2.11	NSMCE4A	NSE4 homolog A, SMC5-SMC6 complex component
**228492_at**	0.0424716	-2.11	USP9Y	Ubiquitin specific peptidase 9, Y-linked
**238378_at**	0.0001279	-2.1	GPRIN3	GPRIN family member 3
**232879_at**	0.0086688	-2.1	CRTC3	CREB regulated transcription coactivator 3
**227988_s_at**	0.0337402	-2.1	VPS13A	Vacuolar protein sorting 13 homolog A
**210017_at**	0.0179986	-2.1	MALT1	MALT1 paracaspase
**206958_s_at**	0.016028	-2.09	UPF3A	UPF3 regulator of nonsense transcripts homolog A (yeast)
**229665_at**	0.001575	-2.08	CSTF3	Cleavage stimulation factor subunit 3
**1569652_at**	0.0052409	-2.08	MLLT3	MLLT3, super elongation complex subunit
**201788_at**	0.0435666	-2.08	DDX42	DEAD-box helicase 42
**207078_at**	0.0026387	-2.07	MED6	Mediator complex subunit 6
**243362_s_at**	0.005313	-2.07	LEF1-AS1	LEF1 antisense RNA 1
**226337_at**	0.0059817	-2.07	GORAB	Golgin, RAB6 interacting
**205178_s_at**	0.0184603	-2.07	RBBP6	RB binding protein 6, ubiquitin ligase
**227565_at**	0.0271291	-2.07	KLHL5	Kelch like family member 5
**212074_at**	0.0220728	-2.07	SUN1	Sad1 and UNC84 domain containing 1
**1555960_at**	0.0410661	-2.07	HINT1	Histidine triad nucleotide binding protein 1
**226181_at**	0.0126838	-2.06	TUBE1	Tubulin epsilon 1
**218515_at**	0.0123325	-2.06	PAXBP1	PAX3 and PAX7 binding protein 1
**213077_at**	0.0456418	-2.06	YTHDC2	YTH domain containing 2
**222309_at**	0.0095466	-2.05	C6orf62	Chromosome 6 open reading frame 62
**223988_x_at**	0.0081361	-2.05	METTL17	Methyltransferase like 17
**222150_s_at**	0.0457413	-2.05	GSAP	Gamma-secretase activating protein
**213410_at**	0.0467236	-2.05	EDRF1	Erythroid differentiation regulatory factor 1
**213883_s_at**	0.0003518	-2.03	TM2D1	TM2 domain containing 1
**228334_x_at**	0.0044389	-2.03	CEP44	Centrosomal protein 44
**203569_s_at**	0.0150214	-2.03	OFD1	OFD1, centriole and centriolar satellite protein
**229028_s_at**	0.0155001	-2.03	ARL17B///ARL17A	ADP ribosylation factor like gtpase 17B///ADP ribosylation factor like gtpase 17A
**215009_s_at**	0.0246258	-2.03	THAP9-AS1	THAP9 antisense RNA 1
**240246_at**	0.0005297	-2.02	FRG1JP	FSHD region gene 1 family member J, pseudogene
**235409_at**	0.0102798	-2.02	MGA	MGA, MAX dimerization protein
**220044_x_at**	0.0194756	-2.02	LUC7L3	LUC7 like 3 pre-mrna splicing factor

EXCLI Journal 2021;20:126-141– ISSN 1611-2156
Received: December 08, 2020, accepted: January 11, 2021, published: January 18, 2021

**236562_at**	0.0194767	-2.02	ZNF439	Zinc finger protein 439
**220609_at**	0.0001946	-2.01	LOC202181	SUMO interacting motifs containing 1 pseudogene
**213659_at**	0.0154313	-2.01	ZNF75D	Zinc finger protein 75D
**225885_at**	0.0327734	-2.01	EEA1	Early endosome antigen 1
**222490_at**	0.0292631	-2.01	LOC101060521///POLR3E	DNA-directed RNA polymerase III subunit RPC5///RNA polymerase III subunit E
**1564310_a_at**	0.0023615	-2	PARP15	Poly(ADP-ribose) polymerase family member 15
**210425_x_at**	0.0374035	-2	LOC101930583///GOLGA8B///GOLGA8A	Uncharacterized LOC101930583///golgin A8 family member B///golgin A8 family member A
**218918_at**	0.0237225	-2	MAN1C1	Mannosidase alpha class 1C member 1
**227082_at**	0.0159781	-2	ZBTB20	Zinc finger and BTB domain containing 20
**230083_at**	0.0246846	-2	USP53	Ubiquitin specific peptidase 53
**201464_x_at**	0.0008752	-1.99	JUN	Jun proto-oncogene, AP-1 transcription factor subunit
**202778_s_at**	0.0304111	-1.99	ZMYM2	Zinc finger MYM-type containing 2
**215743_at**	0.0110602	-1.99	NMT2	N-myristoyltransferase 2
**218577_at**	0.0131027	-1.99	LRRC40	Leucine rich repeat containing 40
**229437_at**	0.0142564	-1.99	MIR155///MIR155HG	Microrna 155///MIR155 host gene
**238722_x_at**	0.0067525	-1.99	NAPEPLD	N-acyl phosphatidylethanolamine phospholipase D
**1568983_a_at**	0.0002747	-1.98	GABPB1-AS1	GABPB1 antisense RNA 1
**201221_s_at**	0.0339511	-1.98	SNRNP70	Small nuclear ribonucleoprotein U1 subunit 70
**219822_at**	0.0453299	-1.98	MTRF1	Mitochondrial translational release factor 1
**225740_x_at**	0.0147572	-1.98	MDM4	MDM4, p53 regulator
**227350_at**	0.0015594	-1.98	HELLS	Helicase, lymphoid-specific
**242289_at**	0.0476639	-1.98	MRPL42	Mitochondrial ribosomal protein L42
**223716_s_at**	0.023834	-1.97	ZRANB2	Zinc finger RANBP2-type containing 2
**228455_at**	0.0248626	-1.97	RBM15	RNA binding motif protein 15
**236816_at**	0.0383656	-1.97	NAA25	N(alpha)-acetyltransferase 25, natb auxiliary subunit
**224367_at**	0.0342343	-1.96	BEX2	Brain expressed X-linked 2
**226753_at**	0.0298615	-1.96	FAM76B	Family with sequence similarity 76 member B
**227485_at**	0.0188514	-1.96	INTS6L	Integrator complex subunit 6 like
**244786_at**	0.0160929	-1.96	SCARNA13///SNHG10	Small Cajal body-specific RNA 13///small nucleolar RNA host gene 10

**1553292_s_at**	0.0112387	-1.95	SGK494	Uncharacterized serine/threonine-protein kinase sgk494
**1569129_s_at**	0.0416365	-1.95	C3orf38	Chromosome 3 open reading frame 38
**223117_s_at**	0.0157017	-1.95	USP47	Ubiquitin specific peptidase 47
**226626_at**	0.0090201	-1.95	THOC2	THO complex 2
**226670_s_at**	0.0033003	-1.95	PABPC1L	Poly(A) binding protein cytoplasmic 1 like
**233093_s_at**	0.0023762	-1.95	BIRC6	Baculoviral IAP repeat containing 6
**233251_at**	0.0047224	-1.95	STRBP	Spermatid perinuclear RNA binding protein
**212274_at**	0.0181556	-1.94	LPIN1	Lipin 1
**214092_x_at**	0.0167041	-1.94	SUGP2	SURP and G-patch domain containing 2
**223528_s_at**	0.0221965	-1.94	METTL17	Methyltransferase like 17
**228702_at**	0.0037949	-1.94	LINC-PINT	Long intergenic non-protein coding RNA, p53 induced transcript
**201448_at**	0.0250574	-1.93	TIA1	TIA1 cytotoxic granule-associated RNA binding protein
**204299_at**	0.0059306	-1.93	SRSF10	Serine and arginine rich splicing factor 10
**205250_s_at**	0.0082577	-1.93	CEP290	Centrosomal protein 290
**209654_at**	0.0163212	-1.93	ICE1	Interactor of little elongation complex ELL subunit 1
**218128_at**	0.0007889	-1.93	NFYB	Nuclear transcription factor Y subunit beta
**228238_at**	0.0096033	-1.93	SNORD77///SNORD76///SNORD74///GAS5///SNORD44///SNORD47///SNORD80///SNORD79///SNORD81	Small nucleolar RNA, C/D box 77///small nucleolar RNA, C/D box 76///small nucleolar RNA, C/D box 74///growth arrest specific 5 (non-protein coding)///small nucleolar RNA, C/D box 44///small nucleolar RNA, C/D box 47///small nucleolar RNA, C/D box 80///small nucleolar RNA, C/D box 79///small nucleolar RNA, C/D box 81
**228941_at**	0.0125394	-1.93	ALG10B///ALG10	ALG10B, alpha-1,2-glucosyltransferase///ALG10, alpha-1,2-glucosyltransferase
**231775_at**	0.0376058	-1.93	TNFRSF10A	TNF receptor superfamily member 10a
**232001_at**	0.0156156	-1.93	PRKCQ-AS1	PRKCQ antisense RNA 1
**235347_at**	0.0141691	-1.93	LRCH3	Leucine rich repeats and calponin homology domain containing 3
**209007_s_at**	0.0176522	-1.92	RSRP1	Arginine and serine rich protein 1
**226327_at**	0.0128735	-1.92	ZNF507	Zinc finger protein 507
**236079_at**	0.0258917	-1.92	LOC202025	Uncharacterized LOC202025
**238121_at**	0.0290318	-1.92	GK5	Glycerol kinase 5 (putative)
**238825_at**	0.0275503	-1.92	ACRC	Acidic repeat containing

EXCLI Journal 2021;20:126-141– ISSN 1611-2156
Received: December 08, 2020, accepted: January 11, 2021, published: January 18, 2021

**241863_x_at**	0.0441199	-1.92	TTC14	Tetratricopeptide repeat domain 14
**244462_at**	0.0184112	-1.92	ZNF224	Zinc finger protein 224
**213906_at**	0.046056	-1.91	MYBL1	MYB proto-oncogene like 1
**215175_at**	0.0336336	-1.91	PCNX1	Pecanex homolog 1 (Drosophila)
**220046_s_at**	0.0295244	-1.91	CCNL1	Cyclin L1
**221645_s_at**	0.0327819	-1.91	ZNF83	Zinc finger protein 83
**225819_at**	0.0177266	-1.91	TBRG1	Transforming growth factor beta regulator 1
**1554608_at**	0.0281137	-1.9	TGOLN2	Trans-golgi network protein 2
**203387_s_at**	0.0484219	-1.9	TBC1D4	TBC1 domain family member 4
**212779_at**	0.0252999	-1.9	KIAA1109	Kiaa1109
**227701_at**	0.033691	-1.9	CCDC186	Coiled-coil domain containing 186
**228592_at**	0.0077645	-1.9	MS4A1	Membrane spanning 4-domains A1
**1555890_at**	0.0022466	-1.89	LOC101928605///OR2A1-AS1///OR2A9P///OR2A20P	Uncharacterized LOC101928605///OR2A1 antisense RNA 1///olfactory receptor family 2 subfamily A member 9 pseudo-gene///olfactory receptor family 2 subfamily A member 20 pseudo-gene
**209374_s_at**	0.0053225	-1.89	IGHM	Immunoglobulin heavy constant mu
**213670_x_at**	0.0298824	-1.89	NSUN5P1	NOP2/Sun RNA methyltransferase family member 5 pseudogene 1
**213817_at**	0.014291	-1.89	IRAK3	Interleukin 1 receptor associated kinase 3
**221558_s_at**	0.03047	-1.89	LEF1	Lymphoid enhancer binding factor 1
**228905_at**	0.0459359	-1.89	PCM1	Pericentriolar material 1
**232338_at**	0.0128272	-1.89	ZNF431	Zinc finger protein 431
**60815_at**	0.0159347	-1.89	POLR2J4	RNA polymerase II subunit J4, pseudogene
**202979_s_at**	0.0424835	-1.88	CREBZF	CREB/ATF bzip transcription factor
**209259_s_at**	0.0329294	-1.88	SMC3	Structural maintenance of chromo-somes 3
**212319_at**	0.0028857	-1.88	SGSM2	Small G protein signaling modula-tor 2
**214709_s_at**	0.0249089	-1.88	KTN1	Kinectin 1
**227802_at**	0.0175425	-1.88	RUFY3	RUN and FYVE domain containing 3
**227987_at**	0.030243	-1.88	VPS13A	Vacuolar protein sorting 13 homo-log A
**203358_s_at**	0.0176361	-1.87	EZH2	Enhancer of zeste 2 polycomb re-pressive complex 2 subunit
**209377_s_at**	0.0140688	-1.87	HMGN3	High mobility group nucleosomal binding domain 3
**211386_at**	0.0385374	-1.87	MGC12488	Uncharacterized protein MGC12488
**213645_at**	0.0055428	-1.87	ENOSF1	Enolase superfamily member 1

EXCLI Journal 2021;20:126-141– ISSN 1611-2156
Received: December 08, 2020, accepted: January 11, 2021, published: January 18, 2021

**228426_at**	0.04134	-1.87	CLEC2D	C-type lectin domain family 2 member D
**235020_at**	0.0232775	-1.87	TAF4B	TATA-box binding protein associated factor 4b
**240573_at**	0.0005811	-1.87	LOC374443	C-type lectin domain family 2 member D pseudogene
**1569482_at**	0.0414929	-1.86	TNRC6C	Trinucleotide repeat containing 6C
**213742_at**	0.0361644	-1.86	SRSF11	Serine and arginine rich splicing factor 11
**219363_s_at**	0.0413208	-1.86	MTERF3	Mitochondrial transcription termination factor 3
**225045_at**	0.0231157	-1.86	CCDC88A	Coiled-coil domain containing 88A
**228245_s_at**	0.0251847	-1.86	LOC100509445///LOC728715///OVOS///OVOS2	Uncharacterized LOC100509445///ovostatin homolog 2///ovostatin///ovostatin 2
**229317_at**	0.0399333	-1.86	KPNA5	Karyopherin subunit alpha 5
**212834_at**	0.003919	-1.85	DDX52	DEAD-box helicase 52
**213018_at**	0.0463896	-1.85	GATAD1	GATA zinc finger domain containing 1
**218827_s_at**	0.0057513	-1.85	CEP192	Centrosomal protein 192
**1559097_at**	0.0035459	-1.84	LINC01550	Long intergenic non-protein coding RNA 1550
**203566_s_at**	0.0326278	-1.84	AGL	Amylo-alpha-1, 6-glucosidase, 4-alpha-glucanotransferase
**212754_s_at**	0.032774	-1.84	MON2	MON2 homolog, regulator of endosome-to-Golgi trafficking
**218249_at**	0.0292881	-1.84	ZDHHC6	Zinc finger DHHC-type containing 6
**221229_s_at**	0.0063235	-1.84	TRMT61B	Trna methyltransferase 61B
**222369_at**	0.0066827	-1.84	NAA40	N(alpha)-acetyltransferase 40, natd catalytic subunit
**225421_at**	0.020936	-1.84	PM20D2	Peptidase M20 domain containing 2
**235482_at**	0.0390991	-1.84	PCBP1-AS1	PCBP1 antisense RNA 1
**1553349_at**	0.009773	-1.83	ARID2	AT-rich interaction domain 2
**1554251_at**	0.0262441	-1.83	HP1BP3	Heterochromatin protein 1 binding protein 3
**1557293_at**	0.0191697	-1.83	LINC00969	Long intergenic non-protein coding RNA 969
**201088_at**	0.0114905	-1.83	KPNA2	Karyopherin subunit alpha 2
**206965_at**	0.0255196	-1.83	KLF12	Kruppel like factor 12
**209430_at**	0.0158317	-1.83	BTAF1	B-TFIID TATA-box binding protein associated factor 1
**222140_s_at**	0.0225144	-1.83	GPR89A///GPR89B	G protein-coupled receptor 89A///G protein-coupled receptor 89B
**244189_at**	0.0273243	-1.83	TTC28-AS1	TTC28 antisense RNA 1

EXCLI Journal 2021;20:126-141– ISSN 1611-2156
Received: December 08, 2020, accepted: January 11, 2021, published: January 18, 2021

**213070_at**	0.0146626	-1.82	PIK3C2A	Phosphatidylinositol-4-phosphate 3-kinase catalytic subunit type 2 alpha
**242539_at**	0.0395997	-1.82	DIS3L2	DIS3 like 3'-5' exoribonuclease 2
**209995_s_at**	0.009895	-1.81	TCL1A	T-cell leukemia/lymphoma 1A
**215599_at**	0.0316804	-1.81	GUSBP9///GUSBP3	Glucuronidase, beta pseudogene 9///glucuronidase, beta pseudogene 3
**218532_s_at**	0.027385	-1.81	FAM134B	Family with sequence similarity 134 member B
**227520_at**	0.0058663	-1.81	TXLNG	Taxilin gamma
**227982_at**	0.0055189	-1.81	SEPSECS	Sep (O-phosphoserine) trna:Sec (selenocysteine) trna synthase
**228046_at**	0.0251893	-1.81	ZNF827	Zinc finger protein 827
**229202_at**	0.0281351	-1.81	PCNX2	Pecanex homolog 2 (Drosophila)
**229891_x_at**	0.0225104	-1.81	GPALPP1	GPALPP motifs containing 1
**233893_s_at**	0.0225226	-1.81	UVSSA	UV stimulated scaffold protein A
**241751_at**	0.0278735	-1.81	OFD1	OFD1, centriole and centriolar satellite protein
**1554067_at**	0.0208307	-1.8	C12orf66	Chromosome 12 open reading frame 66
**225116_at**	0.0446813	-1.8	HIPK2	Homeodomain interacting protein kinase 2
**226528_at**	0.013962	-1.8	MTX3	Metaxin 3
**227040_at**	0.0184393	-1.8	NHLRC3	NHL repeat containing 3
**235230_at**	0.0062031	-1.8	PLCXD2	Phosphatidylinositol specific phospholipase C X domain containing 2
**238593_at**	0.0299254	-1.8	C11orf80	Chromosome 11 open reading frame 80
**1557706_at**	0.0362558	-1.79	ZHX2	Zinc fingers and homeoboxes 2
**212522_at**	0.0134507	-1.79	PDE8A	Phosphodiesterase 8A
**221648_s_at**	0.0050966	-1.79	AGMAT	Agmatinase
**223245_at**	0.0037722	-1.79	STRBP	Spermatid perinuclear RNA binding protein
**225077_at**	0.0435844	-1.79	CHD2	Chromodomain helicase DNA binding protein 2
**228045_at**	0.0159108	-1.79	SUGT1	SGT1 homolog, MIS12 kinetochore complex assembly cochaperone
**228122_at**	0.0074818	-1.79	CCDC66	Coiled-coil domain containing 66
**229253_at**	0.0367939	-1.79	THEM4	Thioesterase superfamily member 4
**231940_at**	0.0202944	-1.79	ZNF529	Zinc finger protein 529
**201613_s_at**	0.0451804	-1.78	AP1G2	Adaptor related protein complex 1 gamma 2 subunit
**208691_at**	0.0069604	-1.78	TFRC	Transferrin receptor
**224875_at**	0.0464164	-1.78	C5orf24	Chromosome 5 open reading frame 24
**231864_at**	0.048889	-1.78	ZNF33A	Zinc finger protein 33A
**233678_at**	0.040581	-1.78	TCF12	Transcription factor 12

EXCLI Journal 2021;20:126-141– ISSN 1611-2156
Received: December 08, 2020, accepted: January 11, 2021, published: January 18, 2021

**212871_at**	0.0007336	-1.77	MAPKAPK5	Mitogen-activated protein kinase-activated protein kinase 5
**215338_s_at**	0.0246042	-1.77	NKTR	Natural killer cell triggering receptor
**223685_s_at**	0.0021901	-1.77	LOC101928524///PRPF18	Uncharacterized LOC101928524///pre-mrna processing factor 18
**226352_at**	0.0098042	-1.77	JMY	Junction mediating and regulatory protein, p53 cofactor
**229231_at**	0.0054438	-1.77	LRRC37B	Leucine rich repeat containing 37B
**229513_at**	0.0090868	-1.77	STRBP	Spermatid perinuclear RNA binding protein
**1553719_s_at**	0.0022856	-1.76	ZNF548	Zinc finger protein 548
**205006_s_at**	0.0204547	-1.76	NMT2	N-myristoyltransferase 2
**206583_at**	0.0181615	-1.76	KRBOX4	KRAB box domain containing 4
**218920_at**	0.033772	-1.76	FAM193B	Family with sequence similarity 193 member B
**222687_s_at**	0.0016006	-1.76	ACER3	Alkaline ceramidase 3
**226765_at**	0.0257949	-1.76	SPTBN1	Spectrin beta, non-erythrocytic 1
**232165_at**	0.0022125	-1.76	EPPK1	Epiplakin 1
**235509_at**	0.0039424	-1.76	LOC100506538///NDUFAF6	Uncharacterized LOC100506538///NADH:ubiquinone oxidoreductase complex assembly factor 6
**238365_s_at**	0.0107305	-1.76	C1orf228	Chromosome 1 open reading frame 228
**1558996_at**	0.046528	-1.75	FOXP1	Forkhead box P1
**205047_s_at**	0.0398059	-1.75	ASNS	Asparagine synthetase (glutamine-hydrolyzing)
**219029_at**	0.0021076	-1.75	TMEM267	Transmembrane protein 267
**220553_s_at**	0.0465014	-1.75	PRPF39	Pre-mrna processing factor 39
**242292_at**	0.0159563	-1.75	FAM226B///FAM226A	Family with sequence similarity 226 member B (non-protein coding)///family with sequence similarity 226 member A (non-protein coding)
**242549_at**	0.0230906	-1.75	PRKD3	Protein kinase D3
**242560_at**	0.0199324	-1.75	FANCD2	Fanconi anemia complementation group D2
**1558692_at**	0.0016144	-1.74	GLMP	Glycosylated lysosomal membrane protein
**209048_s_at**	0.0200091	-1.74	ZMYND8	Zinc finger MYND-type containing 8
**212721_at**	0.0192522	-1.74	SREK1	Splicing regulatory glutamic acid and lysine rich protein 1
**213097_s_at**	0.0482837	-1.74	DNAJC2	Dnaj heat shock protein family (Hsp40) member C2
**214061_at**	0.0277041	-1.74	TBC1D31	TBC1 domain family member 31
**214323_s_at**	0.0282401	-1.74	UPF3A	UPF3 regulator of nonsense transcripts homolog A (yeast)
**218490_s_at**	0.0289144	-1.74	ZNF302	Zinc finger protein 302

EXCLI Journal 2021;20:126-141– ISSN 1611-2156
Received: December 08, 2020, accepted: January 11, 2021, published: January 18, 2021

**218932_at**	0.0054889	-1.74	ZNHIT6	Zinc finger HIT-type containing 6
**219037_at**	0.0049319	-1.74	RRP15	Ribosomal RNA processing 15 homolog
**222014_x_at**	0.0287644	-1.74	MTO1	Mitochondrial trna translation optimization 1
**226433_at**	0.0159616	-1.74	RNF157	Ring finger protein 157
**228826_at**	0.0173837	-1.74	TSPOAP1-AS1	TSPOAP1 antisense RNA 1
**229029_at**	0.0331103	-1.74	CAMK4	Calcium/calmodulin dependent protein kinase IV
**229844_at**	0.0028275	-1.74	FOXP1	Forkhead box P1
**238653_at**	0.0440166	-1.74	LRIG2	Leucine rich repeats and immunoglobulin like domains 2
**32541_at**	0.0221909	-1.74	PPP3CC	Protein phosphatase 3 catalytic subunit gamma
**39318_at**	0.0080637	-1.74	TCL1A	T-cell leukemia/lymphoma 1A
**202104_s_at**	0.0285244	-1.73	LOC101930112///SPG7	Uncharacterized LOC101930112///SPG7, paraplegin matrix AAA peptidase subunit
**206055_s_at**	0.0453331	-1.73	SNRPA1	Small nuclear ribonucleoprotein polypeptide A'
**214221_at**	0.0046739	-1.73	ALMS1	ALMS1, centrosome and basal body associated protein
**214791_at**	0.0106181	-1.73	SP140L	SP140 nuclear body protein like
**219700_at**	0.0499748	-1.73	PLXDC1	Plexin domain containing 1
**235151_at**	0.0420373	-1.73	LOC283357	Uncharacterized LOC283357
**235982_at**	0.0040044	-1.73	FCRL1	Fc receptor like 1
**1563629_a_at**	0.0145401	-1.72	ERVK13-1	Endogenous retrovirus group K13 member 1
**205518_s_at**	0.0144968	-1.72	CMAHP	Cytidine monophospho-N-acetylneuraminic acid hydroxylase, pseudogene
**209315_at**	0.004492	-1.72	HBS1L	HBS1 like translational gtpase
**212945_s_at**	0.0195064	-1.72	MGA	MGA, MAX dimerization protein
**213046_at**	0.0445115	-1.72	PABPN1	Poly(A) binding protein nuclear 1
**214132_at**	0.0355315	-1.72	ATP5C1	ATP synthase, H+ transporting, mitochondrial F1 complex, gamma polypeptide 1
**214684_at**	0.0410429	-1.72	MEF2A	Myocyte enhancer factor 2A
**216908_x_at**	0.0206033	-1.72	RRN3P1	RRN3 homolog, RNA polymerase I transcription factor pseudogene 1
**219754_at**	0.0090675	-1.72	RBM41	RNA binding motif protein 41
**223444_at**	0.0051961	-1.72	SENP7	SUMO1/sentrin specific peptidase 7
**224873_s_at**	0.0178659	-1.72	MRPS25	Mitochondrial ribosomal protein S25
**226591_at**	0.0188041	-1.72	PWAR6	Prader Willi/Angelman region RNA 6
**229007_at**	0.0104519	-1.72	LOC283788	FSHD region gene 1 pseudogene

EXCLI Journal 2021;20:126-141– ISSN 1611-2156
Received: December 08, 2020, accepted: January 11, 2021, published: January 18, 2021

**232000_at**	0.005977	-1.72	TTC39B	Tetratricopeptide repeat domain 39B
**233849_s_at**	0.0067096	-1.72	ARHGAP5	Rho gtpase activating protein 5
**244872_at**	0.0178699	-1.72	RBBP4	RB binding protein 4, chromatin re-modeling factor
**201837_s_at**	0.0023008	-1.71	SUPT7L	SPT7-like STAGA complex gamma subunit
**202066_at**	0.0162302	-1.71	PPFIA1	PTPRF interacting protein alpha 1
**203124_s_at**	0.0323709	-1.71	SLC11A2	Solute carrier family 11 member 2
**213043_s_at**	0.018376	-1.71	MIR6884///MED24	Microrna 6884///mediator complex subunit 24
**213899_at**	0.009801	-1.71	METAP2	Methionyl aminopeptidase 2
**214100_x_at**	0.0230621	-1.71	NSUN5P1	NOP2/Sun RNA methyltransferase family member 5 pseudogene 1
**219078_at**	0.0197639	-1.71	GPATCH2	G-patch domain containing 2
**225521_at**	0.0116788	-1.71	ANAPC7	Anaphase promoting complex sub-unit 7
**227772_at**	0.0114538	-1.71	LATS1	Large tumor suppressor kinase 1
**228652_at**	0.00518	-1.71	ZNF776	Zinc finger protein 776
**232633_at**	0.0241966	-1.71	XRCC5	X-ray repair cross complementing 5
**234192_s_at**	0.0172476	-1.71	GKAP1	G kinase anchoring protein 1
**239014_at**	0.0289191	-1.71	CCAR1	Cell division cycle and apoptosis regulator 1
**205372_at**	0.003415	-1.7	PLAG1	PLAG1 zinc finger
**209815_at**	0.0117149	-1.7	PTCH1	Patched 1
**212709_at**	0.0317373	-1.7	NUP160	Nucleoporin 160
**213353_at**	0.0434521	-1.7	ABCA5	ATP binding cassette subfamily A member 5
**215211_at**	0.0264216	-1.7	RRN3P1	RRN3 homolog, RNA polymerase I transcription factor pseudogene 1
**219359_at**	0.0181163	-1.7	PGGHG	Protein-glucosylgalactosylhydrox-ylysine glucosidase
**224705_s_at**	0.0222402	-1.7	TNRC6A	Trinucleotide repeat containing 6A
**226665_at**	0.0264478	-1.7	AHSA2	AHA1, activator of heat shock 90kda protein atpase homolog 2 (yeast)
**228023_x_at**	0.0393945	-1.7	ACTG1P4///RNPC3///AMY2B	Actin gamma 1 pseudogene 4///RNA binding region (RNP1, RRM) containing 3///amylase, al-pha 2B (pancreatic)
**203386_at**	0.0312421	-1.69	TBC1D4	TBC1 domain family member 4
**210512_s_at**	0.0011307	-1.69	VEGFA	Vascular endothelial growth factor A
**218538_s_at**	0.0109083	-1.69	MRS2	MRS2, magnesium transporter
**221825_at**	0.0410095	-1.69	ANGEL2	Angel homolog 2
**242239_at**	0.0364621	-1.69	NSUN6	NOP2/Sun RNA methyltransferase family member 6
**211704_s_at**	0.0013335	-1.68	SPIN2B///SPIN2A	Spindlin family member 2B///spin-dlin family member 2A

EXCLI Journal 2021;20:126-141– ISSN 1611-2156
Received: December 08, 2020, accepted: January 11, 2021, published: January 18, 2021

**211953_s_at**	0.0399552	-1.68	IPO5	Importin 5
**212885_at**	0.024136	-1.68	MPHOSPH 10	M-phase phosphoprotein 10
**214937_x_at**	0.0191009	-1.68	PCM1	Pericentriolar material 1
**218701_at**	0.0062274	-1.68	LACTB2	Lactamase beta 2
**226869_at**	0.0488186	-1.68	MEGF6	Multiple EGF like domains 6
**1558292_s_at**	0.0056591	-1.67	PIGW	Phosphatidylinositol glycan anchor biosynthesis class W
**201948_at**	0.0113709	-1.67	GNL2	G protein nucleolar 2
**217805_at**	0.0307214	-1.67	ILF3	Interleukin enhancer binding factor 3
**222273_at**	0.020605	-1.67	PAPOLG	Poly(A) polymerase gamma
**224610_at**	0.0354472	-1.67	SNHG1///SNORD22///SNORD25///SNORD26///SNORD27///SNORD28///SNORD30///SNORD31///SNORD29	Small nucleolar RNA host gene 1///small nucleolar RNA, C/D box 22///small nucleolar RNA, C/D box 25///small nucleolar RNA, C/D box 26///small nucleolar RNA, C/D box 27///small nucleolar RNA, C/D box 28///small nucleolar RNA, C/D box 30///small nucleolar RNA, C/D box 31///small nucleolar RNA, C/D box 29
**225176_at**	0.0334785	-1.67	LNPEP	Leucyl and cystinyl aminopeptidase
**226558_at**	0.0271626	-1.67	TEKT4P2///MAFIP///LOC389834	Tektin 4 pseudogene 2///MAFF interacting protein (pseudogene)///ankyrin repeat domain 57 pseudogene
**227766_at**	0.0275668	-1.67	LIG4	DNA ligase 4
**230187_s_at**	0.0034802	-1.67	LOC102724 851	Uncharacterized LOC102724851
**1569484_s_at**	0.0278356	-1.66	MDN1	Midasin AAA atpase 1
**208070_s_at**	0.0039472	-1.66	REV3L	REV3 like, DNA directed polymerase zeta catalytic subunit
**212058_at**	0.0376412	-1.66	U2SURP	U2 snrnp associated SURP domain containing
**212493_s_at**	0.0330084	-1.66	SETD2	SET domain containing 2
**218228_s_at**	0.013302	-1.66	TNKS2	Tankyrase 2
**225035_x_at**	0.0400391	-1.66	LOC102723897///MIR6859-1///MIR6859-2///LOC101930154///LOC100288778///WASH1///WASH7P///WASH2P///WASH3P	WAS protein family homolog 2-like///microrna 6859-1///microrna 6859-2///WAS protein family homolog 6-like///WAS protein family homolog 1 pseudogene///WAS protein family homolog 1///WAS protein family homolog 7 pseudogene///WAS protein family homolog 2 pseudogene///WAS protein family homolog 3 pseudogene
**203049_s_at**	0.0132849	-1.65	TTC37	Tetratricopeptide repeat domain 37

EXCLI Journal 2021;20:126-141– ISSN 1611-2156
Received: December 08, 2020, accepted: January 11, 2021, published: January 18, 2021

**204190_at**	0.0135324	-1.65	USPL1	Ubiquitin specific peptidase like 1
**204407_at**	0.0026997	-1.65	TTF2	Transcription termination factor 2
**206715_at**	0.0308594	-1.65	TFEC	Transcription factor EC
**220577_at**	0.001347	-1.65	GVINP1	Gtpase, very large interferon inducible pseudogene 1
**221538_s_at**	0.0234325	-1.65	PLXNA1	Plexin A1
**221782_at**	0.0181015	-1.65	DNAJC10	Dnaj heat shock protein family (Hsp40) member C10
**224737_x_at**	0.022431	-1.65	CCAR1	Cell division cycle and apoptosis regulator 1
**228423_at**	0.0287067	-1.65	MAP9	Microtubule associated protein 9
**228697_at**	0.0174697	-1.65	HINT3	Histidine triad nucleotide binding protein 3
**232055_at**	0.0056368	-1.65	SFXN1	Sideroflexin 1
**238890_at**	0.009751	-1.65	BRWD1	Bromodomain and WD repeat domain containing 1
**208051_s_at**	0.0029759	-1.64	PAIP1	Poly(A) binding protein interacting protein 1
**213064_at**	0.0409777	-1.64	ZC3H14	Zinc finger CCCH-type containing 14
**213804_at**	0.0266961	-1.64	INPP5B	Inositol polyphosphate-5-phosphatase B
**220148_at**	0.001596	-1.64	ALDH8A1	Aldehyde dehydrogenase 8 family member A1
**222047_s_at**	0.0421327	-1.64	SRRT	Serrate, RNA effector molecule
**223096_at**	0.0056379	-1.64	NOP58	NOP58 ribonucleoprotein
**227489_at**	0.0063791	-1.64	SMURF2	SMAD specific E3 ubiquitin protein ligase 2
**228191_at**	0.0279736	-1.64	FLVCR1	Feline leukemia virus subgroup C cellular receptor 1
**228370_at**	0.0283758	-1.64	LOC101930404///SNORD116-28///SNORD115-26///SNORD115-13///SNORD115-7///SNORD116-22///SNORD116-4///PWARSN///SNORD107///IPW	Uncharacterized LOC101930404///small nucleolar RNA, C/D box 116-28///small nucleolar RNA, C/D box 115-26///small nucleolar RNA, C/D box 115-13///small nucleolar RNA, C/D box 115-7///small nucleolar RNA, C/D box 116-22///small nucleolar RNA, C/D box 116-4///Prader Willi/Angelman region RNA, SNRPN neighbor///small nucleolar RNA, C/D box 107///imprinted in Prader-Willi syndrome (non-protein coding)
**229220_x_at**	0.0292803	-1.64	NOM1	Nucleolar protein with MIF4G domain 1
**229614_at**	0.010251	-1.64	ZNF320	Zinc finger protein 320
**235154_at**	0.0036797	-1.64	TAF3	TATA-box binding protein associated factor 3

**239376_at**	0.0200438	-1.64	DCLRE1C	DNA cross-link repair 1C
**227375_at**	0.0085566	-1.63	ANKRD13C	Ankyrin repeat domain 13C
**229431_at**	0.0273285	-1.63	RFXAP	Regulatory factor X associated protein
**230918_at**	0.0164633	-1.63	GALK2	Galactokinase 2
**201450_s_at**	0.0092993	-1.62	TIA1	TIA1 cytotoxic granule-associated RNA binding protein
**201821_s_at**	0.0349228	-1.62	TIMM17A	Translocase of inner mitochondrial membrane 17 homolog A (yeast)
**209688_s_at**	0.0192969	-1.62	CCDC93	Coiled-coil domain containing 93
**214833_at**	0.0338254	-1.62	TMEM63A	Transmembrane protein 63A
**214917_at**	0.0261899	-1.62	PRKAA1	Protein kinase AMP-activated catalytic subunit alpha 1
**226800_at**	0.003291	-1.62	EFCAB7	EF-hand calcium binding domain 7
**230563_at**	0.0185402	-1.62	RASGEF1A	Rasgef domain family member 1A
**231111_at**	0.0361222	-1.62	SCYL3	SCY1 like pseudokinase 3
**235125_x_at**	0.0175126	-1.62	MIGA1	Mitoguardin 1
**236386_at**	0.0111618	-1.62	SUZ12P1	SUZ12 polycomb repressive complex 2 subunit pseudogene 1
**242814_at**	0.0017693	-1.62	SERPINB9	Serpin family B member 9
**206828_at**	0.044298	-1.61	TXK	TXK tyrosine kinase
**208896_at**	0.0390198	-1.61	DDX18	DEAD-box helicase 18
**212061_at**	0.0150351	-1.61	U2SURP	U2 snrnp associated SURP domain containing
**219481_at**	0.0297493	-1.61	TTC13	Tetratricopeptide repeat domain 13
**222999_s_at**	0.0149416	-1.61	CCNL2	Cyclin L2
**229153_at**	0.0041564	-1.61	SLC7A6OS	Solute carrier family 7 member 6 opposite strand
**213398_s_at**	0.0271408	-1.6	SDR39U1	Short chain dehydrogenase/reductase family 39U member 1
**224450_s_at**	0.0337606	-1.6	RIOK1	RIO kinase 1
**225504_at**	0.0277124	-1.6	HMBOX1	Homeobox containing 1
**226474_at**	0.0484502	-1.6	NLRC5	NLR family CARD domain containing 5
**228029_at**	0.0495867	-1.6	ZNF721	Zinc finger protein 721
**237759_at**	0.0376763	-1.6	CD48	CD48 molecule
**244663_at**	0.0146766	-1.6	ZNF431	Zinc finger protein 431
**1569369_at**	0.007668	-1.59	ZFYVE28	Zinc finger FYVE-type containing 28
**204193_at**	0.022426	-1.59	CHKB	Choline kinase beta
**209774_x_at**	0.00806	-1.59	CXCL2	C-X-C motif chemokine ligand 2
**211929_at**	0.026018	-1.59	HNRNPA3	Heterogeneous nuclear ribonucleoprotein A3
**212289_at**	0.0202896	-1.59	ANKRD12	Ankyrin repeat domain 12
**212366_at**	0.0028628	-1.59	ZNF292	Zinc finger protein 292
**214785_at**	0.0316191	-1.59	VPS13A	Vacuolar protein sorting 13 homolog A

EXCLI Journal 2021;20:126-141– ISSN 1611-2156
Received: December 08, 2020, accepted: January 11, 2021, published: January 18, 2021

**215691_x_at**	0.0345653	-1.59	HSPB11	Heat shock protein family B (small) member 11
**226384_at**	0.0253187	-1.59	PLPP5	Phospholipid phosphatase 5
**226611_s_at**	0.0124161	-1.59	CENPV	Centromere protein V
**227274_at**	0.0093482	-1.59	SYNJ2BP-COX16///SYNJ2BP	SYNJ2BP-COX16 readthrough///synaptojanin 2 binding protein
**230142_s_at**	0.040257	-1.59	CIRBP	Cold inducible RNA binding protein
**231323_at**	0.00412	-1.59	PSMB2	Proteasome subunit beta 2
**232395_x_at**	0.0044946	-1.59	AGBL3	ATP/GTP binding protein like 3
**235469_at**	0.0222868	-1.59	FAM133DP///FAM133B	Family with sequence similarity 133, member A pseudogene///family with sequence similarity 133 member B
**238538_at**	0.0200217	-1.59	ANKRD11	Ankyrin repeat domain 11
**240072_at**	0.0407345	-1.59	ASXL2	Additional sex combs like 2, transcriptional regulator
**241689_at**	0.0068449	-1.59	METTL14	Methyltransferase like 14
**242915_at**	0.001322	-1.59	ZNF682	Zinc finger protein 682
**204076_at**	0.0308256	-1.58	ENTPD4	Ectonucleoside triphosphate diphosphohydrolase 4
**219802_at**	0.0388519	-1.58	PYROXD1	Pyridine nucleotide-disulphide oxidoreductase domain 1
**220917_s_at**	0.0188482	-1.58	WDR19	WD repeat domain 19
**221988_at**	0.0490862	-1.58	SMIM7	Small integral membrane protein 7
**224635_s_at**	0.0090582	-1.58	BIRC6	Baculoviral IAP repeat containing 6
**225988_at**	0.042205	-1.58	HERC4	HECT and RLD domain containing E3 ubiquitin protein ligase 4
**228397_at**	0.0289119	-1.58	TUG1	Taurine up-regulated 1 (non-protein coding)
**228468_at**	0.0009263	-1.58	MASTL	Microtubule associated serine/threonine kinase like
**230177_at**	0.0206854	-1.58	GTF2H2B///GTF2H2	General transcription factor IIH subunit 2B (pseudogene)///general transcription factor IIH subunit 2
**235444_at**	0.0136534	-1.58	FOXP1	Forkhead box P1
**1553749_at**	0.0298793	-1.57	FAM76B	Family with sequence similarity 76 member B
**1557820_at**	0.0008749	-1.57	AFG3L2	AFG3 like matrix AAA peptidase subunit 2
**201711_x_at**	0.0241059	-1.57	RANBP2	RAN binding protein 2
**203970_s_at**	0.0025805	-1.57	PEX3	Peroxisomal biogenesis factor 3
**209902_at**	0.0447385	-1.57	ATR	ATR serine/threonine kinase
**213331_s_at**	0.0308697	-1.57	NEK1	NIMA related kinase 1
**214894_x_at**	0.0330903	-1.57	MACF1	Microtubule-actin crosslinking factor 1
**221802_s_at**	0.0221772	-1.57	SHTN1	Shootin 1
**225698_at**	0.0411317	-1.57	EPB41L4A-AS1	EPB41L4A antisense RNA 1

EXCLI Journal 2021;20:126-141– ISSN 1611-2156
Received: December 08, 2020, accepted: January 11, 2021, published: January 18, 2021

**226631_at**	0.0203941	-1.57	METTL10	Methyltransferase like 10
**228264_at**	0.0236132	-1.57	ACCS	1-aminocyclopropane-1-carboxylate synthase homolog (inactive)
**228529_at**	0.0030903	-1.57	LSM8	LSM8 homolog, U6 small nuclear RNA associated
**231989_s_at**	0.0018129	-1.57	LOC101060604///NPIPA5///SMG1P3///NPIPB5///SMG1P1///LOC613037///NPIPB4///SLC7A5P1///NPIPB3	Putative L-type amino acid transporter 1-like protein IMAA///nuclear pore complex interacting protein family member A5///SMG1P3, nonsense mediated mrna decay associated PI3K related kinase pseudogene 3///nuclear pore complex interacting protein family member B5///SMG1P1, nonsense mediated mrna decay associated PI3K related kinase pseudogene 1///nuclear pore complex interacting protein member///nuclear pore complex interacting protein family member B4///solute carrier family 7 member 5 pseudogene 1///nuclear pore complex interacting protein family member B3
**235902_at**	0.0405212	-1.57	HERC2P7	Hect domain and RLD 2 pseudogene 7
**243495_s_at**	0.0174967	-1.57	ZNF652	Zinc finger protein 652
**209841_s_at**	0.0447956	-1.56	LRRN3	Leucine rich repeat neuronal 3
**212326_at**	0.0282565	-1.56	VPS13D	Vacuolar protein sorting 13 homolog D
**219335_at**	0.0240684	-1.56	ARMCX5	Armadillo repeat containing, X-linked 5
**223231_at**	0.0227728	-1.56	TATDN1	Tatd dnase domain containing 1
**226124_at**	0.031883	-1.56	ZFP90	ZFP90 zinc finger protein
**228160_at**	0.0024407	-1.56	LINC00667	Long intergenic non-protein coding RNA 667
**235007_at**	0.0018638	-1.56	BBS7	Bardet-Biedl syndrome 7
**235730_at**	0.0096278	-1.56	NUTM2B-AS1	NUTM2B antisense RNA 1
**238694_at**	0.0231814	-1.56	DGKE	Diacylglycerol kinase epsilon
**1559496_at**	0.0497133	-1.55	PPA2	Pyrophosphatase (inorganic) 2
**204244_s_at**	0.0228199	-1.55	DBF4	DBF4 zinc finger
**210944_s_at**	0.0298745	-1.55	CAPN3	Calpain 3
**212789_at**	0.0226108	-1.55	NCAPD3	Non-SMC condensin II complex subunit D3
**212999_x_at**	0.0260326	-1.55	LOC101060835///HLA-DQB1	HLA class II histocompatibility antigen, DQ beta 1 chain-like///major histocompatibility complex, class II, DQ beta 1
**218184_at**	0.005939	-1.55	TULP4	Tubby like protein 4
**222765_x_at**	0.0240611	-1.55	ESF1	ESF1 nucleolar pre-rrna processing protein homolog

**226831_at**	0.036527	-1.55	SLC25A46	Solute carrier family 25 member 46
**228287_at**	0.0126395	-1.55	ING5	Inhibitor of growth family member 5
**229926_at**	0.0044264	-1.55	MIR3682	Microrna 3682
**235167_at**	0.0091505	-1.55	LOC100190986	Uncharacterized LOC100190986
**236704_at**	0.0140308	-1.55	LOC101929787	Uncharacterized LOC101929787
**243612_at**	0.0490006	-1.55	NSD1	Nuclear receptor binding SET domain protein 1
**1558620_at**	0.0296649	-1.54	ZNF621	Zinc finger protein 621
**1558711_at**	0.0031777	-1.54	FAM13A-AS1	FAM13A antisense RNA 1
**203625_x_at**	0.0299839	-1.54	SKP2	S-phase kinase-associated protein 2, E3 ubiquitin protein ligase
**203848_at**	0.0234761	-1.54	AKAP8	A-kinase anchoring protein 8
**209627_s_at**	0.026119	-1.54	OSBPL3	Oxysterol binding protein like 3
**212785_s_at**	0.0156007	-1.54	LARP7	La ribonucleoprotein domain family member 7
**214870_x_at**	0.0313423	-1.54	LOC102724993///LOC101930075///NPIPA8///NPIPA7///NPIPA5///NPIPA2///NPIPA3///PKD1P1///NPIPA1	Nuclear pore complex-interacting protein family member A7-like///polycystin-1-like///nuclear pore complex interacting protein family member A8///nuclear pore complex interacting protein family member A7///nuclear pore complex interacting protein family member A5///nuclear pore complex interacting protein family member A2///nuclear pore complex interacting protein family member A3///polycystin 1, transient receptor potential channel interacting pseudogene 1///nuclear pore complex interacting protein family member A1
**222557_at**	0.0144623	-1.54	STMN3	Stathmin 3
**227761_at**	0.0186787	-1.54	MYO5A	Myosin VA
**228577_x_at**	0.0049704	-1.54	ODF2L	Outer dense fiber of sperm tails 2 like
**229025_s_at**	0.0262009	-1.54	IMMP1L	Inner mitochondrial membrane peptidase subunit 1
**230069_at**	0.0144789	-1.54	SFXN1	Sideroflexin 1
**240239_at**	0.0409778	-1.54	ZNF566	Zinc finger protein 566
**242140_at**	0.0179841	-1.54	ERVK3-1///ZNF8	Endogenous retrovirus group K3 member 1///zinc finger protein 8
**1552343_s_at**	0.0407516	-1.53	PDE7A	Phosphodiesterase 7A
**1554287_at**	0.0113716	-1.53	TRIM4	Tripartite motif containing 4
**201230_s_at**	0.0345115	-1.53	ARIH2	Ariadne RBR E3 ubiquitin protein ligase 2
**203431_s_at**	0.0056852	-1.53	ARHGAP32	Rho gtpase activating protein 32

EXCLI Journal 2021;20:126-141– ISSN 1611-2156
Received: December 08, 2020, accepted: January 11, 2021, published: January 18, 2021

**212798_s_at**	0.0195134	-1.53	ANKMY2	Ankyrin repeat and MYND domain containing 2
**217707_x_at**	0.0186929	-1.53	SMARCA2	SWI/SNF related, matrix associated, actin dependent regulator of chromatin, subfamily a, member 2
**228242_at**	0.0232942	-1.53	N4BP2	NEDD4 binding protein 2
**229074_at**	0.0045566	-1.53	EHD4	EH domain containing 4
**230759_at**	0.0474584	-1.53	SNX14	Sorting nexin 14
**232874_at**	0.0411752	-1.53	DOCK9	Dedicator of cytokinesis 9
**233852_at**	0.0023712	-1.53	POLH	DNA polymerase eta
**241793_at**	0.0112677	-1.53	MSS51	MSS51 mitochondrial translational activator
**242818_x_at**	0.0078826	-1.53	YIPF4	Yip1 domain family member 4
**1552347_at**	0.0157486	-1.52	CRYZL1	Crystallin zeta like 1
**1560145_at**	0.0330365	-1.52	MKLN1	Muskelin 1
**202034_x_at**	0.026115	-1.52	RB1CC1	RB1 inducible coiled-coil 1
**210701_at**	0.0072292	-1.52	CFDP1	Craniofacial development protein 1
**215898_at**	0.0040892	-1.52	TTLL5	Tubulin tyrosine ligase like 5
**218397_at**	0.0037645	-1.52	FANCL	Fanconi anemia complementation group L
**219627_at**	0.0263661	-1.52	ZNF767P	Zinc finger family member 767, pseudogene
**226019_at**	0.0441323	-1.52	OMA1	OMA1 zinc metallopeptidase
**226791_at**	0.0364517	-1.52	KIFC2	Kinesin family member C2
**230479_at**	0.0228544	-1.52	EIF3F	Eukaryotic translation initiation factor 3 subunit F
**235590_at**	0.0178126	-1.52	SLF2	SMC5-SMC6 complex localization factor 2
**235603_at**	0.0033487	-1.52	HNRNPU	Heterogeneous nuclear ribonucleoprotein U
**236075_s_at**	0.0094326	-1.52	LOC101928000	Uncharacterized LOC101928000
**1565743_at**	0.0205972	-1.51	N4BP2L2	NEDD4 binding protein 2 like 2
**201680_x_at**	0.0258381	-1.51	SRRT	Serrate, RNA effector molecule
**203607_at**	0.0139868	-1.51	INPP5F	Inositol polyphosphate-5-phosphatase F
**203801_at**	0.0043882	-1.51	MRPS14	Mitochondrial ribosomal protein S14
**207655_s_at**	0.0087505	-1.51	BLNK	B-cell linker
**209271_at**	0.0334377	-1.51	BPTF	Bromodomain PHD finger transcription factor
**210054_at**	0.0231126	-1.51	HAUS3	HAUS augmin like complex subunit 3
**212753_at**	0.0265367	-1.51	PCGF3	Polycomb group ring finger 3
**226588_at**	0.0448979	-1.51	CWC22	CWC22 homolog, spliceosome-associated protein
**227001_at**	0.003515	-1.51	NIPAL2	NIPA like domain containing 2
**228324_at**	0.0185244	-1.51	CARNMT1	Carnosine N-methyltransferase 1

EXCLI Journal 2021;20:126-141– ISSN 1611-2156
Received: December 08, 2020, accepted: January 11, 2021, published: January 18, 2021

**228528_at**	0.0071484	-1.51	MIR29C///MIR29B2	Microrna 29c///microrna 29b-2
**228566_at**	0.0022506	-1.51	RPRD1A	Regulation of nuclear pre-mrna domain containing 1A
**229804_x_at**	0.0247955	-1.51	CBWD7///CBWD6///CBWD3///CBWD5///CBWD2///CBWD1	COBW domain containing 7///COBW domain containing 6///COBW domain containing 3///COBW domain containing 5///COBW domain containing 2///COBW domain containing 1
**235346_at**	0.0227738	-1.51	FUNDC1	FUN14 domain containing 1
**235675_at**	0.0170925	-1.51	DHFR2	Dihydrofolate reductase 2
**238005_s_at**	0.0274078	-1.51	SIN3A	SIN3 transcription regulator family member A
**242273_at**	0.0193154	-1.51	LLPH	LLP homolog, long-term synaptic facilitation
**243363_at**	0.0036823	-1.51	LEF1-AS1	LEF1 antisense RNA 1
**243417_at**	0.0445622	-1.51	ZADH2	Zinc binding alcohol dehydrogenase domain containing 2
**243511_at**	0.0132442	-1.51	HEXA	Hexosaminidase subunit alpha
**201694_s_at**	0.0148691	-1.5	EGR1	Early growth response 1
**203551_s_at**	0.0242726	-1.5	COX11	COX11, cytochrome c oxidase copper chaperone
**204089_x_at**	0.0267695	-1.5	MAP3K4	Mitogen-activated protein kinase kinase kinase 4
**208393_s_at**	0.0238853	-1.5	RAD50	RAD50 double strand break repair protein
**212462_at**	0.040004	-1.5	KAT6B	Lysine acetyltransferase 6B
**213267_at**	0.0019428	-1.5	DOPEY1	Dopey family member 1
**213581_at**	0.0090145	-1.5	PDCD2	Programmed cell death 2
**219220_x_at**	0.0054395	-1.5	MRPS22	Mitochondrial ribosomal protein S22
**223203_at**	0.0014188	-1.5	FAM156B///FAM156A	Family with sequence similarity 156 member B///family with sequence similarity 156 member A
**223301_s_at**	0.0308838	-1.5	CCDC82	Coiled-coil domain containing 82
**224591_at**	0.0206886	-1.5	HP1BP3	Heterochromatin protein 1 binding protein 3
**225188_at**	0.0126782	-1.5	RAPH1	Ras association (ralgds/AF-6) and pleckstrin homology domains 1
**227611_at**	0.0113815	-1.5	TARSL2	Threonyl-trna synthetase like 2
**227622_at**	0.0342044	-1.5	PCF11	PCF11 cleavage and polyadenylation factor subunit
**227810_at**	0.0042302	-1.5	ZNF558	Zinc finger protein 558
**228330_at**	0.0277163	-1.5	ZUFSP	Zinc finger with UFM1 specific peptidase domain
**229705_at**	0.0163885	-1.5	PIK3C3	Phosphatidylinositol 3-kinase catalytic subunit type 3
**230235_at**	0.0293512	-1.5	MCTS1	MCTS1, re-initiation and release factor

EXCLI Journal 2021;20:126-141– ISSN 1611-2156
Received: December 08, 2020, accepted: January 11, 2021, published: January 18, 2021

**230407_at**	0.0257827	-1.5	SBNO1	Strawberry notch homolog 1
**239644_at**	0.0223683	-1.5	ZC3H8	Zinc finger CCCH-type containing 8
**244881_at**	0.013838	-1.5	LMLN	Leishmanolysin like peptidase
**1559094_at**	0.0381847	-1.49	FBXO9	F-box protein 9
**202968_s_at**	0.0272876	-1.49	DYRK2	Dual specificity tyrosine phosphory-lation regulated kinase 2
**204521_at**	0.0181639	-1.49	FAM216A	Family with sequence similarity 216 member A
**205407_at**	0.0314716	-1.49	RECK	Reversion inducing cysteine rich protein with kazal motifs
**209871_s_at**	0.0371714	-1.49	APBA2	Amyloid beta precursor protein binding family A member 2
**212827_at**	0.0080895	-1.49	IGHM	Immunoglobulin heavy constant mu
**218859_s_at**	0.0314432	-1.49	ESF1	ESF1 nucleolar pre-rrna pro-cessing protein homolog
**222610_s_at**	0.0056651	-1.49	S100PBP	S100P binding protein
**226628_at**	0.0161026	-1.49	THOC2	THO complex 2
**230120_s_at**	0.0323992	-1.49	PLGLB1///PLGLB2	Plasminogen-like B1///plasmino-gen-like B2
**233665_x_at**	0.0031693	-1.49	MTO1	Mitochondrial trna translation opti-mization 1
**233869_x_at**	0.0132467	-1.49	DKFZP586B0319	DKFZP586B0319 protein
**236429_at**	0.0222414	-1.49	ZNF83	Zinc finger protein 83
**237189_at**	0.0398655	-1.49	HOXB-AS1	HOXB cluster antisense RNA 1
**239413_at**	0.0200362	-1.49	CEP152	Centrosomal protein 152
**202860_at**	0.0383197	-1.48	DENND4B	DENN domain containing 4B
**206314_at**	0.0082291	-1.48	ZKSCAN7	Zinc finger with KRAB and SCAN domains 7
**207877_s_at**	0.0100126	-1.48	NVL	Nuclear VCP-like
**212414_s_at**	0.0148143	-1.48	GLYR1///SEPT6	Glyoxylate reductase 1 homo-log///septin 6
**212913_at**	0.017154	-1.48	MSH5-SAPCD1///SAPCD1	MSH5-SAPCD1 readthrough (NMD candidate)///suppressor APC do-main containing 1
**213485_s_at**	0.0169674	-1.48	ABCC10	ATP binding cassette subfamily C member 10
**219603_s_at**	0.0037166	-1.48	ZNF226	Zinc finger protein 226
**220178_at**	0.0015428	-1.48	MFSD12	Major facilitator superfamily domain containing 12
**222843_at**	0.0154597	-1.48	FIGNL1	Fidgetin like 1
**223197_s_at**	0.0081106	-1.48	SMAR-CAD1	SWI/SNF-related, matrix-associ-ated actin-dependent regulator of chromatin, subfamily a, containing DEAD/H box 1
**225278_at**	0.0466426	-1.48	PRKAB2	Protein kinase AMP-activated non-catalytic subunit beta 2
**226395_at**	0.0290576	-1.48	HOOK3	Hook microtubule tethering protein 3

EXCLI Journal 2021;20:126-141– ISSN 1611-2156
Received: December 08, 2020, accepted: January 11, 2021, published: January 18, 2021

**226503_at**	0.0320894	-1.48	RIF1	Replication timing regulatory factor 1
**229710_at**	0.0414204	-1.48	ERCC6L2	ERCC excision repair 6 like 2
**235027_at**	0.0025929	-1.48	ZNF736	Zinc finger protein 736
**235107_at**	0.019065	-1.48	PIAS2	Protein inhibitor of activated STAT 2
**235762_at**	0.0180375	-1.48	TAS2R14	Taste 2 receptor member 14
**243042_at**	0.0388841	-1.48	MIGA1	Mitoguardin 1
**1553099_at**	0.0049647	-1.47	TIGD1	Tigger transposable element derived 1
**1553987_at**	0.0243653	-1.47	MAPKAPK5-AS1	MAPKAPK5 antisense RNA 1
**204510_at**	0.0363444	-1.47	CDC7	Cell division cycle 7
**210873_x_at**	0.0068124	-1.47	APOBEC3A_B///APOBEC3A	APOBEC3A and APOBEC3B deletion hybrid///apolipoprotein B mrna editing enzyme catalytic subunit 3A
**212402_at**	0.0363364	-1.47	ZC3H13	Zinc finger CCCH-type containing 13
**212936_at**	0.0157906	-1.47	FAM172A	Family with sequence similarity 172 member A
**213758_at**	0.0048957	-1.47	COX4I1	Cytochrome c oxidase subunit 4I1
**213842_x_at**	0.0280873	-1.47	NSUN5P2///NSUN5P1	NOP2/Sun RNA methyltransferase family member 5 pseudogene 2///NOP2/Sun RNA methyltransferase family member 5 pseudogene 1
**216305_s_at**	0.027107	-1.47	GCFC2	GC-rich sequence DNA-binding factor 2
**222825_at**	0.0156895	-1.47	OTUD6B	OTU domain containing 6B
**224721_at**	0.0258614	-1.47	WDR75	WD repeat domain 75
**225792_at**	0.0356311	-1.47	HOOK1	Hook microtubule tethering protein 1
**226102_at**	0.0089937	-1.47	ZKSCAN8	Zinc finger with KRAB and SCAN domains 8
**228381_at**	0.0062719	-1.47	ATF7IP2	Activating transcription factor 7 interacting protein 2
**238912_x_at**	0.0065062	-1.47	C9orf85	Chromosome 9 open reading frame 85
**241365_at**	0.0173074	-1.47	SATB1	SATB homeobox 1
**243910_x_at**	0.0098181	-1.47	CAND1	Cullin associated and neddylation dissociated 1
**202548_s_at**	0.0381487	-1.46	ARHGEF7	Rho guanine nucleotide exchange factor 7
**203177_x_at**	0.0305048	-1.46	TFAM	Transcription factor A, mitochondrial
**206003_at**	0.0443178	-1.46	CEP135	Centrosomal protein 135
**206928_at**	0.0182373	-1.46	ZNF124	Zinc finger protein 124
**212196_at**	0.0153135	-1.46	IL6ST	Interleukin 6 signal transducer
**212621_at**	0.0199366	-1.46	NEMP1	Nuclear envelope integral membrane protein 1

EXCLI Journal 2021;20:126-141– ISSN 1611-2156
Received: December 08, 2020, accepted: January 11, 2021, published: January 18, 2021

**213186_at**	0.0059208	-1.46	DZIP3	DAZ interacting zinc finger protein 3
**215483_at**	0.016941	-1.46	AKAP9	A-kinase anchoring protein 9
**216527_at**	0.0158276	-1.46	HCG18	HLA complex group 18 (non-protein coding)
**219109_at**	0.0107947	-1.46	SPAG16	Sperm associated antigen 16
**221510_s_at**	0.0389397	-1.46	GLS	Glutaminase
**225688_s_at**	0.0464242	-1.46	PHLDB2	Pleckstrin homology like domain family B member 2
**225839_at**	0.0139805	-1.46	RBM33	RNA binding motif protein 33
**226693_at**	0.0275383	-1.46	SDHAP2///LINC00969///SDHAP1	Succinate dehydrogenase complex flavoprotein subunit A pseudogene 2///long intergenic non-protein coding RNA 969///succinate dehydrogenase complex flavoprotein subunit A pseudogene 1
**227409_at**	0.0058063	-1.46	PPP1R3E	Protein phosphatase 1 regulatory subunit 3E
**227531_at**	0.0364457	-1.46	CLOCK	Clock circadian regulator
**230885_at**	0.0340499	-1.46	LOC101930112///SPG7	Uncharacterized LOC101930112///SPG7, paraplegin matrix AAA peptidase subunit
**232568_at**	0.0072784	-1.46	MGC24103	Uncharacterized MGC24103
**235031_at**	0.0012405	-1.46	INVS///WSB1	Inversin///WD repeat and SOCS box containing 1
**236832_at**	0.0405056	-1.46	ADCY10P1	Adenylate cyclase 10, soluble pseudogene 1
**1558675_s_at**	0.0250551	-1.45	NEMF	Nuclear export mediator factor
**201567_s_at**	0.0439583	-1.45	GOLGA4	Golgin A4
**202309_at**	0.0251536	-1.45	MTHFD1	Methylenetetrahydrofolate dehydrogenase, cyclohydrolase and formyltetrahydrofolate synthetase 1
**209795_at**	0.0044017	-1.45	CD69	CD69 molecule
**210396_s_at**	0.0063917	-1.45	SMG1P5///BOLA2///SMG1P2	SMG1P5, nonsense mediated mrna decay associated PI3K related kinase pseudogene 5///bola family member 2///SMG1P2, nonsense mediated mrna decay associated PI3K related kinase pseudogene 2
**212982_at**	0.0291306	-1.45	ZDHHC17	Zinc finger DHHC-type containing 17
**219173_at**	0.0157955	-1.45	MYO15B	Myosin XVB
**223251_s_at**	0.0108865	-1.45	ANKRD10	Ankyrin repeat domain 10
**225758_s_at**	0.0197664	-1.45	TUBGCP6	Tubulin gamma complex associated protein 6
**225949_at**	0.0111398	-1.45	MIR6845///NRBP2	Microrna 6845///nuclear receptor binding protein 2
**226203_at**	0.0180287	-1.45	MYO9A	Myosin IXA

EXCLI Journal 2021;20:126-141– ISSN 1611-2156
Received: December 08, 2020, accepted: January 11, 2021, published: January 18, 2021

**228781_at**	0.00524	-1.45	OBFC1	Oligonucleotide/oligosaccharide binding fold containing 1
**229041_s_at**	0.0252839	-1.45	ITGB2-AS1	ITGB2 antisense RNA 1
**238458_at**	0.0079056	-1.45	MICU3	Mitochondrial calcium uptake family member 3
**238788_at**	0.0341171	-1.45	PHBP19	Prohibitin pseudogene 19
**239482_x_at**	0.0296991	-1.45	ZNF708	Zinc finger protein 708
**1558458_at**	0.0262681	-1.44	LOC101928623///LOC401320	Uncharacterized LOC101928623///uncharacterized LOC401320
**205976_at**	0.0110656	-1.44	FASTKD2	FAST kinase domains 2
**208309_s_at**	0.043449	-1.44	MALT1	MALT1 paracaspase
**209306_s_at**	0.0342563	-1.44	SWAP70	SWAP switching B-cell complex 70kda subunit
**212373_at**	0.0086886	-1.44	FEM1B	Fem-1 homolog B
**213773_x_at**	0.0341511	-1.44	NSUN5	NOP2/Sun RNA methyltransferase family member 5
**214241_at**	0.0418552	-1.44	NDUFB8	NADH:ubiquinone oxidoreductase subunit B8
**219848_s_at**	0.0396429	-1.44	ZNF432	Zinc finger protein 432
**219915_s_at**	0.0102654	-1.44	SLC16A10	Solute carrier family 16 member 10
**222849_s_at**	0.0044337	-1.44	SCRN3	Secernin 3
**226922_at**	0.0403762	-1.44	RANBP2	RAN binding protein 2
**231929_at**	0.0315461	-1.44	IKZF2	IKAROS family zinc finger 2
**232138_at**	0.0050497	-1.44	MBNL2	Muscleblind like splicing regulator 2
**236401_at**	0.0176589	-1.44	GIMAP1	Gtpase, IMAP family member 1
**241018_at**	0.0033075	-1.44	TMEM59	Transmembrane protein 59
**201177_s_at**	0.0229803	-1.43	UBA2	Ubiquitin like modifier activating enzyme 2
**203233_at**	0.0159175	-1.43	IL4R	Interleukin 4 receptor
**210042_s_at**	0.0080317	-1.43	CTSZ	Cathepsin Z
**212361_s_at**	0.013858	-1.43	ATP2A2	Atpase sarcoplasmic/endoplasmic reticulum Ca2+ transporting 2
**215201_at**	0.0343738	-1.43	REPS1	RALBP1 associated Eps domain containing 1
**225189_s_at**	0.0453091	-1.43	RAPH1	Ras association (ralgds/AF-6) and pleckstrin homology domains 1
**226508_at**	0.0419146	-1.43	PHC3	Polyhomeotic homolog 3
**226925_at**	0.003897	-1.43	PXYLP1	2-phosphoxylose phosphatase 1
**227471_at**	0.0354439	-1.43	HACE1	HECT domain and ankyrin repeat containing E3 ubiquitin protein ligase 1
**227809_at**	0.0065416	-1.43	ZC3H6	Zinc finger CCCH-type containing 6
**229712_at**	0.0013843	-1.43	SNAPC3	Small nuclear RNA activating complex polypeptide 3
**233019_at**	0.0193546	-1.43	CNOT7	CCR4-NOT transcription complex subunit 7
**235695_at**	0.0152717	-1.43	INPP4A	Inositol polyphosphate-4-phosphatase type I A

EXCLI Journal 2021;20:126-141– ISSN 1611-2156
Received: December 08, 2020, accepted: January 11, 2021, published: January 18, 2021

**240173_at**	0.0012212	-1.43	GPRIN3	GPRIN family member 3
**1559975_at**	0.005431	-1.42	BTG1	BTG anti-proliferation factor 1
**202259_s_at**	0.0346411	-1.42	N4BP2L2	NEDD4 binding protein 2 like 2
**203146_s_at**	0.021376	-1.42	GABBR1	Gamma-aminobutyric acid type B receptor subunit 1
**204332_s_at**	0.0069861	-1.42	AGA	Aspartylglucosaminidase
**209358_at**	0.0199377	-1.42	TAF11	TATA-box binding protein associ-ated factor 11
**218371_s_at**	0.0234418	-1.42	PSPC1	Paraspeckle component 1
**219342_at**	0.0224206	-1.42	CASD1	CAS1 domain containing 1
**221234_s_at**	0.0346578	-1.42	BACH2	BTB domain and CNC homolog 2
**222294_s_at**	0.0321751	-1.42	RAB27A	RAB27A, member RAS oncogene family
**225835_at**	0.0246476	-1.42	SLC12A2	Solute carrier family 12 member 2
**227732_at**	0.0034106	-1.42	ATXN7L1	Ataxin 7 like 1
**228991_at**	0.0425462	-1.42	CDK13	Cyclin dependent kinase 13
**231843_at**	0.0472329	-1.42	DDX55	DEAD-box helicase 55
**238057_at**	0.010541	-1.42	USP45	Ubiquitin specific peptidase 45
**239250_at**	0.0246868	-1.42	ZNF542P	Zinc finger protein 542, pseudo-gene
**1557113_at**	0.0037826	-1.41	LOC283588	Uncharacterized LOC283588
**218962_s_at**	0.0327141	-1.41	TMEM168	Transmembrane protein 168
**219002_at**	0.0199892	-1.41	FASTKD1	FAST kinase domains 1
**220576_at**	0.038363	-1.41	PGAP1	Post-GPI attachment to proteins 1
**222127_s_at**	0.0385719	-1.41	EXOC1	Exocyst complex component 1
**224406_s_at**	0.0344549	-1.41	FCRL5	Fc receptor like 5
**225470_at**	0.0141121	-1.41	NUP35	Nucleoporin 35
**227492_at**	0.0430771	-1.41	OCLN	Occludin
**235220_at**	0.0050349	-1.41	YIPF4	Yip1 domain family member 4
**238744_at**	0.0060829	-1.41	DPH6	Diphthamine biosynthesis 6
**1554345_a_at**	0.0201379	-1.4	GIN1	Gypsy retrotransposon integrase 1
**1557302_at**	0.0072151	-1.4	ZNF585B	Zinc finger protein 585B
**1568900_a_at**	0.0030906	-1.4	ZNF568	Zinc finger protein 568
**201018_at**	0.0107057	-1.4	EIF1AX	Eukaryotic translation initiation fac-tor 1A, X-linked
**202515_at**	0.0039979	-1.4	DLG1	Discs large MAGUK scaffold pro-tein 1
**203519_s_at**	0.0186427	-1.4	UPF2	UPF2 regulator of nonsense tran-scripts homolog (yeast)
**204861_s_at**	0.0022376	-1.4	NAIP	NLR family apoptosis inhibitory protein
**205267_at**	0.0434024	-1.4	POU2AF1	POU class 2 associating factor 1
**209681_at**	0.0035685	-1.4	SLC19A2	Solute carrier family 19 member 2
**213213_at**	0.0454585	-1.4	DIDO1	Death inducer-obliterator 1
**214148_at**	0.0216003	-1.4	LOC100507 424///ITFG2	Uncharacterized LOC100507424///integrin alpha FG-GAP repeat containing 2

EXCLI Journal 2021;20:126-141– ISSN 1611-2156
Received: December 08, 2020, accepted: January 11, 2021, published: January 18, 2021

**218263_s_at**	0.0402554	-1.4	ZBED5	Zinc finger BED-type containing 5
**218545_at**	0.0042238	-1.4	CCDC91	Coiled-coil domain containing 91
**226660_at**	0.0064176	-1.4	RPS6KB1	Ribosomal protein S6 kinase B1
**227025_at**	0.0218471	-1.4	PPHLN1	Periphilin 1
**227505_at**	0.0348619	-1.4	MED28	Mediator complex subunit 28
**228077_at**	0.0248985	-1.4	MRI1	Methylthioribose-1-phosphate isomerase 1
**228555_at**	0.0184552	-1.4	CAMK2D	Calcium/calmodulin dependent protein kinase II delta
**229510_at**	0.0075888	-1.4	MS4A14	Membrane spanning 4-domains A14
**230178_s_at**	0.0209649	-1.4	ELP2	Elongator acetyltransferase complex subunit 2
**230229_at**	0.0372815	-1.4	DLG1	Discs large MAGUK scaffold protein 1
**230300_at**	0.0073441	-1.4	PSMA5	Proteasome subunit alpha 5
**235124_at**	0.0306967	-1.4	EIF3J-AS1	EIF3J antisense RNA 1 (head to head)
**235434_at**	0.0159233	-1.4	CCDC134	Coiled-coil domain containing 134
**239193_at**	0.0011559	-1.4	FUBP3	Far upstream element binding protein 3
**243264_s_at**	0.0042352	-1.4	C8orf44-SGK3///C8orf44///SGK3	C8orf44-SGK3 readthrough///chromosome 8 open reading frame 44///serum/glucocorticoid regulated kinase family member 3
**1568870_at**	0.0058652	-1.39	SLC24A4	Solute carrier family 24 member 4
**209064_x_at**	0.0091068	-1.39	PAIP1	Poly(A) binding protein interacting protein 1
**209989_at**	0.0170889	-1.39	ZNF268	Zinc finger protein 268
**210528_at**	0.049459	-1.39	MR1	Major histocompatibility complex, class I-related
**213888_s_at**	0.0318649	-1.39	TRAF3IP3	TRAF3 interacting protein 3
**214764_at**	0.0216483	-1.39	RRP15	Ribosomal RNA processing 15 homolog
**222311_s_at**	0.0050502	-1.39	SCAF4	SR-related CTD associated factor 4
**225346_at**	0.0096261	-1.39	MTERF2	Mitochondrial transcription termination factor 2
**226363_at**	0.0078465	-1.39	ABCC5	ATP binding cassette subfamily C member 5
**226776_at**	0.0015813	-1.39	ENY2	ENY2, transcription and export complex 2 subunit
**227877_at**	0.0114077	-1.39	ANXA2R	Annexin A2 receptor
**232312_at**	0.0432016	-1.39	PPP6R3	Protein phosphatase 6 regulatory subunit 3
**1552733_at**	0.0168807	-1.38	KLHDC1	Kelch domain containing 1
**1554036_at**	0.0014558	-1.38	ZBTB24	Zinc finger and BTB domain containing 24
**1555120_at**	0.011809	-1.38	CD96	CD96 molecule
**201636_at**	0.0090455	-1.38	FXR1	FMR1 autosomal homolog 1

EXCLI Journal 2021;20:126-141– ISSN 1611-2156
Received: December 08, 2020, accepted: January 11, 2021, published: January 18, 2021

**202850_at**	0.0108822	-1.38	ABCD3	ATP binding cassette subfamily D member 3
**204568_at**	0.0208178	-1.38	ATG14	Autophagy related 14
**206874_s_at**	0.0213972	-1.38	SLK	STE20 like kinase
**209884_s_at**	0.0424378	-1.38	SLC4A7	Solute carrier family 4 member 7
**218769_s_at**	0.0324714	-1.38	ANKRA2	Ankyrin repeat family A member 2
**218984_at**	0.0194966	-1.38	PUS7	Pseudouridylate synthase 7 (putative)
**219487_at**	0.0166373	-1.38	BBS10	Bardet-Biedl syndrome 10
**219551_at**	0.0181623	-1.38	EAF2	ELL associated factor 2
**221221_s_at**	0.0032861	-1.38	KLHL3	Kelch like family member 3
**223213_s_at**	0.0301688	-1.38	ZHX1	Zinc fingers and homeoboxes 1
**225431_x_at**	0.0479925	-1.38	PM20D2	Peptidase M20 domain containing 2
**225953_at**	0.0083013	-1.38	RPRD1A	Regulation of nuclear pre-mrna domain containing 1A
**227162_at**	0.0109188	-1.38	ZBTB26	Zinc finger and BTB domain containing 26
**227776_at**	0.009518	-1.38	ACER3	Alkaline ceramidase 3
**228853_at**	0.0144535	-1.38	STYX	Serine/threonine/tyrosine interacting protein
**231075_x_at**	0.0036201	-1.38	ABI2	Abl-interactor 2
**242916_at**	0.006084	-1.38	CNTRL	Centriolin
**244741_s_at**	0.0147026	-1.38	ZNF667-AS1	ZNF667 antisense RNA 1 (head to head)
**1552633_at**	0.0092225	-1.37	ZNF101	Zinc finger protein 101
**202326_at**	0.0260133	-1.37	EHMT2	Euchromatic histone lysine methyltransferase 2
**204773_at**	0.0354883	-1.37	IL11RA	Interleukin 11 receptor subunit alpha
**205255_x_at**	0.0244989	-1.37	TCF7	Transcription factor 7 (T-cell specific, HMG-box)
**206565_x_at**	0.0068866	-1.37	SMA4	Glucuronidase beta pseudogene
**212922_s_at**	0.044962	-1.37	SMYD2	SET and MYND domain containing 2
**213518_at**	0.012209	-1.37	PRKCI	Protein kinase C iota
**213743_at**	0.0270868	-1.37	CCNT2	Cyclin T2
**225814_at**	0.0283249	-1.37	XRN1	5'-3' exoribonuclease 1
**227587_at**	0.0440828	-1.37	KRI1	KRI1 homolog
**228416_at**	0.0337622	-1.37	ACVR2A	Activin A receptor type 2A
**235728_at**	0.010515	-1.37	ZFP3	ZFP3 zinc finger protein
**244743_x_at**	0.0085704	-1.37	ZNF138	Zinc finger protein 138
**55081_at**	0.0030728	-1.37	MICALL1	MICAL like 1
**1557394_at**	0.0019589	-1.36	DLGAP4	DLG associated protein 4
**1558338_at**	0.0090406	-1.36	SEMA6A	Semaphorin 6A
**211106_at**	0.0285252	-1.36	SUPT3H	SPT3 homolog, SAGA and STAGA complex component

EXCLI Journal 2021;20:126-141– ISSN 1611-2156
Received: December 08, 2020, accepted: January 11, 2021, published: January 18, 2021

**213908_at**	0.024702	-1.36	WHAMMP2///WHAMMP3	WAS protein homolog associated with actin, golgi membranes and microtubules pseudogene 2///WAS protein homolog associated with actin, golgi membranes and micro-tubules pseudogene 3
**218239_s_at**	0.0349287	-1.36	GTPBP4	GTP binding protein 4
**219972_s_at**	0.0067213	-1.36	PCNX4	Pecanex homolog 4 (Drosophila)
**219980_at**	0.0333056	-1.36	ABHD18	Abhydrolase domain containing 18
**222696_at**	0.031904	-1.36	AXIN2	Axin 2
**224876_at**	0.0353693	-1.36	C5orf24	Chromosome 5 open reading frame 24
**227749_at**	0.0310562	-1.36	POU2F2	POU class 2 homeobox 2
**228291_s_at**	0.0079457	-1.36	KIZ	Kizuna centrosomal protein
**230434_at**	0.0372431	-1.36	PHOS-PHO2	Phosphatase, orphan 2
**230683_at**	0.0035967	-1.36	PPP4R1L	Protein phosphatase 4 regulatory subunit 1 like (pseudogene)
**233241_at**	0.0071083	-1.36	KIZ	Kizuna centrosomal protein
**234762_x_at**	0.0393893	-1.36	NLN	Neurolysin
**235620_x_at**	0.0391612	-1.36	ZMYM5	Zinc finger MYM-type containing 5
**239146_at**	0.0116231	-1.36	CLDND1	Claudin domain containing 1
**240344_x_at**	0.0315297	-1.36	LYRM7	LYR motif containing 7
**243664_at**	0.0356003	-1.36	TXNL1	Thioredoxin like 1
**243957_at**	0.0188207	-1.36	LOC100128108	Putative ubiquitin-conjugating en-zyme E2Q2-like protein
**203263_s_at**	0.014973	-1.35	ARHGEF9	Cdc42 guanine nucleotide ex-change factor 9
**210705_s_at**	0.0058792	-1.35	TRIM5	Tripartite motif containing 5
**213170_at**	0.0423985	-1.35	GPX7	Glutathione peroxidase 7
**214220_s_at**	0.003507	-1.35	ALMS1	ALMS1, centrosome and basal body associated protein
**221535_at**	0.0320726	-1.35	LSG1	Large 60S subunit nuclear export gtpase 1
**221564_at**	0.0226117	-1.35	PRMT2	Protein arginine methyltransferase 2
**221568_s_at**	0.0466277	-1.35	LIN7C	Lin-7 homolog C, crumbs cell po-larity complex component
**227064_at**	0.0347297	-1.35	ANKRD40	Ankyrin repeat domain 40
**232012_at**	0.0079571	-1.35	CAPN1	Calpain 1
**238633_at**	0.0066264	-1.35	EPC1	Enhancer of polycomb homolog 1
**238649_at**	0.0100495	-1.35	PITPNC1	Phosphatidylinositol transfer pro-tein, cytoplasmic 1
**1553955_at**	0.0275593	-1.34	PPP1R21	Protein phosphatase 1 regulatory subunit 21
**1563051_at**	0.0136326	-1.34	OSBP	Oxysterol binding protein
**202724_s_at**	0.0266696	-1.34	FOXO1	Forkhead box O1
**205176_s_at**	0.04136	-1.34	ITGB3BP	Integrin subunit beta 3 binding pro-tein

EXCLI Journal 2021;20:126-141– ISSN 1611-2156
Received: December 08, 2020, accepted: January 11, 2021, published: January 18, 2021

**206060_s_at**	0.0345582	-1.34	PTPN22	Protein tyrosine phosphatase, non-receptor type 22
**213918_s_at**	0.0278241	-1.34	NIPBL	NIPBL, cohesin loading factor
**218316_at**	0.0120543	-1.34	TIMM9	Translocase of inner mitochondrial membrane 9
**221626_at**	0.0493506	-1.34	ZNF506	Zinc finger protein 506
**222146_s_at**	0.0403448	-1.34	TCF4	Transcription factor 4
**222566_at**	0.0032093	-1.34	KMT5B	Lysine methyltransferase 5B
**225463_x_at**	0.0220539	-1.34	GPR89A/// GPR89B	G protein-coupled receptor 89A///G protein-coupled receptor 89B
**226747_at**	0.0171295	-1.34	TXNDC16	Thioredoxin domain containing 16
**227077_at**	0.0265746	-1.34	ZNF286B/// ZNF286A	Zinc finger protein 286B///zinc finger protein 286A
**228331_at**	0.0461176	-1.34	C11orf31	Chromosome 11 open reading frame 31
**231022_at**	0.0077752	-1.34	OCLN	Occludin
**1556474_a_at**	0.0359462	-1.33	LOC285097	Uncharacterized FLJ38379
**1557263_s_at**	0.003185	-1.33	LOC102724 814	Uncharacterized LOC102724814
**1569472_s_at**	0.025507	-1.33	TTC3P1///T TC3	Tetratricopeptide repeat domain 3 pseudogene 1///tetratricopeptide repeat domain 3
**201690_s_at**	0.0261415	-1.33	TPD52	Tumor protein D52
**202042_at**	0.0373391	-1.33	LOC101928 623///HARS	Uncharacterized LOC101928623///histidyl-trna synthetase
**203743_s_at**	0.0277342	-1.33	TDG	Thymine DNA glycosylase
**203818_s_at**	0.037515	-1.33	SF3A3	Splicing factor 3a subunit 3
**209268_at**	0.0323701	-1.33	VPS45	Vacuolar protein sorting 45 homolog
**212310_at**	0.0047902	-1.33	MIA3	MIA family member 3, ER export factor
**213480_at**	0.0439464	-1.33	VAMP4	Vesicle associated membrane protein 4
**218712_at**	0.0088103	-1.33	C1orf109	Chromosome 1 open reading frame 109
**222336_at**	0.0015934	-1.33	SMIM14	Small integral membrane protein 14
**224365_s_at**	0.0081666	-1.33	TIGD7	Tigger transposable element derived 7
**225580_at**	0.0228853	-1.33	MRPL50	Mitochondrial ribosomal protein L50
**225586_at**	0.0069554	-1.33	DPH7	Diphthamide biosynthesis 7
**228095_at**	0.0234773	-1.33	PHF14	PHD finger protein 14
**228099_at**	0.0151534	-1.33	ZNF550	Zinc finger protein 550
**228109_at**	0.0253071	-1.33	RASGRF2	Ras protein specific guanine nucleotide releasing factor 2
**229672_at**	0.0058957	-1.33	UQCC1	Ubiquinol-cytochrome c reductase complex assembly factor 1

**230820_at**	0.0225716	-1.33	SMURF2	SMAD specific E3 ubiquitin protein ligase 2
**231964_at**	0.0160617	-1.33	BICD1	BICD cargo adaptor 1
**232515_at**	0.024288	-1.33	GPR75-ASB3///ASB3///GPR75	GPR75-ASB3 readthrough///ankyrin repeat and SOCS box containing 3///G protein-coupled receptor 75
**233656_s_at**	0.0196435	-1.33	VPS54	VPS54, GARP complex subunit
**233874_at**	0.005119	-1.33	SLAIN2	SLAIN motif family member 2
**235661_at**	0.0302557	-1.33	POU2F2	POU class 2 homeobox 2
**52285_f_at**	0.0342941	-1.33	CEP76	Centrosomal protein 76
**202983_at**	0.0228008	-1.32	HLTF	Helicase like transcription factor
**218346_s_at**	0.0121224	-1.32	SESN1	Sestrin 1
**221773_at**	0.0142776	-1.32	ELK3	ELK3, ETS transcription factor
**221969_at**	0.0196603	-1.32	PAX5	Paired box 5
**222630_at**	0.0288121	-1.32	RFX7	Regulatory factor X7
**225394_s_at**	0.0290562	-1.32	ZCRB1	Zinc finger CCHC-type and RNA binding motif containing 1
**225409_at**	0.0494073	-1.32	COA5	Cytochrome c oxidase assembly factor 5
**226527_at**	0.0127352	-1.32	RPRD2	Regulation of nuclear pre-mrna domain containing 2
**226679_at**	0.0451475	-1.32	SLC26A11	Solute carrier family 26 member 11
**226964_at**	0.0144096	-1.32	TTBK2	Tau tubulin kinase 2
**232295_at**	0.0059119	-1.32	GFM1	G elongation factor mitochondrial 1
**238049_at**	0.0040623	-1.32	GRAMD3	GRAM domain containing 3
**238695_s_at**	0.0363643	-1.32	RAB39B	RAB39B, member RAS oncogene family
**1554628_at**	0.0037929	-1.31	ZNF57	Zinc finger protein 57
**1564520_s_at**	0.0097721	-1.31	PRMT5	Protein arginine methyltransferase 5
**1569107_s_at**	0.0263213	-1.31	ZFP69	ZFP69 zinc finger protein
**201189_s_at**	0.0224637	-1.31	ITPR3	Inositol 1,4,5-trisphosphate receptor type 3
**204662_at**	0.0273976	-1.31	CCP110	Centriolar coiled-coil protein 110
**215921_at**	0.0331422	-1.31	NPIPA5///NPIPB6///NPIPB8///NPIPB3	Nuclear pore complex interacting protein family member A5///nuclear pore complex interacting protein family member B6///nuclear pore complex interacting protein family member B8///nuclear pore complex interacting protein family member B3
**217842_at**	0.0365346	-1.31	C7orf55-LUC7L2///LUC7L2	C7orf55-LUC7L2 readthrough///LUC7 like 2, pre-mrna splicing factor
**220019_s_at**	0.0188328	-1.31	ZNF224	Zinc finger protein 224
**223937_at**	0.0212423	-1.31	FOXP1	Forkhead box P1
**224304_x_at**	0.0057488	-1.31	NIN	Ninein

EXCLI Journal 2021;20:126-141– ISSN 1611-2156
Received: December 08, 2020, accepted: January 11, 2021, published: January 18, 2021

**225741_at**	0.0113755	-1.31	THUMPD3	THUMP domain containing 3
**227680_at**	0.0453594	-1.31	ZNF326	Zinc finger protein 326
**228084_at**	0.0498903	-1.31	PLA2G12A	Phospholipase A2 group XIIA
**229615_at**	0.0101617	-1.31	SLX4IP	SLX4 interacting protein
**232156_at**	0.0145252	-1.31	AP5M1	Adaptor related protein complex 5 mu 1 subunit
**235722_at**	0.0280451	-1.31	SYNJ2BP-COX16///SYNJ2BP	SYNJ2BP-COX16 readthrough///synaptojanin 2 binding protein
**239846_at**	0.0161562	-1.31	MTHFD1	Methylenetetrahydrofolate dehydrogenase, cyclohydrolase and formyltetrahydrofolate synthetase 1
**242028_at**	0.0028148	-1.31	ZNF709	Zinc finger protein 709
**1553743_at**	0.0119396	-1.3	METTL21A	Methyltransferase like 21A
**205055_at**	0.0496827	-1.3	ITGAE	Integrin subunit alpha E
**207394_at**	0.0198291	-1.3	ZNF137P	Zinc finger protein 137, pseudogene
**217752_s_at**	0.0383341	-1.3	CNDP2	CNDP dipeptidase 2 (metallopeptidase M20 family)
**218311_at**	0.0126641	-1.3	MAP4K3	Mitogen-activated protein kinase kinase kinase kinase 3
**219467_at**	0.0409019	-1.3	GIN1	Gypsy retrotransposon integrase 1
**219757_s_at**	0.0060434	-1.3	TMEM260	Transmembrane protein 260
**222317_at**	0.0236819	-1.3	PDE3B	Phosphodiesterase 3B
**222762_x_at**	0.0035368	-1.3	LIMD1	LIM domains containing 1
**223418_x_at**	0.026937	-1.3	ANKRD13C	Ankyrin repeat domain 13C
**225348_at**	0.0272803	-1.3	SRSF10	Serine and arginine rich splicing factor 10
**225444_at**	0.0439456	-1.3	UBN2	Ubinuclein 2
**225847_at**	0.0325433	-1.3	NCEH1	Neutral cholesterol ester hydrolase 1
**226027_at**	0.0020769	-1.3	SWI5	SWI5 homologous recombination repair protein
**226962_at**	0.0337231	-1.3	ZBTB41	Zinc finger and BTB domain containing 41
**227247_at**	0.012915	-1.3	PLEKHA8	Pleckstrin homology domain containing A8
**239726_at**	0.0447565	-1.3	ANK3	Ankyrin 3, node of Ranvier (ankyrin G)
**242297_at**	0.0482775	-1.3	RREB1	Ras responsive element binding protein 1
**244761_at**	0.0309878	-1.3	C5orf63	Chromosome 5 open reading frame 63
**1570210_x_at**	0.0345156	-1.29	PPP6R2	Protein phosphatase 6 regulatory subunit 2
**204619_s_at**	0.0188232	-1.29	VCAN	Versican
**209045_at**	0.0423284	-1.29	XPNPEP1	X-prolyl aminopeptidase 1
**209153_s_at**	0.0236786	-1.29	TCF3	Transcription factor 3

**209805_at**	0.021511	-1.29	PMS2CL///PMS2	PMS2 C-terminal like pseudo-gene///PMS1 homolog 2, mismatch repair system component
**216857_at**	0.032986	-1.29	IL23A	Interleukin 23 subunit alpha
**218155_x_at**	0.0066968	-1.29	TSR1	TSR1, ribosome maturation factor
**218602_s_at**	0.0358337	-1.29	HAUS6	HAUS augmin like complex subunit 6
**221090_s_at**	0.041446	-1.29	OGFOD1	2-oxoglutarate and iron dependent oxygenase domain containing 1
**221669_s_at**	0.0418146	-1.29	ACAD8	Acyl-coa dehydrogenase family member 8
**225216_at**	0.0078947	-1.29	FAM199X	Family with sequence similarity 199, X-linked
**226868_at**	0.044415	-1.29	GXYLT1	Glucoside xylosyltransferase 1
**227009_at**	0.0174666	-1.29	LOC100507431	Uncharacterized LOC100507431
**227450_at**	0.0344873	-1.29	ERP27	Endoplasmic reticulum protein 27
**228258_at**	0.0484911	-1.29	TBC1D10C	TBC1 domain family member 10C
**229455_at**	0.0019048	-1.29	LOC102723694	Uncharacterized LOC102723694
**229732_at**	0.0068345	-1.29	ZNF823	Zinc finger protein 823
**229785_at**	0.010299	-1.29	KRIT1	KRIT1, ankyrin repeat containing
**230379_x_at**	0.017289	-1.29	NDUFAF7	NADH:ubiquinone oxidoreductase complex assembly factor 7
**230640_at**	0.0263406	-1.29	FMNL3	Formin like 3
**231836_at**	0.0413743	-1.29	HKR1	HKR1, GLI-Kruppel zinc finger family member
**239412_at**	0.0199853	-1.29	IRF5	Interferon regulatory factor 5
**241968_at**	0.0190559	-1.29	ABHD18	Abhydrolase domain containing 18
**1552323_s_at**	0.0181997	-1.28	FAM122C	Family with sequence similarity 122C
**202946_s_at**	0.0329103	-1.28	BTBD3	BTB domain containing 3
**203753_at**	0.007431	-1.28	TCF4	Transcription factor 4
**204523_at**	0.0117466	-1.28	ZNF140	Zinc finger protein 140
**211721_s_at**	0.0063983	-1.28	ZNF551	Zinc finger protein 551
**212160_at**	0.0226781	-1.28	XPOT	Exportin for trna
**218501_at**	0.0456311	-1.28	ARHGEF3	Rho guanine nucleotide exchange factor 3
**218943_s_at**	0.0381417	-1.28	DDX58	DEXD/H-box helicase 58
**223437_at**	0.047176	-1.28	PPARA	Peroxisome proliferator activated receptor alpha
**226815_at**	0.0032347	-1.28	TAMM41	TAM41 mitochondrial translocator assembly and maintenance homolog
**227172_at**	0.0449678	-1.28	TMEM116	Transmembrane protein 116
**227626_at**	0.029883	-1.28	PAQR8	Progestin and adipoq receptor family member 8
**227847_at**	0.0180003	-1.28	EPM2AIP1	EPM2A interacting protein 1
**228365_at**	0.0154394	-1.28	CPNE8	Copine 8

EXCLI Journal 2021;20:126-141– ISSN 1611-2156
Received: December 08, 2020, accepted: January 11, 2021, published: January 18, 2021

**229468_at**	0.0073258	-1.28	TEN1-CDK3///CDK3	TEN1-CDK3 readthrough (NMD candidate)///cyclin dependent kinase 3
**229549_at**	0.0312826	-1.28	CALU	Calumenin
**229656_s_at**	0.0442202	-1.28	EML6	Echinoderm microtubule associated protein like 6
**230298_at**	0.0433104	-1.28	MBLAC2	Metallo-beta-lactamase domain containing 2
**230815_at**	0.0092986	-1.28	LOC389765	Kinesin family member 27 pseudogene
**231909_x_at**	0.0018535	-1.28	ODF2L	Outer dense fiber of sperm tails 2 like
**239043_at**	0.0419698	-1.28	ZNF404	Zinc finger protein 404
**1555883_s_at**	0.0025434	-1.27	SPIN3	Spindlin family member 3
**1559067_a_at**	0.0147812	-1.27	LOC158402	Uncharacterized LOC158402
**200597_at**	0.0100377	-1.27	EIF3A	Eukaryotic translation initiation factor 3 subunit A
**203062_s_at**	0.0309021	-1.27	MDC1	Mediator of DNA damage checkpoint 1
**213007_at**	0.0085117	-1.27	FANCI	Fanconi anemia complementation group I
**213882_at**	0.0313463	-1.27	TM2D1	TM2 domain containing 1
**218259_at**	0.0230491	-1.27	MKL2	MKL1/myocardin like 2
**220175_s_at**	0.0340787	-1.27	CBWD7///CBWD6///CBWD3///CBWD5///CBWD2///CBWD1	COBW domain containing 7///COBW domain containing 6///COBW domain containing 3///COBW domain containing 5///COBW domain containing 2///COBW domain containing 1
**221087_s_at**	0.0142506	-1.27	APOL3	Apolipoprotein L3
**222073_at**	0.0309235	-1.27	COL4A3	Collagen type IV alpha 3 chain
**223236_at**	0.0046979	-1.27	NSRP1	Nuclear speckle splicing regulatory protein 1
**226603_at**	0.0334164	-1.27	SAMD9L	Sterile alpha motif domain containing 9 like
**227121_at**	0.0468245	-1.27	ZBTB20	Zinc finger and BTB domain containing 20
**227173_s_at**	0.0076747	-1.27	BACH2	BTB domain and CNC homolog 2
**228624_at**	0.0078505	-1.27	TMEM144	Transmembrane protein 144
**228961_at**	0.0232441	-1.27	MIER3	MIER family member 3
**231234_at**	0.0041255	-1.27	CTSC	Cathepsin C
**231960_at**	0.0080895	-1.27	BRWD1	Bromodomain and WD repeat domain containing 1
**232637_at**	0.041475	-1.27	2-Sep	Septin 2
**235241_at**	0.0183207	-1.27	SLC38A9	Solute carrier family 38 member 9
**236382_at**	0.0020776	-1.27	WRAP73	WD repeat containing, antisense to TP73
**238191_at**	0.0101967	-1.27	SDHC	Succinate dehydrogenase complex subunit C

EXCLI Journal 2021;20:126-141– ISSN 1611-2156
Received: December 08, 2020, accepted: January 11, 2021, published: January 18, 2021

**239407_at**	0.034651	-1.27	PAXBP1-AS1	PAXBP1 antisense RNA 1
**242139_s_at**	0.0283416	-1.27	ERVK3-1///ZNF8	Endogenous retrovirus group K3 member 1///zinc finger protein 8
**242518_at**	0.0418485	-1.27	RAD51-AS1	RAD51 antisense RNA 1 (head to head)
**1558097_at**	0.044593	-1.26	PRR14L	Proline rich 14 like
**203240_at**	0.0144934	-1.26	FCGBP	Fc fragment of igg binding protein
**204349_at**	0.00646	-1.26	MED7	Mediator complex subunit 7
**210622_x_at**	0.0463178	-1.26	CDK10	Cyclin dependent kinase 10
**212401_s_at**	0.0364082	-1.26	CDK11A///CDK11B	Cyclin dependent kinase 11A///cyclin dependent kinase 11B
**213304_at**	0.0324886	-1.26	FAM179B	Family with sequence similarity 179 member B
**214960_at**	0.0194063	-1.26	API5	Apoptosis inhibitor 5
**219244_s_at**	0.0281208	-1.26	MRPL46	Mitochondrial ribosomal protein L46
**221935_s_at**	0.0026024	-1.26	EOGT	EGF domain specific O-linked N-acetylglucosamine transferase
**222487_s_at**	0.0262002	-1.26	RPS27L	Ribosomal protein S27 like
**224820_at**	0.0304244	-1.26	COX20	COX20, cytochrome c oxidase assembly factor
**226046_at**	0.0044475	-1.26	MAPK8	Mitogen-activated protein kinase 8
**227533_at**	0.0075232	-1.26	RALGPS2	Ral GEF with PH domain and SH3 binding motif 2
**227882_at**	0.0188891	-1.26	FKRP	Fukutin related protein
**227926_s_at**	0.0068996	-1.26	NBPF20	Neuroblastoma breakpoint family member 20
**230416_at**	0.0042044	-1.26	PDZD8	PDZ domain containing 8
**236826_at**	0.0111298	-1.26	TTC39B	Tetratricopeptide repeat domain 39B
**238681_at**	0.0449087	-1.26	GDPD1	Glycerophosphodiester phosphodiesterase domain containing 1
**239278_at**	0.0337712	-1.26	BCAS4	Breast carcinoma amplified sequence 4
**1555872_a_at**	0.0193438	-1.25	LOC728903///GLIDR	Uncharacterized LOC728903///glioblastoma down-regulated RNA
**1556672_a_at**	0.0094029	-1.25	RBM6	RNA binding motif protein 6
**1558740_s_at**	0.0152698	-1.25	LOC105369662	Uncharacterized LOC105369662
**1569315_s_at**	0.0095746	-1.25	LINC00894	Long intergenic non-protein coding RNA 894
**200602_at**	0.025362	-1.25	APP	Amyloid beta precursor protein
**201517_at**	0.0305758	-1.25	NCBP2	Nuclear cap binding protein subunit 2
**202582_s_at**	0.0120605	-1.25	RANBP9	RAN binding protein 9
**203856_at**	0.0331567	-1.25	VRK1	Vaccinia related kinase 1
**204142_at**	0.0018758	-1.25	ENOSF1	Enolase superfamily member 1

EXCLI Journal 2021;20:126-141– ISSN 1611-2156
Received: December 08, 2020, accepted: January 11, 2021, published: January 18, 2021

**209412_at**	0.035207	-1.25	LOC102724200///TRAPPC10	Trafficking protein particle complex subunit 10-like///trafficking protein particle complex 10
**209625_at**	0.0227215	-1.25	PIGH	Phosphatidylinositol glycan anchor biosynthesis class H
**209798_at**	0.0183383	-1.25	NPAT	Nuclear protein, coactivator of histone transcription
**213596_at**	0.0214082	-1.25	CASP4	Caspase 4
**213626_at**	0.0118595	-1.25	CBR4	Carbonyl reductase 4
**214048_at**	0.0258607	-1.25	MBD4	Methyl-cpg binding domain 4, DNA glycosylase
**218350_s_at**	0.0098839	-1.25	GMNN	Geminin, DNA replication inhibitor
**218840_s_at**	0.0481363	-1.25	NADSYN1	NAD synthetase 1
**219595_at**	0.0087906	-1.25	ZNF26	Zinc finger protein 26
**219999_at**	0.0114402	-1.25	MAN2A2	Mannosidase alpha class 2A member 2
**220643_s_at**	0.0256304	-1.25	FAIM	Fas apoptotic inhibitory molecule
**220720_x_at**	0.0262727	-1.25	MZT2B	Mitotic spindle organizing protein 2B
**221963_x_at**	0.0193243	-1.25	ZNF587B	Zinc finger protein 587B
**226126_at**	0.0361725	-1.25	TBCK	TBC1 domain containing kinase
**228377_at**	0.0320705	-1.25	KLHL14	Kelch like family member 14
**228384_s_at**	0.0030368	-1.25	PYROXD2	Pyridine nucleotide-disulphide oxidoreductase domain 2
**228930_at**	0.0142222	-1.25	SCARNA15	Small Cajal body-specific RNA 15
**231500_s_at**	0.0125079	-1.25	BOLA2	Bola family member 2
**236668_at**	0.0281143	-1.25	LOC101926967	Uncharacterized LOC101926967
**239283_at**	0.0398721	-1.25	TMED5	Transmembrane p24 trafficking protein 5
**200995_at**	0.0244306	-1.24	IPO7	Importin 7
**201025_at**	0.0177786	-1.24	EIF5B	Eukaryotic translation initiation factor 5B
**208072_s_at**	0.0409816	-1.24	DGKD	Diacylglycerol kinase delta
**209433_s_at**	0.0392061	-1.24	PPAT	Phosphoribosyl pyrophosphate amidotransferase
**212508_at**	0.0446691	-1.24	MOAP1	Modulator of apoptosis 1
**215597_x_at**	0.0356364	-1.24	LOC101929165	Uncharacterized LOC101929165
**216110_x_at**	0.0036803	-1.24	LOC105373738	Uncharacterized LOC105373738
**217936_at**	0.0350714	-1.24	ARHGAP5	Rho gtpase activating protein 5
**217979_at**	0.0137781	-1.24	TSPAN13	Tetraspanin 13
**219443_at**	0.0067773	-1.24	TASP1	Taspase 1
**226496_at**	0.0099204	-1.24	ZCCHC7	Zinc finger CCHC-type containing 7
**228391_at**	0.0269628	-1.24	CYP4V2	Cytochrome P450 family 4 subfamily V member 2
**236754_at**	0.0465756	-1.24	PPP1R2	Protein phosphatase 1 regulatory inhibitor subunit 2

EXCLI Journal 2021;20:126-141– ISSN 1611-2156
Received: December 08, 2020, accepted: January 11, 2021, published: January 18, 2021

**242711_x_at**	0.0241324	-1.24	FANCM	Fanconi anemia complementation group M
**1562364_at**	0.0026054	-1.23	GVINP1	Gtpase, very large interferon inducible pseudogene 1
**202789_at**	0.0403152	-1.23	PLCG1	Phospholipase C gamma 1
**204278_s_at**	0.0488824	-1.23	EBAG9	Estrogen receptor binding site associated, antigen, 9
**204333_s_at**	0.0128957	-1.23	AGA	Aspartylglucosaminidase
**204977_at**	0.0085261	-1.23	DDX10	DEAD-box helicase 10
**205251_at**	0.0340757	-1.23	PER2	Period circadian clock 2
**205316_at**	0.0126653	-1.23	SLC15A2	Solute carrier family 15 member 2
**206205_at**	0.018155	-1.23	MPHOSPH 9	M-phase phosphoprotein 9
**212594_at**	0.0243631	-1.23	MIR4680/// PDCD4	Microrna 4680///programmed cell death 4 (neoplastic transformation inhibitor)
**212855_at**	0.0418179	-1.23	DCUN1D4	Defective in cullin neddylation 1 domain containing 4
**221193_s_at**	0.042039	-1.23	ZCCHC10	Zinc finger CCHC-type containing 10
**224320_s_at**	0.0357952	-1.23	MCM8	Minichromosome maintenance 8 homologous recombination repair factor
**226873_at**	0.0266205	-1.23	FAM63B	Family with sequence similarity 63 member B
**227224_at**	0.011105	-1.23	RALGPS2	Ral GEF with PH domain and SH3 binding motif 2
**227896_at**	0.0026224	-1.23	BCCIP	BRCA2 and CDKN1A interacting protein
**236616_at**	0.0305948	-1.23	PSD4	Pleckstrin and Sec7 domain containing 4
**242195_x_at**	0.0181628	-1.23	NUMBL	NUMB like, endocytic adaptor protein
**244046_at**	0.0430938	-1.23	URGCP	Upregulator of cell proliferation
**244811_at**	0.0181646	-1.23	PHIP	Pleckstrin homology domain interacting protein
**1557260_a_at**	0.0346305	-1.22	ZNF382	Zinc finger protein 382
**209603_at**	0.0261616	-1.22	GATA3	GATA binding protein 3
**214918_at**	0.0479085	-1.22	HNRNPM	Heterogeneous nuclear ribonucleoprotein M
**216211_at**	0.0094559	-1.22	FAM208B	Family with sequence similarity 208 member B
**220750_s_at**	0.0151636	-1.22	P3H1	Prolyl 3-hydroxylase 1
**221738_at**	0.002375	-1.22	RALGAPB	Ral gtpase activating protein non-catalytic beta subunit
**222010_at**	0.0279597	-1.22	SNORA29/// TCP1	Small nucleolar RNA, H/ACA box 29///t-complex 1
**225164_s_at**	0.0346666	-1.22	EIF2AK4	Eukaryotic translation initiation factor 2 alpha kinase 4
**229433_at**	0.047414	-1.22	RBM26	RNA binding motif protein 26

EXCLI Journal 2021;20:126-141– ISSN 1611-2156
Received: December 08, 2020, accepted: January 11, 2021, published: January 18, 2021

**229501_s_at**	0.0201382	-1.22	USP8	Ubiquitin specific peptidase 8
**230871_at**	0.0149796	-1.22	DHX30	DEAH-box helicase 30
**232038_at**	0.042655	-1.22	TBC1D32	TBC1 domain family member 32
**235200_at**	0.0205135	-1.22	ZNF561///ZNF562	Zinc finger protein 561///zinc finger protein 562
**237747_at**	0.0030754	-1.22	LOC100507281	Uncharacterized LOC100507281
**1557953_at**	0.0250069	-1.21	ZKSCAN1	Zinc finger with KRAB and SCAN domains 1
**1562697_at**	0.0135194	-1.21	LOC339988	Uncharacterized LOC339988
**201027_s_at**	0.0256675	-1.21	EIF5B	Eukaryotic translation initiation factor 5B
**201693_s_at**	0.0476065	-1.21	EGR1	Early growth response 1
**208913_at**	0.0263149	-1.21	GGA2	Golgi associated, gamma adaptin ear containing, ARF binding protein 2
**212238_at**	0.0269346	-1.21	ASXL1	Additional sex combs like 1, transcriptional regulator
**215171_s_at**	0.0416277	-1.21	TIMM17A	Translocase of inner mitochondrial membrane 17 homolog A (yeast)
**219482_at**	0.0062833	-1.21	SETD4	SET domain containing 4
**222412_s_at**	0.0496369	-1.21	SSR3	Signal sequence receptor subunit 3
**226085_at**	0.0336811	-1.21	CBX5	Chromobox 5
**226339_at**	0.0228656	-1.21	TRUB1	Trub pseudouridine synthase family member 1
**226687_at**	0.0478383	-1.21	PRPF40A	Pre-mrna processing factor 40 homolog A
**226760_at**	0.0119559	-1.21	MBTPS2	Membrane bound transcription factor peptidase, site 2
**227900_at**	0.0226451	-1.21	CBLB	Cbl proto-oncogene B
**228209_at**	0.0211065	-1.21	LHX4-AS1	LHX4 antisense RNA 1
**229980_s_at**	0.0334074	-1.21	SNX5	Sorting nexin 5
**234970_at**	0.0138809	-1.21	TC2N	Tandem C2 domains, nuclear
**235697_at**	0.0148847	-1.21	ZNF544	Zinc finger protein 544
**243968_x_at**	0.0077583	-1.21	FCRL1	Fc receptor like 1
**1552283_s_at**	0.0217715	-1.2	ZDHHC11B///ZDHHC11	Zinc finger DHHC-type containing 11B///zinc finger DHHC-type containing 11
**1556203_a_at**	0.0200246	-1.2	SRGAP2	SLIT-ROBO Rho gtpase activating protein 2
**1556472_s_at**	0.0440985	-1.2	SCML4	Sex comb on midleg-like 4 (Drosophila)
**1557586_s_at**	0.0114967	-1.2	ATP6V1H	Atpase H+ transporting V1 subunit H
**1562238_at**	0.0284525	-1.2	USPL1	Ubiquitin specific peptidase like 1
**200845_s_at**	0.0174333	-1.2	PRDX6	Peroxiredoxin 6
**201501_s_at**	0.0165887	-1.2	GRSF1	G-rich RNA sequence binding factor 1

EXCLI Journal 2021;20:126-141– ISSN 1611-2156
Received: December 08, 2020, accepted: January 11, 2021, published: January 18, 2021

**201873_s_at**	0.0433641	-1.2	ABCE1	ATP binding cassette subfamily E member 1
**202683_s_at**	0.0429711	-1.2	RNMT	RNA guanine-7 methyltransferase
**213226_at**	0.0080485	-1.2	CCNA2	Cyclin A2
**213538_at**	0.0289495	-1.2	SON	SON DNA binding protein
**215155_at**	0.0051625	-1.2	HEXA	Hexosaminidase subunit alpha
**215947_s_at**	0.0447106	-1.2	LOC100287852///FAM136A	Protein FAM136A pseudo-gene///family with sequence similarity 136 member A
**219324_at**	0.0221187	-1.2	NOL12	Nucleolar protein 12
**219817_at**	0.0359601	-1.2	MAP-KAPK5-AS1	MAPKAPK5 antisense RNA 1
**221239_s_at**	0.0133673	-1.2	FCRL2	Fc receptor like 2
**223434_at**	0.0248267	-1.2	GBP3	Guanylate binding protein 3
**226555_at**	0.0260671	-1.2	INO80D	INO80 complex subunit D
**229642_at**	0.0246408	-1.2	ARHGEF7	Rho guanine nucleotide exchange factor 7
**235536_at**	0.0282488	-1.2	SNORD89	Small nucleolar RNA, C/D box 89
**238041_at**	0.0443798	-1.2	TCF12	Transcription factor 12
**241472_at**	0.0396311	-1.2	DMXL1	Dmx like 1
**1554248_at**	0.0200189	-1.19	ZNF638	Zinc finger protein 638
**1563497_at**	0.0129008	-1.19	USP25	Ubiquitin specific peptidase 25
**212771_at**	0.0039562	-1.19	FAM171A1	Family with sequence similarity 171 member A1
**212893_at**	0.044639	-1.19	ZZZ3	Zinc finger ZZ-type containing 3
**214059_at**	0.0428067	-1.19	IFI44	Interferon induced protein 44
**227284_at**	0.036748	-1.19	ZNF766	Zinc finger protein 766
**227646_at**	0.0421318	-1.19	EBF1	Early B-cell factor 1
**227839_at**	0.0156003	-1.19	MBD5	Methyl-cpg binding domain protein 5
**228239_at**	0.0367676	-1.19	SMIM11A	Small integral membrane protein 11A
**228570_at**	0.0297633	-1.19	BTBD11	BTB domain containing 11
**228594_at**	0.0386087	-1.19	NADK2	NAD kinase 2, mitochondrial
**229903_x_at**	0.0208178	-1.19	RNPC3	RNA binding region (RNP1, RRM) containing 3
**236134_at**	0.0393184	-1.19	DCAF7	DDB1 and CUL4 associated factor 7
**236266_at**	0.0348106	-1.19	RORA	RAR related orphan receptor A
**241925_x_at**	0.0262583	-1.19	SLC16A7	Solute carrier family 16 member 7
**204274_at**	0.0080239	-1.18	EBAG9	Estrogen receptor binding site associated, antigen, 9
**204831_at**	0.029874	-1.18	CDK8	Cyclin dependent kinase 8
**206214_at**	0.0211402	-1.18	PLA2G7	Phospholipase A2 group VII
**210018_x_at**	0.0075467	-1.18	MALT1	MALT1 paracaspase
**212653_s_at**	0.026392	-1.18	EHBP1	EH domain binding protein 1
**213956_at**	0.0343108	-1.18	CEP350	Centrosomal protein 350

EXCLI Journal 2021;20:126-141– ISSN 1611-2156
Received: December 08, 2020, accepted: January 11, 2021, published: January 18, 2021

**214719_at**	0.004316	-1.18	SLC46A3	Solute carrier family 46 member 3
**214735_at**	0.0402797	-1.18	IPCEF1	Interaction protein for cytohesin exchange factors 1
**214751_at**	0.0376954	-1.18	ZNF468	Zinc finger protein 468
**221781_s_at**	0.0311516	-1.18	DNAJC10	Dnaj heat shock protein family (Hsp40) member C10
**222541_at**	0.0082683	-1.18	RSF1	Remodeling and spacing factor 1
**222785_x_at**	0.0261502	-1.18	C11orf1	Chromosome 11 open reading frame 1
**225439_at**	0.0180875	-1.18	NUDCD1	Nudc domain containing 1
**226003_at**	0.0377238	-1.18	KIF21A	Kinesin family member 21A
**227085_at**	0.0189354	-1.18	H2AFV	H2A histone family member V
**227177_at**	0.0048276	-1.18	CORO2A	Coronin 2A
**228920_at**	0.0273654	-1.18	ZNF260	Zinc finger protein 260
**229872_s_at**	0.0296786	-1.18	LOC100996740	Uncharacterized LOC100996740
**59375_at**	0.0476783	-1.18	MYO15B	Myosin XVB
**1557239_at**	0.03574	-1.17	BBX	BBX, HMG-box containing
**1558792_x_at**	0.0084119	-1.17	AP2A1	Adaptor related protein complex 2 alpha 1 subunit
**205027_s_at**	0.0461708	-1.17	MAP3K8	Mitogen-activated protein kinase kinase kinase 8
**205728_at**	0.0481087	-1.17	TENM1	Teneurin transmembrane protein 1
**205760_s_at**	0.0398893	-1.17	OGG1	8-oxoguanine DNA glycosylase
**207130_at**	0.0493031	-1.17	ZMYND8	Zinc finger MYND-type containing 8
**207563_s_at**	0.0292065	-1.17	OGT	O-linked N-acetylglucosamine (glcnac) transferase
**212984_at**	0.0352315	-1.17	ATF2	Activating transcription factor 2
**214787_at**	0.036017	-1.17	DENND4A	DENN domain containing 4A
**220985_s_at**	0.0399878	-1.17	RNF170	Ring finger protein 170
**222360_at**	0.0034929	-1.17	DPH5	Diphthamide biosynthesis 5
**225191_at**	0.0396481	-1.17	CIRBP	Cold inducible RNA binding protein
**225445_at**	0.0286878	-1.17	UBN2	Ubinuclein 2
**226604_at**	0.0075895	-1.17	TMTC3	Transmembrane and tetratricopeptide repeat containing 3
**226773_at**	0.0383668	-1.17	PPM1K	Protein phosphatase, Mg2+/Mn2+ dependent 1K
**229268_at**	0.0233929	-1.17	OTULIN	OTU deubiquitinase with linear linkage specificity
**229371_at**	0.0115339	-1.17	SLC20A2	Solute carrier family 20 member 2
**230211_at**	0.021938	-1.17	TRIP11	Thyroid hormone receptor interactor 11
**232362_at**	0.0097878	-1.17	CCDC18	Coiled-coil domain containing 18
**233255_s_at**	0.0284174	-1.17	BIVM	Basic, immunoglobulin-like variable motif containing
**235132_at**	0.0206393	-1.17	NIFK-AS1	NIFK antisense RNA 1

**236700_at**	0.0123245	-1.17	EIF3C	Eukaryotic translation initiation factor 3 subunit C
**37860_at**	0.0098264	-1.17	ZNF337	Zinc finger protein 337
**1552862_at**	0.0030911	-1.16	RUSC1-AS1	RUSC1 antisense RNA 1
**1560659_at**	0.0234307	-1.16	LINC00342	Long intergenic non-protein coding RNA 342
**206500_s_at**	0.0330486	-1.16	MIS18BP1	MIS18 binding protein 1
**212977_at**	0.015451	-1.16	ACKR3	Atypical chemokine receptor 3
**213340_s_at**	0.0268121	-1.16	TP73-AS1	TP73 antisense RNA 1
**213857_s_at**	0.0064515	-1.16	CD47	CD47 molecule
**214030_at**	0.0401257	-1.16	CRYBG3	Crystallin beta-gamma domain containing 3
**214594_x_at**	0.0067646	-1.16	ATP8B1	Atpase phospholipid transporting 8B1
**216711_s_at**	0.0265686	-1.16	TAF1	TATA-box binding protein associated factor 1
**218621_at**	0.0152548	-1.16	HEMK1	Hemk methyltransferase family member 1
**218642_s_at**	0.0204299	-1.16	CHCHD7	Coiled-coil-helix-coiled-coil-helix domain containing 7
**220071_x_at**	0.0174232	-1.16	HAUS2	HAUS augmin like complex subunit 2
**223161_at**	0.0066686	-1.16	KIAA1147	Kiaa1147
**223238_s_at**	0.0403954	-1.16	PBRM1	Polybromo 1
**223424_s_at**	0.0230673	-1.16	ZSCAN21	Zinc finger and SCAN domain containing 21
**226031_at**	0.0199303	-1.16	VPS50	VPS50, EARP/GARPII complex subunit
**226150_at**	0.0446873	-1.16	PLPP5	Phospholipid phosphatase 5
**227456_s_at**	0.0279994	-1.16	C6orf136	Chromosome 6 open reading frame 136
**228144_at**	0.0212639	-1.16	ZNF300	Zinc finger protein 300
**228386_s_at**	0.0270782	-1.16	DDX59	DEAD-box helicase 59
**228476_at**	0.0064896	-1.16	CCDC191	Coiled-coil domain containing 191
**228630_at**	0.0309133	-1.16	ZNF84	Zinc finger protein 84
**229173_at**	0.0326549	-1.16	LNPK	Lunapark, ER junction formation factor
**234297_at**	0.0383683	-1.16	SDHAP3///RGS8	Succinate dehydrogenase complex flavoprotein subunit A pseudogene 3///regulator of G-protein signaling 8
**243649_at**	0.0434877	-1.16	FBXO7	F-box protein 7
**1554999_at**	0.0081473	-1.15	RASGEF1B	Rasgef domain family member 1B
**1559739_at**	0.0279994	-1.15	CHPT1	Choline phosphotransferase 1
**200893_at**	0.0413222	-1.15	TRA2B	Transformer 2 beta homolog (Drosophila)
**203517_at**	0.0209575	-1.15	MTX2	Metaxin 2

EXCLI Journal 2021;20:126-141– ISSN 1611-2156
Received: December 08, 2020, accepted: January 11, 2021, published: January 18, 2021

**203688_at**	0.0362252	-1.15	PKD2	Polycystin 2, transient receptor potential cation channel
**206478_at**	0.0358711	-1.15	FAM30A	Family with sequence similarity 30, member A
**206860_s_at**	0.0370589	-1.15	MIOS	Meiosis regulator for oocyte development
**210306_at**	0.031828	-1.15	L3MBTL1	L(3)mbt-like 1 (Drosophila)
**212898_at**	0.0316359	-1.15	TTI1	TELO2 interacting protein 1
**213269_at**	0.0352554	-1.15	ZNF248	Zinc finger protein 248
**218721_s_at**	0.0133579	-1.15	C1orf27	Chromosome 1 open reading frame 27
**218957_s_at**	0.0378134	-1.15	PAAF1	Proteasomal atpase associated factor 1
**219648_at**	0.020133	-1.15	MREG	Melanoregulin
**222023_at**	0.0388775	-1.15	AKAP13	A-kinase anchoring protein 13
**223119_s_at**	0.0291846	-1.15	USP47	Ubiquitin specific peptidase 47
**223506_at**	0.0117358	-1.15	ZC3H8	Zinc finger CCCH-type containing 8
**229491_at**	0.0082209	-1.15	SLC9B2	Solute carrier family 9 member B2
**230776_at**	0.0231657	-1.15	RNF157-AS1	RNF157 antisense RNA 1
**231530_s_at**	0.0435773	-1.15	C11orf1	Chromosome 11 open reading frame 1
**239143_x_at**	0.0401621	-1.15	RNF138	Ring finger protein 138
**239434_at**	0.0191633	-1.15	FGFR1OP	FGFR1 oncogene partner
**243759_at**	0.0236449	-1.15	SCAF4	SR-related CTD associated factor 4
**1553909_x_at**	0.0361553	-1.14	SLF2	SMC5-SMC6 complex localization factor 2
**1558560_s_at**	0.0159968	-1.14	BLZF1	Basic leucine zipper nuclear factor 1
**1560661_x_at**	0.0108714	-1.14	LINC00342	Long intergenic non-protein coding RNA 342
**205308_at**	0.0341661	-1.14	ZC2HC1A	Zinc finger C2HC-type containing 1A
**209146_at**	0.0243181	-1.14	MSMO1	Methylsterol monooxygenase 1
**211575_s_at**	0.0101873	-1.14	UBE3A	Ubiquitin protein ligase E3A
**212193_s_at**	0.0297528	-1.14	LARP1	La ribonucleoprotein domain family member 1
**213620_s_at**	0.0478643	-1.14	ICAM2	Intercellular adhesion molecule 2
**213700_s_at**	0.0323774	-1.14	PKM	Pyruvate kinase, muscle
**214140_at**	0.012051	-1.14	SLC25A16	Solute carrier family 25 member 16
**217665_at**	0.0208619	-1.14	ZNF813	Zinc finger protein 813
**220350_at**	0.0201752	-1.14	ZNF235	Zinc finger protein 235
**224231_at**	0.0104734	-1.14	PRO0471	Uncharacterized LOC28994
**228320_x_at**	0.0272271	-1.14	BICDL1	BICD family like cargo adaptor 1
**229053_at**	0.0346863	-1.14	SYT17	Synaptotagmin 17
**230110_at**	0.0383839	-1.14	MCOLN2	Mucolipin 2

**232309_at**	0.0299833	-1.14	LOC202181	SUMO interacting motifs containing 1 pseudogene
**1559214_at**	0.0148845	-1.13	FBXO22	F-box protein 22
**202813_at**	0.0437325	-1.13	TARBP1	TAR (HIV-1) RNA binding protein 1
**205297_s_at**	0.03088	-1.13	CD79B	CD79b molecule
**207023_x_at**	0.0402922	-1.13	KRT10	Keratin 10
**209407_s_at**	0.0481526	-1.13	DEAF1	DEAF1, transcription factor
**220081_x_at**	0.0247217	-1.13	HSD17B7	Hydroxysteroid 17-beta dehydro-genase 7
**221020_s_at**	0.0481253	-1.13	SLC25A32	Solute carrier family 25 member 32
**226105_at**	0.0456518	-1.13	C1GALT1	Core 1 synthase, glycoprotein-N-acetylgalactosamine 3-beta-galac-tosyltransferase 1
**226441_at**	0.0071216	-1.13	MAP3K2	Mitogen-activated protein kinase ki-nase kinase 2
**226784_at**	0.038007	-1.13	TWISTNB	TWIST neighbor
**228062_at**	0.0313856	-1.13	NAP1L5	Nucleosome assembly protein 1 like 5
**236196_at**	0.0475089	-1.13	ZNF326	Zinc finger protein 326
**238078_at**	0.0111086	-1.13	SEC22A	SEC22 homolog A, vesicle traffick-ing protein
**241704_x_at**	0.0089535	-1.13	ZNF320	Zinc finger protein 320
**241803_s_at**	0.0487146	-1.13	LOC102723709///LOC102723678///LOC101928102	Uncharacterized LOC102723709///uncharacterized LOC102723678///putative unchar-acterized protein LOC401522-like
**202051_s_at**	0.0385024	-1.12	ZMYM4	Zinc finger MYM-type containing 4
**202600_s_at**	0.0283053	-1.12	NRIP1	Nuclear receptor interacting protein 1
**214060_at**	0.0216844	-1.12	SSBP1	Single stranded DNA binding pro-tein 1
**214255_at**	0.0429134	-1.12	ATP10A	Atpase phospholipid transporting 10A (putative)
**221206_at**	0.0259618	-1.12	PMS2CL/// PMS2	PMS2 C-terminal like pseudo-gene///PMS1 homolog 2, mismatch repair system component
**222478_at**	0.0363878	-1.12	VPS36	Vacuolar protein sorting 36 homo-log
**225066_at**	0.0215928	-1.12	PPP2R2D	Protein phosphatase 2 regulatory subunit Bdelta
**225501_at**	0.0464898	-1.12	PHF6	PHD finger protein 6
**225704_at**	0.0140452	-1.12	FBRSL1	Fibrosin like 1
**226501_at**	0.0102192	-1.12	XPNPEP3	X-prolyl aminopeptidase 3
**226858_at**	0.0059903	-1.12	CSNK1E	Casein kinase 1 epsilon
**229204_at**	0.0233689	-1.12	HP1BP3	Heterochromatin protein 1 binding protein 3
**230777_s_at**	0.0261656	-1.12	PRDM15	PR/SET domain 15
**231697_s_at**	0.0398934	-1.12	MIR21///VMP1	Microrna 21///vacuole membrane protein 1

**232589_at**	0.0114938	-1.12	LOC102606465	Uncharacterized LOC102606465
**233078_at**	0.01264	-1.12	API5	Apoptosis inhibitor 5
**244669_at**	0.0261427	-1.12	SNORD50B///SNORD50A	Small nucleolar RNA, C/D box 50B///small nucleolar RNA, C/D box 50A
**1554549_a_at**	0.0339174	-1.11	WDR20	WD repeat domain 20
**1560156_at**	0.0247674	-1.11	LOC101928054	Uncharacterized LOC101928054
**1565716_at**	0.0365175	-1.11	FUS	FUS RNA binding protein
**202450_s_at**	0.0226492	-1.11	CTSK	Cathepsin K
**202768_at**	0.0432779	-1.11	FOSB	Fosb proto-oncogene, AP-1 transcription factor subunit
**202973_x_at**	0.0197352	-1.11	FAM13A	Family with sequence similarity 13 member A
**204202_at**	0.0056356	-1.11	IQCE	IQ motif containing E
**205427_at**	0.0089595	-1.11	ZNF354A	Zinc finger protein 354A
**210109_at**	0.0494978	-1.11	SND1-IT1	SND1 intronic transcript 1
**210111_s_at**	0.0369146	-1.11	KLHDC10	Kelch domain containing 10
**214183_s_at**	0.0177377	-1.11	TKTL1	Transketolase like 1
**216748_at**	0.033145	-1.11	PYHIN1	Pyrin and HIN domain family member 1
**218437_s_at**	0.0434938	-1.11	LZTFL1	Leucine zipper transcription factor like 1
**218592_s_at**	0.034826	-1.11	CECR5	Cat eye syndrome chromosome region, candidate 5
**221616_s_at**	0.0037647	-1.11	TAF9B	TATA-box binding protein associated factor 9b
**223477_s_at**	0.0236485	-1.11	C12orf65	Chromosome 12 open reading frame 65
**223598_at**	0.0210857	-1.11	RAD23B	RAD23 homolog B, nucleotide excision repair protein
**226300_at**	0.0380668	-1.11	MED19	Mediator complex subunit 19
**227605_at**	0.0385532	-1.11	AIMP1	Aminoacyl trna synthetase complex interacting multifunctional protein 1
**228336_at**	0.0392272	-1.11	PWWP2A	PWWP domain containing 2A
**228351_at**	0.0229548	-1.11	HEATR1	HEAT repeat containing 1
**230005_at**	0.0060018	-1.11	SVIP	Small VCP interacting protein
**230790_x_at**	0.0073796	-1.11	FOXN3	Forkhead box N3
**235290_at**	0.0241063	-1.11	ZNF782	Zinc finger protein 782
**235365_at**	0.0217514	-1.11	DFNB59	Deafness, autosomal recessive 59
**235511_at**	0.0243691	-1.11	RBM14-RBM4///RBM4	RBM14-RBM4 readthrough///RNA binding motif protein 4
**236808_at**	0.0095941	-1.11	FGFR1OP2	FGFR1 oncogene partner 2
**239654_at**	0.0168235	-1.11	CHD9	Chromodomain helicase DNA binding protein 9
**243985_at**	0.0443679	-1.11	GTF2A2	General transcription factor IIA subunit 2

EXCLI Journal 2021;20:126-141– ISSN 1611-2156
Received: December 08, 2020, accepted: January 11, 2021, published: January 18, 2021

**1552307_a_at**	0.0128216	-1.1	TTC39C	Tetratricopeptide repeat domain 39C
**1554487_a_at**	0.036763	-1.1	ATF6B	Activating transcription factor 6 beta
**1555864_s_at**	0.0333371	-1.1	PDHA1	Pyruvate dehydrogenase (lipoam-ide) alpha 1
**1557477_at**	0.0207179	-1.1	STIM1	Stromal interaction molecule 1
**201936_s_at**	0.0243265	-1.1	EIF4G3	Eukaryotic translation initiation fac-tor 4 gamma 3
**202114_at**	0.009549	-1.1	SNX2	Sorting nexin 2
**210530_s_at**	0.0474315	-1.1	NR2C1	Nuclear receptor subfamily 2 group C member 1
**211569_s_at**	0.0451227	-1.1	HADH	Hydroxyacyl-coa dehydrogenase
**213203_at**	0.0121049	-1.1	SNAPC5	Small nuclear RNA activating com-plex polypeptide 5
**217892_s_at**	0.0299688	-1.1	LIMA1	LIM domain and actin binding 1
**218193_s_at**	0.0078392	-1.1	GOLT1B	Golgi transport 1B
**218256_s_at**	0.0232885	-1.1	NUP54	Nucleoporin 54
**218285_s_at**	0.0356355	-1.1	BDH2	3-hydroxybutyrate dehydrogenase, type 2
**218476_at**	0.0220483	-1.1	POMT1	Protein O-mannosyltransferase 1
**219353_at**	0.0131079	-1.1	NHLRC2	NHL repeat containing 2
**220078_at**	0.0143584	-1.1	USP48	Ubiquitin specific peptidase 48
**222286_at**	0.0418224	-1.1	SNAPC3	Small nuclear RNA activating com-plex polypeptide 3
**222524_s_at**	0.0201189	-1.1	ASB6	Ankyrin repeat and SOCS box con-taining 6
**225172_at**	0.0307984	-1.1	CRAMP1	Cramped chromatin regulator hom-olog 1
**228834_at**	0.0440074	-1.1	TOB1	Transducer of ERBB2, 1
**229267_at**	0.0048235	-1.1	LOC730268 ///ANAPC1	Anaphase-promoting complex sub-unit 1-like///anaphase promoting complex subunit 1
**229533_x_at**	0.0221633	-1.1	ZNF680	Zinc finger protein 680
**229955_at**	0.0469344	-1.1	FBXO3	F-box protein 3
**230350_at**	0.0483992	-1.1	TIAL1	TIA1 cytotoxic granule-associated RNA binding protein-like 1
**234923_at**	0.0418418	-1.1	RALGAPA1	Ral gtpase activating protein cata-lytic alpha subunit 1
**235032_at**	0.0160464	-1.1	DNAJC21	Dnaj heat shock protein family (Hsp40) member C21
**1556467_at**	0.0481043	-1.09	LOC101929 734	Uncharacterized LOC101929734
**1557036_at**	0.0184682	-1.09	ZBTB1	Zinc finger and BTB domain con-taining 1
**200069_at**	0.03398	-1.09	SART3	Squamous cell carcinoma antigen recognized by T-cells 3
**203285_s_at**	0.045459	-1.09	HS2ST1	Heparan sulfate 2-O-sulfotransfer-ase 1

EXCLI Journal 2021;20:126-141– ISSN 1611-2156
Received: December 08, 2020, accepted: January 11, 2021, published: January 18, 2021

**204376_at**	0.0275407	-1.09	DCAF1	DDB1 and CUL4 associated factor 1
