# Supplementary material for: Common differentially expressed genes and pathways correlating both coronary artery disease and atrial fibrillation
Source: EXCLI J. 2021 Jan 18;20:126–41. doi: 10.17179/excli2020-3262 (PMC7868642; doi:10.17179/excli2020-3262)
Supplement: Supplementary table 3 [file EXCLI-20-126-s-002.pdf]

EXCLI Journal 2021;20:126-141– ISSN 1611-2156
Received: December 08, 2020, accepted: January 11, 2021, published: January 18, 2021

Supplementary material to:

Original article:

COMMON DIFFERENTIALLY EXPRESSED GENES AND PATHWAYS CORRELATING BOTH CORONARY ARTERY DISEASE AND ATRIAL FIBRILLATION

Youjing Zheng, Jia-Qiang He*

Department of Biomedical Sciences and Pathobiology, College of Veterinary Medicine, Virginia Tech, Blacksburg, VA 24061, USA

* **Corresponding author:** Jia-Qiang He, Department of Biomedical Sciences and Pathobiology, Virginia Tech, Phase II, Room 252B, Blacksburg, VA 24061, USA. Tel: 1-540-231-2032. E-mail: jiahe@vt.edu

https://orcid.org/0000-0002-4825-7046 Youjing Zheng
https://orcid.org/0000-0002-0640-5960 Jia-Qiang He

http://dx.doi.org/10.17179/excli2020-3262

This is an Open Access article distributed under the terms of the Creative Commons Attribution License (http://creativecommons.org/licenses/by/4.0/).

Supplemental Table 3: DEGs in GSE31821 dataset. Up- and down-regulated genes are on page S1-S11 and page S11-S13, respectively.

293 up-regulated genes				
**ID**	**p value**	**logFC**	**Gene symbol**	**Gene title**
**212922_s_at**	0.0022946	3.71	SMYD2	SET and MYND domain containing 2
**203872_at**	0.0000227	3.43	ACTA1	Actin, alpha 1, skeletal muscle
**219789_at**	0.0020168	3.4	NPR3	Natriuretic peptide receptor 3
**203400_s_at**	0.0441469	2.86	TF	Transferrin
**219054_at**	0.0016136	2.84	NPR3	Natriuretic peptide receptor 3
**203032_s_at**	0.0193009	2.66	FH	Fumarate hydratase
**200831_s_at**	0.0383017	2.44	SCD	Stearoyl-coa desaturase
**215646_s_at**	0.0341907	2.43	VCAN	Versican
**201694_s_at**	0.0376352	2.39	EGR1	Early growth response 1
**214414_x_at**	0.0491523	2.37	HBA2///HBA1	Hemoglobin subunit alpha 2///hemoglobin subunit alpha 1
**227404_s_at**	0.0290381	2.34	EGR1	Early growth response 1
**205842_s_at**	0.0006616	2.26	JAK2	Janus kinase 2
**235978_at**	0.0361347	2.26	FABP4	Fatty acid binding protein 4

EXCLI Journal 2021;20:126-141– ISSN 1611-2156
Received: December 08, 2020, accepted: January 11, 2021, published: January 18, 2021

**211745_x_at**	0.041438	2.25	HBA2///HBA 1	Hemoglobin subunit alpha 2///hemo-globin subunit alpha 1
**203434_s_at**	0.0127464	2.24	MME	Membrane metallo-endopeptidase
**1557910_at**	0.0124068	2.23	HSP90AB1	Heat shock protein 90 alpha family class B member 1
**217414_x_at**	0.0439746	2.23	HBA2///HBA 1	Hemoglobin subunit alpha 2///hemo-globin subunit alpha 1
**221211_s_at**	0.0360466	2.18	MAP3K7CL	MAP3K7 C-terminal like
**1554333_at**	0.01725	2.17	DNAJA4	Dnaj heat shock protein family (Hsp40) member A4
**201539_s_at**	0.0149806	2.12	FHL1	Four and a half LIM domains 1
**209728_at**	0.0236568	2.12	HLA-DRB4	Major histocompatibility complex, class II, DR beta 4
**225207_at**	0.0322758	2.06	PDK4	Pyruvate dehydrogenase kinase 4
**225424_at**	0.0402638	2.06	GPAM	Glycerol-3-phosphate acyltransfer-ase, mitochondrial
**210764_s_at**	0.012417	2.03	CYR61	Cysteine rich angiogenic inducer 61
**221232_s_at**	0.0089973	2.02	ANKRD2	Ankyrin repeat domain 2
**1558093_s_at**	0.0042892	2.01	SNHG4///MATR3	Small nucleolar RNA host gene 4///matrin 3
**1553530_a_at**	0.0148517	2.01	ITGB1	Integrin subunit beta 1
**200806_s_at**	0.0313805	2	HSPD1	Heat shock protein family D (Hsp60) member 1
**202949_s_at**	0.0114128	1.99	FHL2	Four and a half LIM domains 2
**201852_x_at**	0.0058313	1.97	COL3A1	Collagen type III alpha 1 chain
**212190_at**	0.0435009	1.94	SERPINE2	Serpin family E member 2
**205841_at**	0.0031606	1.91	JAK2	Janus kinase 2
**203980_at**	0.0071416	1.91	FABP4	Fatty acid binding protein 4
**229128_s_at**	0.0330387	1.89	ANP32E	Acidic nuclear phosphoprotein 32 family member E
**228834_at**	0.0365102	1.89	TOB1	Transducer of ERBB2, 1
**205174_s_at**	0.0244375	1.88	QPCT	Glutaminyl-peptide cyclotransferase
**209458_x_at**	0.0251628	1.87	HBA2///HBA 1	Hemoglobin subunit alpha 2///hemo-globin subunit alpha 1
**211708_s_at**	0.019395	1.86	SCD	Stearoyl-coa desaturase
**209896_s_at**	0.0074092	1.84	PTPN11	Protein tyrosine phosphatase, non-receptor type 11
**205984_at**	0.0382338	1.83	CRHBP	Corticotropin releasing hormone binding protein
**211699_x_at**	0.016646	1.82	HBA2///HBA 1	Hemoglobin subunit alpha 2///hemo-globin subunit alpha 1
**210298_x_at**	0.0215877	1.8	FHL1	Four and a half LIM domains 1
**204507_s_at**	0.019323	1.79	PPP3R1	Protein phosphatase 3 regulatory subunit B, alpha
**203441_s_at**	0.0058951	1.77	CDH2	Cadherin 2
**216591_s_at**	0.0269054	1.77	SDHC	Succinate dehydrogenase complex subunit C
**211571_s_at**	0.0492984	1.77	VCAN	Versican

EXCLI Journal 2021;20:126-141– ISSN 1611-2156
Received: December 08, 2020, accepted: January 11, 2021, published: January 18, 2021

**225062_at**	0.0352272	1.75	LOC102724951///LOC389831	Uncharacterized LOC102724951///uncharacterized LOC389831
**210875_s_at**	0.012347	1.71	ZEB1	Zinc finger E-box binding homeobox 1
**202464_s_at**	0.0331309	1.71	PFKFB3	6-phosphofructo-2-kinase/fructose-2,6-biphosphatase 3
**201043_s_at**	0.0046583	1.68	ANP32A	Acidic nuclear phosphoprotein 32 family member A
**216178_x_at**	0.0163979	1.68	ITGB1	Integrin subunit beta 1
**222847_s_at**	0.0003567	1.67	EGLN3	Egl-9 family hypoxia inducible factor 3
**219873_at**	0.0000116	1.66	COLEC11	Collectin subfamily member 11
**243278_at**	0.0033824	1.66	FOXP2	Forkhead box P2
**206544_x_at**	0.044689	1.66	SMARCA2	SWI/SNF related, matrix associated, actin dependent regulator of chromatin, subfamily a, member 2
**209098_s_at**	0.007034	1.65	JAG1	Jagged 1
**227719_at**	0.0123784	1.65	SMAD9	SMAD family member 9
**214505_s_at**	0.0168999	1.65	FHL1	Four and a half LIM domains 1
**1557167_at**	0.0426313	1.65	HCG11	HLA complex group 11 (non-protein coding)
**204018_x_at**	0.0198528	1.64	HBA2///HBA1	Hemoglobin subunit alpha 2///hemoglobin subunit alpha 1
**213994_s_at**	0.0014241	1.63	SPON1	Spondin 1
**210338_s_at**	0.0052762	1.63	SNORD14D///SNORD14C///HSPA8	Small nucleolar RNA, C/D box 14D///small nucleolar RNA, C/D box 14C///heat shock protein family A (Hsp70) member 8
**214545_s_at**	0.0159598	1.63	PROSC	Proline synthetase cotranscribed homolog (bacterial)
**214040_s_at**	0.0310885	1.63	GSN	Gelsolin
**202566_s_at**	0.0062822	1.61	SVIL	Supervillin
**223341_s_at**	0.0083186	1.6	SCOC	Short coiled-coil protein
**204932_at**	0.0290526	1.6	TNFRSF11B	TNF receptor superfamily member 11b
**224582_s_at**	0.0452989	1.6	NUCKS1	Nuclear casein kinase and cyclin dependent kinase substrate 1
**200751_s_at**	0.0052483	1.59	HNRNPC	Heterogeneous nuclear ribonucleoprotein C (C1/C2)
**210299_s_at**	0.0145718	1.59	FHL1	Four and a half LIM domains 1
**212944_at**	0.0206235	1.59	SLC5A3	Solute carrier family 5 member 3
**215714_s_at**	0.0305269	1.59	SMARCA4	SWI/SNF related, matrix associated, actin dependent regulator of chromatin, subfamily a, member 4
**1560169_at**	0.0347219	1.59	LOC101927069	Uncharacterized LOC101927069
**213993_at**	0.0013132	1.58	SPON1	Spondin 1
**203811_s_at**	0.0194899	1.58	DNAJB4	Dnaj heat shock protein family (Hsp40) member B4

EXCLI Journal 2021;20:126-141– ISSN 1611-2156
Received: December 08, 2020, accepted: January 11, 2021, published: January 18, 2021

**219790_s_at**	0.0033515	1.57	NPR3	Natriuretic peptide receptor 3
**207069_s_at**	0.0075751	1.56	SMAD6	SMAD family member 6
**216521_s_at**	0.018236	1.56	BRCC3	BRCA1/BRCA2-containing complex subunit 3
**228284_at**	0.0087725	1.55	TLE1	Transducin like enhancer of split 1
**204933_s_at**	0.0091648	1.55	TNFRSF11 B	TNF receptor superfamily member 11b
**219929_s_at**	0.0368766	1.55	ZFYVE21	Zinc finger FYVE-type containing 21
**209257_s_at**	0.0134086	1.54	SMC3	Structural maintenance of chromo-somes 3
**226069_at**	0.0282802	1.54	PRICKLE1	Prickle planar cell polarity protein 1
**211966_at**	0.0178911	1.53	COL4A2	Collagen type IV alpha 2 chain
**215780_s_at**	0.0111222	1.52	SETSIP///SETP4///SET	SET-like protein///SET pseudogene 4///SET nuclear proto-oncogene
**202742_s_at**	0.0188211	1.52	PRKACB	Protein kinase camp-activated cata-lytic subunit beta
**228425_at**	0.003854	1.51	PAX8-AS1	PAX8 antisense RNA 1
**231918_s_at**	0.0064345	1.51	GFM2	G elongation factor mitochondrial 2
**216733_s_at**	0.0237287	1.51	GATM	Glycine amidinotransferase
**244287_at**	0.0092546	1.5	SREK1	Splicing regulatory glutamic acid and lysine rich protein 1
**212218_s_at**	0.0443936	1.5	FASN	Fatty acid synthase
**233496_s_at**	0.0002775	1.49	CFL2	Cofilin 2
**203548_s_at**	0.0003454	1.49	LPL	Lipoprotein lipase
**219326_s_at**	0.0004097	1.49	B3GNT2	UDP-glcnac:betagal beta-1,3-N-acetylglucosaminyltransferase 2
**1555814_a_at**	0.0332349	1.49	RHOA	Ras homolog family member A
**207332_s_at**	0.0240447	1.47	TFRC	Transferrin receptor
**1555543_a_at**	0.0454656	1.47	CLCC1	Chloride channel CLIC like 1
**201337_s_at**	0.0457033	1.47	VAMP3	Vesicle associated membrane pro-tein 3
**218780_at**	0.0039119	1.46	HOOK2	Hook microtubule tethering protein 2
**209576_at**	0.0071786	1.46	GNAI1	G protein subunit alpha i1
**215076_s_at**	0.0141133	1.46	COL3A1	Collagen type III alpha 1 chain
**226237_at**	0.0497092	1.46	COL8A1	Collagen type VIII alpha 1 chain
**205321_at**	0.0494234	1.45	EIF2S3	Eukaryotic translation initiation fac-tor 2 subunit gamma
**213537_at**	0.0284914	1.44	HLA-DPA1	Major histocompatibility complex, class II, DP alpha 1
**209436_at**	0.0010569	1.43	SPON1	Spondin 1
**1553678_a_at**	0.0140471	1.43	ITGB1	Integrin subunit beta 1
**242496_at**	0.014978	1.43	ART4	ADP-ribosyltransferase 4 (Dom-brock blood group)
**201490_s_at**	0.034949	1.43	PPIF	Peptidylprolyl isomerase F
**217073_x_at**	0.0362718	1.43	APOA1	Apolipoprotein A1

EXCLI Journal 2021;20:126-141– ISSN 1611-2156
Received: December 08, 2020, accepted: January 11, 2021, published: January 18, 2021

**207977_s_at**	0.0059655	1.42	DPT	Dermatopontin
**213135_at**	0.0365554	1.42	TIAM1	T-cell lymphoma invasion and metastasis 1
**222634_s_at**	0.0155621	1.41	TBL1XR1	Transducin (beta)-like 1 X-linked receptor 1
**207275_s_at**	0.0253533	1.41	ACSL1	Acyl-coa synthetase long-chain family member 1
**231715_s_at**	0.0318947	1.41	MIR6741///PYCR2	Microrna 6741///pyrroline-5-carboxylate reductase family member 2
**200665_s_at**	0.0021325	1.4	SPARC	Secreted protein acidic and cysteine rich
**214130_s_at**	0.0040313	1.4	LOC101930416///LOC101929792///LOC100996724///PDE4DIP	Myomegalin-like///uncharacterized LOC101929792///phosphodiesterase 4D interacting protein-like///phosphodiesterase 4D interacting protein
**214845_s_at**	0.0272061	1.4	CALU	Calumenin
**202672_s_at**	0.0280295	1.4	ATF3	Activating transcription factor 3
**214279_s_at**	0.0336867	1.39	NDRG2	NDRG family member 2
**225238_at**	0.0078435	1.38	MSI2	Musashi RNA binding protein 2
**208712_at**	0.0279907	1.38	CCND1	Cyclin D1
**211090_s_at**	0.0462614	1.38	PRPF4B	Pre-mrna processing factor 4B
**1552477_a_at**	0.0063529	1.37	IRF6	Interferon regulatory factor 6
**242715_at**	0.0208695	1.37	ZNF536	Zinc finger protein 536
**211922_s_at**	0.0271541	1.37	CAT	Catalase
**1570523_s_at**	0.0303162	1.37	ATG10	Autophagy related 10
**217140_s_at**	0.0352148	1.37	VDAC1	Voltage dependent anion channel 1
**1555154_a_at**	0.0016639	1.36	QKI	QKI, KH domain containing RNA binding
**228824_s_at**	0.0096583	1.36	PTGR1	Prostaglandin reductase 1
**201289_at**	0.0146091	1.36	CYR61	Cysteine rich angiogenic inducer 61
**232186_at**	0.0291146	1.36	FITM2	Fat storage inducing transmembrane protein 2
**1567458_s_at**	0.0476732	1.36	RAC1	Ras-related C3 botulinum toxin substrate 1 (rho family, small GTP binding protein Rac1)
**212143_s_at**	0.0038804	1.35	IGFBP3	Insulin like growth factor binding protein 3
**1558254_s_at**	0.0046558	1.35	SRPK2	SRSF protein kinase 2
**208853_s_at**	0.0056889	1.35	CANX	Calnexin
**1558027_s_at**	0.0059902	1.35	PRKAB2	Protein kinase AMP-activated non-catalytic subunit beta 2
**205512_s_at**	0.0183686	1.35	AIFM1	Apoptosis inducing factor, mitochondria associated 1
**208456_s_at**	0.0063489	1.34	RRAS2	Related RAS viral (r-ras) oncogene homolog 2

EXCLI Journal 2021;20:126-141– ISSN 1611-2156
Received: December 08, 2020, accepted: January 11, 2021, published: January 18, 2021

**211169_s_at**	0.0097277	1.34	PPP1R3A	Protein phosphatase 1 regulatory subunit 3A
**226834_at**	0.0131477	1.34	CLMP	CXADR like membrane protein
**212488_at**	0.041005	1.34	COL5A1	Collagen type V alpha 1 chain
**204427_s_at**	0.0470327	1.34	TMED2	Transmembrane p24 trafficking protein 2
**221730_at**	0.0008487	1.33	COL5A2	Collagen type V alpha 2 chain
**230360_at**	0.0009105	1.33	GLDN	Gliomedin
**242034_at**	0.0068197	1.33	FBXL17	F-box and leucine rich repeat protein 17
**211015_s_at**	0.0075559	1.33	HSPA4	Heat shock protein family A (Hsp70) member 4
**201496_x_at**	0.0278576	1.33	MYH11	Myosin heavy chain 11
**1558028_x_at**	0.0497851	1.33	NORAD	Non-coding RNA activated by DNA damage
**212724_at**	0.0006849	1.32	RND3	Rho family gtpase 3
**211651_s_at**	0.0014879	1.32	LAMB1	Laminin subunit beta 1
**203357_s_at**	0.028582	1.31	CAPN7	Calpain 7
**233814_at**	0.0292444	1.31	EFNA5	Ephrin A5
**217562_at**	0.0356553	1.31	BRINP3	BMP/retinoic acid inducible neural specific 3
**223235_s_at**	0.0001034	1.29	SMOC2	SPARC related modular calcium binding 2
**220014_at**	0.0133806	1.29	PRR16	Proline rich 16
**210667_s_at**	0.0166368	1.29	C21orf33	Chromosome 21 open reading frame 33
**200064_at**	0.0320795	1.29	HSP90AB1	Heat shock protein 90 alpha family class B member 1
**226065_at**	0.0089545	1.28	PRICKLE1	Prickle planar cell polarity protein 1
**212979_s_at**	0.0113065	1.28	LOC100294033///TCAF1	Protein FAM115A-like///TRPM8 channel associated factor 1
**209742_s_at**	0.011878	1.28	MYL2	Myosin light chain 2
**201465_s_at**	0.0183769	1.28	JUN	Jun proto-oncogene, AP-1 transcription factor subunit
**203771_s_at**	0.0230784	1.28	BLVRA	Biliverdin reductase A
**200641_s_at**	0.0274483	1.28	YWHAZ	Tyrosine 3-monooxygenase/tryptophan 5-monooxygenase activation protein zeta
**203242_s_at**	0.0346093	1.28	PDLIM5	PDZ and LIM domain 5
**221051_s_at**	0.000258	1.27	NMRK2	Nicotinamide riboside kinase 2
**203725_at**	0.0032959	1.27	GADD45A	Growth arrest and DNA damage inducible alpha
**205693_at**	0.0065659	1.27	TNNT3	Troponin T3, fast skeletal type
**244771_at**	0.0109727	1.27	KBTBD12	Kelch repeat and BTB domain containing 12
**201841_s_at**	0.0175109	1.27	HSPB1	Heat shock protein family B (small) member 1
**221430_s_at**	0.0177213	1.27	RNF146	Ring finger protein 146

EXCLI Journal 2021;20:126-141– ISSN 1611-2156
Received: December 08, 2020, accepted: January 11, 2021, published: January 18, 2021

**200730_s_at**	0.0363001	1.27	PTP4A1	Protein tyrosine phosphatase type IVA, member 1
**216048_s_at**	0.00866	1.26	RHOBTB3	Rho related BTB domain containing 3
**1555679_a_ at**	0.0264038	1.26	RTN4IP1	Reticulon 4 interacting protein 1
**1552721_a_ at**	0.0345177	1.26	FGF1	Fibroblast growth factor 1
**235331_x_at**	0.0400264	1.26	PCGF5	Polycomb group ring finger 5
**209392_at**	0.0056681	1.25	ENPP2	Ectonucleotide pyrophospha-tase/phosphodiesterase 2
**211968_s_at**	0.0095766	1.25	HSP90AA1	Heat shock protein 90 alpha family class A member 1
**235321_at**	0.0096551	1.25	NDUFS1	NADH:ubiquinone oxidoreductase core subunit S1
**1555403_a_ at**	0.0430809	1.25	CDH19	Cadherin 19
**211317_s_at**	0.0498033	1.25	CFLAR	CASP8 and FADD like apoptosis regulator
**201552_at**	0.0391825	1.24	LAMP1	Lysosomal associated membrane protein 1
**208691_at**	0.0463388	1.24	TFRC	Transferrin receptor
**218839_at**	0.0003993	1.23	HEY1	Hes related family bhlh transcription factor with YRPW motif 1
**201946_s_at**	0.0039256	1.23	CCT2	Chaperonin containing TCP1 subu-nit 2
**239537_at**	0.0150926	1.23	ST8SIA2	ST8 alpha-N-acetyl-neuraminide al-pha-2,8-sialyltransferase 2
**215719_x_at**	0.0231005	1.23	FAS	Fas cell surface death receptor
**216640_s_at**	0.0383095	1.23	PDIA6	Protein disulfide isomerase family A member 6
**230708_at**	0.0450248	1.23	PRICKLE1	Prickle planar cell polarity protein 1
**226311_at**	0.0485591	1.23	ADAMTS2	ADAM metallopeptidase with throm-bospondin type 1 motif 2
**234304_s_at**	0.0041095	1.22	IPO11-LRRC70///IPO11	IPO11-LRRC70 readthrough///im-portin 11
**1557938_s_ at**	0.0153277	1.22	PTRF	Polymerase I and transcript release factor
**230788_at**	0.0467827	1.22	GCNT2	Glucosaminyl (N-acetyl) transferase 2, I-branching enzyme (I blood group)
**210218_s_at**	0.0041163	1.21	SP100	SP100 nuclear antigen
**227474_at**	0.0116171	1.21	PAX8-AS1	PAX8 antisense RNA 1
**203424_s_at**	0.025873	1.21	IGFBP5	Insulin like growth factor binding protein 5
**202458_at**	0.041184	1.21	PRSS23	Protease, serine 23
**228523_at**	0.0441877	1.21	NANOS1	Nanos C2HC-type zinc finger 1
**221423_s_at**	0.0465419	1.21	YIPF5	Yip1 domain family member 5

EXCLI Journal 2021;20:126-141– ISSN 1611-2156
Received: December 08, 2020, accepted: January 11, 2021, published: January 18, 2021

**220415_at**	0.0057141	1.2	FPGT-TNNI3K///TNNI3K	FPGT-TNNI3K readthrough///TNNI3 interacting kinase
**216234_s_at**	0.0091663	1.2	PRKACA	Protein kinase camp-activated catalytic subunit alpha
**208359_s_at**	0.0158636	1.2	KCNJ4	Potassium voltage-gated channel subfamily J member 4
**1555730_a_at**	0.0193469	1.2	CFL1	Cofilin 1
**217599_s_at**	0.0361966	1.2	MDFIC	Myod family inhibitor domain containing
**210954_s_at**	0.0366007	1.2	TSC22D2	TSC22 domain family member 2
**210839_s_at**	0.0112595	1.19	ENPP2	Ectonucleotide pyrophosphatase/phosphodiesterase 2
**202784_s_at**	0.0199335	1.19	NNT	Nicotinamide nucleotide transhydrogenase
**208893_s_at**	0.0064801	1.18	DUSP6	Dual specificity phosphatase 6
**217356_s_at**	0.0120975	1.18	PGK1	Phosphoglycerate kinase 1
**238081_at**	0.0127295	1.18	WDFY3-AS2	WDFY3 antisense RNA 2
**215464_s_at**	0.0202954	1.18	P2RX5-TAX1BP3///TAX1BP3///P2RX5	P2RX5-TAX1BP3 readthrough (NMD candidate)///Tax1 binding protein 3///purinergic receptor P2X 5
**216804_s_at**	0.0219862	1.18	PDLIM5	PDZ and LIM domain 5
**203582_s_at**	0.0321953	1.18	SPHAR///RAB4A	S-phase response (cyclin related)///RAB4A, member RAS oncogene family
**238794_at**	0.0406689	1.18	SFR1	SWI5 dependent homologous recombination repair protein 1
**214691_x_at**	0.0012502	1.17	FAM63B	Family with sequence similarity 63 member B
**1554414_a_at**	0.0031496	1.17	OSGIN2	Oxidative stress induced growth inhibitor family member 2
**213139_at**	0.011394	1.17	SNAI2	Snail family transcriptional repressor 2
**215505_s_at**	0.034034	1.17	STRN3	Striatin 3
**233230_s_at**	0.0462447	1.17	SLAIN2	SLAIN motif family member 2
**203642_s_at**	0.0065246	1.16	COBLL1	Cordon-bleu WH2 repeat protein like 1
**215711_s_at**	0.0316932	1.16	WEE1	WEE1 G2 checkpoint kinase
**202403_s_at**	0.0402818	1.16	COL1A2	Collagen type I alpha 2 chain
**218129_s_at**	0.0013358	1.15	NFYB	Nuclear transcription factor Y subunit beta
**227299_at**	0.0097686	1.15	CCNI	Cyclin I
**209668_x_at**	0.0100561	1.15	CES2	Carboxylesterase 2
**202137_s_at**	0.0104	1.15	ZMYND11	Zinc finger MYND-type containing 11
**214659_x_at**	0.0116445	1.15	YLPM1	YLP motif containing 1

EXCLI Journal 2021;20:126-141– ISSN 1611-2156
Received: December 08, 2020, accepted: January 11, 2021, published: January 18, 2021

**1555226_s_at**	0.0341456	1.15	C1orf43	Chromosome 1 open reading frame 43
**201466_s_at**	0.0360148	1.15	JUN	Jun proto-oncogene, AP-1 transcription factor subunit
**202465_at**	0.0366493	1.15	PCOLCE	Procollagen C-endopeptidase enhancer
**211981_at**	0.0419635	1.15	COL4A1	Collagen type IV alpha 1 chain
**200628_s_at**	0.0448476	1.15	WARS	Tryptophanyl-trna synthetase
**219645_at**	0.0008254	1.14	CASQ1	Calsequestrin 1
**233208_x_at**	0.013733	1.14	CPSF2	Cleavage and polyadenylation specific factor 2
**1554678_s_at**	0.0204427	1.14	HNRNPDL	Heterogeneous nuclear ribonucleoprotein D like
**1554411_at**	0.0286952	1.14	CTNNB1	Catenin beta 1
**222693_at**	0.0321084	1.14	LOC101928615///FNDC3B	Uncharacterized LOC101928615///fibronectin type III domain containing 3B
**214720_x_at**	0.0434061	1.14	10-Sep	Septin 10
**1554868_s_at**	0.0092093	1.13	PCNP	PEST proteolytic signal containing nuclear protein
**214541_s_at**	0.0214303	1.13	QKI	QKI, KH domain containing RNA binding
**222532_at**	0.0248206	1.13	SRPRB	SRP receptor beta subunit
**226282_at**	0.0328357	1.13	PTPN14	Protein tyrosine phosphatase, non-receptor type 14
**224595_at**	0.033906	1.13	SLC44A1	Solute carrier family 44 member 1
**241752_at**	0.0348258	1.13	SLC8A1	Solute carrier family 8 member A1
**209882_at**	0.0457294	1.13	RIT1	Ras like without CAAX 1
**226134_s_at**	0.0196859	1.12	MSI2	Musashi RNA binding protein 2
**223879_s_at**	0.0282281	1.12	OXR1	Oxidation resistance 1
**204238_s_at**	0.0347315	1.12	DNPH1	2'-deoxynucleoside 5'-phosphate N-hydrolase 1
**201120_s_at**	0.0391307	1.12	PGRMC1	Progesterone receptor membrane component 1
**1553873_at**	0.0394515	1.12	KLHL34	Kelch like family member 34
**214449_s_at**	0.0009546	1.11	RHOQ	Ras homolog family member Q
**200951_s_at**	0.0018346	1.11	CCND2	Cyclin D2
**1554747_a_at**	0.0070355	1.11	2-Sep	Septin 2
**226506_at**	0.0084189	1.11	THSD4	Thrombospondin type 1 domain containing 4
**222698_s_at**	0.008548	1.11	IMPACT	Impact RWD domain protein
**238613_at**	0.0122257	1.11	ZAK	Sterile alpha motif and leucine zipper containing kinase AZK
**228851_s_at**	0.0153161	1.11	ENSA	Endosulfine alpha
**200622_x_at**	0.0163304	1.11	CALM3///CALM2///CALM1	Calmodulin 3///calmodulin 2///calmodulin 1
**214730_s_at**	0.0246613	1.11	GLG1	Golgi glycoprotein 1

EXCLI Journal 2021;20:126-141– ISSN 1611-2156
Received: December 08, 2020, accepted: January 11, 2021, published: January 18, 2021

**208852_s_at**	0.0020222	1.1	CANX	Calnexin
**200671_s_at**	0.0049487	1.1	SPTBN1	Spectrin beta, non-erythrocytic 1
**200787_s_at**	0.0235191	1.1	PEA15	Phosphoprotein enriched in astro-cytes 15
**36554_at**	0.0245221	1.1	ASMTL	Acetylserotonin O-methyltransfer-ase-like
**226206_at**	0.0358073	1.1	MAFK	MAF bzip transcription factor K
**224061_at**	0.0000899	1.09	INMT	Indolethylamine N-methyltransfer-ase
**203827_at**	0.0004525	1.09	WIPI1	WD repeat domain, phosphoinosi-tide interacting 1
**213974_at**	0.0019287	1.09	ADAMTSL3	ADAMTS like 3
**225817_at**	0.0090272	1.09	LOC101930349///LOC101930344///CGNL1	Uncharacterized LOC101930349///uncharacterized LOC101930344///cingulin like 1
**212242_at**	0.0124051	1.09	TUBA4A	Tubulin alpha 4a
**1552678_a_at**	0.0468724	1.09	USP28	Ubiquitin specific peptidase 28
**223206_s_at**	0.0100698	1.08	NMRAL1	Nmra like redox sensor 1
**208748_s_at**	0.0157819	1.08	FLOT1	Flotillin 1
**200008_s_at**	0.0184179	1.08	GDI2	GDP dissociation inhibitor 2
**212878_s_at**	0.0374472	1.08	KLC1	Kinesin light chain 1
**202731_at**	0.0386727	1.08	MIR4680///PDCD4	Microrna 4680///programmed cell death 4 (neoplastic transformation inhibitor)
**219529_at**	0.0021657	1.07	CLIC3	Chloride intracellular channel 3
**209653_at**	0.0040063	1.07	KPNA4	Karyopherin subunit alpha 4
**1552417_a_at**	0.0047314	1.07	NEDD1	Neural precursor cell expressed, de-velopmentally down-regulated 1
**218486_at**	0.0108049	1.07	KLF11	Kruppel like factor 11
**210130_s_at**	0.0130961	1.07	TM7SF2	Transmembrane 7 superfamily member 2
**201162_at**	0.0199651	1.07	IGFBP7	Insulin like growth factor binding protein 7
**208677_s_at**	0.0303958	1.07	BSG	Basigin (Ok blood group)
**1555037_a_at**	0.0470131	1.07	IDH1	Isocitrate dehydrogenase (NADP(+)) 1, cytosolic
**213033_s_at**	0.0000974	1.06	NFIB	Nuclear factor I B
**205401_at**	0.0062235	1.06	AGPS	Alkylglycerone phosphate synthase
**1553117_a_at**	0.0062413	1.06	STK38	Serine/threonine kinase 38
**200679_x_at**	0.0070435	1.06	HMGB1	High mobility group box 1
**206042_x_at**	0.011051	1.06	SNURF///SNRPN	SNRPN upstream reading frame///small nuclear ribonucleopro-tein polypeptide N
**223181_at**	0.0138372	1.06	TIMM21	Translocase of inner mitochondrial membrane 21
**200793_s_at**	0.0196001	1.06	ACO2	Aconitase 2

EXCLI Journal 2021;20:126-141– ISSN 1611-2156
Received: December 08, 2020, accepted: January 11, 2021, published: January 18, 2021

**209958_s_at**	0.0200186	1.06	BBS9	Bardet-Biedl syndrome 9
**213068_at**	0.0307388	1.06	DPT	Dermatopontin
**228772_at**	0.0363237	1.06	HNMT	Histamine N-methyltransferase
**200790_at**	0.045314	1.06	ODC1	Ornithine decarboxylase 1

**68 down-regulated genes**				
**ID**	**p value**	**logFC**	**Gene symbol**	**Gene title**
**207558_s_at**	0.0246758	-4.59	PITX2	Paired like homeodomain 2
**241456_at**	0.003456	-2.97	FAM78B	Family with sequence similarity 78 member B
**239552_at**	0.0133993	-2.56	VWDE	Von Willebrand factor D and EGF domains
**209840_s_at**	0.0163268	-2.47	LRRN3	Leucine rich repeat neuronal 3
**224367_at**	0.0355149	-2.17	BEX2	Brain expressed X-linked 2
**239884_at**	0.0270866	-2.08	CADPS	Calcium dependent secretion activator
**203535_at**	0.040043	-1.98	S100A9	S100 calcium binding protein A9
**227830_at**	0.0362017	-1.97	GABRB3	Gamma-aminobutyric acid type A receptor beta3 subunit
**1564083_at**	0.0438367	-1.86	LOC101926975	Uncharacterized LOC101926975
**203407_at**	0.0065808	-1.84	PPL	Periplakin
**234989_at**	0.0483568	-1.8	MIR612///NEAT1	Microrna 612///nuclear paraspeckle assembly transcript 1 (non-protein coding)
**213904_at**	0.002461	-1.8	FRRS1L	Ferric chelate reductase 1 like
**225987_at**	0.0112932	-1.73	STEAP4	STEAP4 metalloreductase
**222927_s_at**	0.0318285	-1.72	CPLX3	Complexin 3
**32128_at**	0.0147193	-1.66	CCL18	C-C motif chemokine ligand 18
**214135_at**	0.011695	-1.64	CLDN18	Claudin 18
**230214_at**	0.0001773	-1.62	MRVI1	Murine retrovirus integration site 1 homolog
**230840_at**	0.0000741	-1.61	SMIM1	Small integral membrane protein 1 (Vel blood group)
**228057_at**	0.0003275	-1.59	DDIT4L	DNA damage inducible transcript 4 like
**243610_at**	0.0269431	-1.59	C9orf135	Chromosome 9 open reading frame 135
**220221_at**	0.0245545	-1.54	VPS13D	Vacuolar protein sorting 13 homolog D
**226973_at**	0.0348061	-1.5	VSTM2L	V-set and transmembrane domain containing 2 like
**229574_at**	0.0469879	-1.5	TRA2A	Transformer 2 alpha homolog
**239461_at**	0.0285949	-1.5	GALNT15	Polypeptide N-acetylgalactosaminyl-transferase 15

EXCLI Journal 2021;20:126-141– ISSN 1611-2156
Received: December 08, 2020, accepted: January 11, 2021, published: January 18, 2021

**204000_at**	0.0162027	-1.43	GNB5	G protein subunit beta 5
**239297_at**	0.000143	-1.42	LOC101927137///KIAA1456	Uncharacterized LOC101927137///KIAA1456
**203929_s_at**	0.0009585	-1.4	MAPT	Microtubule associated protein tau
**232529_at**	0.0159078	-1.38	SP3	Sp3 transcription factor
**229963_at**	0.0022633	-1.38	BEX5	Brain expressed X-linked 5
**213222_at**	0.0355888	-1.37	PLCB1	Phospholipase C beta 1
**214087_s_at**	0.0247119	-1.36	MYBPC1	Myosin binding protein C, slow type
**229414_at**	0.0018817	-1.35	PITPNC1	Phosphatidylinositol transfer protein, cytoplasmic 1
**228723_at**	0.0278874	-1.35	NPTN-IT1	NPTN intronic transcript 1
**231236_at**	0.0142462	-1.34	ZFP57	ZFP57 zinc finger protein
**227933_at**	0.0188981	-1.32	LINGO1	Leucine rich repeat and Ig domain containing 1
**238983_at**	0.0016003	-1.3	NSUN7	NOP2/Sun RNA methyltransferase family member 7
**1558828_s_at**	0.0479301	-1.3	CARMN	Cardiac mesoderm enhancer-associated non-coding RNA
**1569940_at**	0.011145	-1.29	SLC6A16	Solute carrier family 6 member 16
**231009_at**	0.0028721	-1.29	PLA2G12B	Phospholipase A2 group XIIB
**230641_at**	0.0030382	-1.28	LOC100505938	Uncharacterized LOC100505938
**229641_at**	0.0405711	-1.28	CCBE1	Collagen and calcium binding EGF domains 1
**228392_at**	0.0325633	-1.27	ZNF302	Zinc finger protein 302
**244165_at**	0.0339009	-1.25	FAM208B	Family with sequence similarity 208 member B
**206561_s_at**	0.0193367	-1.25	AKR1B10	Aldo-keto reductase family 1 member B10
**219478_at**	0.0349078	-1.24	WFDC1	WAP four-disulfide core domain 1
**219957_at**	0.0453076	-1.23	RUFY2	RUN and FYVE domain containing 2
**230270_at**	0.0262406	-1.22	PRPF38B	Pre-mrna processing factor 38B
**215795_at**	0.0449482	-1.22	MYH7B	Myosin heavy chain 7B
**232458_at**	0.0303198	-1.22	COL3A1	Collagen type III alpha 1 chain
**232027_at**	0.0134531	-1.21	SYNE1	Spectrin repeat containing nuclear envelope protein 1
**210432_s_at**	0.0340129	-1.21	SCN3A	Sodium voltage-gated channel alpha subunit 3
**213110_s_at**	0.0236621	-1.21	COL4A5	Collagen type IV alpha 5 chain
**219572_at**	0.0122784	-1.21	CADPS2	Calcium dependent secretion activator 2
**230913_at**	0.0268032	-1.21	ABCG1	ATP binding cassette subfamily G member 1
**227607_at**	0.0018609	-1.2	STAMBPL1	STAM binding protein like 1
**213280_at**	0.0000683	-1.2	RAP1GAP2	RAP1 gtpase activating protein 2
**229694_at**	0.0498724	-1.19	WDR11	WD repeat domain 11
**204232_at**	0.0246344	-1.19	FCER1G	Fc fragment of ige receptor Ig

EXCLI Journal 2021;20:126-141– ISSN 1611-2156
Received: December 08, 2020, accepted: January 11, 2021, published: January 18, 2021

**227654_at**	0.0354988	-1.16	FAM65C	Family with sequence similarity 65 member C
**230991_at**	0.0071354	-1.15	LOC102724 156	Uncharacterized LOC102724156
**220116_at**	0.0033171	-1.14	KCNN2	Potassium calcium-activated channel subfamily N member 2
**228559_at**	0.0165908	-1.13	CENPN	Centromere protein N
**226929_at**	0.0005592	-1.12	MTHFR	Methylenetetrahydrofolate reductase (NAD(P)H)
**201272_at**	0.0348511	-1.11	AKR1B1	Aldo-keto reductase family 1 member B
**203438_at**	0.0166568	-1.1	STC2	Stanniocalcin 2
**232500_at**	0.027324	-1.1	RALGAPA2	Ral gtpase activating protein catalytic alpha subunit 2
**1560785_at**	0.0375433	-1.08	DYRK3	Dual specificity tyrosine phosphorylation regulated kinase 3
**209569_x_at**	0.0085849	-1.07	NSG1	Neuron specific gene family member 1
