# Supplementary material for: Common differentially expressed genes and pathways correlating both coronary artery disease and atrial fibrillation
Source: EXCLI J. 2021 Jan 18;20:126–41. doi: 10.17179/excli2020-3262 (PMC7868642; doi:10.17179/excli2020-3262)
Supplement: Supplementary table 1 [file EXCLI-20-126-s-003.pdf]

EXCLI Journal 2021;20:126-141– ISSN 1611-2156
Received: December 08, 2020, accepted: January 11, 2021, published: January 18, 2021

Supplementary material to:

Original article:

COMMON DIFFERENTIALLY EXPRESSED GENES AND PATHWAYS CORRELATING BOTH CORONARY ARTERY DISEASE AND ATRIAL FIBRILLATION

Youjing Zheng, Jia-Qiang He*

Department of Biomedical Sciences and Pathobiology, College of Veterinary Medicine, Virginia Tech, Blacksburg, VA 24061, USA

* **Corresponding author:** Jia-Qiang He, Department of Biomedical Sciences and Pathobiology, Virginia Tech, Phase II, Room 252B, Blacksburg, VA 24061, USA. Tel: 1-540-231-2032. E-mail: jiahe@vt.edu

https://orcid.org/0000-0002-4825-7046 Youjing Zheng
https://orcid.org/0000-0002-0640-5960 Jia-Qiang He

http://dx.doi.org/10.17179/excli2020-3262

This is an Open Access article distributed under the terms of the Creative Commons Attribution License (http://creativecommons.org/licenses/by/4.0/).

Supplemental Table 1: Abbreviations used in the paper

Abbreviation	Full name
**ABCA5**	ATP binding cassette subfamily A member 5
**ABCB6**	ATP binding cassette subfamily B member 6 (Langereis blood group)
**ABCB9**	ATP binding cassette subfamily B member 9
**ABCC10**	ATP binding cassette subfamily C member 10
**ABCC13**	ATP binding cassette subfamily C member 13 (pseudogene)
**ABCC5**	ATP binding cassette subfamily C member 5
**ABCD3**	ATP binding cassette subfamily D member 3
**ABCE1**	ATP binding cassette subfamily E member 1
**ABCG1**	ATP binding cassette subfamily G member 1
**ABCG4**	ATP binding cassette subfamily G member 4
**ABHD18**	Abhydrolase domain containing 18
**ABHD2**	Abhydrolase domain containing 2
**ABHD5**	Abhydrolase domain containing 5
**ABI2**	Abl-interactor 2
**ABTB1**	Ankyrin repeat and BTB domain containing 1
**ACACB**	Acetyl-coa carboxylase beta
**ACAD8**	Acyl-coa dehydrogenase family member 8

EXCLI Journal 2021;20:126-141– ISSN 1611-2156
Received: December 08, 2020, accepted: January 11, 2021, published: January 18, 2021

**ACCS**	1-aminocyclopropane-1-carboxylate synthase homolog (inactive)
**ACER3**	Alkaline ceramidase 3
**ACKR1**	Atypical chemokine receptor 1 (Duffy blood group)
**ACKR3**	Atypical chemokine receptor 3
**ACO2**	Aconitase 2
**ACRC**	Acidic repeat containing
**ACSL1**	Acyl-coa synthetase long-chain family member 1
**ACTA1**	Actin, alpha 1, skeletal muscle
**ACVR2A**	Activin A receptor type 2A
**ADAMTS10**	ADAM metallopeptidase with thrombospondin type 1 motif 10
**ADAMTS2**	ADAM metallopeptidase with thrombospondin type 1 motif 2
**ADAMTSL3**	ADAMTS like 3
**ADAMTSL5**	ADAMTS like 5
**ADAP1**	Arfgap with dual PH domains 1
**ADCY10P1**	Adenylate cyclase 10, soluble pseudogene 1
**ADGRG3**	Adhesion G protein-coupled receptor G3
**AF**	Atrial fibrillation
**AFF3**	AF4/FMR2 family member 3
**AFG3L1P**	AFG3 like matrix AAA peptidase subunit 1, pseudogene
**AFG3L2**	AFG3 like matrix AAA peptidase subunit 2
**AGA**	Aspartylglucosaminidase
**AGBL3**	ATP/GTP binding protein like 3
**AGL**	Amylo-alpha-1, 6-glucosidase, 4-alpha-glucanotransferase
**AGMAT**	Agmatinase
**AGPS**	Alkylglycerone phosphate synthase
**AHSA2**	AHA1, activator of heat shock 90kda protein atpase homolog 2 (yeast)
**AHSP**	Alpha hemoglobin stabilizing protein
**AIFM1**	Apoptosis inducing factor, mitochondria associated 1
**AIMP1**	Aminoacyl trna synthetase complex interacting multifunctional protein 1
**AIRE**	Autoimmune regulator
**AKAP13**	A-kinase anchoring protein 13
**AKAP8**	A-kinase anchoring protein 8
**AKAP9**	A-kinase anchoring protein 9
**AKR1B1**	Aldo-keto reductase family 1 member B
**AKR1B10**	Aldo-keto reductase family 1 member B10
**ALAS2**	Alpha-hemoglobin-stabilizing protein
**ALDH8A1**	Aldehyde dehydrogenase 8 family member A1
**ALG13**	ALG13, UDP-N-acetylglucosaminyltransferase subunit
**ALMS1**	ALMS1, centrosome and basal body associated protein
**ALPPL2///ALPP**	Alkaline phosphatase, placental like 2///alkaline phosphatase, placental
**AMOTL1**	Angiomotin like 1
**ANAPC7**	Anaphase promoting complex subunit 7
**ANGEL2**	Angel homolog 2
**ANGPTL4**	Angiopoietin like 4
**ANK1**	Ankyrin 1

EXCLI Journal 2021;20:126-141– ISSN 1611-2156
Received: December 08, 2020, accepted: January 11, 2021, published: January 18, 2021

**ANK3**	Ankyrin 3, node of Ranvier (ankyrin G)
**ANKH**	ANKH inorganic pyrophosphate transport regulator
**ANKMY2**	Ankyrin repeat and MYND domain containing 2
**ANKRA2**	Ankyrin repeat family A member 2
**ANKRD10**	Ankyrin repeat domain 10
**ANKRD11**	Ankyrin repeat domain 11
**ANKRD12**	Ankyrin repeat domain 12
**ANKRD13C**	Ankyrin repeat domain 13C
**ANKRD2**	Ankyrin repeat domain 2
**ANKRD40**	Ankyrin repeat domain 40
**ANP32A**	Acidic nuclear phosphoprotein 32 family member A
**ANP32E**	Acidic nuclear phosphoprotein 32 family member E
**ANXA2R**	Annexin A2 receptor
**ANXA6**	Annexin A6
**AP1G2**	Adaptor related protein complex 1 gamma 2 subunit
**AP2A1**	Adaptor related protein complex 2 alpha 1 subunit
**AP5M1**	Adaptor related protein complex 5 mu 1 subunit
**APBA2**	Amyloid beta precursor protein binding family A member 2
**APC2**	APC2, WNT signaling pathway regulator
**APCDD1L-AS1**	APCDD1L antisense RNA 1 (head to head)
**API5**	Apoptosis inhibitor 5
**APOA1**	Apolipoprotein A1
**APO-BEC3A_B///APOBEC3A**	APOBEC3A and APOBEC3B deletion hybrid///apolipoprotein B mrna editing enzyme catalytic subunit 3A
**APOBEC3B**	Apolipoprotein B mrna editing enzyme catalytic subunit 3B
**APOL3**	Apolipoprotein L3
**APP**	Amyloid beta precursor protein
**AQP9**	Aquaporin 9
**ARGLU1**	Arginine and glutamate rich 1
**ARHGAP32**	Rho gtpase activating protein 32
**ARHGAP33**	Rho gtpase activating protein 33
**ARHGAP5**	Rho gtpase activating protein 5
**ARHGEF12**	Rho guanine nucleotide exchange factor 12
**ARHGEF3**	Rho guanine nucleotide exchange factor 3
**ARHGEF7**	Rho guanine nucleotide exchange factor 7
**ARHGEF9**	Cdc42 guanine nucleotide exchange factor 9
**ARID2**	AT-rich interaction domain 2
**ARIH2**	Ariadne RBR E3 ubiquitin protein ligase 2
**ARL17B///ARL17A**	ADP ribosylation factor like gtpase 17B///ADP ribosylation factor like gtpase 17A
**ARL4A**	ADP ribosylation factor like gtpase 4A
**ARMCX5**	Armadillo repeat containing, X-linked 5
**ARSI**	Arylsulfatase family member I
**ART4**	ADP-ribosyltransferase 4 (Dombrock blood group)

EXCLI Journal 2021;20:126-141– ISSN 1611-2156
Received: December 08, 2020, accepted: January 11, 2021, published: January 18, 2021

**ASB6**	Ankyrin repeat and SOCS box containing 6
**ASMTL**	Acetylserotonin O-methyltransferase-like
**ASNS**	Asparagine synthetase (glutamine-hydrolyzing)
**ASXL1**	Additional sex combs like 1, transcriptional regulator
**ASXL2**	Additional sex combs like 2, transcriptional regulator
**ATAD2B**	Atpase family, AAA domain containing 2B
**ATF2**	Activating transcription factor 2
**ATF3**	Activating transcription factor 3
**ATF6B**	Activating transcription factor 6 beta
**ATF7**	Activating transcription factor 7
**ATF7IP2**	Activating transcription factor 7 interacting protein 2
**ATG10**	Autophagy related 10
**ATG14**	Autophagy related 14
**ATM**	ATM serine/threonine kinase
**ATP10A**	Atpase phospholipid transporting 10A (putative)
**ATP11A-AS1**	ATP11A antisense RNA 1
**ATP2A2**	Atpase sarcoplasmic/endoplasmic reticulum Ca2+ transporting 2
**ATP5C1**	ATP synthase, H+ transporting, mitochondrial F1 complex, gamma polypeptide 1
**ATP6V1H**	Atpase H+ transporting V1 subunit H
**ATP8B1**	Atpase phospholipid transporting 8B1
**ATR**	ATR serine/threonine kinase
**ATXN7L1**	Ataxin 7 like 1
**AXIN2**	Axin 2
**B3GNT2**	UDP-glcnac:betagal beta-1,3-N-acetylglucosaminyltransferase 2
**BAALC-AS2**	BAALC antisense RNA 2
**BACH2**	BTB domain and CNC homolog 2
**BAG1**	BCL2 associated athanogene 1
**BASP1**	Brain abundant membrane attached signal protein 1
**BBOF1**	Basal body orientation factor 1
**BBS10**	Bardet-Biedl syndrome 10
**BBS7**	Bardet-Biedl syndrome 7
**BBS9**	Bardet-Biedl syndrome 9
**BBX**	BBX, HMG-box containing
**BCAS4**	Breast carcinoma amplified sequence 4
**BCCIP**	BRCA2 and CDKN1A interacting protein
**BCL2L1**	BCL2 like 1
**BDH2**	3-hydroxybutyrate dehydrogenase, type 2
**BEX2**	Brain expressed X-linked 2
**BEX2**	Brain expressed X-linked 2
**BEX5**	Brain expressed X-linked 5
**BICD1**	BICD cargo adaptor 1
**BICDL1**	BICD family like cargo adaptor 1
**BIRC6**	Baculoviral IAP repeat containing 6

EXCLI Journal 2021;20:126-141– ISSN 1611-2156
Received: December 08, 2020, accepted: January 11, 2021, published: January 18, 2021

**BIVM**	Basic, immunoglobulin-like variable motif containing
**BLNK**	B-cell linker
**BLVRA**	Biliverdin reductase A
**BLZF1**	Basic leucine zipper nuclear factor 1
**BOLA2**	Bola family member 2
**BORCS5**	BLOC-1 related complex subunit 5
**BP**	Biological process
**BPTF**	Bromodomain PHD finger transcription factor
**BRCC3**	BRCA1/BRCA2-containing complex subunit 3
**BRINP3**	BMP/retinoic acid inducible neural specific 3
**BRWD1**	Bromodomain and WD repeat domain containing 1
**BSG**	Basigin (Ok blood group)
**BTAF1**	B-TFIID TATA-box binding protein associated factor 1
**BTBD11**	BTB domain containing 11
**BTBD3**	BTB domain containing 3
**BTG1**	BTG anti-proliferation factor 1
**C11orf1**	Chromosome 11 open reading frame 1
**C11orf31**	Chromosome 11 open reading frame 31
**C11orf80**	Chromosome 11 open reading frame 80
**C11orf94**	Chromosome 11 open reading frame 94
**C12orf65**	Chromosome 12 open reading frame 65
**C12orf66**	Chromosome 12 open reading frame 66
**C17orf99**	Chromosome 17 open reading frame 99
**C1GALT1**	Core 1 synthase, glycoprotein-N-acetylgalactosamine 3-beta-galacto-syltransferase 1
**C1orf109**	Chromosome 1 open reading frame 109
**C1orf228**	Chromosome 1 open reading frame 228
**C1orf27**	Chromosome 1 open reading frame 27
**C1orf43**	Chromosome 1 open reading frame 43
**C1QC**	Complement C1q C chain
**C1QTNF5///MFRP**	C1q and tumor necrosis factor related protein 5///membrane frizzled-related protein
**C21orf33**	Chromosome 21 open reading frame 33
**C21orf62-AS1**	C21orf62 antisense RNA 1
**C3**	Complement component 3
**C3orf38**	Chromosome 3 open reading frame 38
**C4B_2///C4B///C4A**	Complement component 4B (Chido blood group), copy 2///complement component 4B (Chido blood group)///complement component 4A (Rodgers blood group)
**C4BPA**	Complement component 4 binding protein alpha
**C5orf24**	Chromosome 5 open reading frame 24
**C5orf58**	Chromosome 5 open reading frame 58
**C5orf63**	Chromosome 5 open reading frame 63
**C6orf136**	Chromosome 6 open reading frame 136
**C6orf62**	Chromosome 6 open reading frame 62

EXCLI Journal 2021;20:126-141– ISSN 1611-2156
Received: December 08, 2020, accepted: January 11, 2021, published: January 18, 2021

**C7orf55-LUC7L2///LUC7L2**	C7orf55-LUC7L2 readthrough///LUC7 like 2, pre-mrna splicing factor
**C8orf44-SGK3///C8orf44///SGK3**	C8orf44-SGK3 readthrough///chromosome 8 open reading frame 44///serum/glucocorticoid regulated kinase family member 3
**C9orf116**	Chromosome 9 open reading frame 116
**C9orf135**	Chromosome 9 open reading frame 135
**C9orf85**	Chromosome 9 open reading frame 85
**CA1**	Carbonic anhydrase 1
**CACNG4**	Calcium voltage-gated channel auxiliary subunit gamma 4
**CAD**	Coronary artery disease
**CADPS**	Calcium dependent secretion activator
**CADPS2**	Calcium dependent secretion activator 2
**CALM3///CALM2///CALM1**	Calmodulin 3///calmodulin 2///calmodulin 1
**CALU**	Calumenin
**CAMK2D**	Calcium/calmodulin dependent protein kinase II delta
**CAMK4**	Calcium/calmodulin dependent protein kinase IV
**CAND1**	Cullin associated and neddylation dissociated 1
**CAND1.11///AMPD3**	Uncharacterized LOC100130460///adenosine monophosphate deaminase 3
**CANT1**	Calcium activated nucleotidase 1
**CANX**	Calnexin
**CAPN1**	Calpain 1
**CAPN3**	Calpain 3
**CAPN7**	Calpain 7
**CAPRIN2**	Caprin family member 2
**CAPS**	Calcyphosine
**CARMN**	Cardiac mesoderm enhancer-associated non-coding RNA
**CARNMT1**	Carnosine N-methyltransferase 1
**CASD1**	CAS1 domain containing 1
**CASP4**	Caspase 4
**CASQ1**	Calsequestrin 1
**CAT**	Catalase
**CBLB**	Cbl proto-oncogene B
**CBR4**	Carbonyl reductase 4
**CBWD7///CBWD6///CBWD3///CBWD5///CBWD2///CBWD1**	COBW domain containing 7///COBW domain containing 6///COBW domain containing 3///COBW domain containing 5///COBW domain containing 2///COBW domain containing 1
**CBX5**	Chromobox 5
**CC**	Cellular component (CC)
**CCAR1**	Cell division cycle and apoptosis regulator 1
**CCBE1**	Collagen and calcium binding EGF domains 1
**CCDC134**	Coiled-coil domain containing 134

EXCLI Journal 2021;20:126-141– ISSN 1611-2156
Received: December 08, 2020, accepted: January 11, 2021, published: January 18, 2021

**CCDC14**	Coiled-coil domain containing 14
**CCDC154**	Coiled-coil domain containing 154
**CCDC18**	Coiled-coil domain containing 18
**CCDC186**	Coiled-coil domain containing 186
**CCDC187**	Coiled-coil domain containing 187
**CCDC191**	Coiled-coil domain containing 191
**CCDC66**	Coiled-coil domain containing 66
**CCDC82**	Coiled-coil domain containing 82
**CCDC84**	Coiled-coil domain containing 84
**CCDC88A**	Coiled-coil domain containing 88A
**CCDC91**	Coiled-coil domain containing 91
**CCDC93**	Coiled-coil domain containing 93
**CCL18**	C-C motif chemokine ligand 18
**CCNA2**	Cyclin A2
**CCND1**	Cyclin D1
**CCND2**	Cyclin D2
**CCND2**	Cyclin D2
**CCNI**	Cyclin I
**CCNJL**	Cyclin J like
**CCNL1**	Cyclin L1
**CCNL2**	Cyclin L2
**CCNT2**	Cyclin T2
**CCP110**	Centriolar coiled-coil protein 110
**CCT2**	Chaperonin containing TCP1 subunit 2
**CD46**	CD46 molecule
**CD47**	CD47 molecule
**CD48**	CD48 molecule
**CD69**	CD69 molecule
**CD79B**	CD79b molecule
**CD96**	CD96 molecule
**CDAN1**	Codanin 1
**CDC42BPA**	CDC42 binding protein kinase alpha
**CDC42EP2**	CDC42 effector protein 2
**CDC7**	Cell division cycle 7
**CDH19**	Cadherin 19
**CDH2**	Cadherin 2
**CDK10**	Cyclin dependent kinase 10
**CDK11A///CDK11B**	Cyclin dependent kinase 11A///cyclin dependent kinase 11B
**CDK13**	Cyclin dependent kinase 13
**CDK15**	Cyclin dependent kinase 15
**CDK8**	Cyclin dependent kinase 8
**CEACAM1**	Carcinoembryonic antigen related cell adhesion molecule 1
**CEACAM3**	Carcinoembryonic antigen related cell adhesion molecule 3

EXCLI Journal 2021;20:126-141– ISSN 1611-2156
Received: December 08, 2020, accepted: January 11, 2021, published: January 18, 2021

**CECR5**	Cat eye syndrome chromosome region, candidate 5
**CENPK**	Centromere protein K
**CENPN**	Centromere protein N
**CENPV**	Centromere protein V
**CEP135**	Centrosomal protein 135
**CEP152**	Centrosomal protein 152
**CEP192**	Centrosomal protein 192
**CEP290**	Centrosomal protein 290
**CEP350**	Centrosomal protein 350
**CEP44**	Centrosomal protein 44
**CEP76**	Centrosomal protein 76
**CEP95**	Centrosomal protein 95
**CES2**	Carboxylesterase 2
**CFDP1**	Craniofacial development protein 1
**CFL1**	Cofilin 1
**CFL2**	Cofilin 2
**CFLAR**	CASP8 and FADD like apoptosis regulator
**CHADL**	Chondroadherin like
**CHCHD7**	Coiled-coil-helix-coiled-coil-helix domain containing 7
**CHD2**	Chromodomain helicase DNA binding protein 2
**CHD9**	Chromodomain helicase DNA binding protein 9
**CHGA**	Chromogranin A
**CHI3L1**	Chitinase 3 like 1
**CHKB**	Choline kinase beta
**CHPT1**	Choline phosphotransferase 1
**CHRM3-AS2**	CHRM3 antisense RNA 2
**CIRBP**	Cold inducible RNA binding protein
**CLC**	Charcot-Leyden crystal galectin
**CLCC1**	Chloride channel CLIC like 1
**CLDN14**	Claudin 14
**CLDN18**	Claudin 18
**CLDN6**	Claudin 6
**CLDND1**	Claudin domain containing 1
**CLEC2D**	C-type lectin domain family 2 member D
**CLEC7A**	C-type lectin domain family 7 member A
**CLIC3**	Chloride intracellular channel 3
**CLK4**	CDC like kinase 4
**CLMP**	CXADR like membrane protein
**CLOCK**	Clock circadian regulator
**CMAHP**	Cytidine monophospho-N-acetylneuraminic acid hydroxylase, pseudo-gene
**CNDP2**	CNDP dipeptidase 2 (metallopeptidase M20 family)
**CNOT7**	CCR4-NOT transcription complex subunit 7
**CNR1**	Cannabinoid receptor 1

EXCLI Journal 2021;20:126-141– ISSN 1611-2156
Received: December 08, 2020, accepted: January 11, 2021, published: January 18, 2021

**CNTN2**	Contactin 2
**CNTRL**	Centriolin
**COA5**	Cytochrome c oxidase assembly factor 5
**COBLL1**	Cordon-bleu WH2 repeat protein like 1
**COL11A2**	Collagen type XI alpha 2 chain
**COL17A1**	Collagen type XVII alpha 1 chain
**COL1A1**	Collagen type I alpha 1 chain
**COL1A2**	Collagen type I alpha 2 chain
**COL3A1**	Collagen type III alpha 1 chain
**COL4A1**	Collagen type IV alpha 1 chain
**COL4A2**	Collagen type IV alpha 2 chain
**COL4A3**	Collagen type IV alpha 3 chain
**COL4A5**	Collagen type IV alpha 5 chain
**COL5A1**	Collagen type V alpha 1 chain
**COL5A2**	Collagen type V alpha 2 chain
**COL8A1**	Collagen type VIII alpha 1 chain
**COLEC11**	Collectin subfamily member 11
**CORO2A**	Coronin 2A
**COX11**	COX11, cytochrome c oxidase copper chaperone
**COX20**	COX20, cytochrome c oxidase assembly factor
**COX4I1**	Cytochrome c oxidase subunit 4I1
**CPA4**	Carboxypeptidase A4
**CPLX3**	Complexin 3
**CPNE8**	Copine 8
**CPSF2**	Cleavage and polyadenylation specific factor 2
**CR1L**	Complement component 3b/4b receptor 1-like
**CRAMP1**	Cramped chromatin regulator homolog 1
**CREB1**	Camp responsive element binding protein 1
**CREB3L1**	Camp responsive element binding protein 3 like 1
**CREBZF**	CREB/ATF bzip transcription factor
**CRHBP**	Corticotropin releasing hormone binding protein
**CRHR1**	Corticotropin releasing hormone receptor 1
**CRIPAK**	Cysteine rich PAK1 inhibitor
**CROCCP2**	Ciliary rootlet coiled-coil, rootletin pseudogene 2
**CRTC3**	CREB regulated transcription coactivator 3
**CRYBB2**	Crystallin beta B2
**CRYBG3**	Crystallin beta-gamma domain containing 3
**CRYZL1**	Crystallin zeta like 1
**CSDC2**	Cold shock domain containing C2
**CSNK1E**	Casein kinase 1 epsilon
**CSNK1G1**	Casein kinase 1 gamma 1
**CSPP1**	Centrosome and spindle pole associated protein 1
**CSTF3**	Cleavage stimulation factor subunit 3
**CTNNB1**	Catenin beta 1

EXCLI Journal 2021;20:126-141– ISSN 1611-2156
Received: December 08, 2020, accepted: January 11, 2021, published: January 18, 2021

**CTRC**	Chymotrypsin C
**CTSC**	Cathepsin C
**CTSK**	Cathepsin K
**CTSZ**	Cathepsin Z
**CUL4A**	Cullin 4A
**CWC22**	CWC22 homolog, spliceosome-associated protein
**CXCL2**	C-X-C motif chemokine ligand 2
**CXCR1**	C-X-C motif chemokine receptor 1
**CYBRD1**	Cytochrome b reductase 1
**CYP4F3**	Cytochrome P450 family 4 subfamily F member 3
**CYP4V2**	Cytochrome P450 family 4 subfamily V member 2
**CYR61**	Cysteine rich angiogenic inducer 61
**CYR61**	Protein CYR61
**D21S2088E**	D21s2088e
**DAVID**	Database for Annotation, Visualization and Integrated Discovery
**DBF4**	DBF4 zinc finger
**DCAF1**	DDB1 and CUL4 associated factor 1
**DCAF10**	DDB1 and CUL4 associated factor 10
**DCAF16**	DDB1 and CUL4 associated factor 16
**DCAF7**	DDB1 and CUL4 associated factor 7
**DCLRE1C**	DNA cross-link repair 1C
**DCUN1D1**	Defective in cullin neddylation 1 domain containing 1
**DCUN1D4**	Defective in cullin neddylation 1 domain containing 4
**DDIT4L**	DNA damage inducible transcript 4 like
**DDX10**	DEAD-box helicase 10
**DDX18**	DEAD-box helicase 18
**DDX42**	DEAD-box helicase 42
**DDX51**	DEAD-box helicase 51
**DDX52**	DEAD-box helicase 52
**DDX55**	DEAD-box helicase 55
**DDX58**	DEXD/H-box helicase 58
**DDX59**	DEAD-box helicase 59
**DDX6**	DEAD-box helicase 6
**DEAF1**	DEAF1, transcription factor
**DEFA4**	Defensin alpha 4
**DEGs**	Common differentially expressed genes
**DEGS2**	Delta 4-desaturase, sphingolipid 2
**DENND4A**	DENN domain containing 4A
**DENND4B**	DENN domain containing 4B
**DFNB59**	Deafness, autosomal recessive 59
**DGAT2**	Diacylglycerol O-acyltransferase 2
**DGKD**	Diacylglycerol kinase delta
**DGKE**	Diacylglycerol kinase epsilon
**DHFR2**	Dihydrofolate reductase 2

EXCLI Journal 2021;20:126-141– ISSN 1611-2156
Received: December 08, 2020, accepted: January 11, 2021, published: January 18, 2021

**DHRS2**	Dehydrogenase/reductase 2
**DHRS9**	Dehydrogenase/reductase 9
**DHX30**	DEAH-box helicase 30
**DIDO1**	Death inducer-obliterator 1
**DIS3L2**	DIS3 like 3'-5' exoribonuclease 2
**DKFZP586B0319**	DKFZP586B0319 protein
**DLG1**	Discs large MAGUK scaffold protein 1
**DLG4**	Discs large MAGUK scaffold protein 4
**DLGAP4**	DLG associated protein 4
**DMTN**	Dematin actin binding protein
**DMXL1**	Dmx like 1
**DNAH2**	Dynein axonemal heavy chain 2
**DNAJA4**	Dnaj heat shock protein family (Hsp40) member A4
**DNAJB4**	Dnaj heat shock protein family (Hsp40) member B4
**DNAJC10**	Dnaj heat shock protein family (Hsp40) member C10
**DNAJC2**	Dnaj heat shock protein family (Hsp40) member C2
**DNAJC21**	Dnaj heat shock protein family (Hsp40) member C21
**DNPH1**	2'-deoxynucleoside 5'-phosphate N-hydrolase 1
**DOCK4**	Dedicator of cytokinesis 4
**DOCK9**	Dedicator of cytokinesis 9
**DOPEY1**	Dopey family member 1
**DPCD**	Deleted in primary ciliary dyskinesia homolog (mouse)
**DPF1**	Double PHD fingers 1
**DPH5**	Diphthamide biosynthesis 5
**DPH6**	Diphthamine biosynthesis 6
**DPH7**	Diphthamide biosynthesis 7
**DPT**	Dermatopontin
**DROSHA**	Drosha ribonuclease III
**DSC2**	Desmocollin 2
**DSCAM**	DS cell adhesion molecule
**DUSP1**	Dual specificity phosphatase 1
**DUSP6**	Dual specificity phosphatase 6
**DYRK1B**	Dual specificity tyrosine phosphorylation regulated kinase 1B
**DYRK2**	Dual specificity tyrosine phosphorylation regulated kinase 2
**DYRK3**	Dual specificity tyrosine phosphorylation regulated kinase 3
**DZIP3**	DAZ interacting zinc finger protein 3
**E2F2**	E2F transcription factor 2
**EAF2**	ELL associated factor 2
**EBAG9**	Estrogen receptor binding site associated, antigen, 9
**EBF1**	Early B-cell factor 1
**EBP41**	Protein 4.1
**EDRF1**	Erythroid differentiation regulatory factor 1
**EEA1**	Early endosome antigen 1

EXCLI Journal 2021;20:126-141– ISSN 1611-2156
Received: December 08, 2020, accepted: January 11, 2021, published: January 18, 2021

**EFCAB7**	EF-hand calcium binding domain 7
**EFNA5**	Ephrin A5
**EGFL7**	EGF like domain multiple 7
**EGLN1**	Egl-9 family hypoxia inducible factor 1
**EGLN3**	Egl-9 family hypoxia inducible factor 3
**EGR1**	Early growth response 1
**EHBP1**	EH domain binding protein 1
**EHD4**	EH domain containing 4
**EHMT2**	Euchromatic histone lysine methyltransferase 2
**EIF1AX**	Eukaryotic translation initiation factor 1A, X-linked
**EIF2AK4**	Eukaryotic translation initiation factor 2 alpha kinase 4
**EIF2S3**	Eukaryotic translation initiation factor 2 subunit gamma
**EIF3A**	Eukaryotic translation initiation factor 3 subunit A
**EIF3C**	Eukaryotic translation initiation factor 3 subunit C
**EIF3F**	Eukaryotic translation initiation factor 3 subunit F
**EIF3J-AS1**	EIF3J antisense RNA 1 (head to head)
**EIF4G3**	Eukaryotic translation initiation factor 4 gamma 3
**EIF5B**	Eukaryotic translation initiation factor 5B
**ELF3**	E74 like ETS transcription factor 3
**ELK3**	ELK3, ETS transcription factor
**ELK4**	ELK4, ETS transcription factor
**ELL2**	Elongation factor for RNA polymerase II 2
**ELP2**	Elongator acetyltransferase complex subunit 2
**EMID1**	EMI domain containing 1
**EML6**	Echinoderm microtubule associated protein like 6
**ENOSF1**	Enolase superfamily member 1
**ENPP2**	Ectonucleotide pyrophosphatase/phosphodiesterase 2
**ENPP7**	Ectonucleotide pyrophosphatase/phosphodiesterase 7
**ENSA**	Endosulfine alpha
**ENTPD4**	Ectonucleoside triphosphate diphosphohydrolase 4
**ENY2**	ENY2, transcription and export complex 2 subunit
**EOGT**	EGF domain specific O-linked N-acetylglucosamine transferase
**EPB41**	Erythrocyte membrane protein band 4.1
**EPB41L4A-AS1**	EPB41L4A antisense RNA 1
**EPC1**	Enhancer of polycomb homolog 1
**EPHB2**	EPH receptor B2
**EPM2AIP1**	EPM2A interacting protein 1
**EPPK1**	Epiplakin 1
**ERCC6L2**	ERCC excision repair 6 like 2
**ERP27**	Endoplasmic reticulum protein 27
**ERVK13-1**	Endogenous retrovirus group K13 member 1
**ERVK3-1///ZNF8**	Endogenous retrovirus group K3 member 1///zinc finger protein 8
**ESF1**	ESF1 nucleolar pre-rrna processing protein homolog
**ESPN**	Espin

EXCLI Journal 2021;20:126-141– ISSN 1611-2156
Received: December 08, 2020, accepted: January 11, 2021, published: January 18, 2021

**EVX1**	Even-skipped homeobox 1
**EXOC1**	Exocyst complex component 1
**EXOG**	Endo/exonuclease (5'-3'), endonuclease G-like
**EZH2**	Enhancer of zeste 2 polycomb repressive complex 2 subunit
**F10**	Coagulation factor X
**F2RL1**	F2R like trypsin receptor 1
**FABP4**	Fatty acid binding protein 4
**FABP4**	Fatty acid-binding protein
**FAIM**	Fas apoptotic inhibitory molecule
**FAM122C**	Family with sequence similarity 122C
**FAM124A**	Family with sequence similarity 124 member A
**FAM133DP///FAM133B**	Family with sequence similarity 133, member A pseudogene///family with sequence similarity 133 member B
**FAM134B**	Family with sequence similarity 134 member B
**FAM13A**	Family with sequence similarity 13 member A
**FAM13A-AS1**	FAM13A antisense RNA 1
**FAM156B///FAM156A**	Family with sequence similarity 156 member B///family with sequence similarity 156 member A
**FAM171A1**	Family with sequence similarity 171 member A1
**FAM172A**	Family with sequence similarity 172 member A
**FAM179B**	Family with sequence similarity 179 member B
**FAM193B**	Family with sequence similarity 193 member B
**FAM199X**	Family with sequence similarity 199, X-linked
**FAM208B**	Family with sequence similarity 208 member B
**FAM208B**	Family with sequence similarity 208 member B
**FAM210B**	Family with sequence similarity 210 member B
**FAM216A**	Family with sequence similarity 216 member A
**FAM226B///FAM226A**	Family with sequence similarity 226 member B (non-protein coding)///family with sequence similarity 226 member A (non-protein coding)
**FAM231D///LOC388692**	Family with sequence similarity 231 member D///uncharacterized LOC388692
**FAM30A**	Family with sequence similarity 30, member A
**FAM43A**	Family with sequence similarity 43 member A
**FAM57B**	Family with sequence similarity 57 member B
**FAM63B**	Family with sequence similarity 63 member B
**FAM65C**	Family with sequence similarity 65 member C
**FAM76B**	Family with sequence similarity 76 member B
**FAM78B**	Family with sequence similarity 78 member B
**FANCA**	Fanconi anemia complementation group A
**FANCD2**	Fanconi anemia complementation group D2
**FANCI**	Fanconi anemia complementation group I
**FANCL**	Fanconi anemia complementation group L
**FANCM**	Fanconi anemia complementation group M
**FARP1**	FERM, ARH/rhogef and pleckstrin domain protein 1

EXCLI Journal 2021;20:126-141– ISSN 1611-2156
Received: December 08, 2020, accepted: January 11, 2021, published: January 18, 2021

**FAS**	Fas cell surface death receptor
**FASN**	Fatty acid synthase
**FASTKD1**	FAST kinase domains 1
**FASTKD2**	FAST kinase domains 2
**FBRSL1**	Fibrosin like 1
**FBXL17**	F-box and leucine rich repeat protein 17
**FBXL6**	F-box and leucine rich repeat protein 6
**FBXO22**	F-box protein 22
**FBXO3**	F-box protein 3
**FBXO7**	F-box protein 7
**FBXO9**	F-box protein 9
**FCER1G**	Fc fragment of ige receptor Ig
**FCGBP**	Fc fragment of igg binding protein
**FCRL1**	Fc receptor like 1
**FCRL2**	Fc receptor like 2
**FCRL5**	Fc receptor like 5
**FEM1B**	Fem-1 homolog B
**FGF1**	Fibroblast growth factor 1
**FGF13**	Fibroblast growth factor 13
**FGFR1OP**	FGFR1 oncogene partner
**FGFR1OP2**	FGFR1 oncogene partner 2
**FH**	Fumarate hydratase
**FHL1**	Four and a half LIM domains 1
**FHL2**	Four and a half LIM domains 2
**FIGNL1**	Fidgetin like 1
**FITM2**	Fat storage inducing transmembrane protein 2
**FKBP8**	FK506 binding protein 8
**FKRP**	Fukutin related protein
**FLCN**	Folliculin
**FLOT1**	Flotillin 1
**FLVCR1**	Feline leukemia virus subgroup C cellular receptor 1
**FMNL3**	Formin like 3
**FNBP4**	Formin binding protein 4
**FNDC4**	Fibronectin type III domain containing 4
**FOSB**	Fosb proto-oncogene, AP-1 transcription factor subunit
**FOXN3**	Forkhead box N3
**FOXO1**	Forkhead box O1
**FOXO3B///FOXO3**	Forkhead box O3B pseudogene///forkhead box O3
**FOXP1**	Forkhead box P1
**FOXP2**	Forkhead box P2
**FPGT-TNNI3K///TNNI3K**	FPGT-TNNI3K readthrough///TNNI3 interacting kinase
**FPR2**	Formyl peptide receptor 2

EXCLI Journal 2021;20:126-141– ISSN 1611-2156
Received: December 08, 2020, accepted: January 11, 2021, published: January 18, 2021

**FRG1JP**	FSHD region gene 1 family member J, pseudogene
**FRMD4A**	FERM domain containing 4A
**FRRS1L**	Ferric chelate reductase 1 like
**FUBP1**	Far upstream element binding protein 1
**FUBP3**	Far upstream element binding protein 3
**FUNDC1**	FUN14 domain containing 1
**FURIN**	Furin, paired basic amino acid cleaving enzyme
**FUS**	FUS RNA binding protein
**FUT3**	Fucosyltransferase 3 (Lewis blood group)
**FXR1**	FMR1 autosomal homolog 1
**FZD2**	Frizzled class receptor 2
**GABBR1**	Gamma-aminobutyric acid type B receptor subunit 1
**GABPB1-AS1**	GABPB1 antisense RNA 1
**GABRB3**	Gamma-aminobutyric acid type A receptor beta3 subunit
**GADD45A**	Growth arrest and DNA damage inducible alpha
**GAK**	Cyclin G associated kinase
**GALK2**	Galactokinase 2
**GALNT15**	Polypeptide N-acetylgalactosaminyltransferase 15
**GALNT8///KCNA6**	Polypeptide N-acetylgalactosaminyltransferase 8///potassium voltage-gated channel subfamily A member 6
**GALNT9**	Polypeptide N-acetylgalactosaminyltransferase 9
**GALR3**	Galanin receptor 3
**GATA1**	GATA binding protein 1
**GATA3**	GATA binding protein 3
**GATAD1**	GATA zinc finger domain containing 1
**GATM**	Glycine amidinotransferase
**GBGT1**	Globoside alpha-1,3-N-acetylgalactosaminyltransferase 1
**GBP3**	Guanylate binding protein 3
**GCAT**	Glycine C-acetyltransferase
**GCFC2**	GC-rich sequence DNA-binding factor 2
**GCNT2**	Glucosaminyl (N-acetyl) transferase 2, I-branching enzyme (I blood group)
**GDI2**	GDP dissociation inhibitor 2
**GDPD1**	Glycerophosphodiester phosphodiesterase domain containing 1
**GEO**	Gene expression omnibus
**GFM1**	G elongation factor mitochondrial 1
**GFM2**	G elongation factor mitochondrial 2
**GGA2**	Golgi associated, gamma adaptin ear containing, ARF binding protein 2
**GIMAP1**	Gtpase, IMAP family member 1
**GIN1**	Gypsy retrotransposon integrase 1
**GIT1**	GIT arfgap 1
**GJC2**	Gap junction protein gamma 2
**GK**	Glycerol kinase
**GK5**	Glycerol kinase 5 (putative)

EXCLI Journal 2021;20:126-141– ISSN 1611-2156
Received: December 08, 2020, accepted: January 11, 2021, published: January 18, 2021

**GKAP1**	G kinase anchoring protein 1
**GLDN**	Gliomedin
**GLG1**	Golgi glycoprotein 1
**GLMP**	Glycosylated lysosomal membrane protein
**GLS**	Glutaminase
**GLUL**	Glutamate-ammonia ligase
**GLUL**	Glutamate-ammonia ligase
**GLYR1///SEPT6**	Glyoxylate reductase 1 homolog///septin 6
**GMNN**	Geminin, DNA replication inhibitor
**GNAI1**	G protein subunit alpha i1
**GNB5**	G protein subunit beta 5
**GNL2**	G protein nucleolar 2
**GNRH2**	Gonadotropin releasing hormone 2
**GO**	Gene ontology
**GOLGA4**	Golgin A4
**GOLGA8A**	Golgin A8 family member A
**GOLT1B**	Golgi transport 1B
**GORAB**	Golgin, RAB6 interacting
**GPALPP1**	GPALPP motifs containing 1
**GPAM**	Glycerol-3-phosphate acyltransferase 1
**GPAM**	Glycerol-3-phosphate acyltransferase, mitochondrial
**GPATCH2**	G-patch domain containing 2
**GPR20**	G protein-coupled receptor 20
**GPR75-ASB3///ASB3///GPR75**	GPR75-ASB3 readthrough///ankyrin repeat and SOCS box containing 3///G protein-coupled receptor 75
**GPR78///CPZ**	G protein-coupled receptor 78///carboxypeptidase Z
**GPR89A///GPR89B**	G protein-coupled receptor 89A///G protein-coupled receptor 89B
**GPRASP1**	G protein-coupled receptor associated sorting protein 1
**GPRIN3**	GPRIN family member 3
**GPX3**	Glutathione peroxidase 3
**GPX7**	Glutathione peroxidase 7
**GRAMD3**	GRAM domain containing 3
**GRAP**	GRB2-related adaptor protein
**GRSF1**	G-rich RNA sequence binding factor 1
**GSAP**	Gamma-secretase activating protein
**GSDMB**	Gasdermin B
**GSN**	Gelsolin
**GSPT1**	G1 to S phase transition 1
**GTF2A2**	General transcription factor IIA subunit 2
**GTF2H2B///GTF2H2**	General transcription factor IIH subunit 2B (pseudogene)///general transcription factor IIH subunit 2
**GTPBP4**	GTP binding protein 4

EXCLI Journal 2021;20:126-141– ISSN 1611-2156
Received: December 08, 2020, accepted: January 11, 2021, published: January 18, 2021

**GUSBP3///SMA5///SMA4**	Glucuronidase, beta pseudogene 3///glucuronidase beta pseudogene///glucuronidase beta pseudogene
**GUSBP9///GUSBP3**	Glucuronidase, beta pseudogene 9///glucuronidase, beta pseudogene 3
**GVINP1**	Gtpase, very large interferon inducible pseudogene 1
**GXYLT1**	Glucoside xylosyltransferase 1
**GYPA**	Glycophorin A (MNS blood group)
**GYPB**	Glycophorin B (MNS blood group)
**GYPC**	Glycophorin C (Gerbich blood group)
**GYPC**	Glycophorin-C
**H2AFV**	H2A histone family member V
**H2AFX**	H2A histone family member X
**HAB1**	B1 for mucin
**HACE1**	HECT domain and ankyrin repeat containing E3 ubiquitin protein ligase 1
**HADH**	Hydroxyacyl-coa dehydrogenase
**HAUS1**	HAUS augmin like complex subunit 1
**HAUS2**	HAUS augmin like complex subunit 2
**HAUS3**	HAUS augmin like complex subunit 3
**HAUS6**	HAUS augmin like complex subunit 6
**HBA2///HBA1**	Hemoglobin subunit alpha 2///hemoglobin subunit alpha 1
**HBBP1**	Hemoglobin subunit beta pseudogene 1
**HBE1**	Hemoglobin subunit epsilon 1
**HBG2///HBG1**	Hemoglobin subunit gamma 2///hemoglobin subunit gamma 1
**HBM**	Hemoglobin subunit mu
**HBQ1**	Hemoglobin subunit theta 1
**HBS1L**	HBS1 like translational gtpase
**HBZ**	Hemoglobin subunit zeta
**HCG11**	HLA complex group 11 (non-protein coding)
**HCG18**	HLA complex group 18 (non-protein coding)
**HCG22**	HLA complex group 22
**HEATR1**	HEAT repeat containing 1
**HELLS**	Helicase, lymphoid-specific
**HELZ2**	Helicase with zinc finger 2
**HEMK1**	Hemk methyltransferase family member 1
**HERC2P7**	Hect domain and RLD 2 pseudogene 7
**HERC2P9///HERC2P2**	Hect domain and RLD 2 pseudogene 9///hect domain and RLD 2 pseudogene 2
**HERC4**	HECT and RLD domain containing E3 ubiquitin protein ligase 4
**HEXA**	Hexosaminidase subunit alpha
**HEY1**	Hes related family bhlh transcription factor with YRPW motif 1
**HEY1**	Hes related family bhlh transcription factor with YRPW motif 1
**HINT1**	Histidine triad nucleotide binding protein 1
**HINT3**	Histidine triad nucleotide binding protein 3
**HIPK1**	Homeodomain interacting protein kinase 1
**HIPK2**	Homeodomain interacting protein kinase 2

EXCLI Journal 2021;20:126-141– ISSN 1611-2156
Received: December 08, 2020, accepted: January 11, 2021, published: January 18, 2021

**HIST1H1D**	Histone cluster 1, H1d
**HKR1**	HKR1, GLI-Kruppel zinc finger family member
**HLA-DPA1**	Major histocompatibility complex, class II, DP alpha 1
**HLA-DRB4**	Major histocompatibility complex, class II, DR beta 4
**HLTF**	Helicase like transcription factor
**HLX**	H2.0 like homeobox
**HMBOX1**	Homeobox containing 1
**HMBS**	Hydroxymethylbilane synthase
**HMGB1**	High mobility group box 1
**HMGN3**	High mobility group nucleosomal binding domain 3
**HNMT**	Histamine N-methyltransferase
**HNRNPA2B1**	Heterogeneous nuclear ribonucleoprotein A2/B1
**HNRNPA3**	Heterogeneous nuclear ribonucleoprotein A3
**HNRNPC**	Heterogeneous nuclear ribonucleoprotein C (C1/C2)
**HNRNPDL**	Heterogeneous nuclear ribonucleoprotein D like
**HNRNPM**	Heterogeneous nuclear ribonucleoprotein M
**HNRNPU**	Heterogeneous nuclear ribonucleoprotein U
**HOOK1**	Hook microtubule tethering protein 1
**HOOK2**	Hook microtubule tethering protein 2
**HOOK3**	Hook microtubule tethering protein 3
**HOXB-AS1**	HOXB cluster antisense RNA 1
**HOXC10**	Homeobox C10
**HOXD9**	Homeobox D9
**HP1BP3**	Heterochromatin protein 1 binding protein 3
**HR**	Hair growth associated
**HS2ST1**	Heparan sulfate 2-O-sulfotransferase 1
**HSD17B14**	Hydroxysteroid 17-beta dehydrogenase 14
**HSD17B7**	Hydroxysteroid 17-beta dehydrogenase 7
**HSP90AA1**	Heat shock protein 90 alpha family class A member 1
**HSP90AB1**	Heat shock protein 90 alpha family class B member 1
**HSPA4**	Heat shock protein family A (Hsp70) member 4
**HSPB1**	Heat shock protein family B (small) member 1
**HSPB11**	Heat shock protein family B (small) member 11
**HSPD1**	Heat shock protein family D (Hsp60) member 1
**ICAM2**	Intercellular adhesion molecule 2
**ICE1**	Interactor of little elongation complex ELL subunit 1
**IDH1**	Isocitrate dehydrogenase (NADP(+)) 1, cytosolic
**IFI27**	Interferon alpha inducible protein 27
**IFI44**	Interferon induced protein 44
**IFNA21**	Interferon alpha 21
**IFT80**	Intraflagellar transport 80
**IGF2BP2**	Insulin like growth factor 2 mrna binding protein 2
**IGFALS**	Insulin like growth factor binding protein acid labile subunit
**IGFBP2**	Insulin like growth factor binding protein 2

EXCLI Journal 2021;20:126-141– ISSN 1611-2156
Received: December 08, 2020, accepted: January 11, 2021, published: January 18, 2021

**IGFBP3**	Insulin like growth factor binding protein 3
**IGFBP5**	Insulin like growth factor binding protein 5
**IGFBP7**	Insulin like growth factor binding protein 7
**IGHM**	Immunoglobulin heavy constant mu
**IGHM///IGHG1///IGHA1**	Immunoglobulin heavy constant mu///immunoglobulin heavy constant gamma 1 (G1m marker)///immunoglobulin heavy constant alpha 1
**IKZF2**	IKAROS family zinc finger 2
**IL11RA**	Interleukin 11 receptor subunit alpha
**IL13RA1**	Interleukin 13 receptor subunit alpha 1
**IL13RA1**	Interleukin 13 receptor subunit alpha 1
**IL1R2**	Interleukin 1 receptor type 2
**IL1RN**	Interleukin 1 receptor antagonist
**IL22**	Interleukin 22
**IL23A**	Interleukin 23 subunit alpha
**IL27RA**	Interleukin 27 receptor subunit alpha
**IL4R**	Interleukin 4 receptor
**IL6ST**	Interleukin 6 signal transducer
**ILDR1**	Immunoglobulin like domain containing receptor 1
**ILF3**	Interleukin enhancer binding factor 3
**IMMP1L**	Inner mitochondrial membrane peptidase subunit 1
**IMPACT**	Impact RWD domain protein
**ING5**	Inhibitor of growth family member 5
**INMT**	Indolethylamine N-methyltransferase
**INO80D**	INO80 complex subunit D
**INPP4A**	Inositol polyphosphate-4-phosphatase type I A
**INPP5B**	Inositol polyphosphate-5-phosphatase B
**INPP5F**	Inositol polyphosphate-5-phosphatase F
**INS-IGF2///IGF2**	INS-IGF2 readthrough///insulin like growth factor 2
**INTS6L**	Integrator complex subunit 6 like
**INVS///WSB1**	Inversin///WD repeat and SOCS box containing 1
**IPCEF1**	Interaction protein for cytohesin exchange factors 1
**IPO11-LRRC70///IPO11**	IPO11-LRRC70 readthrough///importin 11
**IPO5**	Importin 5
**IPO7**	Importin 7
**IQCE**	IQ motif containing E
**IRAK3**	Interleukin 1 receptor associated kinase 3
**IRF2BP2**	Interferon regulatory factor 2 binding protein 2
**IRF5**	Interferon regulatory factor 5
**IRF6**	Interferon regulatory factor 6
**ITGA3**	Integrin subunit alpha 3
**ITGAE**	Integrin subunit alpha E
**ITGB1**	Integrin subunit beta 1
**ITGB2-AS1**	ITGB2 antisense RNA 1
**ITGB3BP**	Integrin subunit beta 3 binding protein

EXCLI Journal 2021;20:126-141– ISSN 1611-2156
Received: December 08, 2020, accepted: January 11, 2021, published: January 18, 2021

**ITLN1**	Intelectin 1
**ITPR3**	Inositol 1,4,5-trisphosphate receptor type 3
**JAG1**	Jagged 1
**JAK2**	Janus kinase 2
**JAZF1**	JAZF zinc finger 1
**JMY**	Junction mediating and regulatory protein, p53 cofactor
**JPH2**	Junctophilin 2
**JPX**	JPX transcript, XIST activator (non-protein coding)
**JUN**	Jun proto-oncogene, AP-1 transcription factor subunit
**KAT6B**	Lysine acetyltransferase 6B
**KAZN**	Kazrin, periplakin interacting protein
**KBTBD12**	Kelch repeat and BTB domain containing 12
**KCNC4**	Potassium voltage-gated channel subfamily C member 4
**KCNIP2-AS1**	KCNIP2 antisense RNA 1
**KCNJ15**	Potassium voltage-gated channel subfamily J member 15
**KCNJ2**	Potassium voltage-gated channel subfamily J member 2
**KCNJ4**	Potassium voltage-gated channel subfamily J member 4
**KCNN2**	Potassium calcium-activated channel subfamily N member 2
**KCNQ3**	Potassium voltage-gated channel subfamily Q member 3
**KCTD5**	Potassium channel tetramerization domain containing 5
**KEGG**	Kyoto Encyclopedia of Gene and Genome
**KHSRP**	KH-type splicing regulatory protein
**KIAA1109**	Kiaa1109
**KIAA1147**	Kiaa1147
**KIAA1324**	Kiaa1324
**KIF21A**	Kinesin family member 21A
**KIFC2**	Kinesin family member C2
**KIFC3**	Kinesin family member C3
**KIZ**	Kizuna centrosomal protein
**KLC1**	Kinesin light chain 1
**KLC3**	Kinesin light chain 3
**KLF1**	Kruppel like factor 1
**KLF11**	Kruppel like factor 11
**KLF12**	Kruppel like factor 12
**KLHDC1**	Kelch domain containing 1
**KLHDC10**	Kelch domain containing 10
**KLHDC4**	Kelch domain containing 4
**KLHDC8A**	Kelch domain containing 8A
**KLHL14**	Kelch like family member 14
**KLHL3**	Kelch like family member 3
**KLHL34**	Kelch like family member 34
**KLHL5**	Kelch like family member 5
**KLK14**	Kallikrein related peptidase 14
**KMT5B**	Lysine methyltransferase 5B
**KPNA2**	Karyopherin subunit alpha 2

EXCLI Journal 2021;20:126-141– ISSN 1611-2156
Received: December 08, 2020, accepted: January 11, 2021, published: January 18, 2021

**KPNA4**	Karyopherin subunit alpha 4
**KPNA5**	Karyopherin subunit alpha 5
**KRBOX4**	KRAB box domain containing 4
**KRI1**	KRI1 homolog
**KRIT1**	KRIT1, ankyrin repeat containing
**KRT1**	Keratin 1
**KRT10**	Keratin 10
**KRT23**	Keratin 23
**KRT33B**	Keratin 33B
**KTN1**	Kinectin 1
**L3MBTL1**	L(3)mbt-like 1 (Drosophila)
**LACTB2**	Lactamase beta 2
**LAMB1**	Laminin subunit beta 1
**LAMP1**	Lysosomal associated membrane protein 1
**LAMP1**	Lysosomal associated membrane protein 1
**LARP1**	La ribonucleoprotein domain family member 1
**LARP6**	La ribonucleoprotein domain family member 6
**LARP7**	La ribonucleoprotein domain family member 7
**LATS1**	Large tumor suppressor kinase 1
**LEF1**	Lymphoid enhancer binding factor 1
**LEF1-AS1**	LEF1 antisense RNA 1
**LHX4-AS1**	LHX4 antisense RNA 1
**LIG4**	DNA ligase 4
**LIMA1**	LIM domain and actin binding 1
**LIMD1**	LIM domains containing 1
**LIN7A**	Lin-7 homolog A, crumbs cell polarity complex component
**LIN7C**	Lin-7 homolog C, crumbs cell polarity complex component
**LINC-PINT**	Long intergenic non-protein coding RNA, p53 induced transcript
**LINC00342**	Long intergenic non-protein coding RNA 342
**LINC00658**	Long intergenic non-protein coding RNA 658
**LINC00667**	Long intergenic non-protein coding RNA 667
**LINC00894**	Long intergenic non-protein coding RNA 894
**LINC00908**	Long intergenic non-protein coding RNA 908
**LINC00926**	Long intergenic non-protein coding RNA 926
**LINC00938**	Long intergenic non-protein coding RNA 938
**LINC00969**	Long intergenic non-protein coding RNA 969
**LINC00982**	Long intergenic non-protein coding RNA 982
**LINC01118**	Long intergenic non-protein coding RNA 1118
**LINC01133**	Long intergenic non-protein coding RNA 1133
**LINC01159**	Long intergenic non-protein coding RNA 1159
**LINC01215**	Long intergenic non-protein coding RNA 1215
**LINC01550**	Long intergenic non-protein coding RNA 1550
**LINC01620**	Long intergenic non-protein coding RNA 1620
**LINGO1**	Leucine rich repeat and Ig domain containing 1

EXCLI Journal 2021;20:126-141– ISSN 1611-2156
Received: December 08, 2020, accepted: January 11, 2021, published: January 18, 2021

**LLGL1**	LLGL1, scribble cell polarity complex component
**LLPH**	LLP homolog, long-term synaptic facilitation
**LMLN**	Leishmanolysin like peptidase
**LNPEP**	Leucyl and cystinyl aminopeptidase
**LNPK**	Lunapark, ER junction formation factor
**LOC100128108**	Putative ubiquitin-conjugating enzyme E2Q2-like protein
**LOC100128325**	Uncharacterized LOC100128325
**LOC100129129**	Uncharacterized LOC100129129
**LOC100131262**	Uncharacterized LOC100131262
**LOC100131541**	Uncharacterized LOC100131541
**LOC100132731///KIAA1257**	Uncharacterized LOC100132731///KIAA1257
**LOC100190986**	Uncharacterized LOC100190986
**LOC100287852///FAM136A**	Protein FAM136A pseudogene///family with sequence similarity 136 member A
**LOC100294033///TCAF1**	Protein FAM115A-like///TRPM8 channel associated factor 1
**LOC100499194**	Uncharacterized LOC100499194
**LOC100505938**	Uncharacterized LOC100505938
**LOC100506282**	Uncharacterized LOC100506282
**LOC100506538///NDUFAF6**	Uncharacterized LOC100506538///NADH:ubiquinone oxidoreductase complex assembly factor 6
**LOC100507281**	Uncharacterized LOC100507281
**LOC100507291**	Uncharacterized LOC100507291
**LOC100507403**	Uncharacterized LOC100507403
**LOC100507424///ITFG2**	Uncharacterized LOC100507424///integrin alpha FG-GAP repeat containing 2
**LOC100507431**	Uncharacterized LOC100507431
**LOC100509445///LOC728715///OVOS///OVOS2**	Uncharacterized LOC100509445///ovostatin homolog 2///ovostatin///ovostatin 2
**LOC100996740**	Uncharacterized LOC100996740
**LOC101060521///POLR3E**	DNA-directed RNA polymerase III subunit RPC5///RNA polymerase III subunit E
**LOC101060604///NPIPA5///SMG1P3///NPIPB5///SMG1P1///LOC613037///NPIPB4///SLC7A5P1///NPIPB3**	Putative L-type amino acid transporter 1-like protein IMAA///nuclear pore complex interacting protein family member A5///SMG1P3, nonsense mediated mrna decay associated PI3K related kinase pseudogene 3///nuclear pore complex interacting protein family mem
**LOC101060835///HLA-DQB1**	HLA class II histocompatibility antigen, DQ beta 1 chain-like///major histocompatibility complex, class II, DQ beta 1
**LOC101926967**	Uncharacterized LOC101926967
**LOC101926975**	Uncharacterized LOC101926975
**LOC101927069**	Uncharacterized LOC101927069
**LOC101927137///KIAA1456**	Uncharacterized LOC101927137///KIAA1456

EXCLI Journal 2021;20:126-141– ISSN 1611-2156
Received: December 08, 2020, accepted: January 11, 2021, published: January 18, 2021

**LOC101927608**	Uncharacterized LOC101927608
**LOC101928000**	Uncharacterized LOC101928000
**LOC101928054**	Uncharacterized LOC101928054
**LOC101928524///PRPF18**	Uncharacterized LOC101928524///pre-mrna processing factor 18
**LOC101928605///OR2A1-AS1///OR2A9P///OR2A20P**	Uncharacterized LOC101928605///OR2A1 antisense RNA 1///olfactory receptor family 2 subfamily A member 9 pseudogene///olfactory receptor family 2 subfamily A member 20 pseudogene
**LOC101928615///FNDC3B**	Uncharacterized LOC101928615///fibronectin type III domain containing 3B
**LOC101928623///HARS**	Uncharacterized LOC101928623///histidyl-trna synthetase
**LOC101928623///LOC401320**	Uncharacterized LOC101928623///uncharacterized LOC401320
**LOC101929047///HERC2P10///HERC2**	Uncharacterized LOC101929047///hect domain and RLD 2 pseudogene 10///HECT and RLD domain containing E3 ubiquitin protein ligase 2
**LOC101929165**	Uncharacterized LOC101929165
**LOC101929734**	Uncharacterized LOC101929734
**LOC101929787**	Uncharacterized LOC101929787
**LOC101930112///SPG7**	Uncharacterized LOC101930112///SPG7, paraplegin matrix AAA peptidase subunit
**LOC101930349///LOC101930344///CGNL1**	Uncharacterized LOC101930349///uncharacterized LOC101930344///cingulin like 1
**LOC101930363///LOC101928349///LOC100507387///FAM153C///FAM153A///FAM153B**	Uncharacterized LOC101930363///uncharacterized LOC101928349///uncharacterized LOC100507387///family with sequence similarity 153, member C///family with sequence similarity 153 member A///family with sequence similarity 153 member B
**LOC101930404///SNORD116-28///SNORD115-26///SNORD115-13///SNORD115-7///SNORD116-22///SNORD116-4///PWARSN///SNORD107///IPW**	Uncharacterized LOC101930404///small nucleolar RNA, C/D box 116-28///small nucleolar RNA, C/D box 115-26///small nucleolar RNA, C/D box 115-13///small nucleolar RNA, C/D box 115-7///small nucleolar RNA, C/D box 116-22///small nucleolar RNA, C/D box 116-4/
**LOC101930416///LOC101929792///LOC100996724///PDE4DIP**	Myomegalin-like///uncharacterized LOC101929792///phosphodiesterase 4D interacting protein-like///phosphodiesterase 4D interacting protein
**LOC101930489///MIR4435-2HG///LINC00152**	Uncharacterized LOC101930489///MIR4435-2 host gene///long intergenic non-protein coding RNA 152
**LOC101930583///GOLGA8B///GOLGA8A**	Uncharacterized LOC101930583///golgin A8 family member B///golgin A8 family member A

EXCLI Journal 2021;20:126-141– ISSN 1611-2156
Received: December 08, 2020, accepted: January 11, 2021, published: January 18, 2021

**LOC102606465**	Uncharacterized LOC102606465
**LOC102723526**	Uncharacterized LOC102723526
**LOC102723694**	Uncharacterized LOC102723694
**LOC102723709///LOC102723678///LOC101928102**	Uncharacterized LOC102723709///uncharacterized LOC102723678///putative uncharacterized protein LOC401522-like
**LOC102723897///MIR6859-1///MIR6859-2///LOC101930154///LOC100288778///WASH1///WASH7P///WASH2P///WASH3P**	WAS protein family homolog 2-like///microrna 6859-1///microrna 6859-2///WAS protein family homolog 6-like///WAS protein family homolog 1 pseudogene///WAS protein family homolog 1///WAS protein family homolog 7 pseudogene///WAS protein family homolog 2 pse
**LOC102724093///GOLGA6L4///GOLGA6L9///GOLGA6L5P**	Golgin subfamily A member 6-like protein 4///golgin A6 family-like 4///golgin A6 family-like 9///golgin A6 family-like 5, pseudogene
**LOC102724156**	Uncharacterized LOC102724156
**LOC102724200///TRAPPC10**	Trafficking protein particle complex subunit 10-like///trafficking protein particle complex 10
**LOC102724782**	Uncharacterized LOC102724782
**LOC102724814**	Uncharacterized LOC102724814
**LOC102724851**	Uncharacterized LOC102724851
**LOC102724951///LOC389831**	Uncharacterized LOC102724951///uncharacterized LOC389831
**LOC102724993///LOC101930075///NPIPA8///NPIPA7///NPIPA5///NPIPA2///NPIPA3///PKD1P1///NPIPA1**	Nuclear pore complex-interacting protein family member A7-like///poly-cystin-1-like///nuclear pore complex interacting protein family member A8///nuclear pore complex interacting protein family member A7///nu-clear pore complex interacting protein family me
**LOC105369595**	Uncharacterized LOC105369595
**LOC105369662**	Uncharacterized LOC105369662
**LOC105373738**	Uncharacterized LOC105373738
**LOC158402**	Uncharacterized LOC158402
**LOC171391**	Uncharacterized LOC171391
**LOC202025**	Uncharacterized LOC202025
**LOC202181**	SUMO interacting motifs containing 1 pseudogene
**LOC254896///TNFRSF10C**	Uncharacterized LOC254896///TNF receptor superfamily member 10c
**LOC283177**	Uncharacterized LOC283177
**LOC283335**	Uncharacterized LOC283335
**LOC283357**	Uncharacterized LOC283357
**LOC283588**	Uncharacterized LOC283588
**LOC283788**	FSHD region gene 1 pseudogene
**LOC285097**	Uncharacterized FLJ38379
**LOC339988**	Uncharacterized LOC339988

EXCLI Journal 2021;20:126-141– ISSN 1611-2156
Received: December 08, 2020, accepted: January 11, 2021, published: January 18, 2021

**LOC374443**	C-type lectin domain family 2 member D pseudogene
**LOC389765**	Kinesin family member 27 pseudogene
**LOC643072**	Uncharacterized LOC643072
**LOC728903///GLIDR**	Uncharacterized LOC728903///glioblastoma down-regulated RNA
**LOC730268///ANAPC1**	Anaphase-promoting complex subunit 1-like///anaphase promoting complex subunit 1
**LPIN1**	Lipin 1
**LPL**	Lipoprotein lipase
**LRCH3**	Leucine rich repeats and calponin homology domain containing 3
**LRCH4**	Leucine rich repeats and calponin homology domain containing 4
**LRFN2**	Leucine rich repeat and fibronectin type III domain containing 2
**LRIG2**	Leucine rich repeats and immunoglobulin like domains 2
**LRRC37B**	Leucine rich repeat containing 37B
**LRRC40**	Leucine rich repeat containing 40
**LRRN3**	Leucine rich repeat neuronal 3
**LRRN4**	Leucine rich repeat neuronal 3
**LSG1**	Large 60S subunit nuclear export gtpase 1
**LSM8**	LSM8 homolog, U6 small nuclear RNA associated
**LTBR**	Lymphotoxin beta receptor
**LUC7L**	LUC7 like
**LUC7L3**	LUC7 like 3 pre-mrna splicing factor
**LUC7L3**	Luc7-like protein 3
**LYRM7**	LYR motif containing 7
**LZTFL1**	Leucine zipper transcription factor like 1
**MACF1**	Microtubule-actin crosslinking factor 1
**MAFK**	MAF bzip transcription factor K
**MALT1**	MALT1 paracaspase
**MAN1C1**	Mannosidase alpha class 1C member 1
**MAN2A2**	Mannosidase alpha class 2A member 2
**MANSC1**	MANSC domain containing 1
**MAP1LC3A**	Microtubule associated protein 1 light chain 3 alpha
**MAP3K2**	Mitogen-activated protein kinase kinase kinase 2
**MAP3K4**	Mitogen-activated protein kinase kinase kinase 4
**MAP3K7CL**	MAP3K7 C-terminal like
**MAP3K8**	Mitogen-activated protein kinase kinase kinase 8
**MAP4K3**	Mitogen-activated protein kinase kinase kinase kinase 3
**MAP4K5**	Mitogen-activated protein kinase kinase kinase kinase 5
**MAP4K5**	Mitogen-activated protein kinase kinase kinase kinase 5
**MAP9**	Microtubule associated protein 9
**MAPK8**	Mitogen-activated protein kinase 8
**MAPKAPK5**	Mitogen-activated protein kinase-activated protein kinase 5
**MAPKAPK5-AS1**	MAPKAPK5 antisense RNA 1
**MAPT**	Microtubule associated protein tau

EXCLI Journal 2021;20:126-141– ISSN 1611-2156
Received: December 08, 2020, accepted: January 11, 2021, published: January 18, 2021

**MASTL**	Microtubule associated serine/threonine kinase like
**MAX**	MYC associated factor X
**MBD4**	Methyl-cpg binding domain 4, DNA glycosylase
**MBD5**	Methyl-cpg binding domain protein 5
**MBLAC2**	Metallo-beta-lactamase domain containing 2
**MBNL2**	Muscleblind like splicing regulator 2
**MBOAT2**	Membrane bound O-acyltransferase domain containing 2
**MBOAT7**	Membrane bound O-acyltransferase domain containing 7
**MBTPS2**	Membrane bound transcription factor peptidase, site 2
**MCM8**	Minichromosome maintenance 8 homologous recombination repair factor
**MCOLN2**	Mucolipin 2
**MCTS1**	MCTS1, re-initiation and release factor
**MDC1**	Mediator of DNA damage checkpoint 1
**MDFIC**	Myod family inhibitor domain containing
**MDM4**	MDM4, p53 regulator
**MDN1**	Midasin AAA atpase 1
**MED19**	Mediator complex subunit 19
**MED25**	Mediator complex subunit 25
**MED28**	Mediator complex subunit 28
**MED6**	Mediator complex subunit 6
**MED7**	Mediator complex subunit 7
**MEF2A**	Myocyte enhancer factor 2A
**MEG3**	Maternally expressed 3 (non-protein coding)
**MEGF6**	Multiple EGF like domains 6
**METAP2**	Methionyl aminopeptidase 2
**METTL10**	Methyltransferase like 10
**METTL14**	Methyltransferase like 14
**METTL17**	Methyltransferase like 17
**METTL21A**	Methyltransferase like 21A
**METTL3**	Methyltransferase like 3
**MF**	Molecular function
**MFNG**	MFNG O-fucosylpeptide 3-beta-N-acetylglucosaminyltransferase
**MFSD12**	Major facilitator superfamily domain containing 12
**MGA**	MGA, MAX dimerization protein
**MGAM**	Maltase-glucoamylase
**MGC12488**	Uncharacterized protein MGC12488
**MGC24103**	Uncharacterized MGC24103
**MIA3**	MIA family member 3, ER export factor
**MICALL1**	MICAL like 1
**MICU3**	Mitochondrial calcium uptake family member 3
**MIER3**	MIER family member 3
**MIGA1**	Mitoguardin 1
**MIOS**	Meiosis regulator for oocyte development

EXCLI Journal 2021;20:126-141– ISSN 1611-2156
Received: December 08, 2020, accepted: January 11, 2021, published: January 18, 2021

**MIR124-1///LINC00599**	Microrna 124-1///long intergenic non-protein coding RNA 599
**MIR155///MIR155HG**	Microrna 155///MIR155 host gene
**MIR21///VMP1**	Microrna 21///vacuole membrane protein 1
**MIR29C///MIR29B2**	Microrna 29c///microrna 29b-2
**MIR3682**	Microrna 3682
**MIR4680///PDCD4**	Microrna 4680///programmed cell death 4 (neoplastic transformation inhibitor)
**MIR4746///UBXN6**	Microrna 4746///UBX domain protein 6
**MIR612///NEAT1**	Microrna 612///nuclear paraspeckle assembly transcript 1 (non-protein coding)
**MIR6716///PHLDB1**	Microrna 6716///pleckstrin homology like domain family B member 1
**MIR6741///PYCR2**	Microrna 6741///pyrroline-5-carboxylate reductase family member 2
**MIR6785///TMEM94**	Microrna 6785///transmembrane protein 94
**MIR6845///NRBP2**	Microrna 6845///nuclear receptor binding protein 2
**MIR6884///MED24**	Microrna 6884///mediator complex subunit 24
**MIS18BP1**	MIS18 binding protein 1
**MISP**	Mitotic spindle positioning
**MKL2**	MKL1/myocardin like 2
**MKLN1**	Muskelin 1
**MLLT3**	MLLT3, super elongation complex subunit
**MLXIPL**	MLX interacting protein like
**MME**	Membrane metallo-endopeptidase
**MME**	Membrane metalloendopeptidase
**MME**	Neprilysin
**MMP25**	Matrix metallopeptidase 25
**MMP26**	Matrix metallopeptidase 26
**MMP9**	Matrix metallopeptidase 9
**MOAP1**	Modulator of apoptosis 1
**MON2**	MON2 homolog, regulator of endosome-to-Golgi trafficking
**MORN1**	MORN repeat containing 1
**MOSPD1**	Motile sperm domain containing 1
**MPHOSPH10**	M-phase phosphoprotein 10
**MPHOSPH9**	M-phase phosphoprotein 9
**MPO**	Myeloperoxidase
**MPZL1**	Myelin protein zero like 1
**MR1**	Major histocompatibility complex, class I-related
**MRC2**	Mannose receptor C type 2
**MREG**	Melanoregulin
**MRGPRG-AS1**	MRGPRG antisense RNA 1

EXCLI Journal 2021;20:126-141– ISSN 1611-2156
Received: December 08, 2020, accepted: January 11, 2021, published: January 18, 2021

**MRI1**	Methylthioribose-1-phosphate isomerase 1
**MRPL42**	Mitochondrial ribosomal protein L42
**MRPL46**	Mitochondrial ribosomal protein L46
**MRPL50**	Mitochondrial ribosomal protein L50
**MRPS14**	Mitochondrial ribosomal protein S14
**MRPS22**	Mitochondrial ribosomal protein S22
**MRPS25**	Mitochondrial ribosomal protein S25
**MRS2**	MRS2, magnesium transporter
**MRVI1**	Murine retrovirus integration site 1 homolog
**MS4A1**	Membrane spanning 4-domains A1
**MS4A14**	Membrane spanning 4-domains A14
**MSANTD2**	Myb/SANT DNA binding domain containing 2
**MSH5-SAPCD1///SAPCD1**	MSH5-SAPCD1 readthrough (NMD candidate)///suppressor APC domain containing 1
**MSI2**	Musashi RNA binding protein 2
**MSMO1**	Methylsterol monooxygenase 1
**MSS51**	MSS51 mitochondrial translational activator
**MTERF2**	Mitochondrial transcription termination factor 2
**MTERF3**	Mitochondrial transcription termination factor 3
**MTHFD1**	Methylenetetrahydrofolate dehydrogenase, cyclohydrolase and formyltetrahydrofolate synthetase 1
**MTHFR**	Methylenetetrahydrofolate reductase (NAD(P)H)
**MTIF2**	Mitochondrial translational initiation factor 2
**MTO1**	Mitochondrial trna translation optimization 1
**MTRF1**	Mitochondrial translational release factor 1
**MTX2**	Metaxin 2
**MTX3**	Metaxin 3
**MUC3**	Intestinal mucin-like
**MUC3A**	Mucin 3A, cell surface associated
**MUC4**	Mucin 4, cell surface associated
**MUC8**	Mucin 8
**MXD1**	MAX dimerization protein 1
**MYBL1**	MYB proto-oncogene like 1
**MYBL2**	MYB proto-oncogene like 2
**MYBPC1**	Myosin binding protein C, slow type
**MYH11**	Myosin heavy chain 11
**MYH7B**	Myosin heavy chain 7B
**MYL2**	Myosin light chain 2
**MYL4**	Myosin light chain 4
**MYO15B**	Myosin XVB
**MYO5A**	Myosin VA
**MYO9A**	Myosin IXA
**MYOD1**	Myogenic differentiation 1
**MYSM1**	Myb like, SWIRM and MPN domains 1

EXCLI Journal 2021;20:126-141– ISSN 1611-2156
Received: December 08, 2020, accepted: January 11, 2021, published: January 18, 2021

**MZT2B**	Mitotic spindle organizing protein 2B
**N4BP2**	NEDD4 binding protein 2
**N4BP2L2**	NEDD4 binding protein 2 like 2
**NAA16**	N(alpha)-acetyltransferase 16, nata auxiliary subunit
**NAA25**	N(alpha)-acetyltransferase 25, natb auxiliary subunit
**NAA40**	N(alpha)-acetyltransferase 40, natd catalytic subunit
**NADK2**	NAD kinase 2, mitochondrial
**NADSYN1**	NAD synthetase 1
**NAIP**	NLR family apoptosis inhibitory protein
**NAMPT**	Nicotinamide phosphoribosyltransferase
**NANOG**	Nanog homeobox
**NANOS1**	Nanos C2HC-type zinc finger 1
**NAP1L5**	Nucleosome assembly protein 1 like 5
**NAPEPLD**	N-acyl phosphatidylethanolamine phospholipase D
**NAV2**	Neuron navigator 2
**NBAT1**	Neuroblastoma associated transcript 1
**NBPF20**	Neuroblastoma breakpoint family member 20
**NCAPD3**	Non-SMC condensin II complex subunit D3
**NCBP2**	Nuclear cap binding protein subunit 2
**NCEH1**	Neutral cholesterol ester hydrolase 1
**NDRG2**	NDRG family member 2
**NDUFAF7**	NADH:ubiquinone oxidoreductase complex assembly factor 7
**NDUFB8**	NADH:ubiquinone oxidoreductase subunit B8
**NDUFS1**	NADH:ubiquinone oxidoreductase core subunit S1
**NEDD1**	Neural precursor cell expressed, developmentally down-regulated 1
**NEK1**	NIMA related kinase 1
**NEMF**	Nuclear export mediator factor
**NEMP1**	Nuclear envelope integral membrane protein 1
**NFATC2IP**	Nuclear factor of activated T-cells 2 interacting protein
**NFATC4**	Nuclear factor of activated T-cells 4
**NFE4**	Nuclear factor, erythroid 4
**NFIB**	Nuclear factor I B
**NFIX**	Nuclear factor I X
**NFKBIL1**	NFKB inhibitor like 1
**NFKBIZ**	NFKB inhibitor zeta
**NFYB**	Nuclear transcription factor Y subunit beta
**NHLRC2**	NHL repeat containing 2
**NHLRC3**	NHL repeat containing 3
**NHSL1**	NHS like 1
**NIFK-AS1**	NIFK antisense RNA 1
**NIN**	Ninein
**NIPAL2**	NIPA like domain containing 2
**NIPBL**	NIPBL, cohesin loading factor
**NKTR**	Natural killer cell triggering receptor

EXCLI Journal 2021;20:126-141– ISSN 1611-2156
Received: December 08, 2020, accepted: January 11, 2021, published: January 18, 2021

**NKX1-1**	NK1 homeobox 1
**NLN**	Neurolysin
**NLRC5**	NLR family CARD domain containing 5
**NLRP14**	NLR family pyrin domain containing 14
**NMRAL1**	Nmra like redox sensor 1
**NMRK2**	Nicotinamide riboside kinase 2
**NMT2**	N-myristoyltransferase 2
**NNT**	Nicotinamide nucleotide transhydrogenase
**NOL12**	Nucleolar protein 12
**NOL8**	Nucleolar protein 8
**NOM1**	Nucleolar protein with MIF4G domain 1
**NOP58**	NOP58 ribonucleoprotein
**NORAD**	Non-coding RNA activated by DNA damage
**NOV**	Nephroblastoma overexpressed
**NPAT**	Nuclear protein, coactivator of histone transcription
**NPBWR1**	Neuropeptides B/W receptor 1
**NPHP3**	Nephronophthisis 3 (adolescent)
**NPIPA5///NPIPB6///NPIPB8///NPIPB3**	Nuclear pore complex interacting protein family member A5///nuclear pore complex interacting protein family member B6///nuclear pore complex interacting protein family member B8///nuclear pore complex interacting protein family member B3
**NPR3**	Natriuretic peptide receptor 3
**NPRL3**	NPR3 like, GATOR1 complex subunit
**NPTN-IT1**	NPTN intronic transcript 1
**NR2C1**	Nuclear receptor subfamily 2 group C member 1
**NRIP1**	Nuclear receptor interacting protein 1
**NRP2**	Neuropilin 2
**NSD1**	Nuclear receptor binding SET domain protein 1
**NSG1**	Neuron specific gene family member 1
**NSMCE4A**	NSE4 homolog A, SMC5-SMC6 complex component
**NSRP1**	Nuclear speckle splicing regulatory protein 1
**NSUN5**	NOP2/Sun RNA methyltransferase family member 5
**NSUN5P1**	NOP2/Sun RNA methyltransferase family member 5 pseudogene 1
**NSUN5P2///NSUN5P1**	NOP2/Sun RNA methyltransferase family member 5 pseudogene 2///NOP2/Sun RNA methyltransferase family member 5 pseudogene 1
**NSUN6**	NOP2/Sun RNA methyltransferase family member 6
**NSUN7**	NOP2/Sun RNA methyltransferase family member 7
**NUCKS1**	Nuclear casein kinase and cyclin dependent kinase substrate 1
**NUDCD1**	Nudc domain containing 1
**NUDT14**	Nudix hydrolase 14
**NUMBL**	NUMB like, endocytic adaptor protein
**NUP160**	Nucleoporin 160
**NUP210**	Nucleoporin 210
**NUP35**	Nucleoporin 35
**NUP54**	Nucleoporin 54

EXCLI Journal 2021;20:126-141– ISSN 1611-2156
Received: December 08, 2020, accepted: January 11, 2021, published: January 18, 2021

**NUTM2B**	NUT family member 2B
**NUTM2B-AS1**	NUTM2B antisense RNA 1
**NVL**	Nuclear VCP-like
**OAZ2**	Ornithine decarboxylase antizyme 2
**OBFC1**	Oligonucleotide/oligosaccharide binding fold containing 1
**OBSCN**	Obscurin, cytoskeletal calmodulin and titin-interacting rhogef
**OCLN**	Occludin
**ODC1**	Ornithine decarboxylase 1
**ODF2L**	Outer dense fiber of sperm tails 2 like
**ODF3B**	Outer dense fiber of sperm tails 3B
**OFD1**	OFD1, centriole and centriolar satellite protein
**OGFOD1**	2-oxoglutarate and iron dependent oxygenase domain containing 1
**OGG1**	8-oxoguanine DNA glycosylase
**OGT**	O-linked N-acetylglucosamine (glcnac) transferase
**OMA1**	OMA1 zinc metallopeptidase
**OPRL1**	Opioid related nociceptin receptor 1
**OR10D1P**	Olfactory receptor family 10 subfamily D member 1 pseudogene
**OR2S2**	Olfactory receptor family 2 subfamily S member 2 (gene/pseudogene)
**OR3A3///OR1E2///OR1E1**	Olfactory receptor family 3 subfamily A member 3///olfactory receptor family 1 subfamily E member 2///olfactory receptor family 1 subfamily E member 1
**OR4D1**	Olfactory receptor family 4 subfamily D member 1
**ORAI2**	ORAI calcium release-activated calcium modulator 2
**ORM2///ORM1**	Orosomucoid 2///orosomucoid 1
**OSBP**	Oxysterol binding protein
**OSBP2**	Oxysterol binding protein 2
**OSBPL3**	Oxysterol binding protein like 3
**OSGIN2**	Oxidative stress induced growth inhibitor family member 2
**OSM**	Oncostatin M
**OTUD6B**	OTU domain containing 6B
**OTULIN**	OTU deubiquitinase with linear linkage specificity
**OVCH1-AS1**	OVCH1 antisense RNA 1
**OXR1**	Oxidation resistance 1
**P2RX5-TAX1BP3///TAX1BP3///P2RX5**	P2RX5-TAX1BP3 readthrough (NMD candidate)///Tax1 binding protein 3///purinergic receptor P2X 5
**P2RY12**	Purinergic receptor P2Y12
**P3H1**	Prolyl 3-hydroxylase 1
**PAAF1**	Proteasomal atpase associated factor 1
**PABPC1L**	Poly(A) binding protein cytoplasmic 1 like
**PABPN1**	Poly(A) binding protein nuclear 1
**PAIP1**	Poly(A) binding protein interacting protein 1
**PAK4**	P21 (RAC1) activated kinase 4
**PAPOLA**	Poly(A) polymerase alpha
**PAPOLG**	Poly(A) polymerase gamma
**PAQR8**	Progestin and adipoq receptor family member 8

EXCLI Journal 2021;20:126-141– ISSN 1611-2156
Received: December 08, 2020, accepted: January 11, 2021, published: January 18, 2021

**PARP15**	Poly(ADP-ribose) polymerase family member 15
**PART1**	Prostate androgen-regulated transcript 1 (non-protein coding)
**PAX1**	Paired box 1
**PAX5**	Paired box 5
**PAX8-AS1**	PAX8 antisense RNA 1
**PAXBP1**	PAX3 and PAX7 binding protein 1
**PAXBP1-AS1**	PAXBP1 antisense RNA 1
**PBRM1**	Polybromo 1
**PCBP1-AS1**	PCBP1 antisense RNA 1
**PCDHB11**	Protocadherin beta 11
**PCDHB13**	Protocadherin beta 13
**PCF11**	PCF11 cleavage and polyadenylation factor subunit
**PCGF3**	Polycomb group ring finger 3
**PCGF5**	Polycomb group ring finger 5(
**PCGF5**	Polycomb group ring finger 5
**PCM1**	Pericentriolar material 1
**PCMTD1**	Protein-L-isoaspartate (D-aspartate) O-methyltransferase domain containing 1
**PCNP**	PEST proteolytic signal containing nuclear protein
**PCNX1**	Pecanex homolog 1 (Drosophila)
**PCNX2**	Pecanex homolog 2 (Drosophila)
**PCNX4**	Pecanex homolog 4 (Drosophila)
**PCOLCE**	Procollagen C-endopeptidase enhancer
**PDCD2**	Programmed cell death 2
**PDE3B**	Phosphodiesterase 3B
**PDE4B**	Phosphodiesterase 4B
**PDE7A**	Phosphodiesterase 7A
**PDE8A**	Phosphodiesterase 8A
**PDHA1**	Pyruvate dehydrogenase (lipoamide) alpha 1
**PDIA6**	Protein disulfide isomerase family A member 6
**PDK2**	Pyruvate dehydrogenase kinase 2
**PDK4**	Pyruvate dehydrogenase kinase 4
**PDLIM5**	PDZ and LIM domain 5
**PDZD8**	PDZ domain containing 8
**PDZK1IP1**	PDZK1 interacting protein 1
**PEA15**	Phosphoprotein enriched in astrocytes 15
**PER2**	Period circadian clock 2
**PERM1**	PPARGC1 and ESRR induced regulator, muscle 1
**PEX3**	Peroxisomal biogenesis factor 3
**PFKFB3**	6-phosphofructo-2-kinase/fructose-2,6-biphosphatase 3
**PGAP1**	Post-GPI attachment to proteins 1
**PGGHG**	Protein-glucosylgalactosylhydroxylysine glucosidase
**PGK1**	Phosphoglycerate kinase 1
**PGLYRP1**	Peptidoglycan recognition protein 1

EXCLI Journal 2021;20:126-141– ISSN 1611-2156
Received: December 08, 2020, accepted: January 11, 2021, published: January 18, 2021

**PGRMC1**	Progesterone receptor membrane component 1
**PHBP19**	Prohibitin pseudogene 19
**PHC3**	Polyhomeotic homolog 3
**PHF14**	PHD finger protein 14
**PHF6**	PHD finger protein 6
**PHIP**	Pleckstrin homology domain interacting protein
**PHLDB2**	Pleckstrin homology like domain family B member 2
**PHOSPHO1**	Phosphoethanolamine/phosphocholine phosphatase
**PHOSPHO2**	Phosphatase, orphan 2
**PI3**	Peptidase inhibitor 3
**PIAS2**	Protein inhibitor of activated STAT 2
**PIGC**	Phosphatidylinositol glycan anchor biosynthesis class C
**PIGH**	Phosphatidylinositol glycan anchor biosynthesis class H
**PIGQ**	Phosphatidylinositol glycan anchor biosynthesis class Q
**PIGW**	Phosphatidylinositol glycan anchor biosynthesis class W
**PIK3AP1**	Phosphoinositide-3-kinase adaptor protein 1
**PIK3C2A**	Phosphatidylinositol-4-phosphate 3-kinase catalytic subunit type 2 alpha
**PIK3C3**	Phosphatidylinositol 3-kinase catalytic subunit type 3
**PINK1**	PTEN induced putative kinase 1
**PIP5KL1**	Phosphatidylinositol-4-phosphate 5-kinase like 1
**PITHD1**	PITH domain containing 1
**PITPNC1**	Phosphatidylinositol transfer protein, cytoplasmic 1
**PITPNC1**	Phosphatidylinositol transfer protein, cytoplasmic 1
**PITX2**	Paired like homeodomain 2
**PKD2**	Polycystin 2, transient receptor potential cation channel
**PKM**	Pyruvate kinase, muscle
**PLA2G12A**	Phospholipase A2 group XIIA
**PLA2G12B**	Phospholipase A2 group XIIB
**PLA2G7**	Phospholipase A2 group VII
**PLAG1**	PLAG1 zinc finger
**PLCB1**	Phospholipase C beta 1
**PLCG1**	Phospholipase C gamma 1
**PLCH2**	Phospholipase C eta 2
**PLCXD2**	Phosphatidylinositol specific phospholipase C X domain containing 2
**PLEK2**	Pleckstrin 2
**PLEKHA8**	Pleckstrin homology domain containing A8
**PLGLB1///PLGLB2**	Plasminogen-like B1///plasminogen-like B2
**PLPP5**	Phospholipid phosphatase 5
**PLXDC1**	Plexin domain containing 1
**PLXNA1**	Plexin A1
**PLXNB1**	Plexin B1
**PLXNC1**	Plexin C1
**PM20D2**	Peptidase M20 domain containing 2

**PMS1**	PMS1 homolog 1, mismatch repair system component
**PMS2CL///PMS2**	PMS2 C-terminal like pseudogene///PMS1 homolog 2, mismatch repair system component
**PNISR**	PNN interacting serine and arginine rich protein
**PNMA2**	Paraneoplastic Ma antigen 2
**PNN**	Pinin
**PNN**	Pinin, desmosome associated protein
**PNPLA2**	Patatin like phospholipase domain containing 2
**POLH**	DNA polymerase eta
**POLL**	Polymerase (DNA) lambda
**POLR2J4**	RNA polymerase II subunit J4, pseudogene
**POMT1**	Protein O-mannosyltransferase 1
**POU2AF1**	POU class 2 associating factor 1
**POU2F2**	POU class 2 homeobox 2
**POU6F1**	POU class 6 homeobox 1
**PPA2**	Pyrophosphatase (inorganic) 2
**PPARA**	Peroxisome proliferator activated receptor alpha
**PPAT**	Phosphoribosyl pyrophosphate amidotransferase
**PPFIA1**	PTPRF interacting protein alpha 1
**PPHLN1**	Periphilin 1
**PPI**	Protein-protein interaction
**PPIF**	Peptidylprolyl isomerase F
**PPL**	Periplakin
**PPM1H**	Protein phosphatase, Mg2+/Mn2+ dependent 1H
**PPM1J**	Protein phosphatase, Mg2+/Mn2+ dependent 1J
**PPM1K**	Protein phosphatase, Mg2+/Mn2+ dependent 1K
**PPP1R1B**	Protein phosphatase 1 regulatory inhibitor subunit 1B
**PPP1R2**	Protein phosphatase 1 regulatory inhibitor subunit 2
**PPP1R21**	Protein phosphatase 1 regulatory subunit 21
**PPP1R26-AS1**	PPP1R26 antisense RNA 1
**PPP1R3A**	Protein phosphatase 1 regulatory subunit 3A
**PPP1R3E**	Protein phosphatase 1 regulatory subunit 3E
**PPP2R2D**	Protein phosphatase 2 regulatory subunit Bdelta
**PPP2R5B**	Protein phosphatase 2 regulatory subunit b'beta
**PPP3CC**	Protein phosphatase 3 catalytic subunit gamma
**PPP3R1**	Protein phosphatase 3 regulatory subunit B, alpha
**PPP4R1L**	Protein phosphatase 4 regulatory subunit 1 like (pseudogene)
**PPP6R2**	Protein phosphatase 6 regulatory subunit 2
**PPP6R3**	Protein phosphatase 6 regulatory subunit 3
**PPWD1**	Peptidylprolyl isomerase domain and WD repeat containing 1
**PQLC3**	PQ loop repeat containing 3
**PRDM15**	PR/SET domain 15
**PRDX6**	Peroxiredoxin 6
**PRICKLE1**	Prickle planar cell polarity protein 1

EXCLI Journal 2021;20:126-141– ISSN 1611-2156
Received: December 08, 2020, accepted: January 11, 2021, published: January 18, 2021

**PRKAA1**	Protein kinase AMP-activated catalytic subunit alpha 1
**PRKAB2**	Protein kinase AMP-activated non-catalytic subunit beta 2
**PRKACA**	Protein kinase camp-activated catalytic subunit alpha
**PRKACB**	Protein kinase camp-activated catalytic subunit beta
**PRKCI**	Protein kinase C iota
**PRKCQ-AS1**	PRKCQ antisense RNA 1
**PRKD3**	Protein kinase D3
**PRKXP1**	Protein kinase, X-linked, pseudogene 1
**PRMT2**	Protein arginine methyltransferase 2
**PRMT5**	Protein arginine methyltransferase 5
**PRMT8**	Protein arginine methyltransferase 8
**PRO0471**	Uncharacterized LOC28994
**PROSC**	Proline synthetase cotranscribed homolog (bacterial)
**PRPF38B**	Pre-mrna processing factor 38B
**PRPF39**	Pre-mrna processing factor 39
**PRPF40A**	Pre-mrna processing factor 40 homolog A
**PRPF4B**	Pre-mrna processing factor 4B
**PRR14L**	Proline rich 14 like
**PRR16**	Proline rich 16
**PRR35**	Proline rich 35
**PRSS23**	Protease, serine 23
**PRSS33**	Protease, serine 33
**PSD4**	Pleckstrin and Sec7 domain containing 4
**PSMA5**	Proteasome subunit alpha 5
**PSMB2**	Proteasome subunit beta 2
**PSMD5-AS1**	PSMD5 antisense RNA 1 (head to head)
**PSPC1**	Paraspeckle component 1
**PTBP2**	Polypyrimidine tract binding protein 2
**PTCH1**	Patched 1
**PTGDR2**	Prostaglandin D2 receptor 2
**PTGR1**	Prostaglandin reductase 1
**PTGS2**	Prostaglandin-endoperoxide synthase 2
**PTMS**	Parathymosin
**PTP4A1**	Protein tyrosine phosphatase type IVA, member 1
**PTPN11**	Protein tyrosine phosphatase, non-receptor type 11
**PTPN14**	Protein tyrosine phosphatase, non-receptor type 14
**PTPN22**	Protein tyrosine phosphatase, non-receptor type 22
**PTPRN2**	Protein tyrosine phosphatase, receptor type N2
**PTRF**	Polymerase I and transcript release factor
**PUS7**	Pseudouridylate synthase 7 (putative)
**PWAR6**	Prader Willi/Angelman region RNA 6
**PWWP2A**	PWWP domain containing 2A
**PWWP2B**	PWWP domain containing 2B
**PXDN**	Peroxidasin

EXCLI Journal 2021;20:126-141– ISSN 1611-2156
Received: December 08, 2020, accepted: January 11, 2021, published: January 18, 2021

**PXYLP1**	2-phosphoxylose phosphatase 1
**PYHIN1**	Pyrin and HIN domain family member 1
**PYROXD1**	Pyridine nucleotide-disulphide oxidoreductase domain 1
**PYROXD2**	Pyridine nucleotide-disulphide oxidoreductase domain 2
**PYY**	Peptide YY
**QKI**	QKI, KH domain containing RNA binding
**QPCT**	Glutaminyl-peptide cyclotransferase
**QPCTL**	Glutaminyl-peptide cyclotransferase like
**R3HDM4**	R3H domain containing 4
**RAB27A**	RAB27A, member RAS oncogene family
**RAB29**	RAB29, member RAS oncogene family
**RAB39B**	RAB39B, member RAS oncogene family
**RABEP2**	Rabaptin, RAB gtpase binding effector protein 2
**RAC1**	Ras-related C3 botulinum toxin substrate 1 (rho family, small GTP binding protein Rac1)
**RAD23B**	RAD23 homolog B, nucleotide excision repair protein
**RAD50**	RAD50 double strand break repair protein
**RAD51-AS1**	RAD51 antisense RNA 1 (head to head)
**RAI1**	Retinoic acid induced 1
**RALB**	RALB Ras like proto-oncogene B
**RALGAPA1**	Ral gtpase activating protein catalytic alpha subunit 1
**RALGAPA2**	Ral gtpase activating protein catalytic alpha subunit 2
**RALGAPB**	Ral gtpase activating protein non-catalytic beta subunit
**RALGPS2**	Ral GEF with PH domain and SH3 binding motif 2
**RANBP2**	RAN binding protein 2
**RANBP9**	RAN binding protein 9
**RAP1GAP2**	RAP1 gtpase activating protein 2
**RAP2A**	RAP2A, member of RAS oncogene family
**RAPH1**	Ras association (ralgds/AF-6) and pleckstrin homology domains 1
**RASGEF1A**	Rasgef domain family member 1A
**RASGEF1B**	Rasgef domain family member 1B
**RASGRF2**	Ras protein specific guanine nucleotide releasing factor 2
**RASGRP1**	RAS guanyl releasing protein 1
**RB1CC1**	RB1 inducible coiled-coil 1
**RBBP4**	RB binding protein 4, chromatin remodeling factor
**RBBP6**	RB binding protein 6, ubiquitin ligase
**RBM14-RBM4///RBM4**	RBM14-RBM4 readthrough///RNA binding motif protein 4
**RBM15**	RNA binding motif protein 15
**RBM26**	RNA binding motif protein 26
**RBM33**	RNA binding motif protein 33
**RBM39**	RNA binding motif protein 39
**RBM4**	RNA binding motif protein 4
**RBM41**	RNA binding motif protein 41
**RBM6**	RNA binding motif protein 6

**RBP2**	Retinol binding protein 2
**RBPJL**	Recombination signal binding protein for immunoglobulin kappa J region like
**RCBTB1**	RCC1 and BTB domain containing protein 1
**RCOR3**	REST corepressor 3
**RECK**	Reversion inducing cysteine rich protein with kazal motifs
**REPS1**	RALBP1 associated Eps domain containing 1
**REST**	RE1 silencing transcription factor
**RETNLB**	Resistin like beta
**REV3L**	REV3 like, DNA directed polymerase zeta catalytic subunit
**RFX7**	Regulatory factor X7
**RFXAP**	Regulatory factor X associated protein
**RGPD6///RGPD8///RGPD3///RGPD4///RGPD5**	RANBP2-like and GRIP domain containing 6///RANBP2-like and GRIP domain containing 8///RANBP2-like and GRIP domain containing 3///RANBP2-like and GRIP domain containing 4///RANBP2-like and GRIP domain containing 5
**RGR**	Retinal G protein coupled receptor
**RGS9BP**	Regulator of G-protein signaling 9 binding protein
**RHAG**	Rh-associated glycoprotein
**RHD**	Rh blood group D antigen
**RHD///RHCE**	Rh blood group D antigen///Rh blood group ccee antigens
**RHEB**	Ras homolog enriched in brain
**RHOA**	Ras homolog family member A
**RHOBTB3**	Rho related BTB domain containing 3
**RHOBTB3**	Rho related BTB domain containing 3
**RHOQ**	Ras homolog family member Q
**RIF1**	Replication timing regulatory factor 1
**RILP**	Rab interacting lysosomal protein
**RIOK1**	RIO kinase 1
**RIT1**	Ras like without CAAX 1
**RNASE3**	Ribonuclease A family member 3
**RND3**	Rho family gtpase 3
**RNF138**	Ring finger protein 138
**RNF146**	Ring finger protein 146
**RNF157**	Ring finger protein 157
**RNF157-AS1**	RNF157 antisense RNA 1
**RNF170**	Ring finger protein 170
**RNF182**	Ring finger protein 182
**RNMT**	RNA guanine-7 methyltransferase
**RNPC3**	RNA binding region (RNP1, RRM) containing 3
**RORA**	RAR related orphan receptor A
**RPRD1A**	Regulation of nuclear pre-mrna domain containing 1A
**RPRD2**	Regulation of nuclear pre-mrna domain containing 2
**RPS27L**	Ribosomal protein S27 like
**RPS6KB1**	Ribosomal protein S6 kinase B1

EXCLI Journal 2021;20:126-141– ISSN 1611-2156
Received: December 08, 2020, accepted: January 11, 2021, published: January 18, 2021

**RRAS2**	Related RAS viral (r-ras) oncogene homolog 2
**RREB1**	Ras responsive element binding protein 1
**RRN3P1**	RRN3 homolog, RNA polymerase I transcription factor pseudogene 1
**RRP15**	Ribosomal RNA processing 15 homolog
**RSF1**	Remodeling and spacing factor 1
**RSPH6A**	Radial spoke head 6 homolog A
**RSRP1**	Arginine and serine rich protein 1
**RTN4IP1**	Reticulon 4 interacting protein 1
**RUFY2**	RUN and FYVE domain containing 2
**RUFY3**	RUN and FYVE domain containing 3
**RUNDC3A**	RUN domain containing 3A
**RUSC1-AS1**	RUSC1 antisense RNA 1
**S100A9**	S100 calcium binding protein A9
**S100PBP**	S100P binding protein
**S1PR3**	Sphingosine-1-phosphate receptor 3
**SAA2-SAA4///SAA2///SAA1**	SAA2-SAA4 readthrough///serum amyloid A2///serum amyloid A1
**SACS**	Sacsin molecular chaperone
**SAMD9L**	Sterile alpha motif domain containing 9 like
**SAMM50**	SAMM50 sorting and assembly machinery component
**SAP30L**	SAP30 like
**SART3**	Squamous cell carcinoma antigen recognized by T-cells 3
**SATB1**	SATB homeobox 1
**SBNO1**	Strawberry notch homolog 1
**SCAF4**	SR-related CTD associated factor 4
**SCAI**	Suppressor of cancer cell invasion
**SCAMP1**	Secretory carrier membrane protein 1
**SCARNA13///SNHG10**	Small Cajal body-specific RNA 13///small nucleolar RNA host gene 10
**SCARNA15**	Small Cajal body-specific RNA 15
**SCD**	Acyl-coa desaturase
**SCD**	Stearoyl-coa desaturase
**SCML4**	Sex comb on midleg-like 4 (Drosophila)
**SCN3A**	Sodium voltage-gated channel alpha subunit 3
**SCOC**	Short coiled-coil protein
**SCRN3**	Secernin 3
**SCYL2**	SCY1 like pseudokinase 2
**SCYL3**	SCY1 like pseudokinase 3
**SDHAP2///LINC00969///SDHAP1**	Succinate dehydrogenase complex flavoprotein subunit A pseudogene 2///long intergenic non-protein coding RNA 969///succinate dehydrogenase complex flavoprotein subunit A pseudogene 1
**SDHAP3///RGS8**	Succinate dehydrogenase complex flavoprotein subunit A pseudogene 3///regulator of G-protein signaling 8
**SDHC**	Succinate dehydrogenase complex subunit C
**SDR39U1**	Short chain dehydrogenase/reductase family 39U member 1

EXCLI Journal 2021;20:126-141– ISSN 1611-2156
Received: December 08, 2020, accepted: January 11, 2021, published: January 18, 2021

**SEC14L4**	SEC14 like lipid binding 4
**SEC22A**	SEC22 homolog A, vesicle trafficking protein
**SELENBP1**	Selenium binding protein 1
**SEMA6A**	Semaphorin 6A
**SENP7**	SUMO1/sentrin specific peptidase 7
**SEPSECS**	Sep (O-phosphoserine) trna:Sec (selenocysteine) trna synthase
**SERPINB9**	Serpin family B member 9
**SERPINE2**	Serpin family E member 2
**SESN1**	Sestrin 1
**SESN3**	Sestrin 3
**SETD2**	SET domain containing 2
**SETD4**	SET domain containing 4
**SETD5**	SET domain containing 5
**SETSIP///SETP4 ///SET**	SET-like protein///SET pseudogene 4///SET nuclear proto-oncogene
**SF3A3**	Splicing factor 3a subunit 3
**SFPQ**	Splicing factor proline and glutamine rich
**SFR1**	SWI5 dependent homologous recombination repair protein 1
**SFT2D3///WDR3 3**	SFT2 domain containing 3///WD repeat domain 33
**SFXN1**	Sideroflexin 1
**SGK494**	Uncharacterized serine/threonine-protein kinase sgk494
**SGSM2**	Small G protein signaling modulator 2
**SH3GL1P2**	SH3 domain containing GRB2 like 1, endophilin A2 pseudogene 2
**SHISA7**	Shisa family member 7
**SHROOM3**	Shroom family member 3
**SHTN1**	Shootin 1
**SIN3A**	SIN3 transcription regulator family member A
**SIPA1L3**	Signal induced proliferation associated 1 like 3
**SIRPB1**	Signal regulatory protein beta 1
**SKP2**	S-phase kinase-associated protein 2, E3 ubiquitin protein ligase
**SLAIN2**	SLAIN motif family member 2
**SLAMF8**	SLAM family member 8
**SLC11A2**	Solute carrier family 11 member 2
**SLC12A2**	Solute carrier family 12 member 2
**SLC15A2**	Solute carrier family 15 member 2
**SLC16A10**	Solute carrier family 16 member 10
**SLC16A7**	Solute carrier family 16 member 7
**SLC19A2**	Solute carrier family 19 member 2
**SLC1A2**	Solute carrier family 1 member 2
**SLC20A2**	Solute carrier family 20 member 2
**SLC22A11**	Solute carrier family 22 member 11
**SLC22A4**	Solute carrier family 22 member 4
**SLC24A4**	Solute carrier family 24 member 4
**SLC25A16**	Solute carrier family 25 member 16

**SLC25A32**	Solute carrier family 25 member 32
**SLC25A37**	Solute carrier family 25 member 37
**SLC25A46**	Solute carrier family 25 member 46
**SLC26A11**	Solute carrier family 26 member 11
**SLC29A4**	Solute carrier family 29 member 4
**SLC2A1**	Solute carrier family 2 member 1
**SLC33A1**	Solute carrier family 33 member 1
**SLC35A2**	Solute carrier family 35 member A2
**SLC38A9**	Solute carrier family 38 member 9
**SLC39A2**	Solute carrier family 39 member 2
**SLC44A1**	Solute carrier family 44 member 1
**SLC45A1**	Solute carrier family 45 member 1
**SLC46A3**	Solute carrier family 46 member 3
**SLC48A1**	Solute carrier family 48 member 1
**SLC4A1**	Solute carrier family 4 member 1 (Diego blood group)
**SLC4A7**	Solute carrier family 4 member 7
**SLC5A3**	Solute carrier family 5 member 3
**SLC6A16**	Solute carrier family 6 member 16
**SLC6A8**	Solute carrier family 6 member 8
**SLC7A6OS**	Solute carrier family 7 member 6 opposite strand
**SLC8A1**	Solute carrier family 8 member A1
**SLC9B2**	Solute carrier family 9 member B2
**SLF2**	SMC5-SMC6 complex localization factor 2
**SLK**	STE20 like kinase
**SLX4IP**	SLX4 interacting protein
**SMA4**	Glucuronidase beta pseudogene
**SMAD6**	SMAD family member 6
**SMAD9**	SMAD family member 9
**SMARCA2**	SWI/SNF related, matrix associated, actin dependent regulator of chromatin, subfamily a, member 2
**SMARCA4**	SWI/SNF related, matrix associated, actin dependent regulator of chromatin, subfamily a, member 4
**SMARCAD1**	SWI/SNF-related, matrix-associated actin-dependent regulator of chromatin, subfamily a, containing DEAD/H box 1
**SMARCD3**	SWI/SNF related, matrix associated, actin dependent regulator of chromatin, subfamily d, member 3
**SMC3**	Structural maintenance of chromosomes 3
**SMCHD1**	Structural maintenance of chromosomes flexible hinge domain containing 1
**SMG1P5///BOLA2///SMG1P2**	SMG1P5, nonsense mediated mrna decay associated PI3K related kinase pseudogene 5///bola family member 2///SMG1P2, nonsense mediated mrna decay associated PI3K related kinase pseudogene 2
**SMIM1**	Small integral membrane protein 1 (Vel blood group)
**SMIM11A**	Small integral membrane protein 11A
**SMIM14**	Small integral membrane protein 14
**SMIM24**	Small integral membrane protein 24

EXCLI Journal 2021;20:126-141– ISSN 1611-2156
Received: December 08, 2020, accepted: January 11, 2021, published: January 18, 2021

**SMIM7**	Small integral membrane protein 7
**SMOC2**	SPARC related modular calcium binding 2
**SMOX**	Spermine oxidase
**SMURF2**	SMAD specific E3 ubiquitin protein ligase 2
**SMYD2**	SET and MYND domain containing 2
**SNAI2**	Snail family transcriptional repressor 2
**SNAPC3**	Small nuclear RNA activating complex polypeptide 3
**SNAPC5**	Small nuclear RNA activating complex polypeptide 5
**SNCA**	Synuclein alpha
**SND1-IT1**	SND1 intronic transcript 1
**SNHG1///SNORD22///SNORD25///SNORD26///SNORD27///SNORD28///SNORD30///SNORD31///SNORD29**	Small nucleolar RNA host gene 1///small nucleolar RNA, C/D box 22///small nucleolar RNA, C/D box 25///small nucleolar RNA, C/D box 26///small nucleolar RNA, C/D box 27///small nucleolar RNA, C/D box 28///small nucleolar RNA, C/D box 30///small nucleolar R
**SNHG4///MATR3**	Small nucleolar RNA host gene 4///matrin 3
**SNORA29///TCP1**	Small nucleolar RNA, H/ACA box 29///t-complex 1
**SNORD14D///SNORD14C///HSPA8**	Small nucleolar RNA, C/D box 14D///small nucleolar RNA, C/D box 14C///heat shock protein family A (Hsp70) member 8
**SNORD50B///SNORD50A**	Small nucleolar RNA, C/D box 50B///small nucleolar RNA, C/D box 50A
**SNORD77///SNORD76///SNORD74///GAS5///SNORD44///SNORD47///SNORD80///SNORD79///SNORD81**	Small nucleolar RNA, C/D box 77///small nucleolar RNA, C/D box 76///small nucleolar RNA, C/D box 74///growth arrest specific 5 (non-protein coding)///small nucleolar RNA, C/D box 44///small nucleolar RNA, C/D box 47///small nucleolar RNA, C/D box 80///sma
**SNORD89**	Small nucleolar RNA, C/D box 89
**SNRNP70**	Small nuclear ribonucleoprotein U1 subunit 70
**SNRPA1**	Small nuclear ribonucleoprotein polypeptide A'
**SNURF///SNRPN**	SNRPN upstream reading frame///small nuclear ribonucleoprotein polypeptide N
**SNX14**	Sorting nexin 14
**SNX2**	Sorting nexin 2
**SNX5**	Sorting nexin 5
**SOCS1**	Suppressor of cytokine signaling 1
**SON**	SON DNA binding protein
**SOX15**	SRY-box 15
**SP100**	SP100 nuclear antigen
**SP140L**	SP140 nuclear body protein like
**SP3**	Sp3 transcription factor
**SP5**	Sp5 transcription factor

EXCLI Journal 2021;20:126-141– ISSN 1611-2156
Received: December 08, 2020, accepted: January 11, 2021, published: January 18, 2021

**SPAG16**	Sperm associated antigen 16
**SPARC**	Secreted protein acidic and cysteine rich
**SPHAR///RAB4A**	S-phase response (cyclin related)///RAB4A, member RAS oncogene family
**SPIN2B///SPIN2A**	Spindlin family member 2B///spindlin family member 2A
**SPIN3**	Spindlin family member 3
**SPON1**	Spondin 1
**SPTB**	Spectrin beta, erythrocytic
**SPTBN1**	Spectrin beta, non-erythrocytic 1
**SRC**	SRC proto-oncogene, non-receptor tyrosine kinase
**SREK1**	Splicing regulatory glutamic acid and lysine rich protein 1
**SRGAP2**	SLIT-ROBO Rho gtpase activating protein 2
**SRPK2**	SRSF protein kinase 2
**SRPRB**	SRP receptor beta subunit
**SRRD**	SRR1 domain containing
**SRRT**	Serrate, RNA effector molecule
**SRSF10**	Serine and arginine rich splicing factor 10
**SRSF11**	Serine and arginine rich splicing factor 11
**SSBP1**	Single stranded DNA binding protein 1
**SSR3**	Signal sequence receptor subunit 3
**SSTR4**	Somatostatin receptor 4
**SSX3**	SSX family member 3
**ST3GAL3**	ST3 beta-galactoside alpha-2,3-sialyltransferase 3
**ST6GALNAC4**	ST6 N-acetylgalactosaminide alpha-2,6-sialyltransferase 4
**ST8SIA2**	ST8 alpha-N-acetyl-neuraminide alpha-2,8-sialyltransferase 2
**STAG3L2///STAG3L3///TRIM73**	Stromal antigen 3-like 2 (pseudogene)///stromal antigen 3-like 3 (pseudogene)///tripartite motif containing 73
**STAMBPL1**	STAM binding protein like 1
**STC2**	Stanniocalcin 2
**STEAP4**	STEAP4 metalloreductase
**STIM1**	Stromal interaction molecule 1
**STK38**	Serine/threonine kinase 38
**STK40**	Serine/threonine kinase 40
**STMN3**	Stathmin 3
**STPG3**	Sperm-tail PG-rich repeat containing 3
**STRADB**	STE20-related kinase adaptor beta
**STRBP**	Spermatid perinuclear RNA binding protein
**STRING**	Search Toll for the Retrieval of Interacting Genes
**STRN3**	Striatin 3
**STX17-AS1**	STX17 antisense RNA 1
**STYX**	Serine/threonine/tyrosine interacting protein
**SUGP2**	SURP and G-patch domain containing 2
**SUGT1**	SGT1 homolog, MIS12 kinetochore complex assembly cochaperone
**SUN1**	Sad1 and UNC84 domain containing 1

EXCLI Journal 2021;20:126-141– ISSN 1611-2156
Received: December 08, 2020, accepted: January 11, 2021, published: January 18, 2021

**SUPT3H**	SPT3 homolog, SAGA and STAGA complex component
**SUPT7L**	SPT7-like STAGA complex gamma subunit
**SUZ12P1**	SUZ12 polycomb repressive complex 2 subunit pseudogene 1
**SVIL**	Supervillin
**SVIP**	Small VCP interacting protein
**SWAP70**	SWAP switching B-cell complex 70kda subunit
**SWI5**	SWI5 homologous recombination repair protein
**SYNE1**	Spectrin repeat containing nuclear envelope protein 1
**SYNE2**	Spectrin repeat containing nuclear envelope protein 2
**SYNJ2BP-COX16///SYNJ2BP**	SYNJ2BP-COX16 readthrough///synaptojanin 2 binding protein
**SYT17**	Synaptotagmin 17
**TAC3**	Tachykinin 3
**TACR2**	Tachykinin receptor 2
**TAF1**	TATA-box binding protein associated factor 1
**TAF11**	TATA-box binding protein associated factor 11
**TAF3**	TATA-box binding protein associated factor 3
**TAF4B**	TATA-box binding protein associated factor 4b
**TAF9B**	TATA-box binding protein associated factor 9b
**TAGAP**	T-cell activation rhogtpase activating protein
**TAL1**	TAL bhlh transcription factor 1, erythroid differentiation factor
**TAMM41**	TAM41 mitochondrial translocator assembly and maintenance homolog
**TARBP1**	TAR (HIV-1) RNA binding protein 1
**TARSL2**	Threonyl-trna synthetase like 2
**TAS2R14**	Taste 2 receptor member 14
**TASP1**	Taspase 1
**TATDN1**	Tatd dnase domain containing 1
**TBC1D10C**	TBC1 domain family member 10C
**TBC1D22B**	TBC1 domain family member 22B
**TBC1D31**	TBC1 domain family member 31
**TBC1D32**	TBC1 domain family member 32
**TBC1D4**	TBC1 domain family member 4
**TBCK**	TBC1 domain containing kinase
**TBL1XR1**	Transducin (beta)-like 1 X-linked receptor 1
**TBL1XR1**	Transducin beta like 1 X-linked receptor 1
**TBRG1**	Transforming growth factor beta regulator 1
**TC2N**	Tandem C2 domains, nuclear
**TCF12**	Transcription factor 12
**TCF3**	Transcription factor 3
**TCF4**	Transcription factor 4
**TCF7**	Transcription factor 7 (T-cell specific, HMG-box)
**TCL1A**	T-cell leukemia/lymphoma 1A
**TCL6**	T-cell leukemia/lymphoma 6 (non-protein coding)
**TCP11L2**	T-complex 11 like 2

EXCLI Journal 2021;20:126-141– ISSN 1611-2156
Received: December 08, 2020, accepted: January 11, 2021, published: January 18, 2021

**TDG**	Thymine DNA glycosylase
**TECTA**	Tectorin alpha
**TEKT4P2///MAFIP///LOC389834**	Tektin 4 pseudogene 2///MAFF interacting protein (pseudogene)///ankyrin repeat domain 57 pseudogene
**TEN1-CDK3///CDK3**	TEN1-CDK3 readthrough (NMD candidate)///cyclin dependent kinase 3
**TENM1**	Teneurin transmembrane protein 1
**TF**	Serotransferrin
**TF**	Transferrin
**TFAM**	Transcription factor A, mitochondrial
**TFDP2**	Transcription factor Dp-2
**TFEC**	Transcription factor EC
**TFRC**	Transferrin receptor
**TFRC**	Transferrin receptor
**TGFA**	Transforming growth factor alpha
**TGM3**	Transglutaminase 3
**TGOLN2**	Trans-golgi network protein 2
**THAP9-AS1**	THAP9 antisense RNA 1
**THBD**	Thrombomodulin
**THEM4**	Thioesterase superfamily member 4
**THOC2**	THO complex 2
**THSD4**	Thrombospondin type 1 domain containing 4
**THUMPD3**	THUMP domain containing 3
**TIA1**	TIA1 cytotoxic granule-associated RNA binding protein
**TIAL1**	TIA1 cytotoxic granule-associated RNA binding protein-like 1
**TIAM1**	T-cell lymphoma invasion and metastasis 1
**TIGD1**	Tigger transposable element derived 1
**TIGD7**	Tigger transposable element derived 7
**TIMM17A**	Translocase of inner mitochondrial membrane 17 homolog A (yeast)
**TIMM21**	Translocase of inner mitochondrial membrane 21
**TIMM9**	Translocase of inner mitochondrial membrane 9
**TKTL1**	Transketolase like 1
**TLE1**	Transducin like enhancer of split 1
**TLR4**	Toll like receptor 4
**TLR8**	Toll like receptor 8
**TLX2**	T-cell leukemia homeobox 2
**TM2D1**	TM2 domain containing 1
**TM6SF1**	Transmembrane 6 superfamily member 1
**TM7SF2**	Transmembrane 7 superfamily member 2
**TMCC3**	Transmembrane and coiled-coil domain family 3
**TMED2**	Transmembrane p24 trafficking protein 2
**TMED2**	Transmembrane p24 trafficking protein 2
**TMED5**	Transmembrane p24 trafficking protein 5
**TMEM116**	Transmembrane protein 116
**TMEM144**	Transmembrane protein 144

EXCLI Journal 2021;20:126-141– ISSN 1611-2156
Received: December 08, 2020, accepted: January 11, 2021, published: January 18, 2021

**TMEM168**	Transmembrane protein 168
**TMEM174**	Transmembrane protein 174
**TMEM260**	Transmembrane protein 260
**TMEM262**	Transmembrane protein 262
**TMEM267**	Transmembrane protein 267
**TMEM59**	Transmembrane protein 59
**TMEM63A**	Transmembrane protein 63A
**TMEM63B**	Transmembrane protein 63B
**TMTC3**	Transmembrane and tetratricopeptide repeat containing 3
**TNFAIP1**	TNF alpha induced protein 1
**TNFAIP6**	TNF alpha induced protein 6
**TNFRSF10A**	TNF receptor superfamily member 10a
**TNFRSF10C**	TNF receptor superfamily member 10c
**TNFRSF11B**	TNF receptor superfamily member 11b
**TNFRSF12A**	TNF receptor superfamily member 12A
**TNKS2**	Tankyrase 2
**TNNT3**	Troponin T3, fast skeletal type
**TNPO2**	Transportin 2
**TNRC18**	Trinucleotide repeat containing 18
**TNRC6A**	Trinucleotide repeat containing 6A
**TNRC6C**	Trinucleotide repeat containing 6C
**TNS1**	Tensin 1
**TOB1**	Transducer of ERBB2, 1
**TOP2A**	Topoisomerase (DNA) II alpha
**TOX3**	TOX high mobility group box family member 3
**TP73-AS1**	TP73 antisense RNA 1
**TPD52**	Tumor protein D52
**TPSAB1**	Tryptase alpha/beta 1
**TRA2A**	Transformer 2 alpha homolog
**TRA2B**	Transformer 2 beta homolog (Drosophila)
**TRAF3IP3**	TRAF3 interacting protein 3
**TRAF5**	TNF receptor associated factor 5
**TRAPPC9**	Trafficking protein particle complex 9
**TRIB1**	Tribbles pseudokinase 1
**TRIM10**	Tripartite motif containing 10
**TRIM15**	Tripartite motif containing 15
**TRIM23**	Tripartite motif containing 23
**TRIM25**	Tripartite motif containing 25
**TRIM4**	Tripartite motif containing 4
**TRIM5**	Tripartite motif containing 5
**TRIM58**	Tripartite motif containing 58
**TRIP11**	Thyroid hormone receptor interactor 11
**TRMT11**	Trna methyltransferase 11 homolog
**TRMT61B**	Trna methyltransferase 61B

EXCLI Journal 2021;20:126-141– ISSN 1611-2156
Received: December 08, 2020, accepted: January 11, 2021, published: January 18, 2021

**TRUB1**	Trub pseudouridine synthase family member 1
**TSC22D2**	TSC22 domain family member 2
**TSPAN13**	Tetraspanin 13
**TSPAN5**	Tetraspanin 5
**TSPAN7**	Tetraspanin 7
**TSPOAP1-AS1**	TSPOAP1 antisense RNA 1
**TSR1**	TSR1, ribosome maturation factor
**TTBK1**	Tau tubulin kinase 1
**TTBK2**	Tau tubulin kinase 2
**TTC13**	Tetratricopeptide repeat domain 13
**TTC14**	Tetratricopeptide repeat domain 14
**TTC25**	Tetratricopeptide repeat domain 25
**TTC28-AS1**	TTC28 antisense RNA 1
**TTC37**	Tetratricopeptide repeat domain 37
**TTC39B**	Tetratricopeptide repeat domain 39B
**TTC39C**	Tetratricopeptide repeat domain 39C
**TTC3P1///TTC3**	Tetratricopeptide repeat domain 3 pseudogene 1///tetratricopeptide repeat domain 3
**TTF2**	Transcription termination factor 2
**TTI1**	TELO2 interacting protein 1
**TTLL5**	Tubulin tyrosine ligase like 5
**TUBA4A**	Tubulin alpha 4a
**TUBE1**	Tubulin epsilon 1
**TUBGCP6**	Tubulin gamma complex associated protein 6
**TUG1**	Taurine up-regulated 1 (non-protein coding)
**TULP4**	Tubby like protein 4
**TWISTNB**	TWIST neighbor
**TXK**	TXK tyrosine kinase
**TXLNG**	Taxilin gamma
**TXNDC16**	Thioredoxin domain containing 16
**TXNL1**	Thioredoxin like 1
**U2SURP**	U2 snrnp associated SURP domain containing
**UBA2**	Ubiquitin like modifier activating enzyme 2
**UBASH3A**	Ubiquitin associated and SH3 domain containing A
**UBE2J2**	Ubiquitin conjugating enzyme E2 J2
**UBE3A**	Ubiquitin protein ligase E3A
**UBN2**	Ubinuclein 2
**UBTF**	Upstream binding transcription factor, RNA polymerase I
**UBTF**	Upstream binding transcription factor, RNA polymerase I
**UGGT2**	UDP-glucose glycoprotein glucosyltransferase 2
**UNC13D**	Unc-13 homolog D
**UPF2**	UPF2 regulator of nonsense transcripts homolog (yeast)
**UPF3A**	UPF3 regulator of nonsense transcripts homolog A (yeast)
**UPF3B**	UPF3 regulator of nonsense transcripts homolog B (yeast)
**UQCC1**	Ubiquinol-cytochrome c reductase complex assembly factor 1

EXCLI Journal 2021;20:126-141– ISSN 1611-2156
Received: December 08, 2020, accepted: January 11, 2021, published: January 18, 2021

**URGCP**	Upregulator of cell proliferation
**USP10**	Ubiquitin specific peptidase 10
**USP12**	Ubiquitin specific peptidase 12
**USP25**	Ubiquitin specific peptidase 25
**USP28**	Ubiquitin specific peptidase 28
**USP45**	Ubiquitin specific peptidase 45
**USP47**	Ubiquitin specific peptidase 47
**USP48**	Ubiquitin specific peptidase 48
**USP53**	Ubiquitin specific peptidase 53
**USP8**	Ubiquitin specific peptidase 8
**USP9Y**	Ubiquitin specific peptidase 9, Y-linked
**USPL1**	Ubiquitin specific peptidase like 1
**UVSSA**	UV stimulated scaffold protein A
**VAMP1**	Vesicle associated membrane protein 1
**VAMP3**	Vesicle associated membrane protein 3
**VAMP4**	Vesicle associated membrane protein 4
**VAV2**	Vav guanine nucleotide exchange factor 2
**VCAN**	Versican
**VCL**	Vinculin
**VDAC1**	Voltage dependent anion channel 1
**VEGFA**	Vascular endothelial growth factor A
**VMO1**	Vitelline membrane outer layer 1 homolog
**VNN3**	Vanin 3
**VPS13A**	Vacuolar protein sorting 13 homolog A
**VPS13B**	Vacuolar protein sorting 13 homolog B
**VPS13D**	Vacuolar protein sorting 13 homolog D
**VPS13D**	Vacuolar protein sorting 13 homolog D
**VPS36**	Vacuolar protein sorting 36 homolog
**VPS45**	Vacuolar protein sorting 45 homolog
**VPS50**	VPS50, EARP/GARPII complex subunit
**VPS54**	VPS54, GARP complex subunit
**VRK1**	Vaccinia related kinase 1
**VSTM2L**	V-set and transmembrane domain containing 2 like
**VWA5B1**	Von Willebrand factor A domain containing 5B1
**VWDE**	Von Willebrand factor D and EGF domains
**WARS**	Tryptophanyl-trna synthetase
**WDFY3-AS2**	WDFY3 antisense RNA 2
**WDR11**	WD repeat domain 11
**WDR19**	WD repeat domain 19
**WDR20**	WD repeat domain 20
**WDR27**	WD repeat domain 27
**WDR75**	WD repeat domain 75
**WEE1**	WEE1 G2 checkpoint kinase
**WFDC1**	WAP four-disulfide core domain 1

EXCLI Journal 2021;20:126-141– ISSN 1611-2156
Received: December 08, 2020, accepted: January 11, 2021, published: January 18, 2021

**WHAMMP2///WHAMMP3**	WAS protein homolog associated with actin, golgi membranes and microtubules pseudogene 2///WAS protein homolog associated with actin, golgi membranes and microtubules pseudogene 3
**WIPI1**	WD repeat domain, phosphoinositide interacting 1
**WLS**	Wntless Wnt ligand secretion mediator
**WNT5B**	Wnt family member 5B
**WNT7B**	Wnt family member 7B
**WRAP73**	WD repeat containing, antisense to TP73
**WSCD2**	WSC domain containing 2
**XAF1**	XIAP associated factor 1
**XPNPEP1**	X-prolyl aminopeptidase 1
**XPNPEP3**	X-prolyl aminopeptidase 3
**XPO7**	Exportin 7
**XPOT**	Exportin for trna
**XRCC5**	X-ray repair cross complementing 5
**XRN1**	5'-3' exoribonuclease 1
**YBX3**	Y-box binding protein 3
**YIPF4**	Yip1 domain family member 4
**YIPF5**	Yip1 domain family member 5
**YLPM1**	YLP motif containing 1
**yoSYT12**	Synaptotagmin 12
**YTHDC2**	YTH domain containing 2
**YWHAZ**	Tyrosine 3-monooxygenase/tryptophan 5-monooxygenase activation protein zeta
**ZADH2**	Zinc binding alcohol dehydrogenase domain containing 2
**ZAK**	Sterile alpha motif and leucine zipper containing kinase AZK
**ZBED5**	Zinc finger BED-type containing 5
**ZBTB1**	Zinc finger and BTB domain containing 1
**ZBTB20**	Zinc finger and BTB domain containing 20
**ZBTB24**	Zinc finger and BTB domain containing 24
**ZBTB26**	Zinc finger and BTB domain containing 26
**ZBTB41**	Zinc finger and BTB domain containing 41
**ZC2HC1A**	Zinc finger C2HC-type containing 1A
**ZC3H12D**	Zinc finger CCCH-type containing 12D
**ZC3H13**	Zinc finger CCCH-type containing 13
**ZC3H14**	Zinc finger CCCH-type containing 14
**ZC3H6**	Zinc finger CCCH-type containing 6
**ZC3H8**	Zinc finger CCCH-type containing 8
**ZCCHC10**	Zinc finger CCHC-type containing 10
**ZCCHC7**	Zinc finger CCHC-type containing 7
**ZCRB1**	Zinc finger CCHC-type and RNA binding motif containing 1
**ZDHHC11B///ZDHHC11**	Zinc finger DHHC-type containing 11B///zinc finger DHHC-type containing 11
**ZDHHC17**	Zinc finger DHHC-type containing 17
**ZDHHC2**	Zinc finger DHHC-type containing 2

EXCLI Journal 2021;20:126-141– ISSN 1611-2156
Received: December 08, 2020, accepted: January 11, 2021, published: January 18, 2021

**ZDHHC22**	Zinc finger DHHC-type containing 22
**ZDHHC6**	Zinc finger DHHC-type containing 6
**ZEB1**	Zinc finger E-box binding homeobox 1
**ZER1**	Zyg-11 related cell cycle regulator
**ZFP3**	ZFP3 zinc finger protein
**ZFP57**	ZFP57 zinc finger protein
**ZFP69**	ZFP69 zinc finger protein
**ZFP90**	ZFP90 zinc finger protein
**ZFYVE21**	Zinc finger FYVE-type containing 21
**ZFYVE28**	Zinc finger FYVE-type containing 28
**ZHX1**	Zinc fingers and homeoboxes 1
**ZHX2**	Zinc fingers and homeoboxes 2
**ZIC1**	Zic family member 1
**ZKSCAN1**	Zinc finger with KRAB and SCAN domains 1
**ZKSCAN7**	Zinc finger with KRAB and SCAN domains 7
**ZKSCAN8**	Zinc finger with KRAB and SCAN domains 8
**ZMAT1**	Zinc finger matrin-type 1
**ZMYM1**	Zinc finger MYM-type containing 1
**ZMYM2**	Zinc finger MYM-type containing 2
**ZMYM4**	Zinc finger MYM-type containing 4
**ZMYM5**	Zinc finger MYM-type containing 5
**ZMYM6**	Zinc finger MYM-type containing 6
**ZMYND11**	Zinc finger MYND-type containing 11
**ZMYND8**	Zinc finger MYND-type containing 8
**ZNF101**	Zinc finger protein 101
**ZNF107**	Zinc finger protein 107
**ZNF124**	Zinc finger protein 124
**ZNF137P**	Zinc finger protein 137, pseudogene
**ZNF138**	Zinc finger protein 138
**ZNF140**	Zinc finger protein 140
**ZNF207**	Zinc finger protein 207
**ZNF224**	Zinc finger protein 224
**ZNF226**	Zinc finger protein 226
**ZNF235**	Zinc finger protein 235
**ZNF248**	Zinc finger protein 248
**ZNF26**	Zinc finger protein 26
**ZNF260**	Zinc finger protein 260
**ZNF268**	Zinc finger protein 268
**ZNF286B///ZNF286A**	Zinc finger protein 286B///zinc finger protein 286A
**ZNF292**	Zinc finger protein 292
**ZNF300**	Zinc finger protein 300
**ZNF302**	Zinc finger protein 302(ZNF302)
**ZNF302**	Zinc finger protein 302

EXCLI Journal 2021;20:126-141– ISSN 1611-2156
Received: December 08, 2020, accepted: January 11, 2021, published: January 18, 2021

**ZNF320**	Zinc finger protein 320
**ZNF326**	Zinc finger protein 326
**ZNF337**	Zinc finger protein 337
**ZNF33A**	Zinc finger protein 33A
**ZNF354A**	Zinc finger protein 354A
**ZNF382**	Zinc finger protein 382
**ZNF404**	Zinc finger protein 404
**ZNF414**	Zinc finger protein 414
**ZNF425**	Zinc finger protein 425
**ZNF431**	Zinc finger protein 431
**ZNF432**	Zinc finger protein 432
**ZNF439**	Zinc finger protein 439
**ZNF468**	Zinc finger protein 468
**ZNF493**	Zinc finger protein 493
**ZNF493**	Zinc finger protein 493
**ZNF506**	Zinc finger protein 506
**ZNF507**	Zinc finger protein 507
**ZNF512B**	Zinc finger protein 512B
**ZNF518A**	Zinc finger protein 518A
**ZNF529**	Zinc finger protein 529
**ZNF536**	Zinc finger protein 536
**ZNF542P**	Zinc finger protein 542, pseudogene
**ZNF544**	Zinc finger protein 544
**ZNF548**	Zinc finger protein 548
**ZNF550**	Zinc finger protein 550
**ZNF551**	Zinc finger protein 551
**ZNF558**	Zinc finger protein 558
**ZNF561///ZNF562**	Zinc finger protein 561///zinc finger protein 562
**ZNF566**	Zinc finger protein 566
**ZNF568**	Zinc finger protein 568
**ZNF57**	Zinc finger protein 57
**ZNF585B**	Zinc finger protein 585B
**ZNF587B**	Zinc finger protein 587B
**ZNF621**	Zinc finger protein 621
**ZNF638**	Zinc finger protein 638
**ZNF652**	Zinc finger protein 652
**ZNF653**	Zinc finger protein 653
**ZNF667-AS1**	ZNF667 antisense RNA 1 (head to head)
**ZNF677**	Zinc finger protein 677
**ZNF680**	Zinc finger protein 680
**ZNF682**	Zinc finger protein 682
**ZNF708**	Zinc finger protein 708
**ZNF709**	Zinc finger protein 709

EXCLI Journal 2021;20:126-141– ISSN 1611-2156
Received: December 08, 2020, accepted: January 11, 2021, published: January 18, 2021

**ZNF721**	Zinc finger protein 721
**ZNF736**	Zinc finger protein 736
**ZNF75D**	Zinc finger protein 75D
**ZNF766**	Zinc finger protein 766
**ZNF767P**	Zinc finger family member 767, pseudogene
**ZNF768///ZNF747**	Zinc finger protein 768///zinc finger protein 747
**ZNF776**	Zinc finger protein 776
**ZNF777**	Zinc finger protein 777
**ZNF782**	Zinc finger protein 782
**ZNF813**	Zinc finger protein 813
**ZNF823**	Zinc finger protein 823
**ZNF827**	Zinc finger protein 827
**ZNF83**	Zinc finger protein 83
**ZNF84**	Zinc finger protein 84
**ZNHIT6**	Zinc finger HIT-type containing 6
**ZRANB2**	Zinc finger RANBP2-type containing 2
**ZSCAN21**	Zinc finger and SCAN domain containing 21
**ZSWIM8**	Zinc finger SWIM-type containing 8
**ZUFSP**	Zinc finger with UFM1 specific peptidase domain
**ZXDB**	Zinc finger, X-linked, duplicated B
**ZZZ3**	Zinc finger ZZ-type containing 3
